# Genetic analysis of DNA methylation and gene expression levels in whole blood of healthy human subjects

**DOI:** 10.1186/1471-2164-13-636

**Published:** 2012-11-17

**Authors:** Kristel R van Eijk, Simone de Jong, Marco PM Boks, Terry Langeveld, Fabrice Colas, Jan H Veldink, Carolien GF de Kovel, Esther Janson, Eric Strengman, Peter Langfelder, René S Kahn, Leonard H van den Berg, Steve Horvath, Roel A Ophoff

**Affiliations:** 1 Department of Medical Genetics, University Medical Center Utrecht, Utrecht, 3584, CG, The Netherlands; 2 Department of Psychiatry, Rudolf Magnus Institute of Neuroscience, University Medical Center Utrecht, Utrecht, 3508, GA, The Netherlands; 3 Department of Medical Statistics and Bioinformatics, Leiden University Medical Center, Leiden, 2300, RC, The Netherlands; 4 Department of Neurology, Rudolf Magnus Institute of Neuroscience, University Medical Center Utrecht, Utrecht, 3508, GA, The Netherlands; 5 Center for Neurobehavioral Genetics, University of California Los Angeles, Box 951761 Gonda #4357C, 695 Charles E. Young Drive, South Los Angeles, CA 90095-1761, USA; 6 Department of Human Genetics, David Geffen School of Medicine, University of California, Los Angeles, CA, 90095, USA; 7 Department of Biostatistics, School of Public Health, University of California, Los Angeles, CA, 90095, USA

**Keywords:** DNA methylation, Gene expression, Association, Epigenetics, WGCNA

## Abstract

**Background:**

The predominant model for regulation of gene expression through DNA methylation is an inverse association in which increased methylation results in decreased gene expression levels. However, recent studies suggest that the relationship between genetic variation, DNA methylation and expression is more complex.

**Results:**

Systems genetic approaches for examining relationships between gene expression and methylation array data were used to find both negative and positive associations between these levels. A weighted correlation network analysis revealed that i) both transcriptome and methylome are organized in modules, ii) co-expression modules are generally not preserved in the methylation data and vice-versa, and iii) highly significant correlations exist between co-expression and co-methylation modules, suggesting the existence of factors that affect expression and methylation of different modules (i.e., *trans* effects at the level of modules). We observed that methylation probes associated with expression in *cis* were more likely to be located outside CpG islands, whereas specificity for CpG island shores was present when methylation, associated with expression, was under local genetic control. A structural equation model based analysis found strong support in particular for a traditional causal model in which gene expression is regulated by genetic variation via DNA methylation instead of gene expression affecting DNA methylation levels.

**Conclusions:**

Our results provide new insights into the complex mechanisms between genetic markers, epigenetic mechanisms and gene expression. We find strong support for the classical model of genetic variants regulating methylation, which in turn regulates gene expression. Moreover we show that, although the methylation and expression modules differ, they are highly correlated.

## Background

Epigenetics has been described as the structural adaptation of chromosomal regions so as to register, signal or perpetuate altered activity states [1]. DNA methylation is one of several forms of epigenetic modifications and involves the covalent binding of a methyl group to a Cytosine-5 at a C-phosphate-G (CpG) site. These sites are relatively rare in the genome but more common at promoter regions of genes, also called CpG islands (CGIs). CpGs in these islands are less likely to be methylated than CpGs outside these islands. Recent studies have shown that specifically the CpGs in the shore of CGIs are most frequently involved in differential methylation between tissues or experimental groups [2,3]. Increased methylation of CpG islands at 5’ end of a gene is associated with gene repression. Possible mechanisms for repression include interference with transcription factor binding or through the recruitment of repressors such as histone deacetylases [4].

Although one would expect DNA methylation at CGIs and expression of the nearby gene to be inversely correlated, this is not necessarily the case. Recent reports also identified positive associations between expression and methylation levels [5-7]. However, negative associations between methylation and expression were found to be enriched particularly in CGIs [6] and promoter regions [5].

Around 30% of gene expression levels in cell lines [8] and 23% of DNA methylation levels in blood are heritable [9] and genetic variation associated with expression and methylation levels has been identified in several organisms [6,10-12], tissues [13] and populations [14]. Local (*cis*) and distal (*trans*) associations of genetic variation with gene expression levels have been observed. With the arrival of high-throughput DNA methylation assays, methylation quantitative trait loci (mQTLs) can now be studied genome-wide in any tissue or cell type of interest. Similar to expression (eQTLs), more *cis* than *trans* regulation has been identified [5-7] but peak enrichment for mQTLs is located in much closer proximity to transcription start sites than that of eQTLs [6].

Attempts to identify three-way associations between genetic variants, expression and methylation on a genome-wide scale in four different brain regions did not identify co-regulation of methylation and expression by the same genetic variants [6], while a study of cerebellar samples did identify three-way associations for a number of genes [7]. In lymphoblastoid cell lines of 77 individuals of the Yoruba Hapmap population, co-regulation of expression and methylation levels by the same genetic variants was also found, suggesting a shared mechanism, whereby a genetic variant influences methylation, which in turn influences expression levels [5]. Strong evidence exists that both patterns of CpG methylation [15,16] and gene expression [13,17,18] differ between tissues.

The aims of the current study are i) to relate expression levels to methylation levels, ii) to relate co-expression modules (clusters of expression probes) to co-methylation modules, iii) and to study the relationship between genetic markers, methylation and expression in whole blood of a relatively large (n=148) set of healthy human subjects. For the genetic analysis, we examined the associations of methylation and expression levels and identified genetic markers associated with these levels. To infer directionality in the relationships between genetic variants, methylation and expression, we calculated local edge orienting (LEO) scores based on structural equation models [19]. This method has been applied successfully before and will aid in elucidating the nature of relationship between genetic variation, methylation and expression [20-23].

## Results

### Associations between methylation and expression levels

A multivariate linear model analysis for regressing a gene expression level on a methylation level and age and gender resulted in the identification of 522 negative and 276 positive *cis* associations between methylation and expression levels (False Discovery Rate (FDR) 5% corrected). A negative association between methylation and transcript level means that increased methylation levels correlate with decreased expression levels, whereas a positive correlation includes levels that both increase or decrease. These associations involved 517 different *cis*-acting CpG loci (from 461 unique genes) and 495 corresponding expression probes (representing 452 unique genes). For *trans* effects, we found evidence for 844 negative and 1,806 positive associations between methylation and expression levels involving 705 different methylation probes (from 630 distinct genes), and 170 different expression probes (representing 157 unique genes). Full results are given in Table 1 and Additional file 1: Table S1. Because of the stringent Bonferroni corrections for multiple testing with the number of methylation probes multiplied by the number of expression probes, the effect sizes of surviving *trans* effects were significantly larger than for *cis* effects with adjusted explained variance (R^2^) ranging from 23 to 60 percent for *trans* effects and 0.8 to 50 percent for *cis* regulation (Additional file 2: Figure S1a). Another trend that we observed was that *cis* effects are enriched for negative correlations (65.4% overall) while positive correlations between DNA methylation and gene expression are more frequently observed with *trans* associations (68.2%; Fisher’s Exact test for count data *p*<2.2e-16), (Additional file 2: Figure S1b).

**Table 1:** Number of probes constituting significant methylation and expression combinations and their association with SNPs

	**unique**	+	-	**overlap**	**SNP *cis***
				*Cis * associations	
**Methylation**	517	224	354	61	69 probes (13.3%), 86 independent loci
**Expression**	495	214	336	55	62 probes (12.5%), 73 independent loci
				*Trans * associations	
**Methylation**	705	585	230	110	1 probe (<1%)
**Expression**	170	101	117	48	0 probes

This table shows the significant methylation and expression combinations, subdivided into *cis* and *trans* associations. The first column shows the counts of unique probes (for methylation and expression). The second and third columns indicate the number of probes positively (+) or negatively (−) associated. The fourth column indicates the overlapping probes: methylation or expression probes that are associated with expression or methylation levels in both directions. The last column indicates the number (and %) of unique probes associated with SNPs and the number of independent (pruned r^2^ of 0.2) loci in *cis*.

### DNA methylation and gene expression are regulated by genetic variants

Expression levels and methylation levels that were significantly associated with each other were separately tested for regulation by genetic variants. The methylation and expression levels were taken as phenotypes and a linear model of allele dosage, with age and gender as covariates, was tested using PLINK [24]. We focused on local (*cis*) effects only and observed that approximately 13.7% of methylation signals and 12.5% of gene expression levels are associated with single nucleotide polymorphisms (SNPs). Results are given in Table 1, where the number of independent loci, associated with probes, is reported. These were retrieved by pruning the SNPs with an R^2^ of >0.2 to prevent reporting many SNP associations of the same signal due to linkage disequilibrium (LD). Full results are in Additional file 3: Table S2.

### *Cis*-acting methylation sites under genetic control are over-represented in CpG island shores

We examined the regional distribution of methylation sites (n=517) that are associated with nearby gene expression levels and observed a significant overrepresentation of these loci outside CpG islands and shores compared to all probes present on the Illumina array (50.9% vs 26%; Fisher’s Exact *p*<2.2e-16). This coincided with a significant underrepresentation of DNA methylation signal at CpG islands (13.5% vs. 42%, Fisher’s Exact *p*<2.2e-16) and a modest increase at the shores flanking CpG islands (35.6% vs. 32%, Fisher’s Exact *p*=0.056). The regional distribution of DNA methylation associated with gene expression is somewhat different when DNA methylation is under genetic control. In case of *cis* genetic regulation we observed a further enrichment of DNA methylation at shores of CpG islands (53.4%, Fisher’s Exact *p*=1.3e-4), whereas *trans* genetic regulation shows the opposite effect and is less frequently observed for DNA methylation at shores (24.4%, Fisher’s Exact *p*=3.9e-5). The overall results are presented in Table 2.

**Table 2:** Distribution of results over CpG islands and shores

**Location**	**Illumina Human Methylation 27**		**Methylation & expression *cis***		**Methylation & expression *trans***		**Methylation & expression & SNP *cis***	
**Island**	11,582	42%	**70**	**13.5%, *p*** **<2.2e-16↓**	269	38.2%, *p*=0.04	**11**	**15.1%, *p*** **=1.1e-06↓**
**Island shore (2kb)**	8,718	32%	184	35.6%, *p*=0.056	**172**	**24.4%, *p*** **=3.9e-05↓**	**39**	**53.4%, *p*** **=1.3e-04↑**
**Outside island/shore**	7,278	26%	**263**	**50.9%, *p*** **<2.2e-16↑**	**264**	**37.4%, *p*** **=2.5e-10↑**	23	31.5%, ns
**Total**	27,578		517		705		73	

Methylation probes were classified into three categories according to UCSC browser ( http://genome.ucsc.edu/); CpG islands, CGI shores (up to 2kb around an island) and outside islands or shores. Differences compared to Illumina Human Methylation 27K array were tested using Fisher’s Exact for count data (Bonferroni threshold *p*:0.05/9=0.006). A downward arrow indicates significantly lower percentage of probes while an upward arrow indicates significantly higher percentage of observations compared to the overall probe distribution on the Illumina array.

### Causal relationships between *cis*-acting methylation and expression probes

To study the causal relationship between methylation and expression levels that were significantly associated we focused the analysis on pairs of methylation and expression levels with a common *cis*-acting SNP. We selected the top 20 methylation probes, associated with 19 expression probes that were significantly associated with 147 single common SNPs. Since alleles can be considered fixed features of a genome, we selected SNPs as causal anchors and used a model with residuals of the 20 methylation and 19 expression probes corrected for age and gender. For the causal scenario SNP → Methylation → Expression, we found 44 combinations (29.9%) with a LEO score above 0.8, involving seven unique genes (Table 3). Of these, 20 combinations have a strikingly high LEO score of 3 or higher; for most of these 20 combinations, the model fitting p-value of the causal model SNP → Methylation → Expression is above 0.01, indicating a good fit and lending further credence to these results (Additional file 4: Table S3). For the model SNP → Expression → Methylation, we found 10 combinations (6.8%) with a LEO score above 0.8, involving again seven unique genes (Table 3). The model fitting p-values of these combinations are generally worse (below 0.01), indicating that the linear structural equations models do not fit the data as well and suggesting caution in interpreting the results. A full list of combinations is given in Additional file 4: Table S3. Some SNPs were found to be in high linkage disequilibrium (LD), especially in the Major Histocompatibility Complex (MHC) region on chromosome 6. Therefore only the top SNPs are listed in Table 3. We chosen to investigate these two models since we were interested in the causal direction between DNA methylation and gene expression, after regulation by genetic variation, excluding models 4 and 5. Model 3, was not informative since we already selected SNPs for association with both methylation and expression.

**Table 3:** Top results LEO analysis, Results for top SNPs

**Gene symbol**	**M&E**	**CGI**	**LEO model**	**LEO score**	**P-value**	**Top SNP**	**Chr**	**Bp**	**Full name**
***BTN3A2***	-	Outside	S → M → E	6.90	0.15	rs2093169	6	26,603,078	butyrophilin, subfamily 3, member A2
***HP***	-	Outside	S → M → E	4.24	0.82	rs8044555	16	70,710,256	haptoglobin
***CTSW***	-	Outside	S → M → E	2.73	0.13	rs11227306	11	65,335,248	cathepsin W
***NAPRT1***	-	Shore	S → M → E	2.69	0.11	rs4874159	8	144,742,093	nicotinate phosphoribosyltransferase domain containing 1
***PHACS***	-	Shore	S → M → E	1.50	2.9e-03	rs4755227	11	44,078,659	1-aminocyclopropane-1-carboxylate synthase homolog
***PNMA3***	+	Shore	S → M → E	1.36	0.16	rs6627737	X	151,971,610	nicotinate phosphoribosyltransferase domain containing 1
***CDC16***	-	Island	S → M → E	1.09	0.01	rs11147317	13	113,957,498	cell division cycle 16 homolog (S. cerevisiae)
***HRASLS3***	-	Shore	S → E → M	2.42	7.2e-04	rs2030731	11	63,130,224	phospholipase A2, group XVI
***TACSTD2***	-	Island	S → E → M	2.08	9.5e-03	rs11207272	1	58,846,018	tumor-associated calcium signal transducer 2
***SRXN1***	-	Shore	S → E → M	1.87	5.4e-03	rs6076864	20	569,825	sulfiredoxin 1 homolog (S. cerevisiae)
***C21orf56***	-	Outside	S → E → M	1.30	2.8e-03	rs8133866	21	46,423,604	chromosome 21 open reading frame 56
***BTN3A2***	-	Outside	S → E → M	1.14	6.4e-05	rs12199613	6	26,475,197	butyrophilin, subfamily 3, member A2
***WBSCR27***	-	Shore	S → E → M	0.95	8.6e-04	rs11763011	7	72,922,084	Williams Beuren syndrome chromosome region 27
***GSTM3***	-	Island	S → E → M	0.88	1.4e-03	rs11807	1	110,062,265	glutathione S-transferase mu 3 (brain)

This table contains top probes resulting from causality analysis (LEO scores > 0.8). The top seven genes fit the causal scenario SNP → Methylation → Expression (S→M→E), while the bottom seven genes fit the reverse model in which DNA methylation is regulated by gene expression that is under genetic control (S→E→M). The Gene Symbol is given in the first column. The second column indicates whether the methylation and expression levels are associated negatively (−) or positively (+). The third column indicates whether the methylation probe is located in a CpG island (CGI), in the shore, or outside both. The columns “LEO model”, “LEO score” and “P-value” indicate which causal model fits best with the corresponding LEO score and P-value. This model fitting p-value is calculated using the model chi-square statistic statistic. The chi-square statistic tests the null hypothesis that the model is correct, thus a p-value > 0.01 indicates good fit. The next column indicates the SNP most significantly associated. The last three columns contain chromosome number and base pair location (NCBI build 36) of the SNP and full name of the gene.

A locus in the *BTN3A2* gene passed the LEO threshold of 0.8 for both models SNP → Methylation → Expression (LEO score 6.2 based on causal anchor rs9467632) and SNP → Expression → Methylation (LEO score 1.14 based on causal anchor rs12199613). The two SNPs that were used as causal anchors are in moderate LD (R^2^=0.092, D’=0.68 based on 1000 Genomes Pilot 1 CEU population [25]. The significant results in both directions could indicate a bi-directional causal interaction between expression and methylation. However, while the model SNP → Methylation → Expression fits the data well (model fitting p-value p = 0.10), the model SNP → Expression → Methylation does fits the data poorly (model fitting p-value p=6.4e-5). Thus, while the evidence for the SNP → Methylation → Expression model for *BTN3A2* is strong, the evidence for the SNP → Expression → Methylation model is weak.

### Weighted correlation network analysis of expression and methylation data

We separately constructed co-expression and co-methylation networks from the expression and methylation data, respectively (Additional file 5: Supplementary Methods), using the Weighted Correlation Network Analysis framework WGCNA [26,27]. In expression data (13,843 genes) we identified 23 co-expression modules (labeled 1–23) with sizes ranging from 32 to 1,520 genes. Additional file 6; Table 1 provides a brief overview of the expression modules along with 10 top hub genes (genes with highest module membership) in each module. A total of 7,743 (56% of total) genes were assigned to a module while 6,091 background genes were not assigned to a module. Background genes are labeled 0 and colour-coded in grey. Gene ontology (GO) enrichment analysis revealed significant enrichment of multiple modules in various GO terms (Table 4) which provides evidence that these modules are biologically meaningful. A table listing module membership of all genes in expression modules is provided in Additional file 7.

**Table 4:** Top GO enrichment terms for expression modules

**Module**	**Size**	**Rank**	**p**.**Bonf**	**Fraction**	**Ontology**	**Term name**
1	1520	1	1.20E-14	0.65147	CC	membrane-bounded organelle
1	1520	2	1.90E-14	0.65007	CC	intracellular membrane-bounded organelle
2	703	1	1.30E-05	0.04885	CC	ribosome
2	703	2	1.50E-05	0.07481	BP	translation
3	658	1	0.00018	0.16382	CC	extracellular region
4	647	1	0.011	0.63711	CC	membrane-bounded organelle
6	442	1	7.60E-07	0.27229	BP	response to stress
6	442	2	2.80E-06	0.34217	BP	signal transduction
7	426	1	9.40E-07	0.18734	BP	immune system process
8	407	1	0.0042	0.66755	CC	intracellular membrane-bounded organelle
9	387	1	1.70E-05	0.82961	CC	intracellular part
10	355	1	1.80E-05	0.07855	BP	ncRNA metabolic process
10	355	2	3.60E-05	0.19335	CC	mitochondrion
12	306	1	2.00E-16	0.1134	CC	ribosome
12	306	2	4.00E-16	0.17182	CC	ribonucleoprotein complex
12	306	3	1.20E-14	0.14777	BP	translation
12	306	4	2.30E-14	0.09278	BP	viral transcription
13	260	1	0.00041	0.025	CC	hemoglobin complex
13	260	2	0.011	0.025	BP	heme biosynthetic process
14	237	1	0.014	0.61086	MF	protein binding
15	118	1	0.018	0.25688	BP	intracellular signal transduction
15	118	2	0.032	0.12844	BP	small GTPase mediated signal transduction
16	108	1	1.30E-05	0.18478	BP	translation
16	108	2	0.00017	0.1087	BP	ribosome biogenesis
17	99	1	2.30E-09	0.36957	CC	mitochondrion
18	72	1	3.30E-06	0.1791	BP	platelet activation
18	72	2	1.20E-05	0.22388	BP	blood coagulation
19	60	1	1.40E-17	0.31034	MF	structural constituent of ribosome
19	60	2	2.90E-17	0.32759	CC	ribosome
20	52	1	7.10E-23	0.32653	BP	type I interferon-mediated signaling pathway
20	52	2	7.10E-23	0.32653	BP	cellular response to type I interferon
23	32	1	0.053	0.17241	CC	external side of plasma membrane

For each module we list the top enriched GO terms. Columns list the module label, module size, rank of the enrichment p-value for that particular module, the Bonferroni-corrected enrichment p-value (the correction is performed with respect to the number of GO terms), fraction of the module genes also in the GO term, GO ontology, and GO term name. Multiple expression modules exhibit significant enrichment. Row shading separates modules for easier reading. The enrichment provides evidence that the modules are biologically meaningful.

In methylation data (13,569 genes) we identified 9 modules of sizes ranging from 37 to 1,067 genes. Additional file 6; Table 2 provides a brief overview of the methylation modules along with 10 top hub genes (genes with highest module membership) in each module. For reader-friendliness, methylation module labels were chosen such that modules with significant overlap with expression modules carry the same label (Methods). A total of 4,088 (30% of total) genes were assigned to a module, while 9,481 were not assigned. We observed that strong co-expression relationships tend to be more frequent than strong co-methylation. GO enrichment analysis of methylation modules revealed multiple significantly enriched categories (Table 5). A table listing module membership of all genes is provided in Additional file 8.

**Table 5:** Top GO enrichment terms for methylation modules

**Module**	**Size**	**Rank**	**p.Bonf**	**Fraction**	**Ontology**	**Term name**
32	1067	1	4.20E-09	0.1875	CC	extracellular region
1	1045	1	8.20E-16	0.790123	CC	intracellular
1	1045	3	1.50E-11	0.083333	CC	ribonucleoprotein complex
30	616	1	2.50E-21	0.34687	BP	anatomical structure development
30	616	2	1.60E-18	0.189509	BP	nervous system development
7	594	1	8.30E-10	0.157424	BP	immune system process
7	594	2	9.10E-10	0.177102	MF	receptor activity
3	427	1	3.20E-12	0.259947	CC	extracellular region
12	130	1	0.0032	0.075	BP	DNA recombination
2	105	1	1.70E-10	0.191919	BP	lymphocyte activation
2	105	2	2.30E-10	0.20202	BP	leukocyte activation
2	105	3	3.60E-10	0.323232	BP	immune system process

For each module we list the top enriched GO terms. Columns list the module label, module size, rank of the enrichment p-value for that particular module, the Bonferroni-corrected enrichment p-value (the correction is performed with respect to the number of GO terms), fraction of the module genes also in the GO term, GO ontology, and GO term name. Multiple methylation modules exhibit significant enrichment. Row shading separates modules for easier reading. Again, the enrichment provides evidence that the modules are biologically meaningful.

### Preservation of co-expression modules in methylation data and vice versa

**Figure 1:**
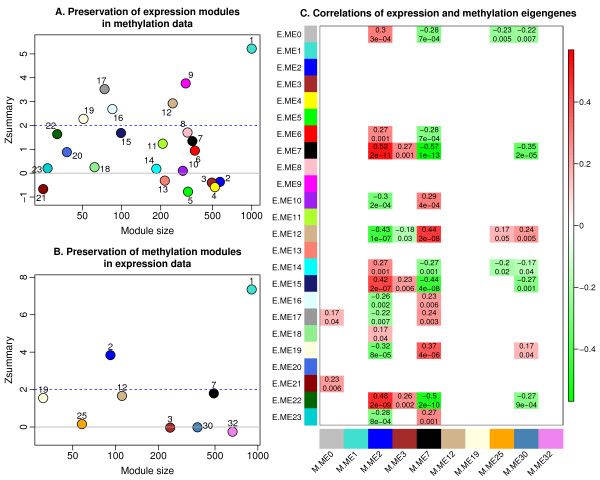
**Preservation and association of co-expression and co-methylation modules. A**. Module preservation statistic*Zsummary * that summarizes evidence of preservation of expression modules in methylation data. Each module is labelled by a numeric label and the corresponding colour. Values of *Zsummary* below 2 indicate no evidence of preservation; values between 2 and 5 indicate weak to moderate evidence for preservation. Only the largest module, labelled 1 (turquoise), exhibits *Zsummary* above 5 that can be considered moderate-strong evidence of preservation. **B**. Analogous plot of the *Zsummary* statistic for preservation of methylation modules in expression data. As in expression data, only the largest module (also labelled 1, turquoise) exhibits moderate-strong evidence of preservation. **C**. Robust correlations and the corresponding p-values of expression (y-axis) and methylation (x-axis) eigengenes. Each row corresponds to an expression eigengene (E.ME) labelled by numeric module label and colour. Each column corresponds to a methylation eigengene (M.ME) labelled by numeric module label and colour. Numbers in the table report the robust correlation and the corresponding p-value of the respective expression and methylation eigengenes. Only correlations whose p-value is below 0.05 are displayed. The table is colour-coded according to correlation such that (strong) green colour corresponds to (strong) negative correlations, and (strong) red colour corresponds to (strong) positive correlations.

A natural question is whether the expression and methylation modules are related. At the most basic level one can ask whether the expression and methylation modules can be matched based on significant overlap of the genes in each module. We found that expression and methylation modules in general exhibit relatively few overlapping genes (Additional file 9) although some of the overlaps are statistically significant. The most significant overlap (p=6e-12) is observed between the largest co-expression module and the largest co-methylation module. While the cross-tabulation based module overlap analysis is a simple and intuitive way of assessing module preservation, it has several limitations. In particular, it cannot be used to make strong statements about the lack of module preservation since alternative module detection methods applied to the test data may lead to different results. A rigorous module preservation analysis is based on the network module preservation statistic Zsummary (Methods) since it is independent of the vagaries of detecting modules in test data [28]. We found that the largest expression module 1 (enriched in intracellular-related terms) exhibits moderate preservation, *Zsummary*≈5. Modules 9 (enriched in intracellular-related terms), 12 (ribosome), 16 (translation), 17 (mitochondrion), and 19 (ribosome) show weak evidence of preservation, while all other expression modules show no evidence of preservation in methylation data (*Zsummary* ≤ 2, Figure 1A). For the methylation modules we found that modules 1 (intracellular) and 2 (lymphocyte activation) show weak to moderate evidence for preservation, while all other modules show no evidence of preservation (*Zsummary* < 2, Figure 1B). It is known that the Zsummary statistic tends to increase with module size, reflecting the intuition that a preservation signal observed among many genes is more significant than a similar preservation signal observed among only a few genes. To measure relative preservation irrespective of module size, the authors of [28] proposed the use of a rank-based statistic *medianRank*. Additional file 10 shows the *medianRank* statistics in this study. The modules with high Zsummary have low (i.e., near top) ranks. Hence, the two preservation statistics offer a largely consistent picture of module preservation, even though they measure very different quantities.

The weak preservation of co-expression modules in methylation data and *vice*-*versa* shows that in general modules (clusters) of expression probes do not correspond to modules of methylation probes. However, we found strong correlations between co-expression modules and co-methylation modules as described in the following.

### Associations of expression and methylation eigengenes

Although the composition of co-expression modules is different from that of co-methylation modules, we observed strong correlations of expression and methylation module eigengenes (Figure 1C). A module eigengene is a mathematically optimal way of summarizing the levels of a module (Methods). For example, eigengenes of methylation modules 2 and 7 (both enriched in immune system/response terms) are strongly correlated with multiple expression eigengenes such as ME 7 (enriched in immune system process), 12 (ribosome), 15 (intracellular signal transduction), 19 (ribosome), and 22 (no significant enrichment). Methylation module eigengenes 3 (extracellular region) and 30 (anatomical structure morphogenesis, nervous system development) also relate to several expression module eigengenes but the associations are weaker. In summary, we observed multiple strong correlations between expression and methylation module eigengenes.

### Module membership of individual genes in expression and methylation modules

Weighted correlation network methods allow one to define a continuous measure of module membership for each variable in each module as the correlation of the variable profile with the module eigengene (Methods). Additional files 7 and 8 report the module membership (based on expression and methylation profiles) of all genes in all modules. Since the expression and methylation data were measured for the same set of samples, we are able to also provide the module membership of expression profiles in methylation modules and *vice*-*versa*. These Supplementary Files serve as a resource for relating expression and methylation probes to the modules.

## Discussion

We investigated the relationship between genetic variation, DNA methylation and gene expression in a sample of 148 healthy subjects using array-based data derived from whole blood. We found both negative (levels in opposite direction) and positive (levels in same direction) associations between *cis*-acting DNA methylation probes and corresponding gene expression levels, confirming previous reports that DNA methylation and gene expression located within a *cis*-region can be both positively and negatively associated, but are predominantly negative [5-7].

In this study we applied FDR correction for multiple testing for *cis* associations between methylation and expression, but imposed a more stringent genome-wide significance threshold for *trans* effects since there is a considerable debate in the literature whether such relationships are reproducible [29,30]. This resulted in a limited number of *trans* associations that do survive this threshold but with relatively strong effect sizes. It is of note that such *trans* associations are enriched for positive correlations, whereas traditionally it is expected that methylation and expression are inversely correlated. We hypothesised that these involve genes involved in general methylation pathways, such as genes that induce the attachment of a methyl group. However, a gene ontology analysis did not show any overrepresented pathways (data not shown).

Furthermore, we observed that methylation probes with *cis*-acting effects on gene expression levels are less likely to be located in CpG islands and more likely to be present outside CGIs and shores insofar they were not regulated by genetic variation. Tissue- and cell type-specific methylation occurs much more often in gene bodies (outside island and shores) than in CpG island promoters [31], indicating that methylation at CpG sites in CpG islands is much more static, which could explain the underrepresentation of CpG sites associated with expression (and SNPs) in CpG islands. Only for those CpGs that were associated with SNPs, we did concur with previous studies showing more frequent associations with expression in island shores [2,3]. CpG sites located in shores tend to be more variable among individuals and this might lead to an increased number of association findings. In addition, *trans* associations are less likely to be located in island shores and more likely to be positioned outside CGIs and shores. Also, trans associations are more likely to be positive (67%).

Identification of genetic variants (SNPs) influencing the methylation and expression levels showed that in more than 12% of methylation-expression *cis*-pairs, the methylation and/or the expression level was associated with a SNP in *cis*, suggesting genetic control of these levels.

Further analysis of genetic regulators (SNPs) of methylation and expression levels investigating the causality revealed three-way causal relationships. Previous studies have attempted to identify three-way associations in various tissues, with mixed results [6,7]. We used local structural equation models to calculate local edge orienting (LEO) scores based on using a *cis*-acting SNP as causal anchor [19,32]. We find that the traditional model of genetic variants regulating methylation, which in turn regulates gene expression to be most common in most of the three-way associations that showed significant evidence for causality (as was hypothesized in literature [5]). The set of genes for which the S→M→E model fits best does not exhibit significant enrichment for specific functions or pathways. Since the S→M→E model is expected to be ubiquitous, the lack of enrichment is not surprising. However, one of the genes that fit this model, *PNMA3*, is located on the X chromosome. Since inactivation in females may be a confounding factor when analyzing X chromosomes, we repeated the association analysis for all significant X chromosomes in males only. We observed no significant differences when using males-only, which confirms that the *PNMA3* finding is likely to be true. Strikingly, the reverse model, in which a genetic variant primarily regulates gene expression, which in turn regulates DNA methylation, was the best causal model for a number of genes (including *C21ORF56*, *HRASLS3*, *TACSTD2*, *WBSCR27*, *SRXN1*, *GSTM3*, *BTN3A2*), although the model p-values of these LEO scores were small, indicating poor fit. For example, one of these genes, *C21ORF56*, was highlighted in a previous genome-wide study where a three-way association for this gene was identified. Additional experiments indicated that genetic variation in this gene affects chromatin structure in this region [5]. The gene itself may be involved in inter-individual differences in response to DNA damaging agents [33]. These mechanisms and our data suggest that loci whereby genetic variation influences expression and in turn methylation may exist and warrants further study. The methylation and expression probes that showed a causal direction in the LEO analysis were all present within the same gene. However, we observed that of all the 798 significant *cis* associations, only 155 (19%) involved probes that represent the same gene. This may suggest that the strongest (detectable) causal correlations between DNA methylation and gene expression are likely to be local events.

The systems level analysis afforded by WGCNA reveals that both transcriptome and methylome can usefully be organized into modules. Many co-methylation and co-expression modules are highly significantly enriched with gene ontology categories, which provides indirect evidence that these modules are biologically meaningful. Our module preservation analysis between expression and methylation data reveals that most co-expression modules are comprised of genes that do not form a module in the methylation data and vice versa. Only the largest co-expression module shows moderate to strong preservation and overlap with the largest co-methylation module. In other words, co-expression modules and co-methylation modules are largely composed of different genes. On the other hand, several pairs of expression and methylation eigengenes show highly significant positive and negative correlations. This suggests the existence of factors that affect expression and methylation of different sets of genes, i.e., *trans* effects at the module level.

A limiting factor of our study may be the fact that the Illumina 27k array covers only a selection of CpG sites and is enriched for promoter regions and CpG islands near genes. Another increasingly important issue is the potential difference between hydroxymethylation and DNA methylation that cannot be distinguished with current methylation arrays [34,35]. To date, the role of 5-hydroxymethylation is not fully understood but it is likely that 5-hydroxymethylation plays a role in demethylation [34-38]. Although there is no reason to assume a systematic influence of 5-hydroxymethylation on our results, we cannot rule this out and further refinement of methylation levels is warranted. A third possible limitation is the use of whole blood comprised of different cell types for our analysis. Yet, although whole blood does not provide the optimal resolution, these cell types can be used to study general genetic mechanisms. Given the sample size we suspect that effects of blood cell composition are limited and do not play a major role in the outcome. We measured gene expression and DNA methylation from the same blood sample so that the composition of different cell types should not substantially affect the overall outcome and conclusions. Moreover, studies have shown that a majority of the strongest eQTLs overlaps between different tissues and cell types [6,39].

## Conclusions

Overall, this study contributes to our understanding about the relationship between genetic markers, methylation and expression levels in whole blood of healthy subjects. We observed *cis*-associations between methylation and expression levels to be both positive and negative, and most likely to be located outside CGIs and shores. Overrepresentation in shores, as previously found, was only present when selecting methylation/expression combinations regulated by genetic variation in *cis*. Methylation/expression combinations in *trans* are enriched for positive correlations and also located mostly outside CGI’s and shores. Results from causality analyses indicate that the conventional model of genetic variants regulating methylation, which in turn regulates gene expression, is most common. This is widely supported in literature [32]. In addition, this indicates that the causal direction analysis is a useful tool for investigating relationships between genotype, methylation and expression. Finally, we showed that methylome and transcriptome are organized into modules. Although the co-expression en co-methylation modules are generally not preserved in one another, we do find highly significant correlations between the modules. These findings suggest that there may be other (*trans*) factors affecting both methylation and expression, although in different modules. This study encompasses lookup tables for associations between methylation, gene expression, and genotype, as well as methylome and transcriptome modules, for further research.

## Methods

### Ethics statement

All participants gave written informed consent. This study was approved by Medical Research Ethics Committee (MREC) of the University Medical Center Utrecht, The Netherlands.

### Pre-processing of genotype, methylation and expression data

Genotype, methylation and expression data were collected for different numbers of samples. For the 148 healthy subjects eventually analyzed in this paper, data was available for all three layers of genetic information after quality control, as described below. Our final data set consisted of 72 males and 76 females with a mean age of 52 (range: 19–88); all subjects were of Dutch ancestry with at least three of the four grandparents born in The Netherlands.

#### Genotype SNP data

Genotype data for subjects was generated on two different array platforms, 105 individuals on Illumina CytoSNP (299,173 SNPs) and 96 on Illumina 300k chips (300,299 SNPs). For each SNP platform, quality control procedures were initially performed separately using PLINK [24]. Subjects were excluded based on > 5% missing genotypes and gender errors (Additional file 11). We used linkage disequilibrium (LD) based SNP pruning to select the most informative SNPs (R^2^<0.2), only for subsequent quality control steps. This resulted in ~60k SNPs for both sets to assess heterozygosity (F<3 Standard Deviation (SD)), homozygosity (F>3SD) and relatedness by pairwise identity by descent (IBD) values (pihat > 0.1). Datasets were merged with Hapmap Phase 3 individuals to check ethnicity (Additional file 12) (ethnic outliers detected by visual inspection). After these QC procedures on subjects (excluding in total 8 individuals) quality control on SNPs was performed as follows. All SNPs were filtered on missingness (> 2%) and Hardy Weinberg (*p*>1e-6) before merging the two datasets. 84,367 SNPs were shared between the two datasets. No related samples were detected in the merged datasets (according to criteria described above). We imputed the merged dataset with Hapmap2, release 24 using Beagle [40]. SNPs with an imputation score > 0.8 and present originally in one or both datasets were extracted and 417,708 SNPs remained for all further analyses.

#### DNA methylation data

Methylation data was obtained using Illumina HumanMethylation 27 beadchips for two batches of 105 and 96 healthy subjects. The assay detects methylation status at CpG sites after bisulfate conversion, by means of probes designed for either methylated or unmethylated sequence. Methylation probes were classified into 3 different categories depending on the location of the probe with respect to a CpG island. Based on the UCSC Table browser ( http://genome.ucsc.edu/; [41]), NCBIbuild36, categories were defined as CpG island, CpG island shore (sequences up to 2kb from an island), or outside CpG islands/shores. Ethnical outliers and samples with gender errors in genotype data were removed from the methylation data. Gender was checked by hierarchical clustering of X-chromosomal probes, excluding four individuals. Another three individuals were removed based on detection *p*-values (> 0.01 for > 1% of probes) and 3,027 of 27,578 probes were excluded based on detection values (*p*>0.01 for > 1% of the samples). Both channels of the methylation array were quantile normalized independently. Beta values of a probe were calculated by dividing the methylated signal by the sum of the methylated and unmethylated signal. Next, five potential array outliers were removed in an unbiased fashion. Specifically, we used the SampleNetwork R function package [42] to calculate the Interarray based sample connectivity score Z.k. We removed samples with a Z.k value less than −3 since their connectivity is 3 standard deviations below the mean value. Batch effects of dataset, plate, array and position were removed using ComBat [43]. After these procedures, 24,561 probes remained and were mapped to the human genome using the UCSC Human BLAT Search function. In total, 25 probes did not map to the human genome, whereas 338 probes did not map uniquely (mapped more than once), and both these probes have been removed. Moreover, 904 probes that contained a SNP, based on Hapmap release 27, with a minor allele frequency (MAF) > 1% have been removed as well, leaving a total of 23,294 probes for analyses.

#### Gene expression data

Gene expression data was generated in two batches, one on Illumina H8 beadchip (26 healthy subjects) and one on Illumina H12 beadchip (147 healthy subjects). BeadStudioÂ© software version 3.2.3 was used to generate background-corrected gene expression data. Data was normalized, transformed and filtered separately before merging and batch effect removal. Specifically, the datasets were separately quantile normalized and log2 transformed using the Lumi package for R [44]. Probes were filtered based on detection values generated by BeadStudioÂ©. The detection *p*-value threshold was set at 0.01. This resulted in 17,433 expression probes overlapping between both batches. Batch effects resulting from the use of different arrays at different time points were removed using ComBat [43]. An unbiased analysis based on interarray correlations identified 16 samples from batch 2 as potential outliers, which were subsequently removed from the analysis. Of 17,433 probes, 15,983 mapped to a single genomic location, based on a previous study [45]. In addition, 465 probes contained a SNP, based on Hapmap release 27, with a MAF > 1% and have been removed, leaving 15,983 probes for analyses.

DNA methylation and gene expression data have been processed using the same blood sample, excluding possible batch effects, such as the effect of different time points.

### Identifying *cis* and *trans* effects between DNA methylation and gene expression

We called a methylation probe *cis* acting with respect to a given gene expression probe if there was a significant association (as defined below) within a 500kb interval between the probes. A methylation probe was called *trans* acting if it was significantly associated with the expression probe (as defined below) outside the 500kb interval.

To determine whether a significant association exists between expression and methylation levels we used a multivariate linear regression model for regressing the gene expression level (dependent variable) on the methylation level (independent variable) with age and gender as covariates. We took methylation levels as independent variable since we are interested in the epigenetic control of gene expression levels. Associations can be positive (DNA methylation levels and gene expression levels both increase or decrease) or negative (increased methylation level corresponds with a decrease in gene expression level and vice versa). The Wald test *p*-value for the association between methylation and expression was used as significance level. Correction of the significance level for multiple testing was performed separately for identifying *cis* acting methylation probes (FDR correction) and *trans* acting methylation probes (Bonferroni correction).

### Identification of *cis*-and *trans*-acting SNPs

Expression levels and methylation levels that were significantly associated with each other were tested for association with SNPs to identify *cis*-and *trans*-acting genetic variations. For this analysis, the real and imputed (imputation score > 0.8) genotypes were used, and a MAF threshold of 5% for these SNPs was set.

Analogous to our previous definition, a SNP significantly associated with a given gene expression or DNA methylation probe was called *cis*-acting with respect to the probe if the SNP and the probe were within 500kb of each other, and *trans*-acting if they were more than 500kb apart.

To determine whether a significant relationship exists between a SNP and a methylation or expression level we again used a multivariate linear regression model for regressing the methylation or expression level (dependent variable) on the SNP (independent variable) with age and gender as covariates. The regressions were performed using the PLINK software [24]. Correction for multiple testing was performed separately for *cis*-acting SNPs (0.05 divided by the number of probes) and *trans*-acting SNPs (0.05 divided by the number of possible combinations (*p*<0.05/(#probes*417,708).

### Evaluating causal relationships using local edge orienting scores of observed *cis* effects

To evaluate the fit of different causal models involving 3 variables (i.e., a *cis*-acting SNP, a *cis*-acting methylation probe, and a corresponding expression probe), we calculated the single marker local edge orienting score (LEO.NB.SingleMarker) as described elsewhere [19,32]. In short, a SNP can be used as causal anchor for evaluating the causal relationships between methylation and expression levels if the SNP is associated with at least one of them. We use the SNP as causal anchor for calculating the LEO score since genotypes are fixed at each locus as opposed to variable methylation and expression levels [19]. In this case, one can evaluate the fit of the following five models describing the causal relationships between a SNP (denoted S), a methylation probe (M) and an expression probe (E): model 1: S→M→E; model 2: S→E→M; model 3: M←S→E; model 4: S→E←M; model 5: S→M←E. For each causal model a chi-square test based model fitting p-value was calculated with the structural equation modelling (SEM) R package [46]. The relative fit of causal model 1 (SNP→Methylation→Expression) was assessed using the single anchor local edge orienting score (LEO.SingleMarker), which is the logarithm (base 10) of the ratio of the model fitting *p*-value divided by that of the next best fitting alternative model [19]. Thus a positive LEO.SingleMarker score indicates that the causal model S→M→E fits the data better than all other competing models. As significance threshold we used the LEO threshold of 0.8, as recommended in [19] based on extensive simulations as well as empirical studies. We decided to focus on local *cis* effects since there is considerable debate in the literature whether *trans* relationships are reproducible [29,30]. Since we were interested in causal direction for predetermined three-way associations, we only selected SNPs associated with both the methylation and expression levels in *cis*. To protect the causal analysis from biases due to age and gender, we utilized residuals of methylation and expression levels corrected for age and gender in the causal analysis using a linear regression by Limma in R [47].

### Weighted correlation network analysis of gene expression and methylation data

A detailed description of our correlation module based analyses can be found in Additional file 5. Here we provide a terse summary. Weighted correlation network analysis implemented in the WGCNA R package [26,27] was first applied to the expression data to identify co-expression modules. Co-expresssion modules correspond to clusters of interconnected genes defined as branches of a hierarchical cluster tree. Since modules are defined without respect to gene ontology information they are initially labelled by arbitrary integers and coded by colours. Next WGCNA was applied to the methylation data to find co-methylation modules. For easier interpretation of the relationships between expression and methylation modules, we use the same module labels for modules that show significant overlap. The matching of module labels was performed using the function matchLabels from the WGCNA R package; it is based on significance of module overlaps quantified using Fisher’s exact test. Weighted networks have the advantage of preserving the continuous nature of co-expression and co-methylation information, which is particularly useful when studying module preservation. To assess the preservation of expression and methylation modules in the corresponding complementary data set, we use the network module preservation statistics described in [28] and implemented in the function modulePreservation in the WGCNA R package. Network module preservation statistics assess whether the density and connectivity patterns of modules defined in a reference data set are preserved in a test data set. Network preservation statistics do not require that modules be identified in the test data set and hence independent of the ambiguities associated with module identification in the test data set. The permutation test of the modulePreservation function leads to a composite module preservation statistic referred to as Zsummary. The Zsummary statistic of a given module summarizes the evidence that the network connections of the module are more significantly preserved than those of random set of genes of equal size. We adopted the following recommended significance thresholds for Zsummary [26-28]: Zsummary<2 implies no evidence that the module is preserved, 2<Zsummary<10 implies weak to moderate evidence, and Zsummary>10 implies strong evidence for module preservation. Thus, we report Z summary for each expression and methylation module in the methylation and expression test data sets, respectively.

Since modules group together highly correlated variables, it is advantageous to summarize the variable profiles using a single representative. We use the module eigengene E, defined as the first principal component of the standardized matrix containing the variables in the module. The module eigengene can be intuitively understood as a weighted average of the variable profiles in the module.

## Abbreviations

CpG: Cytosine-Phosphate-Guanine; CGI: CpG Island; QTL: Quantitative Trait Loci; LEO: Local Edge Orienting; FDR: False Discovery Rate; SNP: Single Nucleotide Polymorphism; LD: Linkage Disequilibrium; MHC: Major Histocompatibility Complex; WGCNA: Weighted Gene Co-expression Network Analysis; GO: Gene Ontology; MREC: Medical Research Ethics Committee; SD: Standard Deviation; IBD: Identity By Descent; SEM: Structural Equation Modeling.

## Competing interests

The authors have declared that no competing interests exist.

## Authors’ contributions

KRE, SJ and PL wrote the paper. KRE, SJ, TL and PL analyzed the data. MPMB, SH and RAO designed the study. TL, FC and PL wrote scripts for the analyses. RAO, MPMB, SH, JHV, LHB and RSK provided data, materials, and analysis tools. EJ and ES processed the data. CGFK advised on statistics. The manuscript has been seen and approved by all listed authors.

## Supplementary Material

Additional file 1 **Table S1.** Comprises two tables that list all significant methylation and expression associations in *cis* (S1a), and *trans* (S1b). Click here for file

Additional file 2 **Figure S1.** Are two figures that show the coefficient and explained variance of associations between methylation and expression. Click here for file

Additional file 3 **Table S2.** Contains tables with all significant *cis* mQTLs (S2a) and eQTLs (S2b). Click here for file

Additional file 4 **Table S3.** Is a table with all LEO results. Combinations that have a LEO score above 0.8 for the model S>M>E are shown in light yellow of which LEO scores above 3 are shown in dark yellow. For the reverse model (S>E>M) combinations with a LEO score above 0.8 are shown in orange. Significant p-values (above 0.01) are coloured in green. Click here for file

Additional file 5 Contains supplementary methods, namely, a more detailed description of Weighted Correlation Network Analysis (WGCNA). Click here for file

Additional file 6 **Is an overview of the modules identified in the expression (Table** 1**) and methylation (Table** 2**) data.** Click here for file

Additional file 7 **Includes a table of continuous module membership *kME***_***i ***_**of all expression profiles in all expression modules.** Each row in the table corresponds to one gene expression profile. Columns give the gene Entrez idenitifier, module label, and *kME* and the corresponding (uncorrected) p-values for each module. Expression modules are labelled by E.0, E.1, etc. Click here for file

Additional file 8 **Includes a table of continuous module membership *kME***_***i ***_**of all methylation profiles in all methylation modules.** Each row in the table corresponds to one methylation profile. Columns give the gene Entrez idenitifier, module label, and *kME* and the corresponding (uncorrected) p-values for each module. Methylation modules are analogously labeled by M.0, M.1, etc. Click here for file

Additional file 9 **Shows the overlap of expression and methylation modules.** Each row corresponds to an expression module (labelled by the numeric labels, colours and total number of genes in the module, on the left), and each column corresponds to a methylation module (labelled the numeric labels, colours, and total number of genes in the module, at the bottom). Numbers in the table indicate number of genes in the overlap, and the Fisher exact test p-value for the overlap. Only overlaps whose p-value is below 0.05 are shown. The table is coloured such that significant overlaps are coloured in strong red colour. Most overlaps are quite small but some are nevertheless statistically highly significant. Click here for file

Additional file 10 **Shows the *medianRank *** **statistics for the Module preservation with in (A) preservation of expression modules in methylation data, and in (B) preservation of methylation modules in expression data.** Click here for file

Additional file 11 Is a table with the number of excluded samples per step. Click here for file

Additional file 12 Is a clusterplot of all samples together with Hapmap phase 3 populations. Click here for file
