# Supplementary material for: Genetic analysis of DNA methylation and gene expression levels in whole blood of healthy human subjects
Source: BMC Genomics. 2012 Nov 17;13:636. doi: 10.1186/1471-2164-13-636 (PMC3583143; doi:10.1186/1471-2164-13-636)
Supplement: Additional file 1 — Table S1. Comprises two tables that list all significant methylation and expression associations in cis (S1a), and trans (S1b). [file 1471-2164-13-636-S1.pdf]

Methprobe	Exprprobe	P_value	Coefficient	T_statistic	F_statistic	R_squared
cg10904672	2370010	4.15E-13	-36.125316	-7.9759706	23.8371721	0.33028967
cg22917487	2370010	9.04E-13	-26.936309	-7.8373546	23.0788591	0.32317813
cg04749372	670369	9.89E-11	-26.338611	-6.9781427	21.0793465	0.3036815
cg04311964	670369	3.67E-12	-25.966575	-7.5850775	24.2468788	0.3340701
cg04749372	3170128	3.04E-11	-25.899796	-7.1978027	20.4081381	0.29688247
cg01500140	2370010	4.00E-15	-25.510946	-8.7893933	28.5549897	0.37138266
cg18493182	670369	5.64E-12	-25.389759	-7.5072333	23.8258579	0.33018466
cg19372178	3170128	1.05E-12	-24.962058	-7.8112545	23.6240872	0.32830648
cg04311964	3170128	6.98E-12	-24.675949	-7.4682515	21.7935333	0.31077295
cg18493182	3170128	4.30E-12	-24.513662	-7.5565281	22.2567976	0.31529617
cg19372178	670369	7.49E-11	-24.065741	-7.0302643	21.341001	0.30629645
cg03842617	670369	2.57E-11	-23.717346	-7.2287937	22.3554664	0.31625189
cg23499956	670369	4.35E-14	-23.607448	-8.3731603	28.753783	0.37300374
cg21518947	670369	1.19E-12	-23.455154	-7.7881418	25.365595	0.34417861
cg26099316	3170128	4.10E-11	-22.984617	-7.1424926	20.1311012	0.29403736
cg16155702	670369	2.69E-13	-22.953919	-8.0527154	26.8675149	0.35727675
cg22680812	670369	5.24E-11	-22.910684	-7.0967946	21.677817	0.30963378
cg21518947	3170128	4.88E-13	-22.861157	-7.9471954	24.3723234	0.33521908
cg23499956	3170128	5.04E-14	-22.638873	-8.3474615	26.6504052	0.3554158
cg03842617	3170128	4.60E-11	-22.540437	-7.1211118	20.0245815	0.29293728
cg24346637	3170128	5.62E-11	-22.428987	-7.0838152	19.8395342	0.29101804
cg07123548	3170128	1.02E-10	-22.293884	-6.9729034	19.2949851	0.28530925
cg05666713	3170128	6.43E-11	-22.230325	-7.0588191	19.7160595	0.2897316
cg00935364	670369	3.77E-13	-22.164253	-7.993256	26.5256078	0.35434121
cg22680812	3170128	4.31E-11	-22.100436	-7.1334026	20.0857757	0.29356968
cg16155702	3170128	3.72E-13	-21.94892	-7.9953481	24.6404582	0.33766175
cg17983307	3170128	8.18E-11	-21.479429	-7.0137217	19.4943931	0.28741039
cg22917487	10504	4.86E-14	-21.396716	-8.3538071	28.0417847	0.36715864
cg22917487	4890241	1.55E-14	-21.345347	-8.5523651	34.5967991	0.41718008
cg22917487	5490768	1.38E-14	-21.182771	-8.5741483	33.6517082	0.41046156
cg24457403	670369	7.76E-12	-21.161181	-7.4489237	23.5133353	0.32727106
cg01500140	10504	1.00E-17	-21.010904	-9.9968473	38.7630357	0.44505915
cg00935364	3170128	9.53E-13	-20.990117	-7.827823	23.7145915	0.32915024
cg04048249	670369	2.05E-11	-20.612252	-7.2704015	22.5716584	0.31833666
cg24457403	3170128	4.34E-12	-20.542611	-7.554643	22.2468479	0.31519964
cg27022827	3170128	7.18E-12	-20.345737	-7.4631525	21.7669406	0.31051149
cg10624445	670369	3.51E-12	-20.239398	-7.5929999	24.2899704	0.33446524
cg09354331	670369	7.74E-12	-20.16313	-7.4495097	23.5164635	0.32730035
cg22951794	670369	1.04E-10	-19.956321	-6.9681332	21.0293208	0.3031793
cg10624445	3170128	9.91E-13	-19.861449	-7.8207789	23.6760901	0.32879155
cg04048249	3170128	2.43E-11	-19.746101	-7.2396484	20.6191547	0.29903424
cg14496375	1440750	2.29E-13	-19.426567	-8.0811296	26.1729519	0.35128515
cg22917487	1440750	1.00E-17	-19.335359	-9.6707844	36.1704662	0.42803361

cg01500140	4890241	1.78E-15	-19.238297	-8.9277513	37.0913585	0.4341995
cg14269477	3170128	3.37E-11	-19.1875	-7.1787774	20.3126031	0.29590394
cg08766149	10504	1.28E-12	-19.166878	-7.7755063	24.7250142	0.33842833
cg11068096	3170128	3.64E-11	-18.939706	-7.1648565	20.2428601	0.29518787
cg01500140	5490768	3.55E-15	-18.893307	-8.8079397	35.1763989	0.42122514
cg09354331	3170128	4.99E-11	-18.717707	-7.1061488	19.9502259	0.29216734
cg00392257	670369	6.57E-11	-18.529	-7.0547478	21.4645822	0.30752469
cg06037693	670369	3.14E-12	-18.262582	-7.6136074	24.4022702	0.33549279
cg08929103	670369	8.42E-12	-18.228526	-7.4340512	23.4340127	0.32652751
cg08766149	5490768	5.25E-12	-18.209076	-7.5202281	27.2876631	0.36084771
cg20993403	670369	3.54E-11	-18.196908	-7.1699274	22.0517192	0.31330117
cg20993403	3170128	5.53E-12	-18.088819	-7.5107522	22.0158919	0.31295145
cg06037693	3170128	5.32E-13	-18.064501	-7.9319186	24.2875942	0.33444346
cg00392257	3170128	3.50E-11	-18.024651	-7.1720317	20.2787907	0.29555696
cg08929103	3170128	2.12E-12	-17.938027	-7.6843457	22.9372158	0.32183302
cg08766149	4890241	2.23E-11	-17.907848	-7.2557139	26.8043594	0.35673653
cg12240237	670369	8.49E-13	-17.873048	-7.8485003	25.7038181	0.3471746
cg22917487	2450427	3.33E-15	-17.800358	-8.812938	31.6509759	0.39571481
cg01500140	5360064	4.39E-11	-17.785474	-7.1297654	21.9154255	0.31196886
cg01500140	1440750	1.00E-17	-17.76637	-10.482685	41.9673783	0.46475136
cg12240237	3170128	2.49E-13	-17.506392	-8.0667517	25.041046	0.34127779
cg01500140	670369	6.89E-13	-16.901516	-7.8857768	25.9140039	0.34902267
cg01500140	3170128	1.46E-13	-16.632103	-8.160897	25.5746628	0.34603377
cg22181664	670369	5.58E-11	-16.590287	-7.0851088	21.6184268	0.30904765
cg16052901	3170128	5.63E-11	-16.367106	-7.0835785	19.8383626	0.29100585
cg22181664	3170128	1.51E-11	-16.342065	-7.3272485	21.0648591	0.30353613
cg08766149	1440750	1.33E-13	-16.32841	-8.1770592	26.7254984	0.35606068
cg01500140	4670193	1.00E-17	-16.281001	-9.5014751	41.8307927	0.46394053
cg24506604	670369	1.08E-11	-16.265052	-7.3879565	23.1891741	0.32422205
cg23001650	5420564	9.99E-11	-16.22747	-6.9762933	19.9296428	0.29195391
cg24506604	3170128	1.33E-12	-16.202638	-7.7684303	23.3910508	0.32612411
cg01500140	5670605	1.20E-14	-16.03972	-8.5979931	24.6706506	0.33793568
cg22917487	5670605	6.62E-11	-15.920482	-7.0532294	16.6083207	0.25574219
cg04126866	5390246	5.28E-12	-15.914283	-7.5189814	32.2414749	0.40014337
cg21902327	3170128	9.37E-11	-15.753027	-6.988341	19.3702651	0.28610392
cg05615487	3170128	1.82E-12	-15.715332	-7.7121026	23.0864835	0.32325039
cg22917487	4670193	9.79E-12	-15.714267	-7.4065541	28.2566299	0.36893385
cg04856043	3310091	5.04E-12	-15.70319	-7.5274713	19.9693443	0.29236547
cg21207436	670369	1.06E-11	-15.670715	-7.3916257	23.2086077	0.32440562
cg08766149	5670605	7.18E-12	-15.619863	-7.4632324	18.5930355	0.2778133
cg08766149	2450427	5.58E-13	-15.594372	-7.9234694	26.3036078	0.35242076
cg01525376	1030296	7.88E-14	-15.541955	-8.2692233	38.4546218	0.44308708
cg05615487	670369	4.86E-11	-15.418512	-7.1108419	21.7493382	0.31033832
cg10904672	7570408	1.42E-11	-15.395914	-7.3379742	18.6139587	0.27803901

cg21207436	3170128	6.10E-12	-15.20785	-7.4928044	21.921837	0.31203165
cg08766149	4670193	5.45E-12	-14.945392	-7.5133768	28.8674069	0.37392656
cg22917487	6580408	6.40E-11	-14.799874	-7.0596085	19.5929338	0.28844414
cg22917487	3190379	4.20E-11	-14.51302	-7.1380813	18.4465092	0.2762287
cg01500140	6580408	1.56E-13	-14.371475	-8.1489587	25.3688348	0.34420744
cg21518947	6020523	5.39E-12	-14.35635	-7.5155132	19.7852	0.29045253
cg01500140	3390594	2.04E-13	-14.294718	-8.1018761	22.8818519	0.3213058
cg17141902	4210619	4.48E-12	-13.700314	-7.5489839	22.9226256	0.32169416
cg03245641	3170128	2.83E-11	-13.667445	-7.2111024	20.4750725	0.29756644
cg15958424	6380672	3.96E-11	-13.664271	-7.1491096	18.094168	0.27238971
cg20987610	1440750	1.14E-11	-13.56601	-7.3781637	22.3228884	0.31593663
cg24457403	4210619	2.86E-13	-13.390292	-8.0422196	25.6355119	0.34657175
cg22375610	4760338	2.78E-11	-13.29026	-7.2142732	17.4558418	0.26533
cg21057494	6020523	2.07E-11	-13.258369	-7.2693853	18.5549135	0.2774017
cg24474182	5260424	1.01E-10	-13.230524	-6.9743604	16.6344086	0.25604105
cg01500140	3190379	5.79E-12	-13.188173	-7.5023221	20.263852	0.29540355
cg12703269	4210619	8.77E-11	-13.064712	-7.0005928	20.107886	0.29379789
cg01643624	6020523	1.05E-10	-13.040695	-6.9664679	17.0969903	0.26130071
cg27299588	4210619	4.37E-13	-12.954474	-7.966831	25.2097481	0.34278885
cg23181133	4220187	9.68E-11	-12.946437	-6.9821632	17.5184497	0.26602848
cg00739120	5690148	6.31E-11	-12.888297	-7.0622164	16.6317608	0.25601072
cg20987610	2450427	4.86E-11	-12.887332	-7.110827	21.9149689	0.31196439
cg20790056	6590228	6.17E-12	-12.844444	-7.4907857	19.804443	0.29065291
cg07285167	6380672	4.89E-11	-12.839806	-7.1098236	17.9044214	0.27030538
cg00935364	6020523	4.10E-11	-12.83621	-7.1427413	17.9378364	0.2706733
cg10624445	4210619	9.88E-14	-12.833128	-8.2296578	26.7114646	0.35594026
cg22630748	4180544	4.85E-12	-12.750835	-7.5343991	20.6810199	0.29966259
cg01782486	5390246	8.37E-12	-12.647159	-7.4351029	31.7399888	0.39638656
cg22917487	6020523	3.97E-14	-12.63496	-8.3893904	24.4842326	0.33624074
cg08399444	6380672	1.49E-11	-12.626894	-7.3298119	18.9804021	0.28196923
cg15361231	6380672	9.76E-11	-12.525511	-6.9805781	17.2875604	0.26344598
cg01525376	3460520	1.51E-11	-12.454255	-7.3273855	23.3952991	0.32616402
cg21842274	5420564	1.35E-11	-12.44531	-7.3470146	21.8012585	0.31084887
cg01500140	6020523	1.00E-17	-12.444536	-10.09697	35.156563	0.42108764
cg01500140	2470161	1.05E-13	-12.394184	-8.2194466	25.3387849	0.34393995
cg02540157	4760338	5.61E-11	-12.388175	-7.0842936	16.8353224	0.25833466
cg16967583	5260424	2.50E-11	-12.371654	-7.2342267	17.8732042	0.26996132
cg14859417	5420564	7.88E-11	-12.366165	-7.0207557	20.1490017	0.29422189
cg19464252	670369	1.79E-12	-12.34595	-7.7144209	24.9560364	0.34051373
cg21842274	6840468	2.60E-11	-12.294569	-7.227066	19.7543646	0.29013119
cg27022827	4210619	4.05E-11	-12.292009	-7.1447218	20.8271054	0.30114189
cg22045288	4180544	2.18E-11	-12.288805	-7.2593759	19.2893471	0.28524967
cg25066857	6020523	1.20E-11	-12.282248	-7.3689075	19.0474488	0.2826837
cg09169633	4180544	4.20E-11	-12.271253	-7.13792	18.6912266	0.27887131

cg02600394	5390246	1.73E-11	-12.232411	-7.301914	30.9552478	0.39041243
cg22951794	4210619	3.59E-11	-12.214862	-7.1673835	20.9415232	0.30229616
cg13650156	6380672	7.77E-11	-12.214667	-7.0233719	17.4905528	0.26571741
cg10624445	6020523	3.24E-11	-12.208119	-7.1860947	18.1478563	0.27297731
cg02540157	4210619	1.28E-11	-12.200643	-7.3581421	21.9190159	0.31200403
cg01525376	3290731	3.39E-11	-12.151163	-7.1780664	27.4625151	0.36232216
cg01500140	1030167	5.68E-13	-12.146197	-7.9203467	26.3821933	0.35310189
cg01525376	4210619	2.68E-11	-12.14296	-7.2216244	21.2168545	0.30505819
cg09106999	540368	1.80E-12	-12.091578	-7.7133605	21.5080777	0.30795594
cg26215727	3370327	1.25E-11	-12.081892	-7.361084	18.0686541	0.27211014
cg05615150	6590228	2.44E-15	-12.06008	-8.8743216	27.4755767	0.36243202
cg17753124	540368	5.93E-11	-12.058947	-7.0739524	18.278872	0.27440725
cg17753124	1030270	1.11E-10	-12.047249	-6.9566661	17.5329891	0.2661905
cg22016649	4180544	1.40E-11	-12.01568	-7.3405779	19.6948624	0.28951029
cg22016649	3310091	1.18E-12	-11.98047	-7.7893208	21.3277563	0.30616455
cg09358725	5420564	4.13E-11	-11.966771	-7.1414314	20.7513889	0.30037595
cg01500140	6760037	9.84E-11	-11.948187	-6.9789635	17.3171597	0.26377806
cg15361231	5260424	1.50E-11	-11.93758	-7.3285464	18.3340394	0.27500768
cg04126866	5390730	1.76E-11	-11.92001	-7.2988032	27.8394595	0.36547773
cg22045288	6480692	8.58E-11	-11.916224	-7.0047025	18.0121794	0.27149055
cg09358725	4180544	1.94E-12	-11.913657	-7.7000473	21.544202	0.30831371
cg01500140	3850246	1.12E-10	-11.902242	-6.9547624	19.2845046	0.28519848
cg09106999	1030270	1.52E-11	-11.785723	-7.3254106	19.3266262	0.28564348
cg07073964	540368	7.50E-11	-11.697474	-7.0300247	18.0672752	0.27209502
cg22045288	3310091	2.72E-11	-11.694228	-7.2189038	18.4281855	0.27603005
cg25372195	6020523	5.00E-14	-11.694174	-8.3495653	24.2588541	0.33417996
cg23889010	6660162	5.90E-11	-11.667661	-7.0747596	17.1053218	0.26139476
cg22016649	6480692	5.37E-11	-11.664392	-7.0922429	18.4340662	0.27609382
cg13471990	5420564	1.03E-10	-11.642748	-6.9706136	19.9017218	0.29166419
cg01525376	2710754	4.90E-11	-11.640169	-7.1094388	28.9041543	0.37422442
cg26991946	4210619	3.80E-14	-11.627534	-8.397489	27.6959011	0.36427963
cg08766149	6020523	2.01E-13	-11.616343	-8.1042519	22.8942244	0.32142369
cg26823505	4210619	4.44E-14	-11.595915	-8.3701825	27.5343774	0.36292617
cg26991946	6590228	6.46E-11	-11.585735	-7.0577602	17.6700065	0.26771382
cg19812619	6590228	6.00E-13	-11.568413	-7.9105411	21.9947488	0.3127449
cg19464252	3170128	8.84E-12	-11.543464	-7.4252135	21.5696488	0.3085655
cg22045288	540368	5.17E-11	-11.522688	-7.0993839	18.401976	0.27574572
cg01500140	7570408	5.77E-15	-11.478405	-8.7206571	26.0895849	0.35055848
cg26191951	4180544	5.64E-12	-11.455014	-7.5070619	20.5403725	0.29823243
cg22016649	540368	1.48E-11	-11.442126	-7.3307042	19.541992	0.2879101
cg08399444	5260424	3.36E-11	-11.434023	-7.1794997	17.6085513	0.26703136
cg15910079	5690148	6.61E-11	-11.311429	-7.0536687	16.5915375	0.2555498
cg20790056	4760338	9.57E-11	-11.301259	-6.9842278	16.3652972	0.25294658
cg05044994	6590228	1.16E-11	-11.287518	-7.3761355	19.226951	0.28458955

cg26991946	5910112	7.07E-13	-11.225568	-7.8812477	21.3420923	0.30630731
cg14859417	540368	4.96E-11	-11.21309	-7.1070965	18.4393967	0.27615161
cg01525376	3520020	2.34E-12	-11.171243	-7.6661546	27.3545455	0.3614125
cg16008138	4760338	1.59E-11	-11.153805	-7.3175218	17.9567877	0.27088181
cg08510456	540368	5.15E-12	-11.139652	-7.5236781	20.5209895	0.29803488
cg17753124	4490528	1.68E-12	-11.130283	-7.7258671	22.4893267	0.31754423
cg20366831	6590228	8.26E-14	-11.108642	-8.2610639	23.915272	0.33101362
cg05037688	5420564	9.07E-11	-11.093689	-6.994425	20.0189287	0.2928788
cg09358725	6480692	7.91E-11	-11.089338	-7.0199037	18.0850631	0.27228997
cg06037693	6020523	2.18E-11	-11.07735	-7.2599135	18.5083868	0.27689872
cg08766149	7570408	7.12E-12	-11.075503	-7.46458	19.2449258	0.28477984
cg23547429	4180544	1.80E-11	-11.05972	-7.294301	19.4632068	0.2870826
cg08766149	60093	5.86E-13	-11.05697	-7.9147233	21.3145322	0.30603281
cg22016649	1450112	9.47E-12	-11.028846	-7.4125455	18.4213999	0.27595646
cg19812619	4760338	1.31E-13	-10.967077	-8.1800943	22.4200036	0.31687556
cg22917487	60093	3.62E-11	-10.936828	-7.1658183	17.5263183	0.26611617
cg17166812	540368	3.09E-11	-10.935351	-7.1952049	18.8697763	0.28078725
cg15958424	3370327	1.06E-11	-10.927623	-7.3925757	18.2235426	0.27380406
cg24427660	5260424	6.56E-11	-10.885871	-7.0549807	17.0138811	0.26036123
cg24474182	5690148	6.31E-12	-10.875354	-7.4866592	18.6903647	0.27886203
cg17753124	540390	4.23E-11	-10.859721	-7.1365572	20.066887	0.2933746
cg15910079	4180544	7.95E-11	-10.839339	-7.0190836	18.1157738	0.27262629
cg01500140	60093	2.35E-14	-10.790727	-8.4803533	24.4250791	0.3357011
cg12417466	6590228	4.28E-11	-10.736906	-7.1347136	18.0400355	0.27179629
cg20981615	5390246	7.11E-12	-10.724868	-7.4649893	31.9180257	0.39772567
cg16935609	5690148	1.03E-10	-10.719175	-6.9710126	16.2050882	0.25109211
cg24474182	4010133	8.41E-11	-10.696963	-7.0084723	16.93214	0.25943488
cg06037693	4210619	1.20E-11	-10.691781	-7.3692836	21.976901	0.31257044
cg23140706	4180544	1.17E-11	-10.641809	-7.3737035	19.8615838	0.29124728
cg19812619	4210619	6.00E-14	-10.623667	-8.3174948	27.2242058	0.36031091
cg21171615	6590228	5.63E-12	-10.621884	-7.507533	19.8895448	0.29153776
cg23181133	540368	1.73E-11	-10.621591	-7.3023547	19.4003119	0.28642061
cg09001777	4180544	7.33E-12	-10.614065	-7.4593276	20.2960087	0.29573369
cg26701826	5690148	4.73E-11	-10.608494	-7.1160209	16.886072	0.25891178
cg15958424	5690148	4.58E-13	-10.587141	-7.9586854	21.1208212	0.30409731
cg26757673	6590228	7.69E-12	-10.544939	-7.450563	19.6008275	0.28852682
cg04353769	3370327	5.87E-12	-10.542513	-7.499871	18.7562272	0.27956998
cg26191951	540368	4.70E-11	-10.501766	-7.117322	18.4890729	0.27668972
cg02266731	5420564	2.16E-11	-10.48149	-7.2608607	21.3576621	0.30646229
cg18669588	4210619	2.21E-12	-10.470156	-7.6766224	23.6081986	0.32815813
cg07548313	6590228	1.07E-11	-10.460663	-7.3904929	19.2987811	0.28534937
cg23889010	540368	2.37E-12	-10.43231	-7.6639515	21.2485911	0.30537516
cg16173109	6590228	8.07E-12	-10.427816	-7.4419426	19.5573316	0.288071
cg18463686	4180544	6.55E-12	-10.410602	-7.4798742	20.4010006	0.29680946

cg22820108	4180544	9.72E-11	-10.399829	-6.9813076	17.9348714	0.27064067
cg08399444	4220187	2.12E-11	-10.395247	-7.264789	18.886992	0.28097145
cg06394229	6480692	9.50E-11	-10.376458	-6.9857508	17.9215345	0.27049385
cg17839611	6590228	2.17E-11	-10.373813	-7.2607585	18.6547897	0.27847906
cg12240237	6020523	8.21E-11	-10.342011	-7.0129893	17.3168622	0.26377472
cg22438810	5690148	6.40E-11	-10.339195	-7.0597461	16.6201311	0.25587752
cg24949488	6590228	1.33E-15	-10.337377	-8.9871533	28.1583848	0.36812332
cg15361231	3370327	5.42E-12	-10.317343	-7.5142368	18.8281311	0.28034128
cg15910079	540368	9.01E-11	-10.306258	-6.995616	17.9024518	0.27028368
cg23889010	4180544	7.79E-11	-10.269042	-7.022822	18.1337297	0.27282279
cg26701826	6480692	2.78E-11	-10.26229	-7.2143707	19.0313955	0.28251276
cg26215727	4180544	1.03E-10	-10.261053	-6.9706883	17.884193	0.27008247
cg05615150	5270544	2.22E-13	-10.260377	-8.0866636	29.6156873	0.37993662
cg26701826	1500524	4.82E-11	-10.256387	-7.1125718	20.4025577	0.29682539
cg09914304	6590228	8.04E-14	-10.225451	-8.2660669	23.9432863	0.33127291
cg02039171	4180544	2.15E-11	-10.194619	-7.2617008	19.3008947	0.2853717
cg01402255	4180544	1.37E-11	-10.156116	-7.344479	19.7144575	0.28971488
cg07285167	6660162	2.56E-11	-10.134433	-7.229533	17.8480776	0.26968415
cg08343834	5910112	5.33E-11	-10.126165	-7.0936032	17.3741733	0.26441687
cg02656594	4180544	4.10E-11	-10.115369	-7.1427435	18.714788	0.27912472
cg26757673	4210619	6.28E-14	-10.098235	-8.3091251	27.1751135	0.35989501
cg02039171	6480692	2.86E-11	-10.096624	-7.2094051	19.0069096	0.28225187
cg12240237	4210619	2.61E-11	-10.095462	-7.2264832	21.2416194	0.30530555
cg08700306	540368	2.41E-11	-10.076257	-7.2413379	19.0972355	0.28321332
cg02600394	6590228	9.88E-12	-10.05357	-7.4049313	19.3711577	0.28611333
cg12380764	3370327	2.56E-11	-10.048363	-7.2295056	17.4286531	0.26502626
cg05615150	4760338	3.83E-12	-10.028865	-7.5773899	19.2491322	0.28482436
cg02656594	6480692	5.52E-11	-10.013317	-7.087152	18.4093881	0.27582615
cg24474182	1500524	9.01E-11	-10.006923	-6.9956723	19.822962	0.29084565
cg18638581	4180544	1.07E-12	-9.9900722	-7.8078861	22.116231	0.31392999
cg11846968	5260424	1.10E-10	-9.984967	-6.9587924	16.5616339	0.25520675
cg23090046	6480692	5.37E-12	-9.9624459	-7.5159791	20.5502594	0.29833315
cg12417466	4760338	3.99E-11	-9.959042	-7.1476698	17.1364696	0.26174616
cg08399444	3370327	8.85E-12	-9.9461457	-7.4250768	18.3840893	0.27555155
cg26757673	4760338	2.80E-12	-9.9402235	-7.6342385	19.5378562	0.28786671
cg16967583	5420564	1.59E-11	-9.9319094	-7.3176847	21.6496549	0.30935597
cg15958424	6480692	1.81E-12	-9.9171251	-7.7126613	21.5742328	0.30861084
cg05615150	5270520	4.58E-12	-9.9142816	-7.5449869	25.4709316	0.34511463
cg19812619	3840554	1.46E-11	-9.9039562	-7.3329153	19.162436	0.28390574
cg07285167	3370327	8.97E-11	-9.9001569	-6.9964676	16.3234757	0.25246337
cg04353769	5420564	5.81E-12	-9.8901974	-7.501809	22.6114229	0.31871873
cg24926276	3370327	5.15E-11	-9.8873619	-7.1002235	16.8110641	0.25805848
cg24474182	540368	1.25E-11	-9.8493521	-7.3616897	19.697474	0.28953756
cg04451770	4180544	1.29E-11	-9.8158743	-7.3565947	19.7753812	0.29035023

cg17676574	4210619	5.50E-11	-9.8042683	-7.0878775	20.5416958	0.29824591
cg06196379	5420564	6.67E-11	-9.8010147	-7.051928	20.3036234	0.29581182
cg15958424	4180544	5.10E-12	-9.7870051	-7.5253657	20.6344877	0.29919008
cg20366831	4760338	2.18E-12	-9.780206	-7.6790807	19.7671243	0.29026419
cg23090046	4180544	1.96E-11	-9.7757712	-7.2789657	19.3867637	0.28627785
cg01525376	6650053	8.17E-14	-9.7574959	-8.2629995	27.044126	0.35878267
cg05564251	6590228	7.04E-11	-9.7516965	-7.0417417	17.5934862	0.26686387
cg18638581	5690148	3.93E-11	-9.7481738	-7.1506169	17.050612	0.26077674
cg16967583	6480692	1.19E-12	-9.7478123	-7.7877112	21.9719321	0.31252186
cg03019000	7150017	1.59E-12	-9.7466597	-7.7363763	32.264629	0.40031569
cg23889010	3310091	1.11E-10	-9.7423121	-6.9558394	17.1652268	0.26207029
cg17813891	6590228	1.44E-14	-9.7393873	-8.5646499	25.6459069	0.34666356
cg26701826	540368	4.68E-11	-9.7275445	-7.1179698	18.4922223	0.27672381
cg07285167	5690148	2.74E-12	-9.703797	-7.6381006	19.4539496	0.28698524
cg16545105	3370327	4.63E-11	-9.6887944	-7.1201182	16.9053767	0.25913107
cg22045288	70343	4.57E-11	-9.6818157	-7.1223173	17.5733902	0.26664033
cg02266731	540368	5.47E-12	-9.6542707	-7.5125967	20.4640829	0.29745424
cg24777950	3370327	8.92E-11	-9.6148441	-6.9975211	16.3283907	0.25252019
cg17823175	4180544	2.71E-11	-9.6099184	-7.2195314	19.0920167	0.28315784
cg20366831	4210619	4.50E-13	-9.6083995	-7.9617638	25.1812746	0.3425343
cg09303642	4180544	1.85E-12	-9.5994313	-7.7087771	21.5902133	0.30876885
cg09303642	5690148	2.01E-11	-9.5719604	-7.2742741	17.6452547	0.26743911
cg11105610	6590228	1.86E-12	-9.5710983	-7.7083064	20.9245734	0.30212541
cg16967583	5690148	4.01E-11	-9.5666773	-7.1465897	17.0314174	0.26055966
cg02656594	540368	6.43E-11	-9.557721	-7.0587432	18.2054612	0.27360672
cg07285167	4180544	7.49E-13	-9.5332368	-7.8710254	22.4548444	0.31721179
cg20981615	3830349	5.93E-12	-9.5306982	-7.4978688	22.197185	0.31471745
cg01124420	6590228	1.77E-13	-9.5300745	-8.1265798	23.1685867	0.32402748
cg02266731	6480692	1.15E-10	-9.523135	-6.9496989	17.749779	0.26859781
cg17839611	4760338	3.73E-11	-9.5111108	-7.1601032	17.1958646	0.26241531
cg02656594	1190528	8.17E-11	-9.509055	-7.0140191	16.9777427	0.25995197
cg02600394	5390730	7.41E-12	-9.5064346	-7.4574085	28.7386048	0.37288027
cg18638581	6480692	1.49E-11	-9.5054882	-7.3290678	19.6016674	0.28853562
cg16967583	4180544	7.04E-12	-9.5006569	-7.4668389	20.3343576	0.296127
cg15958424	1500524	3.94E-11	-9.4813666	-7.1501675	20.5910014	0.29874792
cg02600394	5270544	9.28E-13	-9.4742861	-7.8325689	28.117056	0.36778172
cg01402255	540368	4.70E-11	-9.4598208	-7.1173057	18.4889938	0.27668886
cg03000846	150762	3.30E-11	-9.4502982	-7.1830278	20.1716474	0.29445519
cg15055101	6590228	1.03E-10	-9.438296	-6.9701808	17.2537642	0.26306644
cg02473123	5390246	7.02E-11	-9.4320577	-7.0423243	29.4665255	0.3787478
cg09914304	4760338	1.09E-13	-9.4296264	-8.2119374	22.5942787	0.31855406
cg24474182	3370041	8.13E-12	-9.3900986	-7.4405043	18.6740533	0.27868649
cg18338021	4210619	3.12E-13	-9.3751521	-8.0269858	25.5491545	0.34580799
cg26757673	3840554	2.24E-11	-9.3463551	-7.2540993	18.7720418	0.27973976

cg15361231	5690148	1.33E-11	-9.3342798	-7.3500817	18.0148389	0.27151975
cg05615150	3840554	5.12E-11	-9.3322487	-7.1010782	18.0261395	0.2716438
cg08399444	6480692	1.94E-13	-9.3199895	-8.11113	23.729828	0.32929208
cg02374486	6590228	6.59E-14	-9.3120255	-8.3004964	24.1365339	0.33305614
cg16545105	6480692	2.77E-13	-9.3116647	-8.0477355	23.3796209	0.3260167
cg24474182	1450112	2.51E-11	-9.2969168	-7.2333877	17.5453627	0.26632832
cg12380764	4180544	2.02E-12	-9.2922604	-7.6926474	21.5052413	0.30792784
cg22670733	6590228	3.57E-12	-9.2829738	-7.5901997	20.3124006	0.29590186
cg07509155	4180544	5.86E-11	-9.2744378	-7.0760487	18.3904157	0.27562024
cg01861509	6590228	5.22E-13	-9.2721133	-7.9352816	22.1275718	0.31404041
cg25226014	6590228	1.86E-12	-9.2675366	-7.7076101	20.9209367	0.30208876
cg15361231	6480692	1.70E-12	-9.2634016	-7.7243969	21.6361671	0.30922283
cg01500140	1440564	3.05E-12	-9.2560114	-7.6187949	19.5483503	0.2879768
cg15958424	730528	1.52E-11	-9.2430026	-7.3262226	18.0823057	0.27225976
cg09914304	4210619	2.24E-14	-9.2357666	-8.4886147	28.2387359	0.36878638
cg16003913	150762	4.01E-11	-9.2338414	-7.1469143	19.9913201	0.29259307
cg18350391	6590228	2.06E-11	-9.2332825	-7.2700657	18.7006102	0.27897225
cg26701826	1450112	7.06E-11	-9.2261033	-7.041174	16.6293175	0.25598274
cg15361231	4180544	2.91E-12	-9.2202225	-7.6270241	21.1613694	0.30450334
cg04784315	6480692	4.28E-12	-9.2106216	-7.5573311	20.7633521	0.30049708
cg25839227	4760338	3.79E-11	-9.1983866	-7.1569347	17.1807188	0.26224479
cg15958424	540368	1.19E-11	-9.1894255	-7.3701683	19.740133	0.28998278
cg17753124	6270095	5.22E-11	-9.1882111	-7.0976143	17.3674263	0.26434133
cg04353769	540368	8.08E-13	-9.1843018	-7.8574272	22.2742079	0.315465
cg24612198	6590228	1.00E-17	-9.180853	-9.5697049	31.8209812	0.39699649
cg05615150	5050347	1.48E-11	-9.173498	-7.3307838	20.7165337	0.30002279
cg02266731	1190528	8.79E-11	-9.1632125	-7.0001828	16.9125295	0.25921229
cg04784315	4180544	7.51E-12	-9.1604317	-7.4550575	20.2742249	0.29551008
cg24821554	4180544	7.46E-11	-9.1560592	-7.0308644	18.17239	0.27324551
cg11403598	4210619	2.71E-11	-9.1550271	-7.219458	21.2058177	0.3049479
cg15910079	450202	2.67E-11	-9.1505222	-7.2218026	17.9785295	0.27112086
cg12417466	5050347	1.09E-10	-9.146932	-6.9597671	18.8744088	0.28083682
cg22381196	5420564	7.02E-11	-9.1154376	-7.0422696	20.2556424	0.29531921
cg21917349	6590228	1.87E-11	-9.113519	-7.2881103	18.7896133	0.27992831
cg08399444	4180544	1.16E-12	-9.1027978	-7.7922832	22.0329733	0.31311823
cg16967583	3310091	6.09E-12	-9.1012014	-7.4930883	19.7944232	0.29054858
cg16967583	540368	5.36E-12	-9.0992765	-7.5163312	20.4832514	0.29764993
cg24821554	6480692	8.31E-11	-9.0968755	-7.0107677	18.0412407	0.27180951
cg12125117	4180544	2.11E-12	-9.0908905	-7.6851997	21.4660663	0.30753941
cg16545105	5690148	1.78E-11	-9.0688735	-7.2965003	17.7532172	0.26863586
cg05790038	4210619	3.75E-12	-9.0570422	-7.5810251	23.0936449	0.32331824
cg20720686	3370327	5.35E-12	-9.0532939	-7.5168364	18.8411571	0.28048083
cg24898863	5690148	1.19E-11	-9.0455424	-7.3704844	18.1149629	0.27261742
cg16545105	4180544	2.28E-12	-9.0429696	-7.6709628	21.3912859	0.30679674

cg07285167	6480692	1.34E-11	-9.0377139	-7.3492089	19.7027372	0.28959252
cg13765621	450202	1.04E-10	-9.0299484	-6.9687706	16.7708268	0.25759993
cg00899659	3370327	8.11E-11	-9.0244016	-7.015352	16.4116867	0.25348184
cg22381196	4180544	5.35E-12	-9.0132588	-7.5168837	20.5908459	0.29874633
cg16967583	1030270	1.80E-11	-9.0070571	-7.2948888	19.1746473	0.28403527
cg16173109	3840554	9.01E-11	-9.0050168	-6.9956461	17.5214629	0.26606206
cg00071250	6590228	1.69E-11	-8.997214	-7.3059786	18.877964	0.28087486
cg20987610	60093	1.12E-10	-8.9947395	-6.9549578	16.5275962	0.2548159
cg25712380	4210619	4.74E-11	-8.9872587	-7.1154305	20.6797524	0.29964973
cg04126866	1850546	3.55E-11	-8.9670856	-7.1692524	22.3750218	0.31644099
cg20366831	3840554	6.77E-11	-8.9655376	-7.0490403	17.7761038	0.26888905
cg12044210	6590228	7.03E-13	-8.9613752	-7.8823824	21.844079	0.31126937
cg08399444	1500524	4.41E-12	-8.9516726	-7.5516334	22.6652415	0.31923516
cg01782486	5270544	7.07E-11	-8.949572	-7.0409862	23.7550313	0.32952657
cg21237418	3310091	2.43E-11	-8.944653	-7.2397312	18.5301798	0.2771344
cg25600606	3370327	8.94E-11	-8.9412935	-6.9969572	16.3257599	0.25248978
cg00620024	6590228	1.09E-11	-8.9166028	-7.3868525	19.2805553	0.28515673
cg15958424	5420672	1.07E-10	-8.8993096	-6.9625374	17.4862052	0.26566891
cg23547429	1440243	7.45E-11	-8.884767	-7.0312226	16.7382625	0.2572284
cg25839227	4210619	2.46E-11	-8.8826826	-7.2372908	21.2967644	0.30585573
cg23889010	450202	6.95E-12	-8.8808572	-7.4690239	19.2000953	0.28430506
cg08399444	5690148	4.27E-11	-8.8803497	-7.1347994	16.9752842	0.25992411
cg21991396	540368	7.92E-11	-8.8786223	-7.0198372	18.0183912	0.27155875
cg14859417	6270022	2.93E-11	-8.8785225	-7.2045523	17.3996606	0.26470209
cg11024597	3370327	1.17E-11	-8.8750561	-7.3737919	18.1310769	0.27279377
cg13650156	6480692	4.52E-12	-8.8447752	-7.5472535	20.7113131	0.29996986
cg15337006	6480692	6.17E-11	-8.8441671	-7.0663366	18.3086689	0.27473168
cg24841244	6590228	2.22E-16	-8.8321589	-9.3338003	30.310105	0.38541175
cg21126943	4180544	1.85E-11	-8.8241129	-7.289628	19.4398957	0.28683738
cg07285167	1500524	7.99E-11	-8.8227566	-7.0181943	19.9338807	0.29199787
cg07285167	540368	4.70E-12	-8.8143977	-7.5401858	20.6059179	0.29889965
cg01500140	3390075	5.85E-11	-8.8068759	-7.0763574	18.5512132	0.27736173
cg08539991	6590228	9.41E-13	-8.7959341	-7.8300861	21.5656812	0.30852626
cg13650156	5690148	6.59E-11	-8.7910121	-7.0541851	16.593966	0.25557764
cg06196379	540368	7.30E-11	-8.7908347	-7.0349085	18.090735	0.27235211
cg10126923	6660162	3.78E-11	-8.7833492	-7.1574332	17.5000717	0.26582358
cg12380764	6480692	3.78E-11	-8.7811153	-7.1577369	18.7531303	0.27953672
cg15337006	4180544	1.15E-10	-8.7736563	-6.9504382	17.7877677	0.26901802
cg03574571	3310091	6.49E-11	-8.7730407	-7.0570738	17.6456991	0.26744404
cg04784315	1500524	1.13E-10	-8.7509338	-6.9534525	19.6159939	0.28868562
cg01500140	7000270	3.79E-11	-8.7405077	-7.1573941	18.203982	0.27359057
cg16545105	1500524	2.22E-11	-8.7353975	-7.2560772	21.127201	0.30416122
cg23228178	4210619	3.02E-12	-8.7350868	-7.6202869	23.3041919	0.32530705
cg09914304	3840554	3.18E-12	-8.7245331	-7.6112692	20.5749462	0.29858453

cg16545105	1030270	2.35E-12	-8.7155803	-7.6660218	21.0657074	0.30354465
cg24474182	610113	9.65E-11	-8.7149409	-6.9827737	17.2913658	0.26348869
cg24777950	4180544	2.54E-11	-8.7107375	-7.2312252	19.1498181	0.28377184
cg01500140	520553	1.75E-12	-8.7048636	-7.7185656	19.8904115	0.29154676
cg09303642	3310091	4.33E-11	-8.6701638	-7.1324928	18.0081609	0.27144642
cg08766149	520553	1.12E-10	-8.6661357	-6.9549205	16.1535655	0.25049376
cg12619509	150762	8.16E-11	-8.6658445	-7.0141406	19.3361481	0.285744
cg13531460	4210619	1.40E-13	-8.6627433	-8.1678628	26.354003	0.35285772
cg24777950	6480692	2.92E-11	-8.6503206	-7.2056007	18.9881614	0.28205199
cg24474182	450202	6.24E-12	-8.6473541	-7.4886241	19.2987024	0.28534854
cg27461196	4180544	7.67E-11	-8.6317763	-7.0257242	18.1476753	0.27297533
cg07285167	1940041	6.69E-12	-8.6239805	-7.4760034	18.8441707	0.28051311
cg24926276	4180544	1.11E-10	-8.610165	-6.9569145	17.8185757	0.26935845
cg12125117	6480692	3.59E-11	-8.605067	-7.1671296	18.7991292	0.28003038
cg05564251	4210619	1.02E-10	-8.587409	-6.9719147	19.9665276	0.29233628
cg02473123	6590228	9.28E-14	-8.5841792	-8.2406944	23.8013888	0.32995745
cg22242539	4180544	3.59E-11	-8.5796961	-7.1674693	18.835816	0.28042362
cg22381196	6480692	6.17E-11	-8.5772746	-7.066359	18.3087776	0.27473286
cg15880738	6590228	8.88E-16	-8.5669162	-9.0541431	28.567859	0.37148786
cg20981615	5390730	6.43E-13	-8.544031	-7.8982591	31.3393478	0.39335124
cg04404982	6590228	1.32E-11	-8.5237735	-7.3520793	19.1069104	0.28331615
cg17709873	4760338	1.62E-11	-8.5206722	-7.3144413	17.9417384	0.27071624
cg07285167	730528	5.34E-11	-8.5158578	-7.0933177	16.9596141	0.25974649
cg06625767	4180544	9.31E-12	-8.5148482	-7.4157793	20.074434	0.29345255
cg07285167	1450112	2.99E-12	-8.4933781	-7.6223751	19.4746647	0.28720306
cg01550148	150762	3.52E-11	-8.4860311	-7.1710034	20.1115043	0.29383523
cg20981615	5270544	8.50E-14	-8.4773423	-8.2557924	30.6397152	0.38797686
cg16967583	1450112	2.27E-11	-8.4767735	-7.2521714	17.6362048	0.26733861
cg15361231	1190528	2.36E-11	-8.4642111	-7.2444881	18.08292	0.27226649
cg07924575	3400646	4.86E-11	-8.4633088	-7.1109898	17.1351754	0.26173157
cg15958424	540390	2.32E-12	-8.4595832	-7.6682597	22.8149542	0.32066765
cg27461196	540368	1.85E-11	-8.4551472	-7.2896805	19.3371488	0.28575456
cg15361231	5420672	4.48E-11	-8.4487262	-7.1262083	18.2706628	0.27431782
cg00899659	5690148	3.55E-11	-8.4308074	-7.1691166	17.1389247	0.26177385
cg04784315	1030270	9.26E-11	-8.4245021	-6.9904411	17.6934232	0.26797353
cg00071250	4760338	9.58E-12	-8.420829	-7.4104867	18.413929	0.27587542
cg07285167	1190528	5.66E-11	-8.4171729	-7.0826441	17.3030876	0.26362022
cg08539991	4210619	4.88E-15	-8.4155622	-8.7509891	29.8344172	0.38167169
cg15055101	5050347	2.69E-11	-8.4079598	-7.2203792	20.1583702	0.29431842
cg15958424	3370041	7.47E-11	-8.3967593	-7.0307911	16.6911724	0.25669049
cg17813891	4760338	1.98E-12	-8.3825412	-7.6962848	19.8554412	0.29118343
cg22242539	6480692	9.45E-11	-8.3817638	-6.9866552	17.925855	0.27054142
cg15361231	1450112	3.40E-12	-8.3810575	-7.5989706	19.3557263	0.28595059
cg24949488	4760338	1.03E-11	-8.3795833	-7.3969311	18.3469115	0.27514763

cg26928972	5690148	5.10E-11	-8.3768362	-7.10184	16.8188575	0.25814723
cg24926276	730528	6.63E-11	-8.3766118	-7.0530857	16.7693542	0.25758313
cg05681757	450202	5.93E-11	-8.3686569	-7.0738076	17.266932	0.26321436
cg02600394	5050347	9.25E-11	-8.362306	-6.9907018	19.0243463	0.28243767
cg08368934	4180544	8.07E-11	-8.3552797	-7.0162844	18.1023356	0.27247917
cg25226014	5270544	2.45E-12	-8.3520535	-7.6582795	27.1167722	0.35940006
cg22381196	540368	2.69E-11	-8.343969	-7.2209042	18.9963076	0.28213885
cg24427660	4180544	2.23E-11	-8.3398654	-7.2552312	19.2687698	0.28503211
cg22670733	4760338	2.06E-11	-8.3344289	-7.2698079	17.7244026	0.26831683
cg24427660	6480692	1.84E-11	-8.3337729	-7.2911438	19.4121146	0.28654493
cg08399444	1940041	5.75E-12	-8.3150092	-7.5035532	18.9821716	0.2819881
cg16545105	540368	1.80E-11	-8.3123468	-7.2944098	19.3607052	0.28600311
cg15958424	2140288	1.78E-11	-8.3055378	-7.2969707	18.4066039	0.27579594
cg21969640	5420564	4.59E-11	-8.3005253	-7.1217549	20.6524674	0.29937273
cg08458487	4180544	5.87E-11	-8.2994783	-7.0758938	18.3896659	0.2756121
cg15361231	730528	1.13E-10	-8.2968996	-6.9527766	16.2996974	0.25218835
cg07218880	6590228	1.07E-10	-8.2825826	-6.9637351	17.2233349	0.26272438
cg08458487	6480692	5.56E-11	-8.2720111	-7.0857434	18.4025628	0.27575209
cg20981615	3990224	1.33E-11	-8.2665717	-7.3506635	24.88561	0.33987939
cg08399444	1030270	3.82E-11	-8.2535429	-7.1559198	18.4906915	0.27670724
cg25600606	4180544	9.47E-12	-8.2488421	-7.4125753	20.0581834	0.29328467
cg09303642	1450112	4.97E-11	-8.2473945	-7.1066973	16.9388147	0.25951061
cg08519905	6480692	7.68E-11	-8.2437184	-7.0256357	18.1125868	0.27259141
cg20720686	4180544	9.35E-13	-8.2414262	-7.831316	22.2415664	0.3151484
cg26928972	6480692	1.24E-11	-8.2378944	-7.3635006	19.7746223	0.29034233
cg15361231	540368	1.04E-10	-8.2276808	-6.9694325	17.7775701	0.26890527
cg21969640	4180544	3.19E-12	-8.2118921	-7.6105106	21.075301	0.30364091
cg22381196	1190528	6.21E-11	-8.2074566	-7.0651597	17.2198948	0.26268569
cg02039171	70343	1.59E-11	-8.1913397	-7.3175438	18.5226385	0.27705287
cg23668631	6270022	1.06E-10	-8.1905148	-6.9642343	16.2629645	0.2517631
cg09914304	5050347	1.16E-11	-8.1892116	-7.3749408	20.9421459	0.30230243
cg02374486	4760338	1.96E-12	-8.1868576	-7.6987734	19.8682329	0.29131637
cg15958424	7550500	5.36E-11	-8.1856728	-7.0927404	16.827572	0.25824644
cg15361750	540368	4.24E-11	-8.1830274	-7.1364663	18.5822691	0.27769711
cg22016649	5550279	5.09E-11	-8.1813674	-7.1022332	16.9603415	0.25975474
cg24949488	4210619	3.63E-12	-8.1738576	-7.5870258	23.125754	0.3236223
cg22242539	540368	3.90E-11	-8.1651366	-7.1520016	18.6580804	0.2785145
cg24898863	1030270	5.12E-11	-8.1623655	-7.1011397	18.2246986	0.27381667
cg07728874	6590228	1.00E-17	-8.1599628	-9.5424849	31.6447232	0.39566757
cg23889010	70343	1.03E-10	-8.159959	-6.9712894	16.856657	0.25857738
cg08399444	540368	3.83E-11	-8.1583094	-7.1550136	18.6727979	0.27867298
cg24777950	540368	7.02E-11	-8.1420661	-7.0423833	18.1266726	0.27274558
cg22438810	450202	1.05E-10	-8.1392555	-6.967618	16.7654238	0.25753831
cg11024597	5690148	1.37E-11	-8.1354305	-7.3446952	17.988452	0.27122991

cg16967583	4490528	8.04E-13	-8.1306704	-7.858144	23.2025753	0.32434865
cg21126943	540368	1.10E-10	-8.1263787	-6.9584547	17.7253513	0.26832734
cg26928972	4180544	3.28E-11	-8.1256904	-7.1841885	18.9178902	0.2813018
cg13650156	540368	4.47E-11	-8.1205034	-7.1263588	18.5330339	0.27716526
cg16545105	1190528	6.89E-11	-8.0992103	-7.045729	17.1276829	0.26164707
cg10161121	6590228	2.50E-12	-8.0938347	-7.6545591	20.6448172	0.29929502
cg21969640	5690148	6.10E-11	-8.0904882	-7.0687018	16.6623125	0.25636044
cg25600606	6480692	2.31E-11	-8.0802421	-7.2487873	19.2015696	0.28432068
cg20720686	6480692	2.59E-12	-8.0695064	-7.6483198	21.236345	0.30525289
cg24474182	70343	4.44E-11	-8.0625819	-7.1277518	17.5994669	0.26693037
cg17813891	3840554	5.09E-12	-8.0487079	-7.5259225	20.13626	0.29409055
cg21969640	6480692	8.57E-12	-8.0370382	-7.4309784	20.1159117	0.2938807
cg03330678	4180544	1.73E-11	-8.0031563	-7.3018438	19.500865	0.28747837
cg04759756	6590228	9.33E-15	-8.0008965	-8.640796	26.0897853	0.35056023
cg16545105	1450112	1.37E-11	-7.9875076	-7.3445315	18.0863044	0.27230357
cg00899659	6480692	6.64E-11	-7.9761156	-7.0528334	18.2434899	0.27402163
cg20720686	5690148	5.88E-11	-7.9621987	-7.0753096	16.6934693	0.25671675
cg24898863	540368	1.01E-10	-7.9605474	-6.9734646	17.7967708	0.26911754
cg22670733	5050347	7.85E-12	-7.9462565	-7.4469886	21.3131703	0.30601924
cg27485921	3370327	9.42E-11	-7.9439135	-6.9872586	16.2805461	0.2519667
cg24427660	1030270	4.53E-11	-7.934303	-7.1242111	18.3364762	0.27503418
cg01124420	4760338	8.64E-11	-7.9338626	-7.0034474	16.4550559	0.25398156
cg03330678	6480692	2.21E-11	-7.9323294	-7.2567128	19.2408721	0.28473693
cg22820108	6270022	3.51E-11	-7.9271313	-7.1711407	17.2393172	0.26290408
cg24427660	540368	2.82E-11	-7.914641	-7.2118811	18.9518307	0.28166433
cg05615150	6180088	2.63E-11	-7.9086955	-7.2251597	17.8986168	0.27024143
cg21969640	540368	1.93E-12	-7.8941947	-7.7016041	21.446184	0.30734211
cg07218880	4760338	2.62E-11	-7.8851487	-7.2253135	17.5090678	0.2659239
cg09001777	6270022	1.11E-11	-7.8718406	-7.3830674	18.268991	0.2742996
cg01718139	4180544	2.39E-11	-7.870621	-7.2428354	19.2072984	0.28438139
cg09914304	2710754	6.13E-11	-7.8685994	-7.0678042	28.6710077	0.37232976
cg11024597	6480692	8.22E-12	-7.862666	-7.4384533	20.15391	0.29427247
cg04451770	450202	7.83E-11	-7.8396231	-7.0217871	17.0203039	0.26043392
cg04404982	4760338	2.03E-11	-7.8331167	-7.2725077	17.7375109	0.268462
cg18084554	4180544	3.77E-11	-7.8324616	-7.158048	18.7896511	0.27992872
cg08399444	5420201	9.58E-13	-7.8274304	-7.8269984	20.7528775	0.30039102
cg08044694	540368	2.97E-11	-7.827087	-7.2020671	18.9035184	0.28114818
cg08399444	1450112	3.46E-11	-7.8157308	-7.1740754	17.2600628	0.2631372
cg16967583	70343	1.33E-12	-7.8087777	-7.7683729	20.812777	0.30099708
cg21991396	450202	3.95E-11	-7.8046797	-7.1496876	17.6299416	0.26726905
cg17813891	5270544	3.08E-11	-7.8037219	-7.1958022	24.5716229	0.33703639
cg10599444	150762	1.06E-10	-7.7965587	-6.9643723	19.093732	0.28317608
cg00795812	6590228	4.93E-13	-7.7959888	-7.9453135	22.1815478	0.31456549
cg02600394	6180088	2.31E-12	-7.7959035	-7.6689332	20.1184977	0.29390737

cg12836863	5690148	1.97E-11	-7.7934157	-7.2783912	17.6652282	0.26766081
cg17813891	4210619	1.11E-11	-7.7925435	-7.3832382	22.0495251	0.31327976
cg08539991	4760338	1.34E-11	-7.786387	-7.3492666	18.1122392	0.2725876
cg11024597	4180544	1.89E-11	-7.7788931	-7.2861353	19.4224827	0.2866541
cg24323726	4210619	1.11E-12	-7.7704499	-7.8013283	24.2891216	0.33445746
cg08399444	3370041	2.83E-11	-7.762847	-7.2114176	17.5515083	0.26639676
cg23547429	6270095	9.84E-11	-7.7548021	-6.9790157	16.8060199	0.25800103
cg16173109	6180088	2.18E-11	-7.7437745	-7.2594698	18.0655274	0.27207586
cg24427660	3310091	1.11E-10	-7.7246075	-6.9568653	17.1700612	0.26212475
cg02240622	4180544	2.62E-11	-7.721476	-7.225839	19.1231828	0.28348904
cg15958424	450202	6.74E-11	-7.7121863	-7.0498287	17.1530224	0.26193277
cg00071250	5050347	3.36E-11	-7.7082335	-7.179245	19.9525768	0.29219171
cg23090046	70343	3.98E-11	-7.7072135	-7.1479999	17.6968003	0.26801097
cg25226014	5050347	2.31E-11	-7.6940169	-7.2487956	20.3012229	0.2957872
cg24427660	1450112	1.23E-11	-7.6845523	-7.3648463	18.186069	0.27339496
cg09914304	3890689	3.64E-11	-7.6830079	-7.1644364	18.1497003	0.27299748
cg06196379	450202	5.08E-11	-7.6776919	-7.1028473	17.4054002	0.26476628
cg11283860	5690148	6.53E-11	-7.6671217	-7.0559552	16.6022922	0.25567309
cg07285167	3400646	7.28E-11	-7.6619754	-7.0354841	16.777601	0.25767717
cg15518883	6590228	2.53E-14	-7.6518245	-8.467929	25.087757	0.34169687
cg10126923	4180544	8.61E-11	-7.6489605	-7.0041308	18.044051	0.27184034
cg07548313	6180088	7.08E-11	-7.632658	-7.0406624	17.01464	0.26036982
cg17813891	5050347	1.25E-11	-7.6061501	-7.3616098	20.8738911	0.30161434
cg13650156	360475	2.13E-11	-7.5943669	-7.2638528	17.7126705	0.26818686
cg24612198	4760338	2.79E-13	-7.5942969	-8.0464804	21.6961245	0.30981426
cg11283860	4180544	1.42E-11	-7.590908	-7.3389761	19.6868191	0.28942627
cg07509155	150762	3.39E-11	-7.5829414	-7.1777524	20.145249	0.29418321
cg26701826	6270022	6.71E-11	-7.5716555	-7.050852	16.6682191	0.25642802
cg22534509	6590228	6.73E-14	-7.5714327	-8.29707	24.117266	0.33287877
cg24949488	5050347	7.14E-11	-7.5693117	-7.0392259	19.2608783	0.28494863
cg12380764	4490528	4.82E-12	-7.5561586	-7.5355571	21.4843998	0.30772125
cg20981615	7150017	1.57E-12	-7.5539377	-7.7383981	32.2769384	0.40040727
cg19812619	6650053	3.85E-13	-7.548795	-7.9895491	25.4734115	0.34513663
cg07285167	540390	5.55E-11	-7.5364313	-7.0863647	19.8176672	0.29079055
cg16967583	2030088	1.13E-10	-7.5286273	-6.9538925	16.7812558	0.25771883
cg04404982	5270520	1.06E-10	-7.5221152	-6.9642727	22.3913261	0.31659857
cg22016649	110347	6.43E-11	-7.5174439	-7.058599	16.861026	0.25862707
cg20981615	3840554	9.81E-11	-7.5048623	-6.9796013	17.4453223	0.26521251
cg15958424	6270022	1.29E-12	-7.4842111	-7.774225	20.2482323	0.29524308
cg12380764	450202	9.45E-12	-7.4793702	-7.4131113	18.9202231	0.28132673
cg11283860	6480692	2.49E-11	-7.479222	-7.2347095	19.1318633	0.28358123
cg00974864	4180544	2.12E-11	-7.4586441	-7.2648596	19.31659	0.2855375
cg07073964	7160059	2.28E-11	-7.4579596	-7.2510407	17.8550083	0.26976062
cg15880738	4760338	9.53E-14	-7.4547384	-8.2362829	22.7279769	0.31983616

cg20981615	4200541	1.96E-11	-7.4505259	-7.2794044	27.1338094	0.35954468
cg02473123	5270544	1.47E-12	-7.4471802	-7.749761	27.6389974	0.36380347
cg15361231	450202	1.14E-11	-7.441794	-7.3789548	18.7502863	0.27950618
cg16545105	150762	8.14E-13	-7.4401681	-7.8559761	23.698185	0.32899744
cg21969640	1030270	9.38E-11	-7.4390605	-6.9880439	17.6820106	0.26784698
cg24612198	5050347	1.35E-14	-7.4285518	-8.5757661	27.5969683	0.36345132
cg15958424	70343	9.52E-11	-7.4039514	-6.9852767	16.9223915	0.25932424
cg06625767	1450112	1.15E-10	-7.3974703	-6.9497446	16.2022449	0.25105911
cg08399444	70343	4.03E-13	-7.3961822	-7.9812758	21.9418721	0.31222779
cg01980222	1440243	2.32E-11	-7.3944584	-7.2482656	17.7754768	0.26888212
cg04353769	450202	5.76E-11	-7.3861448	-7.0793227	17.2931854	0.26350911
cg20720686	540368	3.28E-11	-7.3823431	-7.1839443	18.8144765	0.28019494
cg25600606	1450112	2.71E-11	-7.3743682	-7.2195689	17.4786827	0.26558498
cg10161121	4760338	6.67E-12	-7.3720938	-7.4767409	18.7432462	0.27943056
cg03330678	540368	1.05E-10	-7.3675863	-6.966366	17.7629754	0.26874383
cg18084554	540368	7.79E-11	-7.3608923	-7.0227791	18.0325004	0.27171361
cg07285167	70343	6.41E-12	-7.3487396	-7.4838566	19.3515472	0.2859065
cg15361231	3930343	3.00E-11	-7.3336702	-7.2002099	18.9610591	0.28176284
cg09914304	6180133	5.23E-11	-7.3290142	-7.0973899	18.6946496	0.27890813
cg26757673	6650053	1.01E-13	-7.3260697	-8.2262925	26.8302077	0.35695774
cg14435807	6270022	1.09E-10	-7.3260477	-6.9593682	16.2403462	0.25150102
cg20981615	160070	7.88E-13	-7.3249505	-7.8618516	28.7936112	0.37332752
cg15880738	4210619	1.62E-14	-7.3200087	-8.546802	28.5884221	0.37165587
cg08399444	540390	3.42E-11	-7.3152743	-7.1764529	20.2662356	0.29542803
cg08399444	540274	7.41E-12	-7.3126204	-7.457426	19.2421837	0.28475082
cg15958424	6270095	9.02E-13	-7.2984405	-7.8376311	21.0840861	0.30372904
cg24841244	4760338	8.55E-13	-7.2911608	-7.8473184	20.6392548	0.29923851
cg02473123	4760338	1.89E-11	-7.2882251	-7.2853532	17.7999471	0.26915264
cg25087423	5270520	1.16E-11	-7.2821126	-7.3757017	24.5477326	0.33681907
cg07073964	4890743	2.84E-11	-7.2425944	-7.2106098	18.4064466	0.27579424
cg07285167	3930343	1.02E-10	-7.2406715	-6.9724386	17.8574665	0.26978774
cg22381196	4490528	2.80E-11	-7.2182014	-7.2132262	19.8395136	0.29101783
cg15958424	7400653	1.11E-10	-7.2164959	-6.9558106	16.1936684	0.25095957
cg24926276	450202	5.93E-11	-7.2061424	-7.0739717	17.2677127	0.26322313
cg00974864	6480692	1.04E-10	-7.200959	-6.9690476	17.8418479	0.2696154
cg15361231	70343	1.14E-11	-7.1938651	-7.3793394	18.8284549	0.28034475
cg10126923	1940041	7.72E-11	-7.1932319	-7.0246485	16.6555532	0.2562831
cg12125117	2030088	5.22E-11	-7.1653158	-7.0977751	17.462112	0.26540001
cg20720686	1450112	1.51E-11	-7.1614535	-7.3271936	18.0013773	0.27137191
cg22381196	450202	4.70E-11	-7.1587342	-7.1173249	17.4746451	0.26553992
cg16545105	70343	4.05E-12	-7.1554387	-7.5671933	19.7739057	0.29033486
cg02473123	5390730	1.10E-10	-7.1546453	-6.9588338	25.9772664	0.34957686
cg27485921	4180544	3.96E-11	-7.1444087	-7.149082	18.7457739	0.27945771
cg24841244	4210619	2.11E-13	-7.136502	-8.0962121	25.9429034	0.34927595

cg02374486	5050347	1.11E-10	-7.1232379	-6.9572606	18.8622889	0.28070711
cg19464252	4210619	1.65E-11	-7.1194037	-7.3112617	21.6764098	0.3096199
cg24841244	5050347	5.51E-14	-7.1190127	-8.3323772	26.167175	0.35123485
cg04451770	6270022	6.33E-11	-7.1166754	-7.0616946	16.7193005	0.25701189
cg09106999	4890743	1.42E-11	-7.0887656	-7.338685	19.0377543	0.28258048
cg08399444	450202	3.53E-11	-7.0857574	-7.1704159	17.7297792	0.26837638
cg23889010	110719	6.80E-11	-7.071007	-7.0483582	17.6545163	0.26754192
cg24612198	5270544	7.36E-12	-7.0347355	-7.4587335	25.9991727	0.34976855
cg25915982	5090739	7.47E-12	-7.0207511	-7.4558878	19.9492843	0.29215758
cg15880738	3840554	1.38E-12	-6.9795794	-7.7613619	21.3584299	0.30646993
cg12044210	3890689	3.10E-11	-6.9789626	-7.1941533	18.2941915	0.27457408
cg24612198	3840554	1.06E-11	-6.976608	-7.3915954	19.4558314	0.28700503
cg17078393	6590228	4.00E-12	-6.9764937	-7.5697182	20.2072022	0.29482119
cg00071250	6180133	8.12E-11	-6.9750285	-7.01515	18.2964694	0.27459888
cg25226014	6180088	2.50E-12	-6.973639	-7.6548838	20.0461908	0.29316072
cg15518883	1030296	5.27E-11	-6.9673269	-7.0959294	31.1221682	0.39169306
cg25600606	450202	2.87E-12	-6.9613739	-7.6297239	20.0161913	0.29285048
cg22016649	7160059	5.39E-11	-6.9581768	-7.0917724	17.0897508	0.26121897
cg18638581	6270022	5.68E-11	-6.9552932	-7.0817866	16.8141651	0.2580938
cg14859417	7160059	6.28E-11	-6.9550535	-7.0630352	16.9534835	0.25967698
cg10126923	360475	1.52E-11	-6.9502233	-7.3261635	18.0162824	0.2715356
cg04759756	4760338	8.08E-13	-6.9467555	-7.8572681	20.691425	0.29976816
cg24211388	4180544	1.05E-10	-6.9266425	-6.9671507	17.8673279	0.26989652
cg08399444	4490528	1.11E-10	-6.9170542	-6.9569612	18.5830717	0.27770577
cg25634666	4180544	2.44E-11	-6.9159348	-7.238336	19.1850112	0.28414517
cg11024597	1450112	7.00E-11	-6.9138684	-7.0427598	16.6367742	0.25606813
cg09303642	6270022	1.61E-11	-6.9069816	-7.315187	17.9359208	0.27065222
cg24211388	6480692	1.01E-10	-6.9016281	-6.9739513	17.8652224	0.2698733
cg22242539	70343	2.66E-11	-6.8940016	-7.2225554	18.0575712	0.27198863
cg24841244	3840554	6.46E-12	-6.8765877	-7.4823621	19.9142638	0.29179436
cg25087423	5270544	1.14E-10	-6.8703614	-6.9518107	23.2927277	0.32519906
cg24612198	4210619	9.39E-12	-6.8638398	-7.4141431	22.210853	0.31485023
cg12022621	6650053	5.30E-11	-6.8622903	-7.0947423	20.7030497	0.29988607
cg10161121	5270544	1.01E-10	-6.8459186	-6.9743875	23.4092131	0.32629471
cg07285167	6270022	8.24E-12	-6.8417303	-7.4380519	18.5410399	0.27725179
cg20981615	6180088	9.43E-13	-6.8256961	-7.8297161	20.9554253	0.30243615
cg15361231	6770639	9.81E-11	-6.8201921	-6.979682	16.6214563	0.2558927
cg01500140	6370014	3.59E-11	-6.8175106	-7.1672749	20.9246589	0.30212627
cg04353769	6270095	3.66E-12	-6.8085347	-7.5855773	19.7768116	0.29036514
cg12125117	6270022	1.89E-12	-6.805214	-7.7053245	19.8921865	0.29156519
cg15880738	5050347	6.06E-13	-6.7911567	-7.9087323	23.7766435	0.32972752
cg14654385	4180544	9.28E-11	-6.7890851	-6.9900333	17.9765702	0.27109933
cg09914304	460113	3.98E-11	-6.783496	-7.147865	21.228984	0.30517937
cg04759756	4210619	2.33E-13	-6.7829495	-8.0782155	25.8402162	0.34837508

cg24841244	5270544	1.58E-11	-6.7788622	-7.3186355	25.2321541	0.34298902
cg06625767	4490528	7.78E-11	-6.7511387	-7.0231432	18.9032026	0.2811448
cg25634666	6480692	6.91E-11	-6.7496532	-7.0453833	18.2075825	0.27362988
cg02374486	6180133	2.55E-11	-6.7473744	-7.2303345	19.348141	0.28587056
cg15880738	5270544	1.33E-11	-6.7239002	-7.3501323	25.4033277	0.34451421
cg24474182	2970044	4.53E-11	-6.7231116	-7.1240027	16.9767138	0.25994031
cg21842274	4890743	2.45E-11	-6.7210856	-7.2380586	18.5408113	0.27724932
cg23547429	2370358	1.70E-11	-6.7183314	-7.3056581	21.9163858	0.31197827
cg02473123	5050347	4.75E-11	-6.7101816	-7.1152196	19.6345993	0.28888034
cg10161121	4210619	1.14E-10	-6.6918506	-6.9510692	19.8641413	0.29127385
cg11283860	1450112	8.24E-11	-6.6896104	-7.0123144	16.4939099	0.25442868
cg07728874	4760338	5.89E-13	-6.6839144	-7.9137095	20.9886214	0.30277019
cg24427660	70343	2.49E-11	-6.6501366	-7.2352879	18.1195572	0.27266771
cg07728874	4210619	4.66E-14	-6.6479591	-8.36107	27.4805925	0.36247421
cg01861509	6180088	1.26E-11	-6.6456015	-7.3608976	18.5635735	0.27749525
cg26928972	450202	5.98E-11	-6.6388613	-7.0723092	17.2598027	0.26313428
cg03330678	4490528	1.34E-11	-6.6355111	-7.3488042	20.5226208	0.29805151
cg05615150	6510053	6.04E-13	-6.6316507	-7.909353	23.2419398	0.32472024
cg23889010	110347	8.30E-11	-6.6044709	-7.0109933	16.6368914	0.25606948
cg18920397	6590228	1.55E-12	-6.5842078	-7.7403644	21.0923676	0.3038121
cg20764656	6270022	5.51E-11	-6.5838472	-7.0876729	16.8420086	0.25841074
cg04759756	5050347	4.04E-13	-6.5794812	-7.9810119	24.1756769	0.33341618
cg21969640	540390	1.10E-10	-6.5585366	-6.9584034	19.1902609	0.28420082
cg01500140	3460348	6.22E-12	-6.5303741	-7.4894786	20.043852	0.29313655
cg17813891	6180088	3.03E-11	-6.5197238	-7.1985928	17.7699183	0.26882064
cg01500140	3610719	8.35E-11	-6.5192443	-7.009921	16.9608741	0.25976078
cg19812619	1990079	7.42E-11	-6.5186195	-7.0320818	18.7824874	0.27985186
cg09001777	110347	1.07E-10	-6.4974386	-6.9634381	16.4145092	0.25351438
cg01980222	6270022	1.13E-10	-6.4903442	-6.9534561	16.2128876	0.2511826
cg00795812	3840554	5.63E-11	-6.4865654	-7.0835493	17.9417099	0.27071593
cg20748065	4180544	5.35E-11	-6.4759562	-7.0930705	18.4729129	0.27651476
cg05044994	5090739	2.36E-11	-6.4740296	-7.2447008	18.8924613	0.28102994
cg16008138	3610286	1.15E-10	-6.4678643	-6.9501281	18.8692811	0.28078195
cg07285167	450753	8.33E-11	-6.4584344	-7.0102297	17.2533123	0.26306136
cg23889010	2370358	1.37E-11	-6.4408631	-7.3455318	22.1232676	0.31399851
cg24474182	110347	7.20E-11	-6.436785	-7.037526	16.761624	0.25749497
cg21969640	450202	6.40E-11	-6.425589	-7.0595316	17.1990684	0.26245137
cg02212836	5270544	1.23E-11	-6.4151882	-7.3654964	25.4870927	0.345258
cg09914304	6650053	6.88E-13	-6.4147743	-7.8860683	24.8927979	0.33994419
cg20720686	2030088	5.27E-11	-6.4086426	-7.0959052	17.4531739	0.26530021
cg24777950	6270022	5.84E-11	-6.4053931	-7.0766088	16.7896926	0.257815
cg08458487	6270022	1.23E-11	-6.3868549	-7.3653343	18.1816828	0.27334704
cg18338021	6650053	1.96E-11	-6.3866982	-7.2787477	21.6377878	0.30923883
cg21969640	70343	1.48E-11	-6.386638	-7.3313394	18.5906877	0.27778797

cg01980222	2470017	1.41E-11	-6.3837071	-7.3399432	19.9006716	0.29165328
cg16967583	110347	5.49E-13	-6.3829905	-7.9263168	21.2121432	0.30501111
cg13650156	6270022	4.45E-11	-6.3660389	-7.1272925	17.030019	0.26054384
cg23547429	7160059	1.13E-10	-6.3397475	-6.9525597	16.4347773	0.25374798
cg15958424	110347	4.05E-12	-6.3275248	-7.567218	19.3503455	0.28589382
cg20720686	450202	6.50E-11	-6.3174927	-7.0568073	17.1861335	0.26230576
cg15518883	5270544	9.52E-12	-6.3126097	-7.4116966	25.7400301	0.34749374
cg03330678	70343	3.51E-11	-6.3105231	-7.1715415	17.8103127	0.26926718
cg20366831	1990079	9.94E-12	-6.3098322	-7.40368	20.634014	0.29918526
cg07728874	3840554	4.39E-12	-6.3091921	-7.5528045	20.2739011	0.29550675
cg02600394	5090739	9.95E-14	-6.2927754	-8.2284714	24.0741757	0.33248176
cg15518883	5390730	8.95E-11	-6.2871338	-6.9968979	26.1813483	0.35135825
cg02473123	6180088	7.96E-13	-6.2822442	-7.8600003	21.1150079	0.30403905
cg07728874	5050347	5.14E-13	-6.2753606	-7.9381837	23.9387978	0.33123138
cg04759756	3840554	6.54E-11	-6.2701762	-7.0556768	17.8078893	0.2692404
cg15518883	4760338	5.25E-11	-6.2562517	-7.0967116	16.8941183	0.25900319
cg15958424	2570026	1.06E-10	-6.2496367	-6.9648216	16.9626075	0.25978043
cg15518883	4210619	5.68E-12	-6.2426828	-7.5057466	22.6929953	0.31950117
cg25634666	1450112	4.43E-11	-6.2322493	-7.1282972	17.0414697	0.26067336
cg00795812	4200541	6.24E-11	-6.2295209	-7.0642905	25.9577078	0.34940563
cg25087423	7570324	8.58E-11	-6.2247242	-7.0048028	30.9394659	0.39029107
cg17078393	5270544	9.93E-12	-6.2160263	-7.4039577	25.697551	0.34711933
cg05615150	5090739	7.77E-12	-6.2147407	-7.4486903	19.9127664	0.29177882
cg22534509	5270544	3.04E-11	-6.1987885	-7.1982245	24.5845403	0.33715383
cg22534509	4210619	1.11E-11	-6.1886923	-7.3837602	22.0522444	0.31330629
cg03019000	2510253	4.54E-11	-6.1791932	-7.1236834	21.4137088	0.3070196
cg02473123	6180133	4.85E-11	-6.1746154	-7.1114966	18.763416	0.27964717
cg14654385	1450112	1.06E-10	-6.1705319	-6.9645087	16.270831	0.25185421
cg17078393	4760338	5.46E-11	-6.1604477	-7.0893425	16.8592153	0.25860648
cg25087423	1050050	1.75E-11	-6.1462287	-7.2995689	22.6564404	0.31915076
cg24821554	2570026	1.12E-10	-6.1384245	-6.9545219	16.9142317	0.25923162
cg18084554	70343	1.12E-10	-6.1258998	-6.9555451	16.7828236	0.2577367
cg20981615	1850546	2.45E-11	-6.1174275	-7.2380885	22.7320757	0.31987539
cg07285167	5550279	1.03E-10	-6.0895552	-6.9703765	16.3404287	0.25265932
cg22242539	6270095	1.04E-10	-6.0868087	-6.9681266	16.7549489	0.25741882
cg20720686	70343	7.97E-11	-6.0821321	-7.018608	17.0795646	0.26110393
cg23713742	7160059	1.01E-10	-6.0747305	-6.9747844	16.5384699	0.25494081
cg23668631	2680021	7.48E-11	-6.062211	-7.0305707	21.5745296	0.30861378
cg22534509	5050347	1.24E-11	-6.0416355	-7.3637594	20.8848885	0.3017253
cg15518883	5050347	1.16E-11	-6.0295814	-7.3756259	20.9456569	0.30233779
cg15518883	3840554	9.85E-11	-6.0282193	-6.9788366	17.441698	0.26517202
cg17078393	3840554	5.30E-11	-6.013899	-7.0947736	17.9957487	0.27131008
cg16545105	110719	1.99E-11	-5.9777986	-7.2766764	18.7631301	0.2796441
cg21969640	6270022	1.42E-11	-5.9766888	-7.338486	18.0498956	0.27190445

cg15880738	6180133	1.02E-11	-5.9619812	-7.3993617	20.1965075	0.29471114
cg15958424	7160059	1.56E-12	-5.9308421	-7.7396795	20.3088934	0.29586589
cg11024597	6270022	6.61E-12	-5.923292	-7.4782734	18.7413232	0.2794099
cg24612198	6180088	5.22E-12	-5.9109768	-7.5211156	19.364379	0.28604185
cg08399444	110719	3.02E-11	-5.9043555	-7.1993793	18.3838521	0.27554898
cg20720686	6270022	1.13E-11	-5.9024843	-7.3807936	18.2577843	0.27417747
cg18084554	6270022	3.45E-11	-5.8622935	-7.1745915	17.2558431	0.2630898
cg16173109	5090739	6.00E-11	-5.8372752	-7.0715864	18.0488078	0.27189252
cg03602500	1850546	5.71E-11	-5.8323584	-7.0808521	21.9214933	0.31202829
cg18920397	4760338	2.40E-11	-5.8132564	-7.2420712	17.5900131	0.26682525
cg07285167	110347	2.22E-11	-5.791136	-7.256337	17.8082387	0.26924426
cg15880738	6180088	2.44E-12	-5.7847481	-7.6585762	20.0651814	0.29335698
cg24474182	4890743	8.44E-11	-5.7713147	-7.0079518	17.4302132	0.26504369
cg23090046	7160059	2.78E-11	-5.7686519	-7.2143505	17.6772112	0.26779375
cg24841244	6180088	6.98E-12	-5.7456822	-7.4682199	19.0980881	0.28322239
cg10161121	6180088	9.59E-11	-5.7236783	-6.9838364	16.7469767	0.25732786
cg15361231	110347	2.42E-11	-5.7176519	-7.2399954	17.7289663	0.26836738
cg21019522	7160059	4.14E-11	-5.7173571	-7.1408394	17.3236981	0.26385138
cg04353769	110347	8.15E-11	-5.7149787	-7.0144796	16.6532542	0.25625679
cg24926276	110347	3.32E-11	-5.6772727	-7.1817559	17.4479035	0.26524134
cg21969640	6270095	4.90E-11	-5.6614537	-7.1093116	17.4233099	0.26496654
cg08399444	2970044	5.87E-11	-5.6577291	-7.0757062	16.7479066	0.25733847
cg24949488	6650053	6.99E-11	-5.6546864	-7.043181	20.4454139	0.29726354
cg20981615	5090739	6.22E-15	-5.6415659	-8.7136998	26.8726136	0.35732033
cg15518883	6180133	7.02E-12	-5.6004046	-7.467304	20.5430415	0.29825962
cg07728874	6180133	3.78E-12	-5.5912584	-7.5799355	21.124486	0.30413402
cg11822932	6270022	6.81E-11	-5.588371	-7.0479693	16.6546515	0.25627278
cg02600394	2510253	3.22E-11	-5.5861531	-7.1872666	21.7379304	0.31022604
cg27485921	6270022	3.50E-12	-5.5778161	-7.5938281	19.3227391	0.28560244
cg18920397	5270544	6.32E-11	-5.5768616	-7.0619746	23.8646955	0.33054497
cg09914304	5090739	6.03E-12	-5.5484141	-7.4948817	20.1477386	0.29420887
cg02473123	1850546	5.64E-11	-5.5261323	-7.0832422	21.9336811	0.31214762
cg04759756	6180133	9.35E-11	-5.5030509	-6.9886037	18.1689316	0.27320771
cg01124420	5090739	2.89E-13	-5.4976083	-8.0400644	23.0307879	0.32272222
cg11283860	6270022	8.15E-11	-5.4856058	-7.0143821	16.4969785	0.25446397
cg12836863	6270022	7.04E-11	-5.4654718	-7.0417494	16.6253955	0.25593782
cg16545105	110347	7.80E-11	-5.4601329	-7.0225167	16.6910062	0.25668859
cg24427660	110347	9.49E-12	-5.4550397	-7.412279	18.5736947	0.27760454
cg07285167	2370358	3.80E-11	-5.4015708	-7.1568986	21.1544952	0.30443454
cg16967583	7160059	9.04E-11	-5.3786739	-6.9948973	16.6325953	0.25602028
cg25226014	5090739	2.08E-12	-5.3759137	-7.6877888	21.1447421	0.3043369
cg10126923	2470017	1.11E-10	-5.3734652	-6.9567314	18.0209434	0.27158677
cg20720686	2470017	9.54E-11	-5.3555568	-6.9849258	18.1558082	0.27306426
cg11024597	2470017	9.76E-11	-5.3211081	-6.9805505	18.1348437	0.27283498

cg00071250	5090739	7.67E-12	-5.3040284	-7.4512323	19.9256594	0.29191259
cg16967583	4890743	5.71E-11	-5.2928075	-7.0808152	17.7780028	0.26891005
cg04759756	6180088	5.52E-11	-5.2636042	-7.0872493	17.2356928	0.26286333
cg01500140	3120180	6.69E-11	-5.2612743	-7.0512383	19.5645958	0.28814717
cg20981615	2510253	2.00E-13	-5.251145	-8.1053299	26.7404042	0.35618853
cg19154438	6650053	2.94E-11	-5.2258694	-7.2044816	21.2576395	0.30546547
cg12125117	7160059	1.40E-11	-5.2245237	-7.3409869	18.2946939	0.27457955
cg07285167	7160059	7.90E-11	-5.212498	-7.0202628	16.7516879	0.25738162
cg15361231	7160059	4.92E-11	-5.2031064	-7.1086867	17.1702148	0.26212649
cg07728874	6180088	1.31E-11	-5.1773388	-7.3527709	18.523414	0.27706125
cg21969640	110347	1.13E-11	-5.1721078	-7.379943	18.4136327	0.27587221
cg20748065	70343	7.89E-11	-5.1348019	-7.0203934	17.088005	0.26119926
cg15361231	4890743	2.86E-11	-5.126522	-7.2091067	18.3991034	0.27571455
cg22534509	6180088	5.17E-11	-5.1246923	-7.0993297	17.2932519	0.26350985
cg15518883	6180088	4.47E-11	-5.1229199	-7.1264122	17.4226466	0.26495912
cg02473123	5090739	1.73E-14	-5.1076033	-8.5342554	25.8190324	0.34818892
cg04404982	6510053	1.08E-11	-5.0835744	-7.389104	20.4973167	0.29779345
cg09971811	2370358	8.82E-11	-5.0827322	-6.9994909	20.3653787	0.29644484
cg17078393	6180088	3.38E-11	-5.0755518	-7.1783821	17.672329	0.26773959
cg01861509	5090739	1.96E-11	-5.0659668	-7.2791894	19.0629744	0.28284894
cg22242539	7160059	5.94E-11	-5.0465217	-7.0735153	17.0031146	0.26023935
cg16545105	940039	1.07E-12	-5.0106447	-7.8076902	21.1894406	0.30478417
cg17813891	5090739	3.13E-11	-5.0075659	-7.1925153	18.635993	0.27827655
cg13650156	7160059	7.82E-11	-5.0050652	-7.0221818	16.7607151	0.2574846
cg18920397	6180133	3.09E-11	-4.9893807	-7.1948699	19.1726281	0.28401385
cg20720686	110347	3.57E-11	-4.9791872	-7.1681436	17.3825378	0.2645105
cg15958424	830278	1.14E-11	-4.9772199	-7.3785813	18.7321054	0.27931086
cg02374486	5090739	1.45E-11	-4.9598625	-7.3339485	19.3353705	0.28573579
cg09914304	5820333	3.36E-11	-4.9461649	-7.1792264	18.2502383	0.27409521
cg18084554	110347	1.02E-10	-4.9461229	-6.9724417	16.4564965	0.25399814
cg18638581	830278	3.64E-11	-4.8433104	-7.1644352	17.6848716	0.26787871
cg24821554	830278	5.05E-11	-4.7595133	-7.1037424	17.3936763	0.26463514
cg11024597	7160059	1.64E-11	-4.6323268	-7.3117644	18.1512506	0.27301443
cg10126923	2370358	9.56E-11	-4.6313612	-6.9843811	20.2905532	0.2956777
cg20720686	7160059	2.96E-11	-4.6093156	-7.2032906	17.6237932	0.26720074
cg01500140	2070470	1.05E-10	-4.5989741	-6.9668341	16.4393379	0.25380052
cg26928972	4890743	1.12E-10	-4.5943409	-6.9552058	17.1806919	0.26224449
cg00795812	6650053	1.13E-10	-4.5869317	-6.9528343	19.99851	0.2926675
cg24612198	5090739	3.89E-12	-4.5660164	-7.5746256	20.5568077	0.29839985
cg24612198	1990079	1.16E-10	-4.5245437	-6.9482267	18.3778533	0.27548383
cg10161121	5090739	2.99E-11	-4.5000919	-7.2011009	18.67806	0.27872962
cg07285167	830278	1.07E-10	-4.4992101	-6.9628685	16.7273313	0.2571036
cg24841244	5090739	2.75E-12	-4.4874204	-7.6375352	20.8825754	0.30170196
cg15880738	5090739	2.31E-12	-4.4501844	-7.6691334	21.0472184	0.30335905

cg24841244	1990079	1.04E-10	-4.430388	-6.9690726	18.4779896	0.27656973
cg15518883	5090739	2.32E-13	-4.3070382	-8.0791373	23.2451854	0.32475086
cg07728874	5090739	1.56E-13	-4.2793587	-8.1493935	23.6333038	0.3283925
cg00795812	5090739	4.58E-11	-4.1877541	-7.121839	18.2916067	0.27454594
cg07728874	1990079	2.40E-11	-4.1541288	-7.2417773	19.8155985	0.29076903
cg18920397	6650053	5.03E-11	-4.0096933	-7.1044328	20.7516799	0.30037889
cg17078393	5090739	4.45E-11	-3.8803248	-7.1275136	18.319132	0.27484553
cg22534509	2510253	6.53E-11	-3.8312446	-7.055816	21.0708202	0.30359595
cg18920397	5090739	5.01E-11	-3.5960902	-7.1052164	18.2111034	0.27366831
cg17356733	5820333	7.61E-11	3.58462248	7.02729166	17.5190013	0.26603463
cg09076123	4890255	2.32E-11	3.62508929	7.2479279	24.8554516	0.33960738
cg14654385	5090739	6.89E-11	3.81794994	7.04584689	17.9251118	0.27053324
cg17356733	2510253	3.78E-11	3.86036908	7.15761965	21.5863969	0.30873112
cg13053608	5090739	7.59E-11	3.86537138	7.02765747	17.837971	0.26957261
cg25634666	5090739	2.04E-11	3.87928447	7.27157855	19.0252766	0.28244758
cg27485921	2510253	3.16E-11	3.9197036	7.19061626	21.7550906	0.31039492
cg20748065	6650053	8.69E-11	3.99846167	7.0022694	20.2423281	0.2951824
cg21969640	5820333	6.16E-11	4.00060695	7.06662655	17.7068132	0.26812195
cg24211388	5090739	1.26E-11	4.0325315	7.36038337	19.4675995	0.28712879
cg11283860	2510253	5.58E-11	4.04085571	7.08517845	21.2187661	0.30507729
cg17356733	5090739	9.10E-12	4.05582726	7.41990617	19.7670786	0.29026372
cg27485921	5090739	7.58E-12	4.1175976	7.45335992	19.9364545	0.29202456
cg12836863	2510253	7.98E-12	4.16242219	7.4439775	23.0762397	0.32315331
cg03544320	7510377	4.81E-11	4.26586937	7.11290041	21.8174924	0.31100835
cg02679745	2510253	8.01E-11	4.28076738	7.01769866	20.8796781	0.30167273
cg25634666	6650053	2.78E-11	4.3009278	7.21467157	21.3095685	0.30598335
cg12971694	2510253	2.15E-11	4.31365294	7.26231247	22.1243088	0.31400865
cg14654385	6650053	3.47E-11	4.31457142	7.17370215	21.1012304	0.30390096
cg10126923	2510253	1.59E-11	4.3161402	7.31795701	22.4133895	0.3168117
cg20070090	5090739	8.18E-11	4.33782715	7.01376271	17.7715567	0.26883876
cg18084554	6420424	6.24E-11	4.34637808	7.06425924	31.908721	0.39765583
cg02679745	5090739	9.15E-11	4.36889946	6.99263975	17.6708445	0.26772312
cg24777950	5820333	8.94E-11	4.37144271	6.99702442	17.3751984	0.26442835
cg11283860	5090739	2.38E-12	4.37794466	7.66341178	21.0173554	0.30305907
cg18084554	5090739	3.74E-11	4.37924559	7.15939292	18.4741731	0.2765284
cg22242539	5820333	4.33E-11	4.39616588	7.1325704	18.0240264	0.27162061
cg17356733	6650053	3.20E-11	4.41901592	7.188264	21.1751446	0.30464118
cg22566906	4860762	2.13E-11	4.42439119	7.26358488	24.5594818	0.33692596
cg03330678	5090739	2.14E-11	4.4564219	7.26255284	18.9806215	0.28197157
cg26928972	5090739	7.30E-11	4.47246265	7.03508037	17.8735051	0.26996464
cg16545105	5820333	2.01E-11	4.47529416	7.27409468	18.7147555	0.27912437
cg12836863	5090739	3.94E-13	4.47691685	7.98534479	22.7322825	0.31987737
cg24926276	5820333	5.72E-11	4.48628449	7.08045294	17.7730789	0.2688556
cg16967583	1340750	1.03E-10	4.49949846	6.97099809	17.6347031	0.26732193

cg04353769	5560010	3.86E-11	4.50202001	7.15395888	17.8467399	0.26966939
cg12163490	7510377	3.86E-11	4.50505456	7.15399227	22.0277559	0.3130673
cg12971694	1990079	8.53E-11	4.53765828	7.00577094	18.6550045	0.27848138
cg22030890	7510377	3.57E-11	4.53824633	7.16843962	22.1019695	0.31379108
cg24898863	2510253	8.90E-11	4.57434199	6.99799122	20.7812598	0.30067832
cg00899659	5090739	2.05E-11	4.5801348	7.27063042	19.0205831	0.28239758
cg12971694	6840711	4.67E-11	4.5994014	7.11829638	19.1697953	0.28398381
cg10126923	5090739	1.40E-12	4.60624937	7.75844521	21.5162553	0.30803697
cg15958424	5560010	1.15E-10	4.61457983	6.94936317	16.8733375	0.25876705
cg22566906	6420424	1.90E-11	4.63325385	7.28497514	33.2126206	0.40728717
cg08368934	5090739	1.02E-10	4.64889217	6.97208309	17.5731241	0.26663737
cg04353769	5820333	2.51E-11	4.66895548	7.23347426	18.5151132	0.27697148
cg10126923	6840711	1.01E-11	4.7093036	7.40157714	20.588785	0.29872537
cg16967583	5820333	4.87E-11	4.71389084	7.11051142	17.917587	0.27045039
cg08399444	5090739	7.49E-11	4.72262814	7.03024032	17.8503311	0.26970902
cg27485921	7330097	9.43E-11	4.74451484	6.98701981	23.1584465	0.3239316
cg12125117	2510253	2.29E-11	4.76156288	7.25003492	22.0608224	0.31338997
cg10126923	6420424	1.87E-13	4.7649115	8.11680772	38.4872111	0.44329613
cg21969640	5090739	3.13E-13	4.7658099	8.02628602	22.9554324	0.32200631
cg11283860	6650053	9.00E-12	4.77088576	7.42193061	22.381707	0.31650561
cg12971694	5090739	1.99E-13	4.77336238	8.10674711	23.3973103	0.32618291
cg20748065	6180088	2.46E-11	4.78105291	7.23707863	17.9565101	0.27087875
cg16545105	2510253	1.27E-11	4.794723	7.35901856	22.6281245	0.31887908
cg24091474	2510253	2.98E-12	4.79781424	7.62277025	24.0360831	0.3321304
cg15958424	5820333	6.18E-11	4.80628937	7.06607466	17.7041708	0.26809267
cg02240622	6650053	2.76E-11	4.80858044	7.21588578	21.3157611	0.30604506
cg12380764	2510253	3.96E-11	4.81356241	7.14879592	21.5414175	0.30828614
cg27461196	6420424	9.38E-11	4.81764048	6.98810381	31.4681336	0.39433027
cg01980222	2510253	5.07E-11	4.82126458	7.10298912	21.3088065	0.30597576
cg12971694	6650053	6.28E-11	4.82230576	7.0632125	20.5452818	0.29828245
cg15361750	6510053	9.94E-11	4.82548325	6.97717336	18.4565558	0.27633757
cg14324675	5090739	1.53E-12	4.82789086	7.74261118	21.432704	0.30720828
cg08458487	5090739	6.26E-12	4.83616087	7.48812309	20.1132672	0.29385341
cg22381196	5090739	4.73E-11	4.84093107	7.11584231	18.2625429	0.27422934
cg10126923	1340075	9.70E-11	4.85119532	6.98169253	17.0385026	0.26063981
cg24898863	5090739	1.23E-11	4.8608818	7.36496061	19.4905435	0.28736994
cg08519905	6840711	1.08E-10	4.86113739	6.96163072	18.4088147	0.27581993
cg07285167	6420424	1.09E-10	4.88622181	6.96037961	31.3089245	0.3931195
cg13650156	6420424	8.64E-12	4.92174593	7.42932329	34.0870659	0.4135756
cg07684796	2070470	1.64E-11	4.92198524	7.31145253	18.0861456	0.27230183
cg01718139	6650053	1.99E-11	4.92293291	7.27606552	21.6239907	0.3091026
cg16483916	2070470	1.11E-10	4.92423536	6.95675652	16.392377	0.25325913
cg06812844	5090739	3.41E-12	4.9278342	7.59866437	20.6809706	0.29966209
cg27461196	5090739	2.37E-11	4.9359701	7.24392202	18.8886202	0.28098886

cg09971811	5090739	4.54E-12	4.94841702	7.54652588	20.412168	0.29692369
cg24474182	5560010	1.13E-10	4.95245909	6.95375206	16.8939221	0.25900097
cg10644361	2070470	8.82E-13	4.9617812	7.84171269	20.7753305	0.30061832
cg08044694	5090739	2.87E-13	4.96668767	8.04163306	23.0393755	0.32280371
cg12125117	5090739	8.02E-12	4.96678731	7.4430263	19.8840542	0.29148073
cg21126943	6420424	1.45E-11	4.9668067	7.33410389	33.5083113	0.40942862
cg03330678	6650053	2.06E-11	4.97320293	7.27017075	21.5936856	0.30880318
cg24926276	5090739	2.02E-11	4.97496126	7.27375259	19.036041	0.28256224
cg18084554	6650053	1.34E-11	4.97794417	7.3491192	22.0015982	0.31281182
cg02712878	2510253	8.91E-11	4.97931315	6.99766637	20.7796399	0.30066193
cg24777950	5090739	7.09E-12	4.98405228	7.46542444	19.9977239	0.29265937
cg12125117	6420424	6.44E-12	4.99793669	7.48297965	34.4164842	0.41591009
cg04353769	1940288	3.53E-11	5.0001141	7.17043011	19.1934825	0.28423497
cg08519905	5090739	9.76E-13	5.00404977	7.82356665	21.8616756	0.31144202
cg12125117	6840711	8.95E-11	5.01857819	6.9968221	18.578277	0.27765401
cg10266490	6840711	4.67E-11	5.02752199	7.11853371	19.1709609	0.28399617
cg27654142	7510377	6.02E-11	5.03016271	7.07113505	21.6050238	0.30891523
cg08399444	6840711	4.77E-11	5.03839151	7.11457667	19.1515311	0.28379002
cg15361750	6840711	5.98E-11	5.04095795	7.07219581	18.9441101	0.2815819
cg19399532	5090739	8.05E-11	5.04096565	7.0166627	17.7854072	0.26899192
cg04434339	7510377	2.60E-11	5.06555344	7.2271068	22.4048715	0.31672943
cg16545105	6840711	4.40E-11	5.06902564	7.12942632	19.2245016	0.28456361
cg09303642	5090739	4.37E-11	5.07161332	7.13079489	18.335058	0.27501876
cg24091474	5090739	3.73E-13	5.07741526	7.99527718	22.7863141	0.32039408
cg10126923	2810400	7.99E-11	5.08745757	7.01809947	25.2277948	0.34295008
cg04353769	6510053	6.58E-11	5.09460771	7.05448822	18.8306629	0.28036841
cg12836863	1770168	1.34E-11	5.09736833	7.34925179	28.8360187	0.3736719
cg13615963	5090739	1.04E-10	5.09867913	6.96778466	17.552727	0.26641033
cg07285167	6510053	3.03E-11	5.11046409	7.19843347	19.5381606	0.28786991
cg24427660	6650053	5.43E-11	5.11151435	7.09041148	20.6813378	0.29966582
cg14654385	6180088	1.36E-11	5.12317438	7.34659038	18.4929014	0.27673116
cg25634666	6180088	8.28E-12	5.13106577	7.43719961	18.9427977	0.28156788
cg08458487	6650053	8.10E-11	5.14088215	7.01557025	20.3082239	0.29585902
cg17105014	5090739	2.52E-11	5.15018713	7.23292878	18.834445	0.28040893
cg27634151	7510377	3.04E-11	5.1659655	7.19800508	22.2543091	0.31527203
cg25957124	1990079	4.91E-12	5.1759414	7.53230351	21.2971011	0.30585909
cg09868035	5090739	5.88E-11	5.18073879	7.07552861	18.0677929	0.2721007
cg06713098	7510377	3.02E-11	5.18212404	7.19919673	22.2604624	0.31533171
cg12380764	5090739	2.34E-12	5.18225696	7.66669798	21.0345045	0.30323137
cg12836863	1850546	1.70E-11	5.18435975	7.3051353	23.0831281	0.32321859
cg10644361	7510377	7.91E-13	5.18665516	7.86112533	25.835858	0.34833679
cg09076123	5090739	1.20E-12	5.19302912	7.78601308	21.6621297	0.30947905
cg15880738	3420154	2.13E-11	5.19954468	7.26349502	20.8062489	0.30093108
cg06812844	6650053	5.18E-11	5.22931746	7.09902436	20.7245305	0.30010385

cg26112639	5090739	1.15E-10	5.23302222	6.94912747	17.4643398	0.26542489
cg12089698	5090739	1.05E-11	5.24503861	7.39334476	19.6331412	0.28886508
cg27485921	6180088	2.26E-11	5.25213206	7.25254199	18.0317616	0.2717055
cg14654385	6180133	5.20E-11	5.27736301	7.09833937	18.6992739	0.27895788
cg26701826	5820333	3.36E-11	5.28134098	7.17932934	18.2507388	0.27410067
cg05037688	5560010	4.57E-11	5.29889557	7.12234314	17.6944772	0.26798522
cg24211388	6180088	7.03E-12	5.30203649	7.46708769	19.0924086	0.28316201
cg02266731	5820333	1.72E-12	5.31166856	7.72197769	20.9901431	0.30278549
cg24777950	6650053	7.92E-11	5.31205949	7.01972277	20.3288222	0.29607026
cg02266731	5560010	2.58E-13	5.32077129	8.06000091	22.4968694	0.3176169
cg16692277	2510253	3.18E-12	5.32722886	7.61097748	23.9720723	0.33153914
cg08840010	2510253	2.66E-11	5.33322256	7.22304308	21.9216272	0.3120296
cg09303642	6420424	3.00E-12	5.33604386	7.62191433	35.280477	0.42194557
cg15958424	1940288	1.10E-11	5.35185641	7.38585023	20.2715307	0.29548241
cg04988978	5090739	2.24E-11	5.35417924	7.25419267	18.9393087	0.28153062
cg08399444	6650053	3.07E-11	5.35811355	7.19620152	21.2154975	0.30504463
cg12949760	6420424	6.23E-11	5.36152131	7.06465937	31.9110485	0.3976733
cg22242539	5090739	3.75E-14	5.36470638	8.39895026	25.0391059	0.34126037
cg24926276	6650053	1.09E-10	5.36946694	6.96039507	20.0356886	0.29305215
cg17105014	1990079	4.76E-11	5.37138865	7.11490147	19.1868901	0.28416509
cg07285167	5090739	4.03E-13	5.38151007	7.98149109	22.7113367	0.31967686
cg04988978	5220767	8.38E-11	5.38872319	7.00914742	23.8822843	0.33070803
cg15361750	5090739	3.44E-14	5.39192087	8.41474285	25.1294952	0.34207089
cg08368934	6650053	1.23E-11	5.39832583	7.3652625	22.0855503	0.31363108
cg17356733	6180088	2.34E-12	5.40257994	7.66629583	20.1049139	0.29376722
cg07730301	5090739	2.56E-11	5.40613679	7.22964261	18.8182666	0.28023557
cg16632715	7510377	2.81E-11	5.41613651	7.21280057	22.33078	0.31601302
cg10057295	5090739	1.65E-12	5.44686894	7.72924716	21.362319	0.30650863
cg17356733	6180133	3.16E-11	5.44889756	7.19071805	19.1521371	0.28379645
cg09076077	5090739	5.18E-11	5.46373543	7.09914395	18.1817411	0.27334768
cg12836863	6420392	7.14E-11	5.47178032	7.03922095	27.9820496	0.36666329
cg25087423	2140524	3.66E-12	5.47486727	7.58573974	19.5918176	0.28843245
cg20748065	4210619	7.60E-11	5.4887153	7.02737477	20.2404224	0.29516281
cg08840010	5090739	1.74E-11	5.50089072	7.30130806	19.1727568	0.28401522
cg20720686	6180088	1.09E-10	5.5023237	6.95979823	16.6344045	0.256041
cg24354652	6650053	3.19E-11	5.5034002	7.18931242	21.180472	0.30469447
cg13765621	5560010	7.00E-11	5.50468402	7.0428991	17.3148478	0.26375213
cg09305224	5090739	1.97E-11	5.50987437	7.27792418	19.0567051	0.28278223
cg09971811	510452	9.15E-11	5.52334321	6.99260662	18.0395629	0.27179111
cg27485921	6180133	2.77E-11	5.52758385	7.21496043	19.2719492	0.28506573
cg01980222	5090739	5.15E-14	5.53097828	8.34438621	24.7281141	0.3384564
cg04451770	5090739	8.16E-12	5.53239862	7.43974729	19.867442	0.29130815
cg17749456	6420424	1.11E-10	5.54341385	6.95671923	31.2879517	0.39295964
cg02266731	1940288	1.09E-11	5.54601838	7.38728464	20.278816	0.29555722

cg10126923	1850546	5.81E-12	5.54881342	7.50162664	24.1305865	0.3330014
cg25028542	5820333	6.33E-12	5.5525304	7.48616762	19.7752113	0.29034846
cg01718139	6180088	1.11E-10	5.55573305	6.95563228	16.6149345	0.25581798
cg21991396	5090739	2.05E-12	5.56524452	7.69078079	21.1604049	0.30449369
cg22242539	6650053	3.47E-12	5.57879278	7.59546028	23.3027192	0.32529318
cg18463686	6420424	6.77E-11	5.58546797	7.0490432	31.8203088	0.39699143
cg26701826	1940288	8.64E-11	5.58858457	7.00335429	18.3793392	0.27549997
cg07525077	1340750	6.21E-11	5.59128745	7.06520014	18.08526	0.27229213
cg18638581	5090739	7.92E-13	5.61150415	7.86103239	22.0617115	0.31339864
cg04404982	110347	3.23E-11	5.61179846	7.18706268	17.4734199	0.26552624
cg20125091	5670706	2.03E-11	5.62125683	7.27294778	19.8484509	0.29111076
cg24821554	5090739	3.43E-13	5.62236743	8.01001246	22.8665969	0.32116038
cg20125091	6420424	1.70E-11	5.62450489	7.30518799	33.3340348	0.40816835
cg27461196	6650053	5.58E-12	5.64986086	7.50897646	22.841044	0.32091667
cg26701826	5090739	7.29E-11	5.65573885	7.03532653	17.8746841	0.26997764
cg17105014	6650053	5.39E-11	5.65996361	7.09171041	20.6878484	0.29973188
cg17078393	4490528	8.04E-11	5.66191966	7.01703139	18.873512	0.28082723
cg24474182	5090739	3.53E-11	5.66592312	7.17016302	18.5267086	0.27709687
cg12836863	6180133	4.48E-11	5.6712271	7.12622991	18.8353831	0.28041898
cg23090046	5090739	2.23E-12	5.67836572	7.67557549	21.0808685	0.30369676
cg12836863	6180088	1.65E-12	5.69360095	7.72938104	20.4311113	0.29711738
cg12640109	5090739	1.02E-10	5.69448653	6.97221096	17.5737311	0.26664412
cg16967583	6650053	5.11E-11	5.70894994	7.10145011	20.7367048	0.30022721
cg16545105	290603	2.80E-11	5.70908572	7.21345322	18.6087744	0.2779831
cg11283860	6180088	2.03E-12	5.71165292	7.69223928	20.2387372	0.29514549
cg12125117	1770168	9.00E-11	5.73191134	6.99572947	26.869566	0.35729428
cg21019522	5090739	1.13E-12	5.7342854	7.79771214	21.7241912	0.31009076
cg09868035	6650053	7.47E-11	5.74985808	7.03079858	20.3838224	0.29663368
cg06196379	5090739	1.19E-13	5.75165377	8.19768609	23.9020368	0.33089104
cg16692277	5090739	9.48E-14	5.76058108	8.23672771	24.1204517	0.3329081
cg11254522	2510253	2.86E-11	5.76377176	7.20952116	21.8520907	0.31134799
cg18084554	6420392	8.51E-11	5.76805558	7.00628535	27.800589	0.36515377
cg11283860	6180133	2.58E-11	5.76894564	7.22838353	19.3384631	0.28576843
cg12125117	6650053	6.76E-13	5.77278853	7.88918488	24.9101738	0.34010078
cg23713742	6420424	6.62E-11	5.77923014	7.05336572	31.8454052	0.39718017
cg04353769	5090739	6.00E-15	5.78019532	8.71775768	26.8966921	0.35752603
cg12535715	4210619	8.86E-11	5.7959008	6.99877615	20.0989144	0.29370531
cg26215727	5090739	5.04E-11	5.81790931	7.10403245	18.2053765	0.27360579
cg18338021	6510195	3.36E-11	5.81889391	7.17927848	17.9298371	0.27058526
cg15958424	5090739	7.17E-14	5.85222719	8.28582811	24.3966129	0.3354411
cg10126923	7380689	8.60E-11	5.8529822	7.00432343	31.4505145	0.39419651
cg01623438	5090739	1.13E-11	5.86554071	7.37978659	19.5649586	0.28815097
cg18638581	6650053	3.20E-11	5.86755899	7.18864696	21.1770904	0.30466064
cg16967583	5090739	9.55E-15	5.87388614	8.63661477	26.4173342	0.353406

cg17749456	5090739	3.90E-12	5.886036	7.57411489	20.554174	0.29837303
cg02679745	6180088	1.46E-11	5.88772329	7.33289812	18.4253957	0.2759998
cg10126923	6180088	3.64E-12	5.89881447	7.58669501	19.6971332	0.289534
cg06394229	1940288	3.15E-11	5.90226186	7.19124554	19.2962586	0.28532271
cg07285167	6650053	1.11E-12	5.90514279	7.79997082	24.4154873	0.33561351
cg04353769	290603	4.97E-11	5.9152895	7.10693649	18.0905343	0.27234991
cg26233914	5560010	1.47E-11	5.91728643	7.33208805	18.717221	0.27915088
cg14700707	5090739	6.10E-11	5.92372718	7.06861111	18.0344866	0.27173541
cg10549973	7650167	1.14E-10	5.92469185	6.95166402	16.1463703	0.25041012
cg02626929	5090739	9.30E-12	5.94768906	7.41586767	19.7466833	0.29005109
cg04451770	6650053	5.68E-11	5.95019986	7.081984	20.6391255	0.2992372
cg18084554	6180088	3.36E-12	5.95172822	7.601293	19.7715973	0.29031081
cg25634666	4210619	1.53E-11	5.95378591	7.32461375	21.7453489	0.31029906
cg09971811	1770168	5.31E-12	5.96102215	7.51823124	29.810196	0.38148003
cg23889010	5090739	1.02E-11	5.96525409	7.39844349	19.6588146	0.2891336
cg19906550	5090739	3.02E-13	5.97095581	8.03254332	22.9896383	0.32233147
cg17749456	6840711	3.59E-11	5.97912361	7.16732423	19.41142	0.28653761
cg09076123	1770168	1.79E-11	5.98649423	7.29615545	28.5344872	0.37121499
cg09001777	5090739	4.07E-12	5.99490471	7.56650764	20.5149663	0.29797346
cg09419900	2510253	9.14E-11	5.99633216	6.99299086	20.7563323	0.30042601
cg15361750	1770168	6.23E-12	6.00480523	7.48904109	29.6403307	0.38013259
cg25634666	5270544	2.78E-11	6.00877296	7.21491709	24.6736777	0.33796313
cg09971811	1850546	1.10E-11	6.0111604	7.3848883	23.5049244	0.3271923
cg04653308	5090739	3.57E-11	6.01459072	7.16846575	18.5184243	0.27700729
cg20720686	6180133	1.86E-11	6.01510384	7.28833095	19.6370224	0.28890569
cg23547429	6420424	6.74E-11	6.04356535	7.04986814	31.8250972	0.39702745
cg24091474	6180088	6.18E-11	6.04479612	7.06620788	17.1356716	0.26173716
cg23839680	5090739	1.04E-10	6.0492432	6.96921751	17.5595249	0.26648601
cg23889010	6510053	1.54E-11	6.05436224	7.32326665	20.163292	0.29436913
cg14324675	6180088	1.29E-11	6.05506586	7.35600917	18.5394111	0.27723419
cg24211388	4210619	2.78E-11	6.06671183	7.2146319	21.1812434	0.30470218
cg14435807	5090739	3.38E-13	6.0681925	8.01238027	22.8795112	0.32128349
cg09303642	6650053	7.64E-13	6.07061581	7.86722971	24.7879118	0.3389974
cg08965235	5270674	5.53E-11	6.07320139	7.08682001	17.694197	0.26798211
cg21184174	7510377	8.51E-11	6.07416847	7.00623115	21.277328	0.30566191
cg12971694	6180088	7.27E-13	6.08775391	7.87618766	21.2005596	0.30489534
cg13650156	6180088	1.05E-10	6.09290672	6.96635467	16.6650702	0.25639199
cg01980222	1850546	4.71E-11	6.0964964	7.11676098	22.1050414	0.313821
cg02266731	5090739	4.91E-14	6.09675307	8.35266561	24.7751727	0.33888222
cg13765621	1940288	7.00E-11	6.10334459	7.04291583	18.5703842	0.27756879
cg22534509	360475	9.50E-11	6.11852444	6.98561256	16.3885767	0.25321528
cg04353769	1010735	2.95E-11	6.12080441	7.20366544	18.7486758	0.27948888
cg13765621	5090739	9.01E-11	6.12485267	6.99563793	17.685121	0.26788148
cg12971694	6180133	1.28E-11	6.12582021	7.35716236	19.9828681	0.29250555

cg01402255	6650053	7.74E-11	6.12627268	7.02406598	20.3503793	0.2962912
cg23889010	6840711	4.89E-11	6.1263103	7.10967503	19.127478	0.28353466
cg24427660	6420392	5.34E-11	6.13721585	7.09332586	28.2819911	0.36914274
cg09914304	110347	5.28E-11	6.1402096	7.09548931	17.0357538	0.26060872
cg26928972	6180088	3.65E-12	6.14916483	7.58613349	19.6942717	0.28950412
cg23713742	5090739	1.85E-12	6.15039741	7.7089791	21.2558042	0.30544716
cg10126923	1770168	8.88E-16	6.16040817	9.02394547	39.4685226	0.44951809
cg01526089	6650053	1.05E-10	6.16394681	6.96607144	20.0636275	0.29334092
cg11254522	6420424	2.72E-12	6.16573028	7.63948916	35.3909021	0.42270798
cg14654385	4210619	2.90E-12	6.17601263	7.62784977	23.3448736	0.32568998
cg24211388	4760338	1.01E-10	6.17636457	6.9734886	16.3152505	0.25236826
cg24211388	5270544	2.95E-11	6.18400922	7.20383697	24.6144876	0.33742595
cg09076123	1850546	9.18E-12	6.18989831	7.41837343	23.6833852	0.32885954
cg08368934	6180088	3.12E-11	6.19103064	7.19334656	17.7445602	0.26854004
cg21842274	2230528	3.94E-11	6.19359644	7.14987849	17.1238887	0.26160427
cg21126943	6180088	1.13E-10	6.19801731	6.95348781	16.6049167	0.25570318
cg00899659	6180088	2.14E-12	6.20146533	7.68273125	20.1896397	0.29464045
cg06196379	1770168	8.52E-11	6.20839661	7.00602083	26.9254415	0.35777146
cg17356733	4210619	7.81E-12	6.20861701	7.44784997	22.3875761	0.31656233
cg17740645	6650053	1.82E-11	6.21329092	7.29313973	21.7119078	0.30996978
cg25028542	5090739	1.06E-12	6.2208825	7.80957973	21.787242	0.31071111
cg24427660	6180088	5.25E-12	6.22653383	7.52009494	19.3592231	0.28598747
cg22016649	5820333	8.95E-12	6.22936487	7.42303241	19.4563226	0.2870102
cg06812844	6180088	1.60E-11	6.23878564	7.31628286	18.3436482	0.27511216
cg26928972	6180133	3.04E-11	6.25332101	7.19811148	19.1886346	0.28418358
cg08399444	6180088	2.85E-11	6.2607795	7.20972199	17.8237742	0.26941586
cg22045288	5820333	3.42E-11	6.26597936	7.17601734	18.2346317	0.27392503
cg16545105	6180088	3.45E-11	6.26666513	7.17481337	17.6551256	0.26754869
cg20748065	6590228	3.88E-11	6.26849517	7.1528596	18.1278751	0.27275873
cg04988978	1770168	1.08E-10	6.27222832	6.9608047	26.680559	0.35567491
cg06394229	5090739	1.64E-12	6.27242059	7.73069484	21.3699377	0.30658443
cg17173423	6180088	1.94E-11	6.27867612	7.28084596	18.1699172	0.27321848
cg16361890	5090739	4.83E-12	6.27907098	7.53519616	20.3540018	0.29632831
cg04988978	510452	3.15E-11	6.27968547	7.19173434	19.0064184	0.28224664
cg20070090	6180088	1.63E-13	6.28154746	8.14219675	22.6316575	0.31891299
cg15910079	5090739	1.07E-11	6.29419508	7.39060765	19.6193665	0.28872093
cg20070090	6180133	4.31E-12	6.29702135	7.55578855	20.9990976	0.30287554
cg27485921	4760338	6.72E-11	6.3009119	7.05044683	16.6755905	0.25651233
cg24777950	6180088	3.37E-11	6.30257082	7.1791762	17.6761583	0.26778207
cg08519905	6180088	6.68E-12	6.30380776	7.47627252	19.1385057	0.28365176
cg08044694	6180133	3.08E-11	6.30625022	7.19588195	19.1776246	0.28406684
cg09305224	6650053	4.36E-12	6.31220755	7.55399076	23.0806834	0.32319542
cg04600618	1990079	5.90E-12	6.31297476	7.498963	21.1241257	0.30413041
cg01129847	6420424	1.79E-11	6.32223556	7.29572743	33.2771654	0.40775594

cg09001777	6650053	7.82E-11	6.3295316	7.02215715	20.3409033	0.29619409
cg18463686	6650053	2.40E-11	6.33876658	7.24177763	21.4480606	0.30736074
cg10266490	6180088	1.20E-11	6.34524202	7.36880142	18.6026742	0.2779173
cg02039171	6650053	2.33E-11	6.3459465	7.24732473	21.4764663	0.30764257
cg22381196	6180088	3.05E-11	6.35232933	7.19772437	17.7657196	0.26877419
cg09971811	6180088	9.54E-12	6.35526996	7.41135394	18.8139056	0.28018882
cg15958424	290603	1.21E-11	6.36148862	7.3671624	19.3702116	0.28610336
cg15958424	70707	2.38E-11	6.36157501	7.24327386	17.5402543	0.26627143
cg21969640	6180133	1.89E-12	6.36654188	7.70534974	21.7821622	0.31066118
cg11283860	3840554	9.21E-11	6.36720876	6.99155473	17.5020308	0.26584543
cg09671611	5090739	1.02E-11	6.3726063	7.39903362	19.6617872	0.28916468
cg22082462	510452	7.07E-12	6.3732219	7.46602249	20.3826619	0.2966218
cg10787197	2510253	5.35E-11	6.37531885	7.0929991	21.2582749	0.30547182
cg10057295	1770168	1.00E-11	6.37811435	7.40177052	29.1364208	0.3761006
cg27461196	6180088	3.34E-11	6.38115068	7.18061272	17.6830864	0.26785891
cg21969640	6180088	4.35E-14	6.38298096	8.37314744	23.9126927	0.33098973
cg12125117	6420392	6.33E-11	6.39604639	7.0615548	28.1055835	0.36768684
cg23181133	5090739	3.66E-12	6.40321018	7.5854518	20.6126775	0.29896838
cg13650156	6420392	3.20E-11	6.41565069	7.18872682	28.8164605	0.37351312
cg23181133	6420424	3.54E-12	6.42221129	7.59158984	35.0905423	0.42062949
cg17173423	6180133	1.03E-10	6.42270607	6.97060548	18.0827365	0.27226448
cg06196379	1850546	4.44E-11	6.42407021	7.12794577	22.1624022	0.31437933
cg24453664	4210619	2.79E-11	6.43365453	7.21413134	21.1786955	0.3046767
cg02266731	290603	4.32E-11	6.43690581	7.13297892	18.2165275	0.27372751
cg17749456	6650053	1.17E-11	6.43744043	7.37328044	22.1273155	0.31403792
cg09208010	4210619	8.11E-11	6.43959055	7.01522165	20.1802174	0.29454345
cg12125117	6180088	9.76E-12	6.43996195	7.40705263	18.7924986	0.27995927
cg04353769	770411	8.38E-11	6.44338587	7.00925548	20.6288214	0.2991325
cg27485921	5270544	5.39E-12	6.45302325	7.5152467	26.3126922	0.35249957
cg08399444	6180133	9.30E-11	6.46389504	6.98962563	18.1738323	0.27326127
cg15958424	1850546	5.08E-11	6.47181115	7.10252805	22.0321787	0.31311047
cg13471990	5090739	5.89E-12	6.47417124	7.4992464	20.1700164	0.2944384
cg00899659	6180133	4.19E-12	6.47758588	7.56132906	21.0278326	0.30316435
cg25028542	6650053	4.66E-11	6.48781914	7.11878127	20.8238075	0.30110857
cg08458487	6180088	9.25E-13	6.49780151	7.83328157	20.9741813	0.30262492
cg22016649	6420424	8.32E-11	6.50852158	7.01059889	31.597779	0.39531264
cg23090046	6650053	3.75E-13	6.51555648	7.99421874	25.4997909	0.34537061
cg15361750	6180088	3.45E-12	6.53492305	7.59631783	19.746203	0.29004609
cg08840010	1770168	4.05E-11	6.5393109	7.14463583	27.6860366	0.36419713
cg24898863	6180088	1.78E-12	6.53978292	7.71571626	20.3602274	0.29639208
cg23547429	5090739	5.74E-13	6.5565126	7.9182512	22.3690567	0.31638332
cg10126923	7570324	5.75E-13	6.55782409	7.91804981	36.5063483	0.43029804
cg20125091	1770168	9.41E-11	6.55836681	6.98752298	26.8250688	0.35691377
cg16545105	6180133	5.39E-11	6.56363467	7.09161296	18.666528	0.27860547

cg11283860	4210619	6.25E-12	6.57373827	7.48832578	22.6008474	0.31861716
cg15322932	4860762	1.12E-11	6.57738511	7.38236438	25.2007003	0.34270798
cg20125091	510452	2.43E-11	6.57928789	7.23944693	19.2421163	0.2847501
cg05037688	5090739	8.37E-14	6.58340861	8.25882521	24.2445342	0.33404859
cg16545105	6420392	1.09E-11	6.58582236	7.3877291	29.9543221	0.38261871
cg05868799	6180088	7.55E-11	6.59923934	7.02860707	16.9576754	0.25972451
cg23713742	6650053	1.60E-11	6.6049941	7.31627893	21.8313832	0.31114475
cg11051843	3990403	7.78E-11	6.60700289	7.02305546	17.428437	0.26502384
cg09303642	6180088	3.96E-11	6.61163252	7.14924573	17.5321239	0.26618086
cg07525077	5090739	3.57E-12	6.61168505	7.59032141	20.6378337	0.29922408
cg11283860	5270544	1.34E-11	6.61768233	7.34924386	25.3984892	0.3444712
cg24926276	6180088	5.89E-12	6.6183981	7.49916257	19.2536335	0.28487199
cg01980222	6180088	9.51E-12	6.62320899	7.4119218	18.8167327	0.28021912
cg10978355	4210619	6.12E-12	6.64202367	7.49235394	22.6221354	0.31882159
cg08044694	6180088	4.29E-14	6.64484095	8.37615111	23.9295895	0.33114616
cg09106999	5090739	6.53E-11	6.64672869	7.05582137	17.9729926	0.27106
cg17166812	5090739	3.91E-12	6.65694319	7.57379757	20.5525378	0.29835636
cg20720686	5270544	6.38E-11	6.6586118	7.06009053	23.8548381	0.33045356
cg08458487	6180133	5.56E-12	6.66186112	7.5098084	20.7614418	0.30047774
cg15958424	7330523	3.13E-11	6.6663063	7.19242974	30.915538	0.39010698
cg14654385	6590228	3.19E-11	6.66841042	7.18900447	18.3035066	0.27467549
cg22242539	6180133	1.94E-11	6.66963685	7.28056018	19.5981821	0.28849912
cg17105014	6180088	3.21E-11	6.67064996	7.18810829	17.7192588	0.26825986
cg08525145	5090739	5.37E-14	6.67181861	8.33668812	24.6844014	0.33806036
cg20125091	2810400	1.60E-11	6.67313323	7.31600341	26.8448536	0.35708301
cg12380764	6180088	4.21E-12	6.67605533	7.56041578	19.5634439	0.28813509
cg12380764	6180133	7.67E-11	6.68452625	7.02582086	18.3478727	0.27515808
cg26233914	5090739	7.94E-12	6.69090346	7.44487233	19.8934098	0.29157789
cg12836863	3990224	9.56E-11	6.70086926	6.98452894	22.9547791	0.3220001
cg20070090	3840554	1.12E-10	6.70363351	6.95503391	17.3290768	0.26391168
cg16232126	5270674	2.48E-12	6.70950379	7.65606586	20.5326154	0.29815338
cg01718139	4210619	2.48E-11	6.71205173	7.23539548	21.2870879	0.30575925
cg10126923	4200541	6.30E-12	6.7203441	7.48693709	28.3018951	0.36930659
cg14870461	4860762	4.45E-11	6.74079912	7.12751461	23.8377028	0.33029459
cg10287137	4210619	1.11E-11	6.7414616	7.38275379	22.0470019	0.31325514
cg02497700	5270674	2.49E-12	6.74561805	7.65552618	20.5298205	0.2981249
cg03574571	6180088	3.62E-11	6.75442408	7.16552118	17.6103716	0.2670516
cg09971811	7570324	2.11E-11	6.75631588	7.26554712	32.4593885	0.40176129
cg10126923	7150017	7.19E-13	6.76049427	7.87818131	33.1358054	0.40672831
cg24777950	6180133	1.46E-11	6.76284351	7.33298429	19.8610142	0.29124136
cg20340242	6180088	1.67E-11	6.76427859	7.30892431	18.3075032	0.27471899
cg25634666	6590228	9.68E-12	6.76677596	7.4086196	19.3896688	0.28630847
cg12125117	6180133	1.28E-11	6.77006914	7.3568239	19.9811595	0.29248786
cg07285167	6180133	6.42E-11	6.7715386	7.05891018	18.5077652	0.276892

cg11283860	4760338	1.29E-11	6.77589361	7.35658686	18.1481813	0.27298086
cg13265003	6420424	8.12E-11	6.78849491	7.01512185	31.6238964	0.39551016
cg11024597	4760338	5.35E-11	6.79564764	7.09300185	16.876543	0.25880348
cg21969640	3840554	4.73E-11	6.79853645	7.11605452	18.0984398	0.2724365
cg15322932	6420424	1.99E-11	6.80087382	7.27631018	33.1606751	0.40690936
cg12792367	5090739	2.55E-11	6.80443033	7.23051558	18.8225637	0.28028162
cg24821554	1850546	1.12E-12	6.80915811	7.79881744	25.767677	0.34773719
cg12125117	7320382	4.00E-11	6.8349119	7.14720675	23.2778438	0.32505881
cg24926276	6180133	1.81E-11	6.85256938	7.2937702	19.6642337	0.28919026
cg00565688	5270674	2.13E-11	6.85323463	7.26360273	18.5522192	0.27737259
cg08519905	5050347	1.16E-10	6.85481176	6.94816353	18.8183386	0.28023634
cg21969640	4210619	1.06E-11	6.8628373	7.39203414	22.0953726	0.3137268
cg15503752	70343	5.27E-11	6.86869814	7.09586013	17.4467227	0.26522815
cg04988978	6180088	4.48E-11	6.8758129	7.12615779	17.421429	0.26494551
cg10257049	4210619	1.62E-11	6.87711438	7.31456563	21.693457	0.30978797
cg08044694	4210619	7.47E-11	6.88400115	7.03076294	20.2572256	0.29533548
cg03330678	4210619	1.01E-11	6.89766303	7.40097953	22.1420551	0.31418138
cg07285167	6180088	1.02E-12	6.89979937	7.81503503	20.8782849	0.30165867
cg02595219	4760338	8.95E-11	6.90020981	6.99675769	16.4237858	0.25362132
cg18084554	4210619	6.64E-12	6.90104132	7.47733895	22.5428423	0.31805952
cg22242539	6180088	8.30E-14	6.90233978	8.26051736	23.2834814	0.32511194
cg27485921	6590228	2.93E-11	6.91203492	7.20463913	18.3797513	0.27550444
cg02266731	7330523	2.94E-11	6.91552351	7.2042236	30.983935	0.39063291
cg15958424	6420392	9.94E-11	6.92765552	6.97723922	27.6412638	0.36382245
cg23090046	6180088	7.47E-11	6.9307939	7.03066162	16.9673768	0.25983449
cg05868799	6420392	2.39E-11	6.94094996	7.24285792	29.1228968	0.37599166
cg11024597	4210619	1.34E-12	6.9506927	7.76632935	24.0969115	0.33269129
cg12971694	3840554	9.13E-12	6.97260897	7.41922426	19.5947833	0.28846352
cg19399532	6180133	5.65E-11	6.97812614	7.08281766	18.6237572	0.27814466
cg10787197	5090739	1.21E-12	6.97869923	7.7846921	21.655128	0.30940997
cg04653308	6650053	3.42E-12	6.99650382	7.59770979	23.3147983	0.32540693
cg14088811	6650053	3.61E-11	6.9965814	7.16616687	21.0630411	0.30351789
cg09305224	6180088	7.00E-11	6.99740156	7.04279818	17.0247422	0.26048414
cg09358725	6650053	4.97E-11	7.00684272	7.10671431	20.7631391	0.30049492
cg10126923	3990224	1.14E-10	7.01000906	6.9512881	22.7843654	0.32037546
cg09971811	7150017	1.63E-11	7.02345441	7.31346655	29.7604548	0.38108607
cg19906550	6180088	9.37E-11	7.02728408	6.9882139	16.7675187	0.2575622
cg12971694	4210619	1.97E-12	7.02812063	7.6977185	23.722617	0.32922496
cg24821554	6180088	3.74E-12	7.03766516	7.58163939	19.6713778	0.28926493
cg20070090	4760338	3.19E-11	7.0420531	7.18903083	17.3344526	0.26397194
cg12836863	5390730	2.32E-12	7.04337719	7.66774169	29.9608119	0.38266989
cg12836863	5270544	1.97E-13	7.04839409	8.10815571	29.7446403	0.38096071
cg12971694	4760338	1.93E-11	7.05024948	7.28229708	17.7850828	0.26898834
cg18084554	5270544	6.00E-12	7.05472347	7.49574065	26.2042107	0.35155721

cg22045288	5090739	4.88E-12	7.05721921	7.53337082	20.3446388	0.29623238
cg24211388	6590228	4.80E-12	7.05860199	7.53638304	20.0365922	0.29306149
cg12949760	6180088	3.00E-11	7.05860998	7.20075005	17.7803513	0.26893602
cg20340242	6180133	2.73E-11	7.08075661	7.21770056	19.285517	0.28520918
cg18794577	650619	2.41E-12	7.08377648	7.66151978	21.3403826	0.30629029
cg17105014	6180133	2.36E-11	7.08809049	7.24464518	19.4192081	0.28661963
cg23181133	510452	1.12E-10	7.09238186	6.95530584	17.8614695	0.2698319
cg09076077	6180088	5.66E-11	7.09681058	7.08246162	17.2129082	0.2626071
cg18787975	5090739	1.18E-11	7.10108194	7.37239843	19.5278569	0.28776178
cg26928972	5270544	2.68E-11	7.10347357	7.22113837	24.7069518	0.33826472
cg07730301	6180088	1.48E-11	7.10613255	7.33067982	18.4144709	0.2758813
cg21969640	4760338	1.50E-11	7.12088515	7.32894572	18.012652	0.27149573
cg21842274	5090739	8.08E-14	7.12573945	8.26494161	24.2789379	0.33436412
cg04404982	450202	5.52E-11	7.12899388	7.08712882	17.3303803	0.26392629
cg19812619	110347	6.44E-11	7.13246147	7.05836702	16.85993	0.25861461
cg20070090	5270544	4.43E-12	7.13299111	7.55088193	26.5116029	0.3542204
cg24777950	5050347	6.57E-11	7.14011914	7.05478345	19.3370604	0.28575363
cg21969640	5270544	3.06E-12	7.15671945	7.61790109	26.8882432	0.35745386
cg09971811	4200541	2.96E-11	7.15861584	7.20276034	26.7107249	0.35593391
cg21991396	6180088	3.89E-12	7.16305358	7.5745448	19.6352642	0.28888729
cg14859417	5090739	3.47E-13	7.16459617	8.00798113	22.8555207	0.32105476
cg02595219	4210619	9.31E-13	7.17229862	7.83205985	24.4586078	0.33600708
cg12966875	1770168	3.84E-11	7.17900383	7.15489908	27.7429446	0.36467274
cg22319147	6180088	9.31E-11	7.1792759	6.98952114	16.7736555	0.25763218
cg07028533	5270674	1.09E-10	7.18635116	6.9597264	17.090405	0.26122636
cg04404982	4490528	3.28E-11	7.18951081	7.18416653	19.6947526	0.28950914
cg17356733	6590228	1.51E-12	7.19699991	7.7454348	21.1189706	0.30407876
cg15880738	3310091	4.48E-11	7.21145868	7.12613902	17.9774761	0.27110928
cg14324675	5270544	1.69E-11	7.21360872	7.30596601	25.1635072	0.34237536
cg22242539	4200541	2.56E-11	7.2237568	7.22958896	26.8583128	0.3571981
cg07073964	5090739	4.58E-12	7.22485384	7.54509542	20.4048193	0.29684853
cg04451770	6180088	6.55E-12	7.22668268	7.47999558	19.1572072	0.28385026
cg20182358	5270674	2.69E-11	7.23198623	7.22043732	18.3407634	0.27508079
cg21991396	6180133	4.64E-11	7.23359079	7.11970654	18.8035005	0.28007726
cg12125117	4200541	3.50E-11	7.23400009	7.17172515	26.5406806	0.35447119
cg04353769	6180088	1.00E-13	7.23510429	8.22713417	23.0986224	0.32336539
cg25623459	6420424	6.35E-12	7.23844655	7.48555966	34.4323837	0.4160223
cg06812844	5270544	7.79E-11	7.24715291	7.02276656	23.6600978	0.32864245
cg05037688	1850546	6.90E-11	7.25306504	7.04562742	21.7423441	0.31026948
cg00899659	4210619	2.57E-12	7.25371486	7.64961418	23.4621729	0.32679166
cg04353769	6180133	2.71E-12	7.25906849	7.64018448	21.4390866	0.30727165
cg14511156	6180088	1.08E-11	7.25992622	7.38899861	18.7027818	0.27899561
cg24898863	6180133	6.37E-14	7.271823	8.3066225	25.0851585	0.34167357
cg22242539	4210619	5.08E-11	7.2731313	7.10255193	20.6151566	0.29899359

cg08525145	1850546	8.34E-11	7.27640648	7.01016058	21.5628662	0.30849841
cg09868035	6180133	1.81E-11	7.28311082	7.29389382	19.6648524	0.28919672
cg08519905	5270544	4.51E-11	7.28622709	7.12472041	24.1944899	0.33358908
cg08044694	4760338	3.63E-11	7.2893022	7.16519941	17.2202391	0.26268956
cg20720686	6590228	9.48E-11	7.29718208	6.9860215	17.3286658	0.26390707
cg02679745	5270544	2.01E-12	7.29752911	7.69409172	27.3204687	0.36112486
cg24898863	4210619	2.94E-11	7.30199203	7.2044211	21.1293043	0.30418229
cg08519905	4210619	1.35E-11	7.30328131	7.34718036	21.8621499	0.31144668
cg24091474	5050347	3.73E-12	7.30379624	7.58233099	22.0198942	0.31299053
cg14435807	6180088	3.17E-11	7.30720611	7.19025389	17.72962	0.26837462
cg20008332	5270674	2.05E-11	7.31235243	7.2708729	18.5879578	0.27775851
cg23090046	6180133	7.97E-11	7.3128649	7.01867046	18.3134195	0.27478337
cg21578541	5270674	9.20E-11	7.31325985	6.99177955	17.2416517	0.26293032
cg16717225	7160753	6.58E-11	7.32219337	7.05445589	24.7990122	0.33909773
cg02240622	6590228	5.46E-11	7.32380565	7.08929199	17.8211412	0.26938679
cg06812844	3840554	4.06E-11	7.32754152	7.14459597	18.2366491	0.27394703
cg08044694	5270544	7.72E-12	7.33013754	7.4499605	25.950715	0.34934438
cg24427660	4210619	4.26E-12	7.33191792	7.55803376	22.9708556	0.32215296
cg18638581	6180133	1.53E-11	7.3337178	7.32515021	19.8216176	0.29083166
cg15512851	4210619	1.17E-11	7.3371047	7.37397632	22.0013081	0.31280899
cg08458487	5270544	2.66E-11	7.33842145	7.22243302	24.7138797	0.33832748
cg12125117	5050347	1.42E-11	7.34055981	7.33779161	20.7522485	0.30038465
cg22421766	5090739	1.01E-11	7.3521439	7.40028232	19.6680779	0.28923044
cg10126923	160242	6.41E-12	7.35466217	7.48399069	30.6886971	0.38835622
cg02679745	5390730	1.12E-11	7.36107951	7.38249964	28.311532	0.36938589
cg24898863	5270544	5.36E-11	7.36586182	7.09282198	24.026466	0.33204163
cg02656594	6180088	3.86E-11	7.36632755	7.15379126	17.5539596	0.26642405
cg15779716	6180133	5.60E-11	7.37061408	7.08437606	18.6313316	0.27822631
cg23547429	1770168	3.62E-11	7.37123386	7.16584418	27.8037234	0.36517991
cg10059959	5270674	2.99E-11	7.37574283	7.20113389	18.2466089	0.27405564
cg23181133	1770168	4.92E-11	7.3769051	7.10863352	27.4870556	0.36252855
cg25087423	7400673	8.91E-11	7.38783893	6.99760583	20.0277982	0.29297055
cg16545105	5270544	6.86E-11	7.40333894	7.0466192	23.7844315	0.3297999
cg22933847	5090739	9.77E-11	7.40746849	6.98038214	17.6125408	0.2670757
cg24777950	5270544	8.66E-11	7.40805786	7.00304835	23.5576335	0.32768559
cg00333528	6420392	8.94E-11	7.41806395	6.99696668	27.7494017	0.36472666
cg11254522	1850546	9.46E-12	7.42720589	7.41278916	23.6535674	0.32858155
cg16330965	510452	9.28E-12	7.43011986	7.41638885	20.1298077	0.29402402
cg22016649	5090739	2.22E-14	7.4369148	8.49106187	25.5687018	0.34598102
cg18084554	6590228	6.89E-11	7.44674622	7.04584886	17.6130894	0.2670818
cg23713742	6180088	9.49E-11	7.4482168	6.98591841	16.7567452	0.25743932
cg18638581	6180088	2.12E-13	7.45160225	8.0946206	22.3722131	0.31641384
cg15503752	4490528	3.36E-12	7.45908243	7.60131533	21.8288005	0.31111939
cg24777950	4210619	2.01E-11	7.46656074	7.27427324	21.4860893	0.307738

cg08458487	4760338	3.43E-11	7.47275146	7.17592514	17.2715959	0.26326674
cg08458487	4210619	2.83E-12	7.48440558	7.63186512	23.3664892	0.32589326
cg16967583	6180088	4.44E-14	7.48440953	8.36963382	23.892935	0.33080672
cg24898863	4760338	7.58E-11	7.48521218	7.02806559	16.5703874	0.2553072
cg22242539	3840554	2.94E-11	7.48742903	7.20448251	18.5284425	0.27711562
cg01980222	4200541	2.43E-11	7.48772955	7.2391732	26.9111699	0.35764965
cg10126923	5270544	8.84E-14	7.48773919	8.24907534	30.5986412	0.38765838
cg03330678	6590228	6.66E-11	7.50371785	7.05230249	17.6439153	0.26742424
cg12582959	5910112	1.31E-11	7.50946064	7.35302841	18.634981	0.27826564
cg00333528	6180088	1.05E-11	7.51076764	7.39404026	18.7278135	0.27926474
cg24474182	6180088	1.25E-11	7.51601338	7.36149498	18.5665273	0.27752715
cg12125117	3840554	3.18E-11	7.53103087	7.18953884	18.4554023	0.27632507
cg11283860	6590228	2.23E-12	7.54152604	7.67509863	20.7514951	0.30037702
cg08399444	4760338	6.82E-11	7.54166767	7.04785523	16.6633915	0.25637279
cg22242539	4760338	6.62E-11	7.55476078	7.05341183	16.6895525	0.25667198
cg02266731	6180133	2.53E-11	7.57078461	7.2320183	19.3564953	0.2859587
cg16967583	6180133	7.99E-13	7.57219827	7.85932932	22.6043954	0.31865124
cg25957124	5910112	4.02E-12	7.57413607	7.56885743	19.7184178	0.28975622
cg04353769	5050347	2.60E-11	7.57415334	7.22661678	20.1896792	0.29464086
cg15958424	6180088	1.02E-13	7.57615061	8.22422404	23.082543	0.32321305
cg02266731	6180088	1.00E-12	7.57953059	7.81846976	20.8963194	0.30184059
cg16692277	6180088	4.31E-14	7.58066862	8.37527791	23.9246768	0.33110069
cg12836863	6590228	1.08E-12	7.58162147	7.80508332	21.4332347	0.30721355
cg17105014	5050347	5.23E-11	7.5839563	7.09726751	19.5459525	0.28795165
cg00071250	450202	4.59E-11	7.5866977	7.12142028	17.4942582	0.26575875
cg01623438	6180088	1.46E-11	7.5968851	7.33315508	18.4266614	0.27601352
cg21126943	5270544	3.34E-11	7.60022985	7.18048223	24.4900243	0.33629353
cg24091474	4210619	1.23E-12	7.6060595	7.78200226	24.1828783	0.33348237
cg05037688	6180088	8.89E-11	7.6098776	6.99814116	16.8141511	0.25809364
cg07285167	5050347	1.03E-11	7.61301597	7.39642853	21.0524238	0.30341132
cg09971811	5270544	8.98E-12	7.61721015	7.42231454	25.7983847	0.34800738
cg12125117	2810246	2.57E-11	7.62059306	7.2293405	35.5097316	0.42352616
cg09001777	6180088	1.49E-11	7.62283284	7.32947825	18.4085547	0.27581711
cg20191453	4210619	4.17E-11	7.62583413	7.13919472	20.7992546	0.30086035
cg13703437	6180088	5.88E-11	7.62858054	7.0753034	17.178871	0.26222398
cg15227982	4210619	3.36E-12	7.63016193	7.60102461	23.2007592	0.3243315
cg12125117	5270544	1.71E-11	7.63237875	7.30425865	25.1542653	0.34229266
cg11024597	6590228	6.00E-12	7.63882617	7.49580775	19.8299428	0.29091828
cg16465939	6650053	9.25E-11	7.64043139	6.99061675	20.1847007	0.29458961
cg02939139	730528	9.79E-11	7.64127008	6.97991783	16.4261113	0.25364812
cg16545105	4210619	3.44E-12	7.66457343	7.59710305	23.1797337	0.32413284
cg23889010	6180088	2.03E-11	7.66525128	7.27258535	18.1295404	0.27277696
cg08399444	5270544	8.10E-12	7.67090185	7.44128688	25.902862	0.34892497
cg24926276	4760338	1.09E-10	7.67476452	6.9598899	16.2519888	0.25163595

cg03600318	6180088	5.38E-11	7.67488259	7.09186164	17.2576574	0.26311018
cg04451770	6180133	4.73E-12	7.67815488	7.53908006	20.9125693	0.30200443
cg17749456	6180088	3.18E-12	7.68551288	7.61119769	19.8222025	0.29083775
cg24576425	4760338	8.00E-11	7.68881771	7.01782617	16.5223685	0.25475584
cg02204046	650619	8.13E-11	7.69155012	7.01483146	18.0940469	0.27238839
cg02656594	6180133	7.04E-11	7.69166657	7.0417441	18.4247224	0.2759925
cg14726637	5910112	6.27E-11	7.69610769	7.06348791	17.2307464	0.26280772
cg16545105	4760338	2.98E-11	7.69784868	7.20156146	17.3946585	0.26464613
cg25028542	6180088	1.92E-11	7.69858714	7.28300744	18.1804898	0.27333401
cg24091474	4760338	7.63E-12	7.6994852	7.45208509	18.6203518	0.27810795
cg22242539	5390730	5.53E-11	7.69957358	7.08679855	26.6677746	0.35556508
cg08368934	4210619	1.09E-12	7.70309421	7.80386016	24.3030595	0.33458517
cg11254522	1770168	2.85E-13	7.70318521	8.04285197	32.9757662	0.40556059
cg10787197	290603	5.00E-11	7.71305931	7.10556268	18.0839006	0.27227723
cg17753124	1940288	2.29E-13	7.71452706	8.08131119	23.9696834	0.33151706
cg15958424	4200541	1.13E-10	7.72600144	6.95336277	25.3650139	0.34417344
cg09076123	4200541	1.65E-12	7.73150344	7.72923752	29.7072141	0.38066384
cg06810647	5910112	5.58E-11	7.73278613	7.08519152	17.3340505	0.26396743
cg02595219	6590228	1.21E-11	7.7390823	7.36846055	19.1886104	0.28418332
cg15352315	4210619	6.00E-11	7.75483175	7.07172772	20.4610254	0.29742301
cg21019522	7320382	1.53E-11	7.77229824	7.3252368	24.2188165	0.33381253
cg11024597	1580411	4.18E-11	7.77982613	7.13908957	21.728542	0.31013361
cg09671611	1850546	1.79E-11	7.78330933	7.29601584	23.0351891	0.32276399
cg08840010	6180088	9.70E-14	7.79149602	8.23284925	23.1302164	0.32366453
cg02590345	1260193	3.01E-11	7.79953072	7.19988188	20.2413507	0.29517235
cg04353769	3840554	4.66E-11	7.80073186	7.11869106	18.1111838	0.27257605
cg01599709	4210619	5.48E-12	7.80783894	7.512373	22.7281021	0.31983736
cg00031162	110347	2.48E-11	7.82338709	7.23555088	17.7074372	0.26812887
cg00899659	4760338	3.21E-13	7.83044849	8.02151865	21.5622112	0.30849193
cg26701826	6180133	5.01E-11	7.83193326	7.10515589	18.73249	0.27931499
cg24427660	6590228	7.76E-11	7.83240251	7.02368465	17.5074363	0.26590571
cg25882366	6020523	4.99E-11	7.83676229	7.10619606	17.7617845	0.26873066
cg10057295	4200541	1.55E-11	7.84050262	7.32234055	27.3727793	0.3615663
cg13434842	4210619	9.29E-13	7.84142765	7.83231721	24.46003	0.33602006
cg24926276	5270544	1.06E-11	7.84211976	7.39213927	25.6327643	0.34654747
cg17749456	6180133	2.22E-11	7.84372237	7.25649713	19.4781717	0.28723993
cg24673765	4210619	2.50E-11	7.84887769	7.23425994	21.2812915	0.30570145
cg05512099	6180088	7.23E-11	7.85144889	7.03678394	16.9963026	0.26016221
cg16008138	4560576	1.01E-10	7.8520928	6.97456848	16.93931	0.25951623
cg26301908	4540328	6.02E-11	7.85241101	7.07088712	17.4736609	0.26552893
cg09076123	5270544	6.80E-12	7.85660755	7.47304958	26.0783706	0.3504606
cg15503752	360475	6.03E-12	7.86493395	7.49511471	18.8525538	0.28060289
cg15337006	4210619	8.55E-12	7.86637442	7.43129927	22.3007017	0.31572176
cg21581873	6420424	2.32E-11	7.86807211	7.24773098	32.9897841	0.40566305

cg15361750	5270520	5.76E-12	7.86828131	7.50317257	25.2409487	0.34306755
cg10057295	5270544	1.02E-10	7.87136377	6.97229657	23.3984091	0.32619323
cg20340242	4210619	2.58E-11	7.87238027	7.22862206	21.2525262	0.30541444
cg07285167	5390730	1.15E-10	7.88136102	6.94903892	25.9249306	0.34911846
cg12836863	3830349	1.59E-11	7.88198521	7.31729117	21.2594494	0.30548354
cg03554552	6020523	9.69E-11	7.88738694	6.98195383	17.1700182	0.26212427
cg26928972	6590228	1.90E-11	7.89715939	7.2851138	18.774818	0.27976956
cg23889010	6180133	7.48E-11	7.89799266	7.03054607	18.3706596	0.2754057
cg10257049	6590228	5.20E-12	7.91030434	7.52195811	19.9629984	0.29229972
cg13784792	5270674	9.68E-13	7.91073842	7.82511617	21.4177597	0.30705984
cg07285167	5270544	1.25E-11	7.91496622	7.36158732	25.4657638	0.34506877
cg21842274	70707	8.00E-12	7.91501892	7.44354013	18.521452	0.27704003
cg10521852	4210619	3.47E-12	7.92764593	7.59554505	23.1713834	0.32405392
cg02868338	1940288	1.07E-10	7.93097132	6.96371297	18.1889883	0.27342684
cg22794078	5270674	1.13E-11	7.93478607	7.3805143	19.1312674	0.2835749
cg16967583	4210619	2.25E-11	7.93890314	7.25345837	21.3794127	0.30667868
cg01980222	5270544	8.84E-12	7.94019671	7.42526343	25.8146062	0.34815001
cg06196379	6180088	1.33E-15	7.94630194	8.97320489	27.4085619	0.36186792
cg15322932	2810400	3.74E-11	7.96486975	7.15969375	25.9880045	0.34967084
cg26701826	6180088	8.26E-13	7.96900381	7.85336835	21.0800079	0.30368813
cg15361750	5390730	1.08E-11	7.96908936	7.38828733	28.3443753	0.369656
cg03554552	4210619	4.63E-12	7.96931513	7.54291775	22.8903291	0.32138658
cg12089698	4210619	1.36E-11	7.97056281	7.34685083	21.8604418	0.31142992
cg17105014	3840554	3.04E-11	7.98089444	7.19811078	18.4972808	0.27677856
cg04988978	4200541	2.56E-11	7.99273775	7.22951676	26.8579148	0.35719469
cg21969640	6590228	1.53E-12	7.99731287	7.74331392	21.1078409	0.30396722
cg06394229	6180088	5.33E-12	7.99999369	7.5173133	19.3451747	0.28583926
cg12125117	5390730	1.06E-11	8.00034048	7.3920989	28.3660188	0.36983388
cg10126923	5390730	1.20E-14	8.00044914	8.59848424	35.7772043	0.42535936
cg22242539	5270544	6.79E-13	8.00549919	7.8885651	28.4432074	0.37046743
cg16967583	3840554	3.85E-11	8.00556547	7.15422775	18.283415	0.27445673
cg09001777	6180133	1.93E-11	8.01524902	7.28213634	19.6060568	0.28858158
cg12125117	4210619	2.57E-13	8.02264335	8.06128071	25.7437961	0.34752691
cg06379754	5270674	2.17E-11	8.03001937	7.26041779	18.5365739	0.27720352
cg22016649	7330097	2.41E-11	8.03576293	7.24070776	24.4898092	0.33629157
cg15322932	1770168	5.27E-11	8.04489551	7.0960006	27.4174731	0.36194299
cg17753124	5090739	2.09E-14	8.05338563	8.5021544	25.632868	0.34654839
cg15958424	6180133	6.64E-14	8.05375118	8.2995409	25.0448133	0.34131161
cg07285167	4210619	1.36E-12	8.05788144	7.76372722	24.0826554	0.33255992
cg17714799	4760338	1.05E-10	8.06630434	6.96653803	16.2829008	0.25199396
cg24713204	4210619	5.29E-11	8.08468631	7.09528625	20.5787654	0.2986234
cg10266490	6590228	8.44E-11	8.08624344	7.00776047	17.4317339	0.26506069
cg23889010	7320382	1.00E-10	8.09662576	6.97518929	22.3906413	0.31659195
cg21019522	4200541	3.07E-11	8.09930439	7.19647123	26.676207	0.35563752

cg09076077	4200541	8.17E-11	8.10346682	7.01383735	25.6869706	0.34702601
cg16989646	4210619	1.14E-12	8.10528809	7.79549991	24.2570526	0.33416343
cg03014628	5270674	1.24E-12	8.11298549	7.78088589	21.1843035	0.30473279
cg12380764	5270544	1.68E-12	8.11802701	7.72667438	27.5066214	0.36269301
cg17173423	6590228	6.32E-11	8.11832678	7.06193136	17.6899604	0.26793514
cg20764656	4210619	6.80E-13	8.12379917	7.88821878	24.7700493	0.33883589
cg22016649	6650053	9.08E-14	8.12983519	8.24459739	26.9367645	0.35786807
cg24576425	4210619	2.68E-13	8.1307681	8.05386099	25.7016149	0.34715517
cg09971811	5390730	1.77E-12	8.13926462	7.71707338	30.2523904	0.38496038
cg20340242	4760338	4.31E-11	8.13935437	7.13321931	17.0675686	0.2609684
cg24467291	5270674	3.24E-11	8.1571699	7.18650167	18.1754066	0.27327847
cg21019522	5050347	1.82E-11	8.16122317	7.29305599	20.5248437	0.29807417
cg25832796	6180133	8.64E-11	8.16974513	7.00348081	18.240347	0.27398736
cg15503752	3310091	6.76E-11	8.17426796	7.04938776	17.6089768	0.26703609
cg24777950	4760338	1.28E-12	8.17782922	7.77451161	20.2595104	0.29535895
cg26191951	6420392	6.80E-11	8.18417997	7.04820761	28.0317099	0.36707515
cg10787197	7330097	7.06E-12	8.18507785	7.46636824	25.7139823	0.34726421
cg13703437	6180133	2.53E-11	8.19082855	7.23187772	19.3557977	0.28595134
cg26202340	5910500	5.63E-11	8.19573056	7.08357598	26.9288913	0.35780089
cg19906550	4200541	4.62E-11	8.19597797	7.12033713	26.2607361	0.35204858
cg21991396	5270544	4.63E-11	8.19799975	7.12003822	24.1697794	0.33336196
cg17105014	4760338	2.68E-11	8.19946371	7.22135776	17.4899874	0.26571111
cg04404982	3310091	7.04E-11	8.20209925	7.0419209	17.5733398	0.26663977
cg15958424	3840554	3.75E-11	8.2049092	7.15934313	18.3082777	0.27472742
cg10236239	4760338	3.96E-11	8.20999294	7.14918877	17.1437202	0.26182791
cg25028542	6180133	1.11E-11	8.21489088	7.38330446	20.1150717	0.29387203
cg22045288	6650053	3.46E-13	8.21663395	8.00818837	25.5787982	0.34607036
cg15910079	6420392	4.08E-11	8.21699533	7.14336965	28.5614634	0.37143558
cg14700707	6180088	1.68E-12	8.22175622	7.72568702	20.4119368	0.29692133
cg10106388	6180088	3.50E-11	8.22793065	7.17171272	17.6401855	0.26738282
cg00899659	6590228	1.76E-12	8.22812794	7.71822561	20.976417	0.30264742
cg04404982	1030270	9.45E-11	8.22827344	6.98664789	17.6753665	0.26777329
cg22820108	6180133	2.14E-11	8.22929205	7.2624929	19.5080374	0.2875537
cg08044694	6590228	2.79E-12	8.24038724	7.63483619	20.5426503	0.29825564
cg12978308	4210619	4.34E-11	8.24066082	7.13203478	20.7632077	0.30049562
cg17166812	6180088	5.86E-11	8.24247968	7.07614786	17.1828846	0.26226918
cg23090046	3840554	9.42E-11	8.2453783	6.98721741	17.4814429	0.26561578
cg15361750	6590228	4.46E-11	8.25005148	7.1268949	18.0022564	0.27138157
cg07285167	4760338	4.17E-12	8.25706705	7.56207323	19.1717105	0.28400412
cg04425624	2490113	1.41E-11	8.25936637	7.34008885	74.2209427	0.60561692
cg16967583	5270544	7.78E-12	8.25946569	7.44851389	25.94273	0.34927444
cg09419900	6180088	1.53E-11	8.26249093	7.32492231	18.3861314	0.27557372
cg09076123	5390730	3.39E-12	8.26291772	7.59941448	29.5600474	0.3794937
cg09868035	4760338	5.69E-11	8.26306243	7.08157526	16.8224658	0.25818831

cg25623459	1770168	1.09E-10	8.26811258	6.9605354	26.6791053	0.35566242
cg14324675	6590228	1.90E-12	8.27855785	7.70443601	20.9043625	0.3019217
cg18638581	5270544	1.88E-11	8.27955898	7.28703679	25.061165	0.34145836
cg17386185	5090739	7.32E-13	8.28155235	7.87495217	22.1362751	0.31412513
cg12640109	7570324	1.47E-11	8.29454396	7.33158717	32.8531624	0.4046629
cg15439862	4210619	7.43E-11	8.29579938	7.03163301	20.2615419	0.29537982
cg10236239	4210619	1.95E-12	8.29700908	7.69894429	23.729275	0.32928693
cg10599444	5910112	1.02E-10	8.30045313	6.97179388	16.7978019	0.2579074
cg18638581	4760338	5.34E-11	8.30480254	7.09339805	16.8784196	0.25882481
cg25832796	6180088	1.66E-12	8.30805615	7.72855891	20.4268431	0.29707375
cg15512851	6590228	8.98E-12	8.30937774	7.42229124	19.458366	0.28703169
cg23181133	6180088	4.18E-12	8.31291316	7.56168063	19.5698679	0.28820243
cg07218880	730528	9.30E-11	8.31409711	6.9896169	16.4714055	0.25416977
cg09305224	5270544	1.02E-10	8.31486654	6.9719933	23.3968423	0.32617852
cg24576425	6590228	7.09E-11	8.32051724	7.04053006	17.5877052	0.26679958
cg21019522	2810246	8.08E-11	8.32371216	7.01599891	34.2180096	0.41450579
cg21842274	6180088	5.07E-11	8.32438095	7.10308015	17.3111413	0.26371056
cg08319404	4210619	1.46E-11	8.32500239	7.33346427	21.7911149	0.31074918
cg24474182	6180133	6.81E-13	8.34189528	7.88798742	22.7592221	0.3201351
cg06394229	6180133	1.11E-11	8.34233461	7.38306252	20.113846	0.29385939
cg20125091	4200541	2.39E-11	8.34424895	7.24230071	26.9284333	0.35779699
cg21991396	4760338	5.92E-11	8.3477822	7.07420629	16.7876378	0.25779158
cg07730301	4210619	1.36E-11	8.3509352	7.34691688	21.8607842	0.31143328
cg25957124	6590228	8.67E-12	8.35283527	7.42878794	19.4910549	0.28737532
cg04353769	5270544	1.07E-12	8.35391643	7.80761293	27.9724475	0.3665836
cg24821554	1030768	3.86E-12	8.37169536	7.5761585	19.5910395	0.2884243
cg19906550	3840554	1.09E-10	8.37306069	6.95944511	17.3499191	0.26414525
cg16692277	4760338	2.53E-11	8.37335754	7.23180001	17.5403774	0.2662728
cg17813891	3310091	8.66E-11	8.39128331	7.00308272	17.3885863	0.26457819
cg03886110	6590228	3.31E-11	8.39284031	7.18251413	18.2719042	0.27433134
cg08368934	6590228	7.86E-12	8.39709391	7.44676893	19.5816776	0.28832621
cg08965235	5910112	2.76E-11	8.39958553	7.21621835	17.9644403	0.27096597
cg23547429	6180088	1.45E-12	8.40515636	7.75238477	20.5507228	0.29833787
cg08965235	4210619	1.67E-11	8.40797853	7.30908554	21.665186	0.3095092
cg19531130	4210619	2.19E-11	8.40929577	7.25873612	21.4064323	0.30694729
cg10126923	7550358	9.91E-11	8.41300326	6.97778769	31.0579589	0.39120108
cg18638581	4210619	2.35E-12	8.41563343	7.66554156	23.5482254	0.32759759
cg22598028	6020523	2.73E-11	8.42238875	7.21766753	18.3016069	0.27465481
cg24821554	3990224	7.46E-11	8.43894104	7.03088607	23.1937926	0.32426569
cg13765621	6180088	4.05E-12	8.44130514	7.56717611	19.5977911	0.28849502
cg04655481	4760338	3.95E-12	8.44345925	7.57183796	19.2210504	0.28452706
cg15433631	4210619	1.32E-11	8.44445304	7.35180932	21.8861531	0.31168205
cg22016649	6180088	8.33E-11	8.44831635	7.01040417	16.8718469	0.2587501
cg06849477	7320382	4.39E-11	8.45359887	7.12996212	23.1879276	0.32421027

cg07525077	6180088	9.21E-12	8.46153659	7.41773061	18.8456643	0.28052911
cg08458487	6590228	2.32E-12	8.46479288	7.668316	20.716236	0.30001977
cg18638581	3840554	4.94E-12	8.46521958	7.53135143	20.1640174	0.2943766
cg09305224	4210619	1.39E-11	8.46585936	7.34232956	21.8370127	0.31120001
cg24091474	1580411	5.14E-11	8.46714091	7.1004425	21.5317933	0.30819086
cg26191951	6180133	4.67E-11	8.46744283	7.11829497	18.7966053	0.28000332
cg25623459	2810400	1.25E-11	8.47624231	7.36245923	27.1030795	0.35928378
cg17749456	5050347	2.89E-11	8.47852487	7.20760423	20.0943314	0.293658
cg16545105	6590228	9.72E-12	8.4836916	7.40795764	19.3863458	0.28627345
cg10787197	6180088	5.35E-11	8.48881988	7.09304361	17.2632884	0.26317344
cg23090046	4210619	7.51E-12	8.48930086	7.45488229	22.4245472	0.31691943
cg09037813	4210619	2.66E-15	8.4909527	8.8568481	30.4919712	0.38682973
cg14435807	5050347	1.61E-11	8.49311971	7.31547702	20.6386428	0.2992323
cg04784315	6590228	5.32E-11	8.49721005	7.09396812	17.8436117	0.26963487
cg21019522	4210619	6.43E-12	8.5008694	7.48318608	22.5737017	0.31835631
cg04353769	4760338	1.43E-12	8.50694769	7.75526187	20.1596997	0.29433212
cg24091474	6590228	1.95E-12	8.5087944	7.69947324	20.8784621	0.30166046
cg19906550	5050347	4.91E-12	8.52086419	7.53220678	21.7566741	0.3104105
cg15958424	5050347	3.83E-13	8.52413085	7.99060341	24.2289018	0.33390512
cg04404982	4180544	9.86E-11	8.52609778	6.97861087	17.9219943	0.27049892
cg22381196	6590228	1.31E-11	8.53299387	7.35281021	19.110552	0.28335485
cg20070090	6590228	2.42E-14	8.53591483	8.47528339	25.1299746	0.34207519
cg06810647	4210619	8.79E-14	8.5464165	8.24993732	26.8293611	0.3569505
cg16967583	4760338	3.81E-12	8.55665615	7.57848067	19.2546516	0.28488276
cg06812844	6590228	1.88E-12	8.56463492	7.70588156	20.9119098	0.30199778
cg16692277	5270544	1.80E-12	8.57383524	7.71343543	27.4308891	0.36205597
cg25050026	4760338	2.80E-11	8.57393041	7.21345166	17.4518843	0.2652858
cg26154999	6590228	5.26E-11	8.57667902	7.09629896	17.8548178	0.26975852
cg09303642	4210619	9.30E-14	8.58148077	8.24052876	26.7746276	0.35648189
cg03574571	4210619	3.34E-13	8.58634903	8.01436891	25.4777554	0.34517517
cg04655481	6590228	9.83E-11	8.58673927	6.97922366	17.2965018	0.26354632
cg11254522	4200541	1.10E-10	8.59663832	6.95755172	25.3872253	0.34437104
cg08965235	6020523	5.93E-11	8.59693756	7.07374975	17.6062356	0.26700562
cg19906550	5270544	2.87E-11	8.60984407	7.20867827	24.6403387	0.33766067
cg12971694	6590228	6.66E-15	8.61230476	8.69827242	26.4274347	0.35349335
cg15337006	6590228	4.07E-11	8.61453693	7.14414064	18.0856413	0.2722963
cg19731268	5270674	1.11E-10	8.62157912	6.95605585	17.0731295	0.26103123
cg17105014	4210619	9.37E-14	8.62244929	8.23906017	26.7660898	0.35640873
cg15779716	4210619	3.17E-12	8.62501368	7.61138715	23.2563703	0.32485636
cg26055770	4210619	2.02E-12	8.62524883	7.69331567	23.6987115	0.32900234
cg02266731	5270544	1.90E-11	8.62577194	7.28483777	25.0492931	0.34135182
cg16232126	4210619	4.27E-11	8.62684145	7.13503795	20.7783228	0.3006486
cg04451770	4760338	2.63E-11	8.63191019	7.2249954	17.5075329	0.26590678
cg16545105	1580411	6.65E-11	8.63570927	7.05253265	21.2893711	0.30578202

cg08399444	6590228	2.66E-12	8.63765179	7.6434154	20.5870593	0.29870781
cg24474182	4210619	3.76E-11	8.63947335	7.15861938	20.8972308	0.30184978
cg12125117	1580411	7.94E-11	8.63962135	7.01936655	21.1225132	0.30411426
cg04655481	4210619	5.42E-14	8.64652167	8.33526718	27.3286126	0.36119363
cg20764656	6590228	1.93E-11	8.64708251	7.2819247	18.7590785	0.2796006
cg15439862	6020523	9.41E-11	8.64857041	6.9874795	17.1961152	0.26241813
cg14511156	4210619	4.37E-12	8.65427686	7.55332098	22.9457322	0.32191404
cg24354652	4210619	4.44E-15	8.65529738	8.77388584	29.9759728	0.38278941
cg00333528	5270544	7.76E-11	8.65856916	7.0235954	23.6644112	0.32868267
cg21019522	5390730	5.55E-11	8.66151386	7.08625061	26.6647911	0.35553944
cg26191951	6180088	4.54E-13	8.68535708	7.9600679	21.6466946	0.30932675
cg04988978	5270544	2.25E-12	8.68673982	7.67354011	27.2034568	0.3601352
cg15662251	6180088	8.37E-11	8.68850814	7.00940152	16.8671258	0.25869643
cg17714799	4210619	1.01E-13	8.70163789	8.22510943	26.685062	0.35571358
cg19399532	4760338	9.01E-13	8.70620735	7.83789008	20.5898791	0.2987365
cg27096144	4210619	4.74E-11	8.71726612	7.11571649	20.6811879	0.2996643
cg09305224	5390730	6.46E-11	8.71943976	7.05782917	26.5103516	0.3542096
cg12949760	5270544	5.29E-12	8.72131763	7.51878233	26.3323855	0.35267035
cg21917349	540368	1.04E-10	8.73484262	6.96777594	17.769685	0.26881806
cg12311132	6180133	7.78E-11	8.74264576	7.02299402	18.3342478	0.27500994
cg15361750	5270544	1.55E-15	8.74603587	8.94722281	35.0465838	0.42032404
cg16270890	5270674	2.04E-11	8.74987116	7.27173086	18.5921777	0.27780405
cg19491035	4210619	4.18E-12	8.7608383	7.56167072	22.9902546	0.32233733
cg22242539	6590228	1.12E-12	8.77186426	7.79889791	21.4005342	0.30688868
cg12262564	4210619	1.60E-12	8.78266628	7.73518478	23.9265961	0.33111845
cg14564494	4210619	3.28E-11	8.79183097	7.18408784	21.0260947	0.30314689
cg26215727	4210619	8.34E-11	8.79416156	7.01003459	20.1545531	0.29427909
cg12582959	4210619	2.22E-16	8.79606631	9.40592733	34.0294939	0.41316568
cg24467291	7570600	6.32E-12	8.80337019	7.48630572	23.1809272	0.32414412
cg15910079	6180133	3.65E-12	8.81683395	7.58624388	21.1573096	0.30446271
cg25957124	4210619	1.00E-17	8.82070911	9.64006099	35.6026245	0.42416415
cg17296078	4210619	6.96E-12	8.82578862	7.46881799	22.4979145	0.31762697
cg15528736	4210619	1.24E-11	8.83302651	7.36341049	21.9463766	0.31227187
cg06092815	4210619	6.13E-12	8.83515938	7.49200149	22.6202724	0.31880371
cg10057295	5390730	1.28E-12	8.85383761	7.77444151	30.5938206	0.38762098
cg12380764	6590228	3.19E-12	8.87084564	7.61065428	20.4177446	0.29698072
cg00071250	730528	3.75E-11	8.87311924	7.1592553	17.2737785	0.26329125
cg02154186	5270674	7.21E-12	8.87871136	7.46252685	19.5429829	0.2879205
cg10236239	6590228	3.58E-11	8.87998036	7.16758601	18.1993253	0.27353973
cg26112639	4760338	3.98E-12	8.88184468	7.57022997	19.2129211	0.28444095
cg10275770	4210619	3.44E-11	8.89151112	7.17496388	20.9798772	0.30268223
cg19399532	4210619	1.04E-14	8.89899326	8.62236045	29.0460682	0.3753721
cg00333528	4760338	6.04E-11	8.9031419	7.07048288	16.7700537	0.25759111
cg08840010	5390730	1.74E-11	8.90438463	7.3007656	27.8504662	0.3655694

cg23889010	5270544	9.52E-11	8.90754465	6.98523302	23.4653051	0.32682103
cg05125838	1850546	2.19E-12	8.91271553	7.67867018	25.0981815	0.34179033
cg02626929	5270544	7.15E-11	8.91698537	7.03889353	23.7441147	0.32942502
cg10307548	4200541	6.28E-11	8.91781788	7.06313392	25.9514801	0.34935108
cg15958424	5390730	3.21E-12	8.9275409	7.60914041	29.6168747	0.37994606
cg09868035	4210619	3.49E-14	8.94067149	8.41212452	27.78269	0.36500449
cg12836863	5390246	1.20E-11	8.94395433	7.36947596	31.3515459	0.3934441
cg12125117	1030296	3.46E-12	8.94718093	7.59582331	34.1093184	0.41373388
cg13471990	6180088	1.32E-13	8.95324771	8.17864985	22.831474	0.32082535
cg02266731	4760338	8.55E-12	8.95442194	7.43130305	18.517081	0.27699276
cg00622552	5390730	7.53E-11	8.9658212	7.02928169	26.3558522	0.35287374
cg09001777	5270544	3.84E-11	8.96639514	7.15475438	24.3533819	0.33504584
cg09303642	6590228	9.96E-12	8.97034142	7.40341317	19.3635409	0.28603301
cg12125117	6590228	4.23E-13	8.97378205	7.97265167	22.3289849	0.31599565
cg12640109	4200541	6.24E-12	8.98171974	7.4886735	28.3118069	0.36938815
cg09088576	4210619	2.71E-13	8.98479305	8.05139976	25.6876314	0.34703184
cg15910079	6180088	2.98E-14	8.9854873	8.43899428	24.2844951	0.33441506
cg17977409	4210619	9.18E-11	9.0049986	6.99207422	20.0658362	0.29336374
cg18787975	6180088	4.60E-11	9.00733597	7.12104074	17.3969438	0.26467169
cg08840010	3990224	2.51E-11	9.007628	7.2337289	24.258229	0.33417422
cg13703437	4210619	4.13E-11	9.01222715	7.14123319	20.8095239	0.30096419
cg07509155	4210619	2.04E-14	9.01314438	8.50500543	28.3369973	0.36959535
cg06147863	4210619	8.12E-12	9.01396147	7.44072507	22.3501539	0.3162005
cg02181506	4200541	7.76E-11	9.01487173	7.02359216	25.7391647	0.34748612
cg08525145	4200541	2.72E-11	9.01904826	7.21873454	26.798535	0.35668666
cg24898863	6590228	1.17E-13	9.02288903	8.19976381	23.5734006	0.327833
cg17749456	4210619	3.01E-12	9.0254533	7.62089548	23.3074639	0.32533786
cg26112639	4210619	8.90E-14	9.03217857	8.24808162	26.8185608	0.35685808
cg24821554	6590228	1.96E-11	9.03681743	7.27933997	18.746327	0.27946365
cg14603345	6020523	1.75E-12	9.03801882	7.71916985	20.8341603	0.30121317
cg19906550	4210619	5.19E-13	9.03939147	7.9362537	25.038203	0.34125227
cg04600618	5910112	1.63E-11	9.03995906	7.31324486	18.4386932	0.27614398
cg26215727	5270544	5.65E-11	9.0434457	7.08286974	23.9741973	0.33155879
cg23001650	6420424	5.48E-12	9.05093367	7.51228547	34.5974055	0.41718434
cg00071250	4180544	9.13E-11	9.05506391	6.99305195	17.9910083	0.271258
cg16232126	6020523	3.82E-11	9.05540355	7.15565164	18.0002459	0.27135949
cg24777950	6590228	2.28E-13	9.07094107	8.0824676	22.9263352	0.32172947
cg23713742	4210619	1.31E-11	9.07619876	7.35365915	21.8957495	0.3117761
cg11254522	5270544	5.18E-11	9.08415458	7.09892484	24.0585543	0.33233771
cg12089698	6590228	7.10E-12	9.08877461	7.46510674	19.6743244	0.28929572
cg23037403	4210619	1.19E-11	9.09664408	7.3709863	21.9857551	0.312657
cg20395892	4210619	2.17E-11	9.10852664	7.26001169	21.4129657	0.30701221
cg04653308	4210619	5.36E-11	9.110253	7.09286607	20.5666519	0.29850009
cg02656594	4210619	1.81E-12	9.12644345	7.71237347	23.8022857	0.32996578

cg09868035	6590228	1.71E-11	9.12669093	7.30465407	18.8714073	0.2808047
cg14726637	4210619	4.44E-16	9.13736136	9.16806467	32.4709144	0.40184663
cg15958424	5270544	5.88E-14	9.14293009	8.32067643	31.0381956	0.39104949
cg09671611	4200541	9.12E-11	9.14857029	6.99323002	25.5769474	0.34605399
cg19713460	4210619	3.65E-13	9.15134114	7.99869058	25.3891885	0.3443885
cg24256211	4210619	1.56E-11	9.15533311	7.32090492	21.7261869	0.31011042
cg06196379	6590228	1.31E-11	9.16619858	7.35368603	19.1149159	0.28340122
cg24821554	5390730	1.86E-13	9.18619399	8.11818185	32.6925838	0.40348305
cg21494776	5910112	6.73E-12	9.19482249	7.47492402	19.243031	0.28475979
cg12640109	5270544	1.58E-11	9.19537715	7.31884791	25.2333058	0.3429993
cg00278366	4200541	1.00E-11	9.20127681	7.40264018	27.8234752	0.36534455
cg15374234	4210619	1.00E-10	9.20420763	6.97548496	19.9840943	0.29251825
cg17386185	1770168	7.64E-11	9.21108094	7.02642301	27.0364553	0.35871741
cg24474182	5270544	3.27E-12	9.21342295	7.60599565	26.8210928	0.35687975
cg01129847	4200541	6.31E-11	9.21516859	7.06226077	25.9467789	0.3493099
cg15958424	4760338	1.64E-13	9.21584226	8.14009536	22.2020514	0.31476473
cg21237418	4210619	2.00E-15	9.21680119	8.90187661	30.7740672	0.38901629
cg19531130	6590228	8.70E-11	9.22940323	7.00219057	17.4052956	0.26476511
cg07285167	6590228	4.97E-13	9.23004313	7.94410804	22.1750581	0.3145024
cg23889010	4210619	4.73E-12	9.23159436	7.53928106	22.8709797	0.32120217
cg10536916	6590228	7.59E-11	9.24719789	7.02781081	17.5270798	0.26612466
cg03567830	4210619	6.71E-12	9.25465064	7.47548062	22.5330397	0.31796519
cg08525145	1030768	4.07E-11	9.26124124	7.14377275	17.4550644	0.26532132
cg09305224	6590228	6.61E-11	9.2643834	7.05355772	17.6499143	0.26749084
cg08519905	6590228	6.00E-15	9.26553144	8.7138751	26.51948	0.35428835
cg23181133	4200541	2.31E-11	9.27274394	7.24914452	26.9662362	0.35811939
cg22016649	7320382	5.64E-11	9.27516662	7.08318131	22.9450991	0.32190802
cg06394229	5270544	3.12E-11	9.27562832	7.19315365	24.5575033	0.33690796
cg04164824	6590228	6.36E-11	9.27903024	7.06076187	17.6843646	0.26787309
cg02357714	4210619	3.22E-13	9.28007455	8.02101971	25.5153782	0.34550878
cg24821554	5270544	5.55E-15	9.28312284	8.7309366	33.6299979	0.41030541
cg17749456	4760338	7.03E-12	9.28632541	7.4669376	18.694334	0.27890474
cg16361890	4210619	2.78E-11	9.28766536	7.21483951	21.1823002	0.30471276
cg26701826	5270544	4.10E-12	9.29130878	7.56501979	26.5907791	0.35490282
cg24474182	4760338	6.98E-12	9.3039703	7.46838291	18.7015412	0.27898227
cg02656594	4760338	7.20E-12	9.30707884	7.46275749	18.6734974	0.27868051
cg09106999	6420392	5.34E-12	9.3184165	7.51694407	30.7097877	0.38851942
cg22016649	6180133	8.27E-12	9.32206262	7.43737098	20.3899802	0.2966967
cg14611112	6180088	1.14E-10	9.32563232	6.95077074	16.5922284	0.25555772
cg02635407	4210619	2.09E-13	9.37253993	8.09739254	25.949647	0.34933503
cg04653308	4760338	1.12E-10	9.37292336	6.95425263	16.2258001	0.25133237
cg02927346	4210619	1.07E-10	9.37486029	6.96321637	19.9237669	0.29189296
cg16232126	7570408	9.23E-12	9.39318612	7.41733928	19.0082292	0.28226594
cg06196379	5270544	3.55E-15	9.40399294	8.81341283	34.1660946	0.41413735

cg25028542	5270544	6.12E-12	9.40902909	7.49223316	26.1847339	0.35138772
cg07073964	6180088	4.30E-12	9.41070277	7.55625603	19.5423247	0.28791359
cg10362591	5910500	8.80E-12	9.41370826	7.42596048	28.8445403	0.37374106
cg27019278	6590228	4.65E-11	9.4166988	7.11928983	17.9655491	0.27097816
cg22820108	4210619	3.96E-12	9.41953398	7.57158213	23.0431679	0.3228397
cg05037688	5270544	1.53E-11	9.42329966	7.32459434	25.2644817	0.34327761
cg06836736	4210619	3.01E-11	9.42353824	7.19997378	21.1067052	0.30395584
cg08700306	4210619	1.96E-12	9.42937082	7.6982879	23.7257096	0.32925374
cg09350141	4210619	9.33E-15	9.44040667	8.63885625	29.1465165	0.37618189
cg23090046	6590228	1.52E-11	9.4479309	7.3255764	18.9751152	0.28191283
cg22030890	4210619	4.27E-13	9.44914775	7.97083476	25.2322587	0.34298995
cg20340242	6590228	6.53E-13	9.45338926	7.89553244	21.9143745	0.31195857
cg10275770	4760338	1.29E-11	9.45490482	7.35617227	18.1461447	0.27295859
cg01615704	4210619	2.26E-11	9.4676118	7.25256878	21.3748604	0.3066334
cg09671611	5270544	1.05E-10	9.49570253	6.9660569	23.3661872	0.32589043
cg15910079	5270544	5.62E-11	9.50470699	7.08376075	23.9788739	0.33160202
cg21842274	6180133	5.88E-13	9.50690014	7.91411712	22.9008801	0.32148709
cg15958424	1580411	5.50E-11	9.51061124	7.08798141	21.4685826	0.30756438
cg08840010	5270544	2.42E-14	9.51328545	8.47539625	32.0009838	0.39834762
cg00565688	4210619	4.93E-12	9.52169245	7.53163935	22.8303514	0.32081463
cg09169633	7320382	1.15E-10	9.53304145	6.94983447	22.2616982	0.3153437
cg03574571	6590228	7.57E-13	9.55559051	7.86911367	21.7732679	0.31057372
cg00565688	6020523	5.04E-11	9.55686218	7.10431737	17.7527585	0.26863078
cg23547429	4200541	2.84E-12	9.55985915	7.63140216	29.1343911	0.37608425
cg01526089	4210619	6.55E-14	9.56524334	8.30155336	27.1307445	0.35951867
cg08525145	5270544	9.75E-12	9.56712064	7.4072876	25.7158233	0.34728044
cg01410472	4210619	2.43E-11	9.57410231	7.23912112	21.3061119	0.3059489
cg21019522	6590228	6.25E-12	9.58674165	7.48851388	19.7929135	0.29053286
cg09106999	6180088	1.56E-13	9.58762544	8.1488816	22.6682335	0.31926385
cg15910079	4210619	1.20E-11	9.58937746	7.36996105	21.9804236	0.31260488
cg23889010	4760338	6.14E-12	9.59494047	7.49179837	18.8184984	0.28023805
cg01172972	4210619	2.17E-11	9.59775267	7.26067584	21.4163678	0.30704602
cg00135056	3850767	5.37E-11	9.59843353	7.09249412	16.8048265	0.25798743
cg06394229	5390730	3.59E-11	9.61268058	7.16705762	27.1072796	0.35931945
cg00666746	4210619	3.52E-11	9.61337993	7.1708842	20.9592305	0.30247446
cg19399532	6590228	2.07E-13	9.61573524	8.09928371	23.0185289	0.32260586
cg22854223	4210619	1.94E-11	9.61699841	7.28124263	21.5218758	0.30809264
cg02332525	4210619	1.72E-12	9.61917342	7.72183696	23.8538125	0.33044405
cg11254522	3990224	4.89E-11	9.62471323	7.10983073	23.6044637	0.32812325
cg15779716	6590228	5.62E-12	9.6266531	7.50778926	19.8908484	0.29155129
cg04600618	4210619	2.07E-13	9.63825571	8.09926033	25.960319	0.34942849
cg22045288	6180133	7.00E-12	9.64029329	7.46785024	20.5458402	0.29828813
cg15503752	3370327	8.10E-11	9.64081823	7.01565396	16.4130989	0.25349812
cg10126923	5390246	2.61E-12	9.64311481	7.64668675	33.0157927	0.40585307

cg26112639	6590228	2.76E-12	9.64844459	7.63658165	20.5516814	0.29834764
cg15013019	4210619	9.51E-12	9.65526504	7.41187393	22.1989849	0.31473494
cg07730301	6590228	3.13E-12	9.65850876	7.61399907	20.4349976	0.2971571
cg21991396	6590228	1.16E-12	9.66564556	7.79226816	21.3655134	0.30654042
cg09419900	5270544	4.79E-11	9.68200229	7.11382143	24.1369951	0.33306038
cg06849477	5390730	3.73E-11	9.6841195	7.16036409	27.0704362	0.3590064
cg09001777	5390730	3.63E-12	9.72332926	7.58692083	29.4871555	0.37891249
cg07914866	4210619	3.25E-12	9.72945735	7.60688561	23.2322032	0.32462837
cg04451770	6590228	2.23E-12	9.73472874	7.67480482	20.7499671	0.30036155
cg12506971	4210619	3.98E-11	9.73585606	7.14788791	20.8430694	0.30130317
cg16692277	6590228	3.16E-13	9.73646735	8.02460623	22.6105763	0.3187106
cg03608974	4210619	8.64E-11	9.74388506	7.00349033	20.1222008	0.29394557
cg10549973	7570408	3.31E-12	9.758295	7.60365589	19.9504805	0.29216998
cg04113075	4210619	3.87E-12	9.77063319	7.575301	23.0630395	0.32302817
cg11254522	5390730	7.98E-12	9.77361561	7.44395284	28.6615732	0.37225285
cg16967583	6590228	1.05E-13	9.77670317	8.21895848	23.6801757	0.32882963
cg12311132	4210619	5.20E-11	9.78675731	7.09842057	20.5944595	0.2987831
cg07525077	5270544	5.52E-11	9.79761239	7.08725634	23.9972265	0.33177161
cg13765621	5270544	2.27E-11	9.79918181	7.25169123	24.8707776	0.33974564
cg22933847	6180088	4.02E-11	9.80799563	7.14602528	17.5166621	0.26600855
cg13929328	4210619	4.18E-12	9.81365026	7.5617395	22.9906216	0.32234081
cg09358725	3840554	3.84E-11	9.82085488	7.15489361	18.2866503	0.27449197
cg20366831	3310091	7.60E-11	9.831436	7.02744013	17.5043352	0.26587113
cg09914304	4180544	1.01E-11	9.83426557	7.400376	19.9963723	0.29264537
cg25753817	6180088	5.53E-12	9.83832811	7.51064867	19.3115367	0.28548413
cg08510456	4760338	1.03E-10	9.8450976	6.96991458	16.2986121	0.25217579
cg02332525	6020523	6.61E-12	9.84724776	7.47832927	19.596707	0.28848367
cg21842274	3990379	7.44E-11	9.85722631	7.0315048	16.9900794	0.26009173
cg14564494	6590228	4.12E-11	9.86638427	7.1415284	18.0729979	0.27215775
cg15376097	5270544	6.78E-11	9.86763243	7.048815	23.7958985	0.32990645
cg14435807	6590228	1.01E-11	9.8701017	7.4011555	19.3522167	0.28591356
cg10106388	5270544	3.09E-11	9.8746325	7.19489391	24.56678	0.33699234
cg22088368	5270544	1.12E-10	9.88030649	6.95499161	23.3091167	0.32535343
cg11584690	4210619	3.48E-11	9.88567543	7.17320997	20.9709994	0.30259291
cg20713492	6590228	2.48E-11	9.88599106	7.23570875	18.5317584	0.27715147
cg27067618	5270544	3.36E-12	9.88811426	7.60108476	26.7934245	0.3566429
cg01410472	6020523	4.55E-11	9.90939201	7.12300331	17.842639	0.26962413
cg24921089	4210619	1.10E-12	9.91224308	7.8026285	24.2962786	0.33452304
cg21870884	4210619	3.64E-13	9.91671717	7.99921034	25.3921218	0.34441458
cg26215727	6590228	7.69E-11	9.9285605	7.02534127	17.5153216	0.26599361
cg17105014	6590228	2.00E-14	9.9327012	8.50942278	25.3264301	0.34382991
cg01182697	4210619	4.86E-11	9.93401767	7.11100887	20.6575612	0.29942446
cg27634151	4210619	3.61E-11	9.93551547	7.16618373	20.9354567	0.30223506
cg08107272	6020523	1.93E-11	9.93731763	7.28222301	18.6180713	0.27808336

cg00135056	4210619	8.54E-11	9.94173932	7.00552906	20.1322762	0.29404947
cg26701826	6590228	2.31E-11	9.94934861	7.24842008	18.5941367	0.27782518
cg09624565	4210619	8.73E-13	9.95083551	7.84364471	24.522672	0.33659095
cg21842274	4210619	1.89E-11	9.95111836	7.28556424	21.5440837	0.30831254
cg14859417	4210619	7.26E-11	9.96485867	7.03606746	20.2835488	0.29560581
cg16003913	4760338	1.12E-10	9.96647188	6.9551767	16.2300916	0.25138214
cg20083676	4210619	4.55E-12	9.9728125	7.5460959	22.9072464	0.32154773
cg17740645	4210619	2.22E-16	9.9728145	9.20632756	32.7189352	0.40367698
cg09419900	5390730	7.38E-11	9.97572092	7.03306174	26.3762739	0.35305063
cg10549973	6020523	4.35E-13	9.98202656	7.96787254	22.1531717	0.31428955
cg02266731	6590228	1.25E-12	9.98827841	7.77927656	21.2969733	0.30585781
cg09001777	6590228	2.41E-11	9.99178579	7.24104584	18.5579357	0.27743435
cg04353769	6590228	3.77E-15	10.0077421	8.80026611	27.0321143	0.35868047
cg06391468	4210619	2.23E-12	10.01091	7.67481812	23.5984273	0.32806687
cg00468146	4210619	1.68E-12	10.0176504	7.72589815	23.8759442	0.33064926
cg18239753	6020523	6.24E-11	10.0227082	7.06440734	17.5615797	0.26650888
cg19906550	6590228	1.19E-12	10.0555872	7.78864787	21.3464023	0.30635022
cg15125472	4210619	5.34E-12	10.0613032	7.51724066	22.7539107	0.3200843
cg14435807	1580411	6.18E-11	10.0680694	7.06611641	21.3579377	0.30646503
cg09076077	6590228	9.22E-13	10.0951819	7.83384055	21.5856063	0.30872331
cg10362591	6020523	6.84E-11	10.1021472	7.04727705	17.4798518	0.26559802
cg22820108	6590228	5.84E-11	10.106202	7.07684849	17.7614178	0.2687266
cg18818531	1740373	1.14E-10	10.1078594	6.95155596	17.588574	0.26680924
cg00513220	4210619	2.15E-11	10.1324661	7.26198732	21.4230868	0.30711276
cg23547429	5270544	9.35E-13	10.1354064	7.83125686	28.1094417	0.36771875
cg23713742	6590228	2.15E-11	10.1372779	7.26231143	18.6624309	0.27856136
cg21494776	4210619	5.77E-15	10.1418078	8.72220975	29.6570187	0.38026522
cg22933847	6180133	1.12E-10	10.154541	6.95469686	18.0067338	0.27143074
cg23889010	6590228	1.70E-11	10.1704082	7.30528916	18.874551	0.28083835
cg20008332	7570408	9.00E-11	10.1781439	6.9958517	16.9629087	0.25978384
cg16361890	6590228	1.12E-10	10.1877874	6.95547855	17.1843976	0.26228621
cg01623438	6590228	6.23E-12	10.2010354	7.4889696	19.795226	0.29055694
cg12388309	6020523	4.17E-11	10.2110374	7.13963893	17.9228559	0.2705084
cg22045288	5050347	2.75E-11	10.2145112	7.21675515	20.1401919	0.29413108
cg09971811	5390246	1.44E-11	10.2540713	7.33555507	31.1521199	0.39192228
cg23547429	5390730	4.58E-12	10.2567255	7.54479939	29.2422892	0.37695204
cg24881834	4210619	8.52E-11	10.2569303	7.00603844	20.1347941	0.29407543
cg08186362	6020523	4.12E-11	10.2659001	7.14165796	17.9326043	0.27061572
cg09106999	4210619	3.50E-11	10.2703788	7.17171742	20.9634464	0.30251689
cg16872071	4210619	1.69E-14	10.2778864	8.53880151	28.5402004	0.37126172
cg22854223	4760338	5.23E-12	10.28001	7.52102497	18.9649951	0.28180485
cg10599444	4210619	1.00E-17	10.2821229	9.66379291	35.7642368	0.42527075
cg20651453	4210619	7.16E-12	10.3158978	7.46367469	22.4708206	0.31736585
cg07684796	6020523	2.24E-11	10.3209865	7.25412697	18.4799923	0.27659142

cg26985289	4210619	1.07E-11	10.3258103	7.39091816	22.0895528	0.31367009
cg06394229	6590228	2.02E-11	10.3291036	7.2731582	18.715848	0.27913612
cg00333528	6590228	9.77E-13	10.3416573	7.82334843	21.5299483	0.30817259
cg08525145	5390730	8.73E-13	10.3558897	7.84349751	31.008166	0.39081901
cg01980222	5390246	7.48E-11	10.356167	7.03047766	29.3998721	0.3782151
cg17753124	6180133	1.66E-12	10.3665031	7.72778625	21.9009581	0.31182714
cg16749578	4210619	9.56E-11	10.37135	6.98456149	20.028794	0.29298085
cg02656594	6590228	1.09E-12	10.3729968	7.80382852	21.4265988	0.30714764
cg27654142	7570408	3.33E-11	10.38995	7.18096436	17.846459	0.26966629
cg18638581	6590228	4.44E-15	10.3971423	8.76743334	26.8366926	0.35701322
cg17685628	4210619	3.75E-11	10.4031918	7.15910186	20.8996678	0.30187436
cg23181133	5390730	2.72E-12	10.418256	7.63949361	29.7946914	0.38135729
cg16465939	4210619	1.10E-10	10.4227039	6.95727588	19.8945944	0.29159019
cg17166812	5390730	2.47E-11	10.4254732	7.23613345	27.4895073	0.36254916
cg21504918	4210619	1.07E-10	10.4401473	6.96303112	19.9228568	0.29188352
cg23001650	5090739	2.22E-16	10.4416935	9.32059841	30.5983805	0.38765636
cg10059959	6020523	2.80E-11	10.4678155	7.21304513	18.279055	0.27440924
cg22165175	4210619	3.28E-13	10.4726481	8.01769205	25.4965501	0.34534187
cg00739120	4210619	1.03E-10	10.4772963	6.97138153	19.963905	0.29230911
cg17749456	6590228	4.26E-13	10.5008695	7.97164932	22.3235701	0.31594323
cg10787197	5270544	8.75E-12	10.5124431	7.42710026	25.8247137	0.34823886
cg10106388	6330091	9.76E-11	10.5200526	6.98056149	18.2526298	0.27412129
cg25725843	4210619	3.82E-12	10.5264178	7.57771439	23.0759405	0.32315047
cg22854223	6590228	7.68E-11	10.5571182	7.02548911	17.5160254	0.26600146
cg20716064	7570408	9.61E-11	10.5694511	6.98355509	16.9050338	0.25912718
cg10148841	6590228	9.92E-11	10.5715404	6.97756532	17.2886601	0.26345832
cg13853198	4210619	8.78E-11	10.598121	7.00053282	20.1075899	0.29379484
cg22334665	6020523	6.32E-11	10.5991648	7.06187961	17.5495075	0.26637448
cg17166812	5270544	1.04E-12	10.6014077	7.81222429	27.9991334	0.36680504
cg23547429	1030296	1.08E-10	10.6024989	6.96111803	30.3513954	0.38573426
cg25623459	4200541	1.82E-11	10.6039194	7.29275404	27.2079591	0.36017333
cg13471990	5270544	2.70E-13	10.6055051	8.05240408	29.4108367	0.37830279
cg17952262	4210619	1.02E-10	10.6135342	6.97231118	19.9684779	0.29235649
cg22016649	4760338	2.20E-11	10.6182544	7.25762366	17.6653043	0.26766165
cg17753124	6180088	6.66E-15	10.6397254	8.70012971	25.7876668	0.3479131
cg10787197	5390730	3.61E-11	10.6415131	7.16607055	27.1018443	0.35927329
cg24975222	6020523	4.47E-11	10.6508449	7.12638518	17.8589311	0.2698039
cg02525756	4210619	6.21E-11	10.6549578	7.06533045	20.429121	0.29709703
cg00386408	4210619	5.37E-12	10.6596462	7.5160783	22.7477463	0.32002534
cg25432696	5910112	7.70E-11	10.6633164	7.02492385	17.0479722	0.26074689
cg23181133	5270544	4.13E-14	10.6925015	8.38233338	31.4197477	0.39396281
cg16483916	4210619	1.72E-12	10.6992235	7.72188928	23.8540975	0.33044669
cg22747092	4210619	2.85E-12	10.7070463	7.63066173	23.3600099	0.32583234
cg09106999	4760338	3.78E-11	10.7076393	7.15757888	17.1837973	0.26227946

cg03014628	7570408	1.08E-10	10.7149623	6.96098459	16.7990689	0.25792184
cg01830294	4210619	1.90E-13	10.7299397	8.11416682	26.0455795	0.35017424
cg24474182	6590228	1.08E-13	10.7393373	8.21478783	23.6569542	0.32861314
cg15958424	6590228	2.22E-16	10.7857591	9.20089844	29.4755271	0.37881967
cg13765621	6590228	2.24E-11	10.827022	7.25464868	18.6247423	0.27815528
cg02225847	4210619	7.24E-13	10.8335973	7.87706772	24.7080317	0.33827451
cg22436229	4210619	8.57E-12	10.8446323	7.43085643	22.2983799	0.31569926
cg23001650	1850546	2.23E-11	10.8631061	7.25525905	22.8216711	0.32073178
cg26191951	6590228	1.23E-11	10.8680764	7.36511465	19.1719083	0.28400622
cg21019522	7550358	1.14E-11	10.8752137	7.37866639	33.4169533	0.40876864
cg02590345	4210619	4.43E-11	10.8902318	7.12821783	20.7440059	0.30030117
cg26200580	6020523	7.07E-11	10.8928813	7.04113253	17.4505848	0.26527129
cg03554552	2970408	6.47E-12	10.8997325	7.48205668	22.4973553	0.31762158
cg22681784	4210619	7.39E-14	10.9168758	8.28042914	27.0071741	0.35846817
cg10057295	5390246	3.19E-11	10.9332668	7.18921086	30.3022875	0.38535065
cg26128092	6020523	1.00E-10	10.9363467	6.97514708	17.1378992	0.26176228
cg04925864	4210619	8.76E-12	10.9454328	7.42677989	22.2770131	0.31549219
cg15361750	5390246	5.39E-13	10.9502424	7.92958215	34.777517	0.41844737
cg13488201	6020523	6.42E-11	10.9662403	7.05892412	17.5353979	0.26621733
cg06379754	4210619	1.19E-11	10.98732	7.37071272	21.9843323	0.31264309
cg15958424	5390246	8.28E-11	10.9877777	7.01142838	29.2929289	0.37735849
cg07525077	6590228	2.22E-11	11.0118307	7.25614534	18.6321004	0.2782346
cg07028533	7570408	2.80E-12	11.0132251	7.63430586	20.1077243	0.29379622
cg08145177	6020523	9.14E-11	11.0290146	6.99285198	17.2215085	0.26270384
cg19014419	4210619	2.15E-12	11.0340495	7.68170434	23.6357326	0.32841516
cg19005210	4210619	1.63E-13	11.0439193	8.14101871	26.1995595	0.35151674
cg16003913	4210619	1.55E-14	11.0508879	8.55203606	28.6199944	0.37191367
cg07073964	5270544	1.11E-11	11.0811128	7.38392249	25.5877824	0.34614984
cg10061138	4210619	4.96E-13	11.085307	7.94419579	25.0826964	0.3416515
cg15322932	5390730	1.13E-11	11.0968239	7.37977803	28.2960967	0.36925887
cg05989054	4210619	1.33E-14	11.1150965	8.57993525	28.7886094	0.37328688
cg14764661	5910112	1.08E-10	11.1467147	6.96220086	16.7528341	0.25739469
cg09671611	5390730	8.06E-14	11.1513556	8.26542971	33.619405	0.41022918
cg12792367	3990224	3.50E-11	11.1515487	7.17211518	23.9317042	0.33116573
cg17753124	4760338	9.78E-11	11.1614675	6.98029883	16.3469785	0.252735
cg12311132	6590228	2.81E-11	11.1624341	7.21290857	18.4201454	0.27594285
cg02994956	4210619	4.64E-11	11.1677241	7.11940026	20.6996871	0.29985197
cg03000846	4210619	2.93E-14	11.171675	8.44285001	27.965384	0.36652496
cg12792367	5390730	1.15E-11	11.1777022	7.37696111	28.2801268	0.36912739
cg21578541	4210619	6.73E-14	11.1834617	8.29733854	27.1060639	0.35930913
cg15910079	6590228	1.64E-12	11.1853389	7.73008249	21.0384755	0.30327126
cg08965235	3190379	3.29E-11	11.1968154	7.18341403	18.6677652	0.27861879
cg06196379	5390246	1.83E-11	11.1973934	7.29142431	30.8940457	0.38994153
cg24713204	2680189	1.98E-12	11.2438893	7.69648362	25.1748719	0.34247703

cg14088811	4210619	8.88E-16	11.2540483	9.0514269	31.7212396	0.39624519
cg10210238	6020523	2.57E-11	11.2567347	7.22880908	18.3560239	0.27524668
cg14611112	5270544	7.46E-11	11.2615734	7.03092202	23.7025613	0.3290382
cg12262564	2970408	7.31E-11	11.2767336	7.03469616	20.2086323	0.2948359
cg22933847	4210619	1.07E-10	11.2871118	6.96279838	19.9217134	0.29187166
cg01525376	6580750	6.94E-11	11.2926022	7.04457624	20.5800363	0.29863634
cg13703437	6590228	5.26E-14	11.3391164	8.34041158	24.3615766	0.3351208
cg10694152	4210619	1.01E-11	11.3424086	7.40048173	22.1394558	0.31415609
cg17386185	4200541	1.02E-10	11.3468193	6.97254188	25.4668184	0.34507813
cg09076123	5390246	1.42E-13	11.3536342	8.16564354	36.2965621	0.42888582
cg03014628	6020523	2.68E-12	11.3543256	7.64214782	20.4341506	0.29714844
cg09313705	4210619	4.90E-11	11.3558598	7.10944277	20.6497047	0.29934467
cg26163537	4210619	5.59E-11	11.3754587	7.08496963	20.5271571	0.29809775
cg00344372	5090739	1.21E-11	11.3836989	7.3684254	19.5079207	0.28755248
cg03763616	1580411	2.15E-11	11.3920385	7.26195391	22.3611279	0.31630665
cg00234616	4210619	1.45E-11	11.3970251	7.33435242	21.7957106	0.31079435
cg26335299	4210619	6.69E-12	11.4064759	7.47612139	22.5364194	0.31799772
cg24821554	5390246	4.42E-12	11.4075974	7.55141201	32.4368765	0.40159456
cg20322876	6020523	3.46E-11	11.4204761	7.17412119	18.0897252	0.27234105
cg22045288	4210619	1.26E-13	11.4208176	8.18649606	26.461506	0.35378786
cg02919422	4210619	1.37E-12	11.4385793	7.76328418	24.0802287	0.33253755
cg05615150	5420564	4.01E-11	11.4416275	7.1468968	20.7789142	0.30065459
cg10981541	4210619	1.48E-11	11.4424853	7.33032358	21.7748681	0.31058946
cg22016649	4210619	1.09E-14	11.4646586	8.61655744	29.0107775	0.37508709
cg09619786	5910112	5.81E-11	11.5022086	7.0776783	17.2982535	0.26356598
cg27603796	4210619	2.67E-12	11.5040817	7.64248033	23.4236882	0.32643061
cg27223047	4210619	2.42E-14	11.5358579	8.47632832	28.1652047	0.36817965
cg18787975	5390730	9.16E-12	11.5477206	7.41876947	28.5177763	0.37107826
cg18881723	610154	4.34E-11	11.5739183	7.1320197	24.0306778	0.33208051
cg08145177	5910112	7.21E-13	11.575426	7.87778912	21.3237567	0.30612471
cg09037813	3170128	7.62E-11	11.5801086	7.02700221	19.5595231	0.28809398
cg04988978	5390246	6.55E-12	11.580226	7.47988822	32.0070476	0.39839303
cg21842274	4760338	1.84E-14	11.5950064	8.52243317	24.3290909	0.33482355
cg08655844	4210619	1.41E-11	11.6019459	7.339793	21.8238746	0.31107103
cg14859417	6590228	1.20E-11	11.620735	7.36872898	19.1899507	0.28419753
cg09106999	6590228	2.83E-11	11.6231559	7.21109483	18.4112818	0.2758467
cg13471990	6590228	4.48E-13	11.6235459	7.96247523	22.2740432	0.3154634
cg15407570	4210619	2.00E-15	11.6251611	8.91844592	30.8782311	0.38981974
cg25753817	5390730	8.69E-11	11.6265096	7.0022804	26.2102969	0.35161015
cg25374813	5270544	1.27E-11	11.6433115	7.35926626	25.4531049	0.34495641
cg04809787	4210619	4.21E-12	11.6442031	7.56016979	22.9822477	0.32226124
cg08840010	5390246	1.60E-11	11.6527065	7.31632621	31.0394792	0.39105933
cg12582959	3170128	3.88E-11	11.699554	7.15261914	20.181663	0.29455833
cg18972811	6020523	1.00E-10	11.7102724	6.97565457	17.1402928	0.26178927

cg23001650	6180088	6.55E-12	11.7121495	7.47986791	19.1565658	0.28384345
cg22016649	6590228	6.23E-12	11.7275259	7.48889349	19.7948398	0.29055292
cg09619786	4210619	1.33E-11	11.7289038	7.35110828	21.8825169	0.3116464
cg22045288	4760338	3.29E-13	11.754411	8.0174899	21.540637	0.30827842
cg27444994	7570408	1.91E-11	11.7633076	7.28341515	18.3453798	0.27513098
cg15433631	2970408	5.84E-12	11.7872555	7.5008272	22.59647	0.31857511
cg10787197	6590228	3.25E-12	11.8133986	7.60705634	20.3991941	0.29679098
cg22598563	4210619	1.06E-10	11.8213597	6.96590602	19.9369835	0.29203004
cg10549973	3190379	5.18E-11	11.8294981	7.09916726	18.2576978	0.27417653
cg16604516	4210619	2.74E-11	11.8483462	7.21748376	21.195763	0.30484738
cg11884546	7150017	8.32E-12	11.8716907	7.43634688	30.4735602	0.38668648
cg08965235	2970408	3.48E-12	11.8921324	7.59475525	23.0961721	0.32334218
cg14603345	2970408	1.50E-12	11.9009743	7.74711567	23.9199623	0.33105704
cg25027167	4210619	1.66E-11	11.9224054	7.30989366	21.6693537	0.30955031
cg25374813	5390730	2.74E-11	11.9276391	7.21739099	27.3854345	0.36167301
cg13030582	4210619	6.20E-12	11.9316702	7.49003418	22.6098746	0.31870387
cg16772514	4210619	5.26E-12	11.9347845	7.51992976	22.7681757	0.32022071
cg10061138	6590228	7.50E-12	11.9439683	7.45529278	19.6247137	0.28877689
cg25753817	5270544	2.45E-12	11.9441352	7.65788448	27.1145305	0.35938102
cg00401678	1770168	2.57E-11	11.9539978	7.22896429	28.1560459	0.36810399
cg25623459	5390730	1.50E-12	11.9789904	7.74614349	30.425087	0.38630901
cg09106999	1580411	9.44E-11	11.982676	6.98688537	20.9598634	0.30248083
cg08965235	2680189	9.61E-14	11.991192	8.23438514	28.259097	0.36895418
cg19769182	4210619	2.55E-14	12.027045	8.46633171	28.1054555	0.36768578
cg19843036	3170128	3.79E-13	12.0407146	7.99233235	24.6236179	0.33750887
cg02590345	6020523	2.11E-12	12.0472396	7.68542795	20.6584299	0.29943328
cg25432696	4210619	2.82E-14	12.0535858	8.44961158	28.0056778	0.36685932
cg26616347	4210619	4.04E-12	12.0840311	7.56758866	23.0218399	0.32263729
cg21494776	2970408	4.61E-11	12.1062809	7.12070599	20.6377105	0.29922282
cg15439862	2970408	2.66E-12	12.1285651	7.64326903	23.3567034	0.32580125
cg24715735	4210619	2.84E-11	12.1380341	7.21049356	21.1601841	0.30449148
cg20483374	4210619	4.97E-13	12.1414406	7.94403535	25.0817971	0.34164343
cg13434842	3170128	4.61E-12	12.2409039	7.54364079	22.1888272	0.31463624
cg25806808	2970408	1.35E-11	12.248915	7.347335	21.7932674	0.31077034
cg14726637	3170128	5.69E-11	12.2591256	7.08147303	19.8279458	0.2908975
cg02626929	5390246	3.70E-11	12.2629471	7.16148017	30.1431831	0.3841045
cg21842274	6590228	7.66E-14	12.2714333	8.27467138	23.9915067	0.33171877
cg19843036	670369	1.37E-12	12.276712	7.76277626	25.2242365	0.3429183
cg25882366	670369	9.37E-11	12.3280922	6.98833336	21.1303512	0.30419278
cg12582959	670369	1.97E-11	12.3321936	7.27855681	22.6141786	0.3187452
cg01366419	2970408	4.12E-11	12.3421043	7.14172655	20.7433689	0.30029472
cg25623459	1030296	4.82E-11	12.3491758	7.1126163	31.2186021	0.39243045
cg18881723	4670193	7.31E-11	12.363487	7.03479479	26.1992145	0.35151374
cg17977409	2970408	9.91E-11	12.3692	6.97768324	19.9270851	0.29192738

cg05670348	2680189	8.47E-11	12.4210278	7.00714889	21.5268401	0.30814181
cg16008138	540368	1.56E-12	12.4228512	7.739982	21.64858	0.30934536
cg13434842	670369	1.80E-11	12.4316972	7.29441406	22.6969915	0.31953946
cg18765542	7570408	1.47E-12	12.4333729	7.75069427	20.7105943	0.29996257
cg06810647	3170128	2.75E-11	12.4623416	7.21651131	20.5023294	0.29784459
cg22045288	6590228	8.03E-13	12.5136293	7.85854459	21.7169494	0.31001944
cg07846167	4210619	7.48E-12	12.5528571	7.4558036	22.4293934	0.31696621
cg17753124	5270544	1.71E-14	12.5790192	8.5356905	32.380985	0.40118018
cg02868338	5270544	6.06E-11	12.6026569	7.06966658	23.9049681	0.33091819
cg16741710	4210619	5.52E-11	12.6097485	7.08718057	20.5382109	0.2982104
cg09313705	6590228	9.81E-11	12.6251986	6.97954541	17.2980235	0.2635634
cg00565688	2970408	3.66E-11	12.6297923	7.16385492	20.8549321	0.30142297
cg15755084	4210619	5.45E-11	12.6410004	7.08958571	20.5502395	0.29833295
cg27067618	5390246	8.69E-11	12.6435058	7.00239137	29.2422963	0.3769521
cg15526708	4210619	2.18E-11	12.662635	7.25917965	21.4087039	0.30696987
cg08872742	5270544	5.35E-12	12.6901809	7.51671304	26.3208585	0.3525704
cg17386185	5390730	1.60E-11	12.7186122	7.31630656	27.9377384	0.36629534
cg23547429	5390246	5.62E-11	12.7982707	7.08381618	29.7008598	0.3806134
cg06810647	670369	4.76E-11	12.8334593	7.11486981	21.7698725	0.31054033
cg16330965	5390246	5.69E-11	12.8706801	7.08153527	29.6879421	0.38051085
cg01410472	2970408	7.50E-11	12.8884824	7.02998494	20.18528	0.29459557
cg00775197	4210619	1.27E-11	12.9259916	7.35860883	21.9214389	0.31202776
cg05595345	3170128	3.36E-11	12.9505787	7.17925632	20.3150049	0.29592858
cg22598028	3170128	2.12E-11	12.9534389	7.26474314	20.7462874	0.30032428
cg18881723	5670605	8.01E-11	12.9603441	7.01762888	16.4412783	0.25382288
cg08145177	4210619	1.00E-17	12.9619248	9.47058231	34.4600414	0.41621738
cg11668923	3170128	5.64E-11	12.9664431	7.08335299	19.8372469	0.29099425
cg10303487	2970408	5.38E-11	12.9857309	7.09182806	20.4930654	0.29775008
cg08525145	5390246	9.81E-12	12.9882379	7.40625824	31.5688342	0.39509359
cg03544320	3170128	1.07E-10	12.9885159	6.96264216	19.2450392	0.28478104
cg00775197	5910112	1.50E-11	12.9952788	7.32806673	18.5116982	0.27693454
cg21096399	4210619	8.88E-16	13.0253195	9.03415987	31.6110736	0.3954132
cg16158874	4200541	1.55E-12	13.0633564	7.74041534	29.7731243	0.38118646
cg22933847	6590228	2.56E-11	13.0824863	7.22962147	18.501925	0.27682881
cg00401678	6420392	4.37E-11	13.1130401	7.13062975	28.4901303	0.37085194
cg17757055	4210619	8.07E-12	13.1608183	7.44178378	22.3557123	0.31625427
cg20557104	4210619	3.76E-12	13.1896513	7.58085332	23.0927261	0.32330954
cg16638540	670369	5.68E-11	13.2367521	7.08198249	21.6025547	0.30889083
cg10106388	5390246	4.96E-11	13.2860156	7.10716374	29.8333252	0.38166305
cg16028753	4210619	5.06E-14	13.3199221	8.34706941	27.3980699	0.36177951
cg09300114	6020523	1.94E-11	13.343605	7.28084477	18.6112854	0.27801018
cg27654142	2970408	5.92E-11	13.3568127	7.07409474	20.4045332	0.2968456
cg23001650	4200541	9.77E-12	13.3756579	7.40694239	27.8477608	0.36554687
cg21578541	3190379	8.27E-11	13.3768996	7.01161119	17.836649	0.26955801

cg14667273	4210619	5.44E-12	13.3948904	7.51378196	22.7355708	0.31990884
cg20430816	3170128	1.10E-10	13.4562025	6.95730369	19.2190837	0.28450623
cg25250358	3170128	1.21E-11	13.4788256	7.36845094	21.2763484	0.30565214
cg12978308	670369	1.05E-10	13.5106463	6.96673373	21.0223323	0.30310908
cg05595345	670369	2.90E-11	13.5148959	7.20689916	22.2422014	0.31515456
cg03544320	670369	1.06E-10	13.5195408	6.96497325	21.0135429	0.30302076
cg10059959	2970408	5.35E-11	13.5790485	7.09306223	20.4992352	0.29781302
cg18881723	2450427	4.52E-13	13.5822334	7.96082967	26.5167772	0.35426504
cg17753124	6590228	8.26E-14	13.5920055	8.26114813	23.9157437	0.33101798
cg10787197	5390246	9.55E-11	13.6418036	6.98464655	29.1430656	0.37615411
cg13929328	2970408	1.97E-12	13.6735674	7.69773578	23.6511804	0.32855929
cg12594641	3170128	1.84E-12	13.6926349	7.71025833	23.076549	0.32315624
cg08186362	2970408	1.68E-11	13.7129373	7.30759798	21.5880353	0.30874732
cg22598028	670369	6.81E-12	13.759598	7.47291529	23.641628	0.32847017
cg20182358	2970408	6.82E-12	13.8051358	7.47255713	22.4472891	0.31713891
cg23771603	670369	8.70E-11	13.8296132	7.00209838	21.199364	0.30488338
cg08675664	3170128	1.03E-11	13.8440922	7.39772329	21.4273219	0.30715483
cg19616230	2970408	4.32E-11	13.8485152	7.13274198	20.6981704	0.29983659
cg04856043	4210619	1.01E-11	13.8545099	7.40077985	22.1410124	0.31417124
cg13265789	3170128	1.04E-10	13.870836	6.96795219	19.2708762	0.28505438
cg23756272	6590228	5.86E-11	13.8736725	7.0759488	17.7571038	0.26867887
cg09082287	3170128	7.58E-13	13.8749925	7.86888201	23.9396999	0.33123973
cg25044651	2970408	2.08E-11	13.9159272	7.26837893	21.3865697	0.30674985
cg23001650	5270544	9.29E-12	13.93614	7.41606946	25.7640526	0.34770528
cg26609631	3170128	8.08E-14	13.9508628	8.26510566	26.1725388	0.35128156
cg04675937	3170128	6.42E-11	13.9698757	7.05889519	19.7164346	0.28973552
cg09688546	3170128	9.51E-13	13.9776637	7.82827453	23.7170604	0.32917322
cg12594641	670369	5.34E-12	13.9929676	7.51713026	23.8791445	0.33067893
cg00027083	3170128	3.11E-14	13.9944854	8.43223276	27.1472354	0.3596586
cg11668923	670369	7.08E-12	14.0232359	7.46581351	23.6036089	0.32811527
cg09671611	5390246	9.52E-13	14.0329868	7.8280733	34.1380117	0.41393786
cg19713460	670369	4.29E-11	14.0650808	7.13407431	21.8679359	0.31150343
cg08185241	3170128	4.23E-13	14.0786252	7.97292196	24.5153785	0.33652453
cg08899626	3170128	8.13E-12	14.0815605	7.44050044	21.6490236	0.30934974
cg10300684	7570408	8.74E-13	14.0947392	7.84323167	21.1964297	0.30485405
cg10599444	3170128	2.64E-12	14.1165816	7.64482384	22.7256079	0.31981349
cg14473924	3170128	1.90E-13	14.1245112	8.11431433	25.309858	0.34368225
cg26233914	5390246	8.45E-12	14.1365284	7.43342597	31.73002	0.3963114
cg23615676	3170128	8.67E-12	14.1891677	7.42872727	21.5878792	0.30874578
cg09300114	4210619	1.95E-14	14.2118461	8.51189288	28.3783436	0.36993512
cg18765542	2680189	1.12E-10	14.2334756	6.95554527	21.2675099	0.30556397
cg25250358	670369	4.43E-12	14.2766194	7.55097585	24.0619051	0.33236861
cg23001650	3990224	4.91E-11	14.2982051	7.10895775	23.5998974	0.3280806
cg08899626	670369	2.83E-11	14.3224862	7.2111274	22.2640482	0.31536649

cg26202340	3170128	1.58E-11	14.3304288	7.31856465	21.0204368	0.30309004
cg05125838	5390246	7.29E-11	14.3345963	7.03533706	29.4271994	0.37843361
cg00027083	670369	8.44E-14	14.3585473	8.25760669	28.0651033	0.3673518
cg02332525	2970408	6.22E-15	14.3703938	8.71266648	29.5209588	0.37918216
cg04600618	3170128	1.45E-11	14.4032176	7.33463396	21.1026809	0.3039155
cg18787975	5390246	1.05E-10	14.4044085	6.96627219	29.0405798	0.37532779
cg14473924	670369	6.79E-13	14.4130427	7.88829708	25.9282508	0.34914756
cg08432727	2970408	3.21E-11	14.5006577	7.18809163	20.9775208	0.30265852
cg09082287	670369	5.49E-13	14.5122323	7.92630536	26.1436569	0.35102999
cg03554552	3170128	6.66E-16	14.5240318	9.11257469	31.3164199	0.39317661
cg13488201	2970408	4.00E-11	14.5362617	7.14703729	20.7701122	0.30056551
cg16232126	3170128	2.54E-12	14.5512076	7.65137845	22.7606271	0.32014853
cg15250507	3170128	2.40E-14	14.5573816	8.47707769	27.4120942	0.36189768
cg02497700	3170128	3.36E-12	14.5617709	7.60123652	22.4934984	0.31758442
cg04675937	670369	4.79E-11	14.621019	7.11362947	21.763548	0.31047812
cg02265318	2970408	9.88E-14	14.6212828	8.22974298	26.6374611	0.35530451
cg09688546	670369	6.61E-13	14.6300523	7.89326766	25.9563619	0.34939384
cg08675664	670369	3.91E-12	14.6530891	7.57386844	24.1859867	0.33351094
cg10300684	6020523	3.22E-14	14.654707	8.42678608	24.6968388	0.33817309
cg13265789	670369	4.80E-11	14.6579304	7.1131832	21.7612728	0.31045574
cg26609631	670369	3.73E-14	14.6773676	8.39997799	28.9149802	0.37431212
cg16483916	2970408	1.92E-12	14.6919447	7.70184584	23.6734865	0.32876728
cg26055770	3170128	3.53E-14	14.7075838	8.41060081	27.0199771	0.35857717
cg18765542	3190379	8.88E-11	14.7108933	6.99831794	17.7731799	0.26885671
cg15731815	2970408	9.58E-11	14.7117234	6.984015	19.9582402	0.29225041
cg10065825	3170128	5.25E-12	14.7285421	7.52023738	22.0656895	0.31343744
cg04598121	670369	7.71E-11	14.7484135	7.02486818	21.3138216	0.30602573
cg13449778	2970408	3.67E-11	14.7551751	7.16299778	20.8506043	0.30137927
cg08185241	670369	2.58E-13	14.7614093	8.060142	26.9103977	0.35764305
cg12563178	3170128	1.38E-11	14.7988738	7.34351799	21.1482276	0.3043718
cg18881723	1440750	5.77E-15	14.8076616	8.72761311	30.0227391	0.38315779
cg17252960	670369	6.03E-11	14.8076745	7.07078316	21.5457538	0.30832907
cg02497700	670369	1.23E-11	14.8107277	7.36408532	23.0629777	0.32302758
cg19884262	3170128	4.74E-11	14.8545081	7.11563733	19.9973592	0.29265559
cg04600618	670369	2.02E-11	14.8970855	7.27362888	22.5884798	0.31849834
cg08145177	2970408	2.04E-11	14.9114811	7.27201547	21.4052047	0.30693509
cg10599444	670369	1.06E-12	14.9118125	7.8082539	25.4780045	0.34517738
cg24713204	3170128	6.44E-15	14.9630552	8.70898607	28.8041868	0.37341344
cg25993718	3170128	4.16E-11	14.9763483	7.13979337	20.1176358	0.29389848
cg23037403	3170128	2.14E-12	15.0250248	7.68251875	22.9274097	0.32173969
cg27096144	3170128	6.29E-13	15.0722324	7.90199189	24.1220848	0.33292313
cg24396745	3170128	2.96E-11	15.0775715	7.20327394	20.4356586	0.29716385
cg25720804	670369	1.20E-11	15.0866956	7.36877006	23.0877117	0.32326203
cg12163490	3170128	3.45E-13	15.111319	8.00912067	24.717448	0.3383598

cg02501779	3170128	2.27E-13	15.133744	8.0828924	25.1320922	0.34209415
cg20083676	3170128	8.47E-11	15.1440817	7.00711802	19.4620537	0.28707047
cg26202340	670369	6.45E-12	15.1545117	7.4826418	23.6937572	0.32895619
cg07846220	3170128	4.02E-13	15.1618288	7.9815859	24.5636592	0.33696396
cg23001650	5390730	5.17E-13	15.197431	7.9368432	31.5740768	0.39513328
cg25806808	3170128	3.10E-13	15.202805	8.02802718	24.8233523	0.33931762
cg27096144	670369	3.83E-12	15.2258668	7.57747377	24.2055624	0.3336908
cg21481775	3170128	1.14E-11	15.2389212	7.37871101	21.3291971	0.3061789
cg16232126	670369	1.62E-12	15.2569352	7.73304974	25.0591618	0.34144039
cg03554552	670369	2.22E-16	15.2619534	9.25401781	34.3180766	0.41521465
cg01366419	3170128	1.30E-12	15.291257	7.77245491	23.412897	0.3263293
cg10694152	2970408	2.88E-11	15.3063781	7.20815051	21.0792913	0.30368094
cg02624705	3170128	3.09E-12	15.310242	7.61614726	22.5727511	0.31834717
cg08390209	3170128	4.04E-11	15.3296774	7.14534716	20.1453466	0.29418421
cg05472874	3170128	1.77E-11	15.3346883	7.29797274	20.9153093	0.30203205
cg09313439	2970408	5.37E-12	15.3395439	7.516163	22.6776325	0.31935395
cg12262564	3170128	3.33E-15	15.3891858	8.81488184	29.4523493	0.37863458
cg10065825	670369	3.65E-12	15.4229921	7.58604331	24.2521294	0.33411827
cg10362591	3170128	7.36E-11	15.4403972	7.03354089	19.591635	0.28843054
cg09350141	3170128	1.33E-15	15.5145952	8.97314478	30.4356407	0.38639123
cg21870884	3170128	1.56E-12	15.5363437	7.74001158	23.2371121	0.32467469
cg10238818	3170128	1.33E-13	15.5475934	8.17775689	25.6708788	0.34688402
cg00333226	2970408	2.30E-11	15.5483674	7.24930431	21.2889765	0.30577808
cg01615704	3170128	5.73E-12	15.5603488	7.50428316	21.9819652	0.31261995
cg10737521	3170128	5.30E-11	15.5661993	7.09481443	19.8940059	0.29158408
cg21481775	670369	3.04E-11	15.5729326	7.19809663	22.196761	0.31471333
cg11260422	2970408	7.09E-11	15.5770112	7.04051234	20.2374832	0.2951326
cg02501779	670369	4.26E-13	15.5979431	7.97150171	26.401148	0.35326596
cg15433631	3170128	8.88E-16	15.5982268	9.0396824	30.854265	0.38963507
cg09339527	3170128	1.11E-13	15.6216297	8.20934208	25.8516632	0.34847563
cg02613386	3170128	6.77E-14	15.6226896	8.29634869	26.3532675	0.35285134
cg06675478	670369	6.84E-11	15.624817	7.04711253	21.4259967	0.30714166
cg15250507	670369	2.22E-15	15.6317847	8.89129011	31.9594096	0.39803609
cg04720330	3170128	1.12E-10	15.6424344	6.95434809	19.2047222	0.28435409
cg17051440	3830349	6.04E-11	15.6507703	7.07031099	20.0139008	0.29282678
cg09083627	3170128	7.46E-11	15.6677096	7.03088821	19.5786039	0.288294
cg03355526	3170128	7.21E-12	15.6718363	7.46239495	21.7629912	0.31047264
cg01610488	670369	6.50E-11	15.691195	7.05659086	21.4739023	0.30761714
cg10453365	3170128	1.79E-11	15.748866	7.29566394	20.9035406	0.30191341
cg10189695	3170128	1.81E-11	15.7728915	7.29353446	20.8926894	0.30180398
cg14795968	670369	2.62E-11	15.7948757	7.22543831	22.3380859	0.31608373
cg23850212	3170128	6.54E-11	15.8173306	7.05559665	19.700173	0.28956575
cg03355526	670369	3.77E-11	15.8199142	7.15817236	21.9913612	0.31271179
cg20723355	3170128	1.09E-14	15.8238876	8.61445041	28.2321751	0.36873229

cg12563178	670369	2.76E-12	15.8408111	7.63659522	24.5279008	0.33663856
cg06836736	3170128	1.86E-12	15.8724574	7.70830151	23.0660107	0.32305634
cg10238818	670369	4.47E-13	15.8845811	7.96285882	26.3517948	0.35283858
cg24713204	670369	1.33E-15	15.8856523	8.98147382	32.5370285	0.40233564
cg11398680	3170128	5.02E-13	15.8931864	7.94238129	24.3456055	0.3349747
cg24693053	3170128	3.93E-13	15.8962504	7.98563727	24.586254	0.33716941
cg07846220	670369	2.22E-13	15.9214635	8.08686313	27.0650187	0.35896035
cg21902544	670369	9.54E-11	15.929011	6.98487214	21.1130193	0.30401912
cg15057581	3170128	4.64E-12	15.9308168	7.54256475	22.1831572	0.31458113
cg11398680	670369	4.77E-12	15.9374848	7.53744297	23.9887313	0.33169312
cg26320696	3170128	5.72E-12	15.9439212	7.50445991	21.9828917	0.31262901
cg02624705	670369	2.90E-12	15.9491909	7.62753163	24.4783222	0.33618687
cg25993718	670369	1.04E-11	15.9932451	7.39632116	23.2334912	0.32464052
cg19884262	670369	7.03E-12	16.012778	7.46692106	23.6095357	0.32817062
cg06187947	3170128	2.69E-14	16.0142948	8.45734097	27.295354	0.3609127
cg10644361	3170128	8.63E-13	16.0231921	7.84560514	23.8119384	0.33005543
cg17733100	3170128	7.55E-15	16.0302869	8.67717323	28.6109983	0.37184023
cg14155416	3170128	3.33E-14	16.0477355	8.42027385	27.076842	0.35906086
cg09339527	670369	2.58E-13	16.0529081	8.06023719	26.9109476	0.35764775
cg24396745	670369	8.11E-12	16.0663446	7.44092552	23.4706571	0.32687121
cg26055770	670369	8.88E-16	16.0668977	9.0575635	33.0289102	0.40594886
cg22165175	670369	4.22E-11	16.074242	7.13698201	21.8828065	0.31164924
cg06391468	3170128	1.99E-12	16.0814163	7.69586571	22.9991013	0.32242137
cg05472874	670369	1.16E-11	16.0823046	7.37627164	23.1273504	0.32363741
cg23037403	670369	3.42E-13	16.1009327	8.01040887	26.6239811	0.35518857
cg22681784	3170128	1.27E-11	16.1075426	7.3587505	21.22645	0.30515406
cg27118825	670369	2.64E-11	16.128991	7.2240518	22.3309063	0.31601425
cg11965370	3170128	9.69E-12	16.167688	7.40841486	21.4826135	0.30770353
cg22030890	3170128	4.00E-15	16.1780303	8.78653689	29.2780889	0.37723943
cg06836736	670369	5.94E-12	16.1933478	7.49772131	23.7747099	0.32970954
cg12163490	670369	4.35E-14	16.2108206	8.37344371	28.7554839	0.37301758
cg00468146	3170128	9.26E-13	16.2178033	7.83305262	23.7431976	0.32941649
cg11934695	670369	8.61E-11	16.2488125	7.00401934	21.2090058	0.30497976
cg19616230	3170128	2.95E-11	16.2577527	7.20381038	20.438358	0.29719144
cg26985289	3170128	2.78E-11	16.2653432	7.21460328	20.4927119	0.29774647
cg25806808	670369	4.66E-14	16.267152	8.36186076	28.6860177	0.37245208
cg21494776	3170128	5.11E-15	16.2828811	8.74093056	28.9988862	0.374991
cg09500672	670369	3.61E-11	16.2895475	7.1662443	22.032797	0.31311651
cg20723355	670369	2.42E-14	16.2902433	8.47602547	29.3748936	0.37801523
cg04005707	3170128	2.85E-11	16.2990684	7.21030863	20.4710739	0.29752562
cg01615704	670369	3.82E-12	16.3066878	7.5780861	24.2088881	0.33372135
cg08107272	3170128	2.60E-13	16.3487308	8.05928062	24.9989652	0.34089979
cg12262564	670369	6.66E-16	16.3638937	9.11572099	33.4076672	0.40870147
cg09300114	2680189	5.51E-11	16.3727504	7.0875109	21.9345105	0.31215574

cg08390209	670369	9.88E-12	16.3748393	7.40484733	23.2787155	0.32506702
cg17733100	670369	2.98E-14	16.3777309	8.44045506	29.159258	0.37628446
cg17386185	5390246	3.45E-11	16.3812526	7.17452579	30.2179556	0.38469077
cg14603345	3170128	1.00E-17	16.3848703	10.0081627	37.2976176	0.43556234
cg26896762	2970408	1.10E-10	16.3999021	6.95770117	19.8289497	0.29090795
cg15050111	670369	2.58E-12	16.4026782	7.64889099	24.5952537	0.3372512
cg16254309	3170128	1.65E-11	16.4119716	7.31084039	20.9809675	0.3026932
cg15433631	670369	2.22E-16	16.4196127	9.20477882	33.9923644	0.41290102
cg21870884	670369	5.89E-13	16.421859	7.91381437	26.0727519	0.35041155
cg10549973	3170128	2.44E-15	16.4261685	8.87413379	29.818434	0.38154523
cg15050111	3170128	1.90E-13	16.4282963	8.11454322	25.3111554	0.34369382
cg21621248	3170128	8.77E-12	16.4285066	7.42658649	21.5767714	0.30863595
cg02613386	670369	3.15E-14	16.4329045	8.42950494	29.0930585	0.37575119
cg19005210	3170128	1.43E-11	16.4500642	7.33737979	21.1167524	0.30405653
cg22030890	670369	2.07E-14	16.4749016	8.50314564	29.5399113	0.37933325
cg15439862	3170128	1.00E-17	16.4757402	9.63226088	34.7189195	0.41803706
cg10644361	670369	1.75E-12	16.4839977	7.71909724	24.9819005	0.34074638
cg09083627	670369	4.13E-11	16.4935581	7.14105267	21.9036348	0.31185336
cg20008332	3170128	2.24E-12	16.4964313	7.6743152	22.883407	0.32132062
cg16254309	670369	9.75E-11	16.5048743	6.98072516	21.0922648	0.30381106
cg19461621	3170128	1.85E-11	16.5068433	7.29009436	20.8751663	0.3016272
cg01830294	3170128	2.50E-12	16.5152376	7.65480997	22.7789726	0.32032392
cg01366419	670369	9.19E-14	16.5735922	8.24235369	27.974913	0.36660406
cg09350141	670369	1.00E-17	16.5949017	9.37154134	35.1025094	0.42071259
cg24921089	670369	8.44E-13	16.6073916	7.84954521	25.7096964	0.34722642
cg06744574	3170128	6.14E-13	16.6164593	7.90632169	24.145992	0.33314317
cg24924779	670369	1.01E-10	16.6198711	6.97413484	21.0593073	0.30348041
cg27403635	3170128	4.77E-12	16.6288287	7.53766038	22.1573247	0.31432995
cg23850212	670369	3.83E-11	16.6320255	7.15526058	21.9764255	0.31256579
cg10362591	670369	1.08E-11	16.6609025	7.38865351	23.1928652	0.32425693
cg06391468	670369	2.48E-12	16.6727987	7.65586612	24.6335098	0.33759868
cg24693053	670369	2.08E-13	16.703351	8.09851128	27.1325805	0.35953425
cg10189695	670369	6.57E-12	16.7166418	7.47947677	23.6767868	0.32879805
cg14717170	3170128	6.45E-13	16.7314688	7.89765912	24.0981743	0.33270292
cg24921089	3170128	3.09E-14	16.764532	8.43321745	27.1530359	0.3597078
cg19063972	3170128	1.44E-11	16.7836839	7.33563344	21.1078023	0.30396684
cg20182358	3170128	5.11E-13	16.7948349	7.93891665	24.326387	0.3347988
cg19005210	670369	3.93E-11	16.7967114	7.15029196	21.9509536	0.31231666
cg08575537	3170128	2.54E-11	16.8051001	7.23097658	20.5753245	0.29858838
cg11732619	3170128	4.65E-13	16.8341226	7.95587789	24.4205516	0.33565976
cg08965235	3170128	1.00E-17	16.8353434	10.2456275	38.9775076	0.44642231
cg27634151	3170128	1.55E-12	16.8429362	7.74069813	23.2408243	0.32470972
cg14717170	670369	4.57E-12	16.8571133	7.54538496	24.0316586	0.33208956
cg27268486	3170128	1.72E-11	16.8575678	7.30288663	20.9403691	0.30228454

cg17977409	3170128	6.00E-15	16.8579866	8.71581327	28.8457383	0.37375078
cg14603345	670369	1.00E-17	16.8714217	9.83236687	38.2738854	0.44192489
cg14155416	670369	1.31E-14	16.9112877	8.5806676	30.014515	0.38309304
cg25725843	3170128	3.34E-12	16.9159909	7.60240896	22.4997244	0.3176444
cg22681784	670369	7.31E-12	16.9285228	7.45979553	23.5714201	0.32781449
cg02154186	2970408	1.75E-12	16.9343614	7.71891037	23.7662263	0.32963067
cg11965370	670369	6.58E-12	16.9404467	7.47909127	23.6747203	0.32877878
cg23002761	3170128	1.13E-10	16.9495385	6.95332193	19.1997375	0.28430127
cg08107272	670369	3.31E-13	16.9497065	8.01606538	26.656468	0.35546791
cg21504918	3170128	4.64E-11	17.0177237	7.1194352	20.0162423	0.29285101
cg17457560	3170128	4.18E-13	17.0209575	7.97499353	24.5269178	0.33662961
cg04005707	670369	2.34E-11	17.024	7.24665708	22.4481316	0.31714703
cg26985289	670369	1.87E-11	17.0520014	7.28783035	22.6625868	0.31920971
cg05839235	3170128	7.35E-14	17.0691187	8.28180932	26.2690781	0.35212103
cg20182358	670369	2.00E-12	17.097485	7.6949536	24.8485357	0.33954497
cg00565688	3170128	2.22E-15	17.1085713	8.89811453	29.9672947	0.382721
cg26186727	3170128	5.09E-12	17.1213532	7.52591138	22.0955082	0.31372812
cg10549973	670369	2.00E-15	17.1255082	8.89985478	32.0140151	0.3984452
cg00386408	3170128	4.44E-12	17.1463245	7.55041334	22.2245326	0.31498306
cg18438777	3170128	1.35E-11	17.1654465	7.34800296	21.1712421	0.30460214
cg27223047	3170128	3.03E-12	17.1811645	7.61972808	22.5918068	0.31853031
cg01830294	670369	2.44E-12	17.1923673	7.65870674	24.6490996	0.33774017
cg10303487	3170128	1.91E-14	17.2222097	8.51818471	27.6561107	0.36394674
cg08575537	670369	5.38E-11	17.2387312	7.09193152	21.6530897	0.30938986
cg27268486	670369	4.12E-11	17.2567093	7.14166285	21.906758	0.31188396
cg12331389	3170128	2.91E-14	17.2649413	8.44298335	27.2106014	0.36019571
cg08422599	3170128	1.11E-10	17.2746166	6.95593433	19.2124291	0.28443574
cg02919422	3170128	3.88E-11	17.2847009	7.15280856	20.1826095	0.29456808
cg15439862	670369	1.00E-17	17.2855405	9.7627368	37.7849337	0.43875632
cg06187947	670369	1.55E-15	17.2895118	8.94446061	32.2992568	0.40057323
cg13853198	3170128	3.51E-11	17.298652	7.17136631	20.2754569	0.29552273
cg25725843	670369	9.02E-12	17.3006188	7.42150693	23.3672313	0.32590024
cg17619823	3170128	1.69E-14	17.3380847	8.53731733	27.7700869	0.36489933
cg18438777	670369	6.00E-11	17.3656369	7.07160889	21.5499387	0.30837049
cg05670348	3170128	2.58E-14	17.3686224	8.46462769	27.3384223	0.36127644
cg24881834	3170128	4.48E-12	17.3687394	7.54887918	22.2164415	0.3149045
cg15057581	670369	2.47E-13	17.3833982	8.06810823	26.9564405	0.35803588
cg10210238	3170128	1.58E-11	17.3866174	7.3185625	21.0204258	0.30308993
cg19461621	670369	8.98E-12	17.4036241	7.42225377	23.371204	0.32593759
cg02204046	3170128	1.64E-11	17.4103231	7.31184752	20.9861114	0.30274494
cg15013019	670369	7.46E-14	17.410359	8.27873957	28.1903366	0.36838715
cg15426734	3170128	1.80E-13	17.4161181	8.1241018	25.3653703	0.34417661
cg26320696	670369	2.86E-13	17.4222816	8.04209927	26.8062835	0.356753
cg13449778	3170128	1.77E-11	17.4339989	7.29832867	20.9171238	0.30205034

cg21494776	670369	4.44E-16	17.4343403	9.13603443	33.5405319	0.40966103
cg11323198	3170128	2.53E-12	17.451794	7.65268223	22.7675963	0.32021517
cg08315770	3170128	1.37E-11	17.4606991	7.34501108	21.1558878	0.30444848
cg10694152	3170128	8.61E-11	17.4620921	7.00401673	19.4468765	0.28691083
cg00468146	670369	9.64E-14	17.4701007	8.23414852	27.9264652	0.36620167
cg03289872	3170128	1.02E-11	17.4733228	7.39839	21.4307675	0.30718905
cg26619317	670369	3.29E-11	17.4854393	7.18321269	22.1200535	0.31396721
cg27634151	670369	1.61E-12	17.5168714	7.73428606	25.0660147	0.34150187
cg05839235	670369	1.81E-13	17.5291855	8.12302765	27.2750985	0.36074149
cg14407437	3170128	2.18E-11	17.5316719	7.25947114	20.7195424	0.30005329
cg12388309	3170128	3.71E-14	17.5352398	8.40134964	26.9656532	0.35811442
cg06744574	670369	2.27E-13	17.5587502	8.08299385	27.0425975	0.35876967
cg12506971	3170128	2.84E-14	17.562263	8.44775338	27.2387428	0.36043396
cg16898420	3170128	1.36E-11	17.5632817	7.34592613	21.1605832	0.30449548
cg16483916	3170128	3.59E-13	17.5874281	8.0018355	24.6767069	0.33799059
cg08319991	3170128	2.55E-11	17.6050347	7.23085724	20.5747217	0.29858224
cg26200580	3170128	3.55E-12	17.6154373	7.59139701	22.441285	0.31708098
cg20322876	3170128	2.28E-11	17.6187665	7.25118473	20.6775444	0.29962732
cg10453365	670369	2.33E-13	17.6194771	8.07806377	27.0140449	0.35852667
cg08965235	670369	1.00E-17	17.6233411	10.3512585	42.027048	0.46510481
cg26059153	3170128	7.41E-12	17.6271861	7.45735287	21.736716	0.31021408
cg00513220	3170128	1.52E-13	17.6369411	8.15362964	25.5332511	0.34566714
cg03289872	670369	5.08E-11	17.6471002	7.10282726	21.7085147	0.30993635
cg26186727	670369	8.39E-12	17.6610457	7.43484167	23.4382247	0.32656703
cg26335299	3170128	4.40E-11	17.6634917	7.12937472	20.0657098	0.29336243
cg17977409	670369	3.55E-15	17.6710138	8.81344105	31.4654809	0.39431013
cg24881834	670369	1.50E-11	17.6908996	7.3285426	22.8758355	0.32124846
cg25044651	3170128	1.02E-13	17.6992092	8.22370092	25.9340794	0.34919864
cg02525756	670369	7.50E-11	17.7101837	7.02986802	21.3390041	0.30627656
cg12388309	670369	2.82E-13	17.7155376	8.04465874	26.8210385	0.35687929
cg21238818	3170128	1.10E-11	17.725927	7.38454076	21.3592584	0.30647818
cg21504918	670369	4.21E-11	17.7427345	7.13773643	21.8866657	0.31168707
cg20312228	3170128	4.10E-11	17.7433076	7.1426382	20.1318275	0.29404484
cg21637033	3170128	2.83E-11	17.7510613	7.2111099	20.4751101	0.29756683
cg17685628	670369	1.11E-11	17.7543534	7.38388658	23.1676295	0.32401843
cg22821324	3170128	4.61E-11	17.8010627	7.12084931	20.023276	0.29292377
cg15013019	3170128	6.66E-16	17.8049255	9.09993467	31.236013	0.3925634
cg01410472	3170128	1.33E-15	17.8102359	8.98016092	30.4796372	0.38673377
cg08319991	670369	1.07E-10	17.8161517	6.96409985	21.0091832	0.30297694
cg00386408	670369	4.64E-12	17.8296387	7.54262145	24.0167164	0.33195162
cg13853198	670369	5.53E-11	17.8443617	7.08701909	21.6281286	0.30914346
cg19063972	670369	4.25E-12	17.8477548	7.55851082	24.1027044	0.33274466
cg08572611	3170128	5.61E-12	17.8977986	7.50825674	22.002801	0.31282358
cg10303487	670369	1.84E-14	17.929863	8.52391578	29.6666475	0.38034173

cg27444994	3170128	1.32E-11	17.9433481	7.35226648	21.1931332	0.30482109
cg21172540	3170128	2.73E-14	17.946264	8.45434441	27.2776535	0.36076309
cg21621248	670369	4.67E-13	17.9516544	7.95514586	26.307797	0.35245711
cg16898420	670369	3.45E-11	17.9627488	7.17438272	22.0746215	0.31352454
cg11011938	3170128	6.31E-14	17.9724074	8.30839074	26.4231081	0.35345594
cg20008332	670369	1.18E-13	17.9753221	8.19804377	27.7138563	0.36442972
cg13765303	3170128	5.57E-12	18.0075041	7.50931972	22.0083767	0.31287805
cg00565688	670369	8.88E-16	18.0116946	9.060492	33.0479244	0.40608766
cg06379754	3170128	3.35E-12	18.011798	7.60154712	22.4951477	0.31760031
cg05670348	670369	3.31E-14	18.0130321	8.42193982	29.0473734	0.37538263
cg18239753	3170128	2.00E-15	18.0168693	8.89742687	29.9630205	0.3826873
cg01939681	670369	7.21E-11	18.0268432	7.03732698	21.3766059	0.30665076
cg17457560	670369	1.29E-13	18.0295613	8.18289753	27.6249433	0.36368576
cg21096399	3170128	1.79E-11	18.0320341	7.29588077	20.9046457	0.30192455
cg04008901	3170128	1.01E-12	18.0763419	7.81814465	23.6617006	0.3286574
cg08315770	670369	1.79E-11	18.0806585	7.29559018	22.7031408	0.31959836
cg21034676	3170128	1.32E-13	18.0906803	8.17831812	25.6740851	0.34691232
cg13686115	3170128	7.07E-11	18.0967002	7.04102603	19.6284319	0.2888158
cg21513385	3170128	4.52E-12	18.1220557	7.54733043	22.2082753	0.31482519
cg26200580	670369	6.45E-12	18.142204	7.48278309	23.694515	0.32896325
cg23002761	670369	2.48E-11	18.1666999	7.23548067	22.3901278	0.31658699
cg12331389	670369	1.18E-14	18.1909832	8.60195334	30.1455839	0.38412334
cg20483374	3170128	2.57E-11	18.1948403	7.22920587	20.5663813	0.29849734
cg15426734	670369	1.29E-13	18.2140755	8.18331137	27.6273705	0.36370609
cg25084878	3170128	2.92E-11	18.2175978	7.2051997	20.4453502	0.29726289
cg05380982	3170128	8.28E-12	18.2302112	7.43727541	21.6322647	0.3091843
cg11323198	670369	2.01E-12	18.2304681	7.69402813	24.8434319	0.33949891
cg12506971	670369	3.38E-14	18.2322189	8.41781754	29.0224966	0.37518176
cg07684796	3170128	1.55E-15	18.2698774	8.95551917	30.3252666	0.38553021
cg13929328	3170128	1.00E-17	18.2764127	9.45929553	33.5655307	0.40984123
cg27403635	670369	1.51E-13	18.2874775	8.15479331	27.4603986	0.36230435
cg26059153	670369	8.35E-12	18.304573	7.43562062	23.4423757	0.32660598
cg21637033	670369	4.17E-11	18.3366135	7.13949892	21.8956834	0.31177545
cg04809787	670369	9.34E-11	18.3400174	6.98883797	21.1328787	0.3042181
cg27603796	3170128	3.41E-12	18.3677031	7.59818166	22.4772805	0.31742813
cg08422599	670369	3.58E-11	18.3816937	7.16752114	22.0393558	0.31318053
cg02265318	3170128	2.22E-16	18.3887698	9.24373578	32.1573612	0.39951651
cg07684796	670369	1.98E-14	18.3890414	8.51163313	29.5916633	0.37974546
cg00044245	670369	5.55E-11	18.3943225	7.08627523	21.6243504	0.30910615
cg27444994	670369	2.95E-11	18.3946637	7.203442	22.2243483	0.31498127
cg14889768	670369	7.06E-11	18.3978762	7.04138456	21.3970774	0.30685432
cg22794078	3170128	1.98E-13	18.3994826	8.10738624	25.2706039	0.34333223
cg23984434	3170128	3.06E-11	18.4234716	7.19671906	20.4026898	0.29682674
cg07028533	3170128	2.66E-15	18.4562396	8.85254851	29.6847866	0.3804858

cg17619823	670369	2.89E-15	18.4649328	8.84319668	31.6537585	0.39573583
cg27654142	3170128	6.66E-16	18.5084916	9.0849095	31.1405788	0.39183397
cg01939681	3170128	1.80E-12	18.5186367	7.7134273	23.0936208	0.32331801
cg06722216	3170128	6.90E-11	18.5221319	7.04560102	19.6509418	0.28905128
cg27223047	670369	3.21E-13	18.5372343	8.02152768	26.6878613	0.35573762
cg26335299	670369	2.72E-11	18.5505639	7.21861156	22.3027493	0.31574159
cg25432696	3170128	4.43E-13	18.5600368	7.96434585	24.4676389	0.33608945
cg21034676	670369	3.30E-13	18.5690861	8.01700409	26.6618615	0.35551426
cg23001650	5390246	2.42E-11	18.5924737	7.23993509	30.5949079	0.38762941
cg20387341	3170128	8.40E-11	18.6042808	7.00880332	19.4703041	0.28715722
cg00513220	670369	5.93E-14	18.6068959	8.31946	28.4325482	0.37038001
cg08441170	670369	1.38E-11	18.6199263	7.34385316	22.9563388	0.32201493
cg26128092	3170128	2.19E-13	18.6234166	8.08936337	25.1686445	0.34242132
cg24467291	3170128	1.82E-12	18.6256546	7.71205446	23.0862242	0.32324793
cg09313439	3170128	4.42E-13	18.6256978	7.96485558	24.4704749	0.33611531
cg21513385	670369	9.11E-12	18.6275282	7.41964511	23.3573291	0.32580713
cg24659201	3170128	1.71E-12	18.6325509	7.72320246	23.1463258	0.32381696
cg20312228	670369	2.47E-11	18.643117	7.23673995	22.3966588	0.3166501
cg16483916	670369	1.02E-13	18.6499047	8.22400585	27.866644	0.3657041
cg01410472	670369	8.88E-16	18.6518359	9.06968095	33.1076265	0.40652304
cg26220350	3170128	6.57E-11	18.6523584	7.0545976	19.6952493	0.28951433
cg23130254	3170128	2.05E-11	18.673124	7.27078526	20.7769632	0.30063484
cg25044651	670369	3.91E-14	18.6732632	8.39234259	28.8690325	0.37393974
cg26524899	3170128	4.98E-12	18.6831527	7.52964878	22.1151618	0.31391958
cg10210238	670369	2.50E-12	18.688088	7.65444016	24.6256861	0.33752765
cg21238818	670369	5.17E-12	18.6951336	7.52284001	23.9099187	0.33096404
cg08432727	3170128	6.77E-14	18.7050849	8.29597074	26.3510771	0.35283236
cg21096399	670369	2.11E-11	18.7067569	7.26521232	22.5446281	0.3180767
cg24713204	2370010	9.06E-11	18.7185116	6.99464878	18.7548387	0.27955507
cg02204046	670369	2.52E-12	18.7234741	7.65292183	24.6173572	0.33745201
cg09300114	2970408	3.46E-13	18.7574882	8.00861002	25.3719629	0.34423528
cg13297960	3170128	2.44E-14	18.7578567	8.47413198	27.3946534	0.36175072
cg14603345	2370010	9.63E-11	18.7695668	6.98312658	18.699122	0.27895625
cg03014628	3170128	1.62E-14	18.7700473	8.54494937	27.8156236	0.36527912
cg11260422	3170128	1.21E-11	18.7703688	7.36726589	21.270249	0.3055913
cg03963198	670369	1.08E-10	18.7780917	6.96073691	20.9924017	0.30280821
cg10694152	670369	1.43E-11	18.8046409	7.33660185	22.9181903	0.32165193
cg13449778	670369	1.97E-12	18.8523248	7.69767731	24.86356	0.33968054
cg08572611	670369	2.41E-12	18.8953978	7.66102067	24.661803	0.33785543
cg27603796	670369	6.61E-12	18.8967651	7.47833223	23.6706518	0.32874086
cg21172540	670369	1.13E-14	18.8996448	8.60733694	30.1787854	0.38438379
cg20322876	670369	4.15E-12	18.9030036	7.56287975	24.1263793	0.33296267
cg02332525	3170128	1.00E-17	18.9108691	10.6335511	41.8065203	0.46379619
cg03469054	3170128	3.52E-13	18.9353099	8.00548401	24.6971059	0.33817551

cg13929328	670369	1.00E-17	18.94003	9.39613783	35.267936	0.42185886
cg11732619	670369	1.55E-15	18.9458626	8.94185477	32.282554	0.40044903
cg18239753	670369	8.88E-16	18.9563395	9.05111012	32.9870313	0.40564293
cg18765542	3170128	8.92E-13	18.987848	7.8397621	23.7799268	0.32975803
cg07028533	670369	6.44E-15	18.9916337	8.70170546	30.7641436	0.38893963
cg04008901	670369	5.42E-13	18.9961895	7.92845136	26.1558499	0.35113622
cg22821324	670369	1.13E-11	19.0160392	7.37967692	23.1453575	0.3238078
cg24467291	670369	5.58E-12	19.0164614	7.50912932	23.8360608	0.33027935
cg19547629	670369	4.61E-11	19.0176423	7.12064987	21.7993593	0.31083021
cg14407437	670369	1.92E-12	19.0394818	7.70182107	24.8864278	0.33988677
cg22794078	670369	2.81E-13	19.0458486	8.04540727	26.8253545	0.35691622
cg13686115	670369	3.95E-11	19.0470174	7.14962259	21.9475234	0.31228309
cg02265318	670369	4.44E-16	19.0678564	9.19175314	33.9064912	0.41228798
cg19547629	3170128	4.44E-12	19.0736534	7.55053921	22.2251965	0.31498951
cg21816539	3170128	3.10E-13	19.1075495	8.02760875	24.8210058	0.33929643
cg02332525	670369	1.00E-17	19.1125694	10.1062453	40.2308108	0.45425619
cg10059959	3170128	1.00E-17	19.1599657	9.37922482	33.0386701	0.40602011
cg08918749	3170128	4.59E-12	19.1666855	7.54475881	22.1947193	0.31469349
cg11011938	670369	1.02E-14	19.1766655	8.62510643	30.2885199	0.38524302
cg26524899	670369	1.07E-11	19.180184	7.38963993	23.1980893	0.32430628
cg13297960	670369	7.57E-14	19.211626	8.2763535	28.1761807	0.36827029
cg08817120	3170128	9.38E-13	19.2264184	7.83070924	23.7303768	0.32929718
cg15457899	3170128	1.95E-13	19.276708	8.10996984	25.2852385	0.34346277
cg13488201	3170128	1.24E-14	19.2801827	8.59323023	28.104635	0.36767899
cg02590345	3170128	1.20E-13	19.2838921	8.19485136	25.768636	0.34774563
cg26220350	670369	7.41E-11	19.3639394	7.03210188	21.3502612	0.30638863
cg21184174	3170128	1.60E-11	19.4022733	7.31647407	21.0097503	0.30298264
cg25432696	670369	3.20E-13	19.411089	8.02208436	26.6910619	0.35576511
cg05764376	3170128	1.24E-11	19.435204	7.3627252	21.2468876	0.30535815
cg06379754	670369	3.53E-13	19.4387054	8.004931	26.5925421	0.354918
cg08186362	3170128	1.00E-17	19.4530751	9.80753937	35.909053	0.42625874
cg13765303	670369	5.72E-13	19.4582018	7.91890678	26.1016454	0.35066371
cg08965235	2370010	3.00E-11	19.4598194	7.20019298	19.7642024	0.29023374
cg20483374	670369	5.41E-12	19.4749389	7.51465734	23.8658234	0.33055543
cg10539507	3170128	3.03E-13	19.4867569	8.03220486	24.8467871	0.33952919
cg09313439	670369	3.05E-13	19.4935437	8.03079989	26.7412001	0.35619536
cg14764661	3170128	4.96E-13	19.4980945	7.9445128	24.3574332	0.33508291
cg03506489	3170128	3.67E-11	19.4993335	7.16291888	20.2331633	0.29508819
cg08186362	670369	2.22E-16	19.5395156	9.25905133	34.3514709	0.41545083
cg09619786	3170128	1.28E-12	19.5758091	7.77484733	23.4258887	0.32645126
cg26128092	670369	1.06E-13	19.5884065	8.21656844	27.8228252	0.36533914
cg03014628	670369	1.27E-14	19.5960316	8.58681164	30.0523142	0.38339052
cg24659201	670369	8.32E-13	19.6172626	7.85220511	25.7246634	0.34735835
cg08432727	670369	3.75E-14	19.6280099	8.39907391	28.9095376	0.37426804

cg19428336	3170128	3.53E-11	19.6556357	7.17028864	20.2700586	0.2954673
cg27654142	670369	1.00E-17	19.6733532	9.39635054	35.2693685	0.42186876
cg21816539	670369	5.96E-13	19.6836904	7.91171267	26.0608326	0.35030748
cg04418492	3170128	7.64E-13	19.6912612	7.8673449	23.9312514	0.33116154
cg24975222	3170128	2.22E-16	19.7137236	9.41300521	33.2603969	0.40763422
cg25084878	670369	2.84E-12	19.7646414	7.63163683	24.5007707	0.33639146
cg09619786	670369	7.14E-12	19.7862571	7.46412805	23.5945911	0.32803103
cg10059959	670369	2.22E-16	19.8090298	9.28209081	34.5045552	0.41653108
cg26896762	3170128	1.69E-11	19.8163118	7.30592806	20.9558883	0.30244081
cg18059088	3170128	5.92E-11	19.8206368	7.07412652	19.7916226	0.29051942
cg23130254	670369	5.69E-12	19.8854991	7.50552333	23.8166581	0.33009925
cg08817120	670369	1.29E-12	19.9040689	7.77332035	25.2829412	0.34344228
cg04418492	670369	4.27E-12	19.9163031	7.55790713	24.0994341	0.33271453
cg12300353	3170128	1.03E-11	19.9167582	7.39640714	21.420521	0.30708727
cg23984434	670369	2.99E-12	19.9871853	7.62193207	24.4477216	0.33590777
cg05380982	670369	3.48E-13	19.9903701	8.00738806	26.6066412	0.35503937
cg19211800	670369	5.23E-12	19.9971085	7.52089923	23.8994557	0.33086713
cg24975222	670369	8.88E-16	19.9982668	9.03986292	32.9141144	0.40510952
cg13488201	670369	1.38E-14	20.0358426	8.5754328	29.982331	0.38283952
cg06498267	3170128	4.88E-14	20.067484	8.35302964	26.6828851	0.35569488
cg14764661	670369	9.43E-13	20.0847759	7.82984235	25.5989882	0.34624894
cg27501458	3170128	6.13E-11	20.1269853	7.0675616	19.7591956	0.29018155
cg03506489	670369	5.60E-11	20.127	7.08465888	21.6161421	0.30902508
cg23748737	3170128	1.51E-12	20.1329494	7.7451173	23.2647272	0.32493516
cg02590345	670369	8.50E-14	20.1692124	8.25598208	28.0554891	0.36727218
cg02154186	3170128	3.04E-13	20.2915965	8.03130006	24.8417106	0.33948337
cg12300353	670369	3.07E-11	20.3144028	7.19641094	22.1880655	0.31462883
cg08965235	7650451	7.26E-11	20.3775817	7.03614086	18.1018714	0.27247408
cg00333226	3170128	1.13E-14	20.4323856	8.60883628	28.1984017	0.36845371
cg18059088	670369	9.51E-11	20.4327095	6.98546771	21.116001	0.30404901
cg22380033	2370010	1.07E-10	20.4468428	6.96419664	18.6077839	0.27797241
cg18765542	670369	8.46E-14	20.4805564	8.25712443	28.0622492	0.36732817
cg16270890	3170128	2.14E-13	20.4862112	8.09337677	25.1913296	0.34262421
cg20496643	670369	2.00E-11	20.4935897	7.27578637	22.5997288	0.31860642
cg03469054	670369	2.31E-14	20.4984985	8.48361673	29.4210309	0.3783843
cg19731268	3170128	6.23E-13	20.5208346	7.90384261	24.132302	0.33301719
cg08918749	670369	7.44E-13	20.5553648	7.8720951	25.8367433	0.34834457
cg15731815	3170128	4.44E-16	20.6383122	9.12415741	31.3901989	0.39373819
cg15457899	670369	1.91E-14	20.7344182	8.51584991	29.6173941	0.3799502
cg03379131	3170128	2.68E-13	20.828533	8.05352859	24.9665934	0.34060871
cg19428336	670369	1.28E-11	20.8451132	7.35787958	23.0302375	0.322717
cg06498267	670369	4.02E-14	20.9395348	8.38677651	28.8355637	0.37366821
cg03379131	670369	2.02E-12	20.9896149	7.69269202	24.8360646	0.3394324
cg11051843	2370010	3.77E-11	21.0819376	7.15792141	19.5542322	0.2880385

cg10300684	3170128	1.78E-12	21.1065409	7.71553268	23.1049668	0.32342548
cg23748737	670369	8.55E-13	21.1451971	7.84729694	25.6970496	0.34711491
cg18972811	3170128	2.89E-15	21.1781055	8.85100797	29.6752607	0.38041015
cg20052718	3170128	1.03E-10	21.2541716	6.96991506	19.2804319	0.28515543
cg15731815	670369	1.11E-15	21.2719641	8.9883462	32.5812845	0.40266253
cg16270890	670369	2.29E-13	21.296394	8.08124313	27.0324562	0.35868338
cg21184174	670369	6.81E-13	21.3059807	7.88781435	25.9255216	0.34912364
cg02154186	670369	1.60E-13	21.3219439	8.1452712	27.404777	0.36183603
cg16065186	3170128	6.07E-12	21.3942477	7.4938428	21.9272727	0.31208488
cg26111030	3170128	1.13E-12	21.4750086	7.79733299	23.5481898	0.32759726
cg27501458	670369	1.61E-11	21.4840973	7.31565582	22.8082067	0.32060322
cg18972811	670369	1.29E-14	21.6018044	8.5851461	30.0420648	0.38330988
cg08145177	3170128	1.00E-17	21.6136464	10.1085812	38.0031987	0.44017518
cg19286604	3170128	1.67E-14	21.6840777	8.5420032	27.7980404	0.36513252
cg10300684	670369	2.95E-12	21.7799005	7.6248008	24.4633959	0.33605076
cg18952560	3170128	1.04E-12	21.799893	7.81268963	23.6319186	0.32837957
cg08731300	3170128	1.96E-11	21.8006957	7.27949873	20.8212463	0.30108268
cg15647515	670369	1.53E-11	21.8123468	7.32467591	22.8555309	0.32105486
cg22380033	7650451	8.47E-11	21.8803208	7.00710283	17.9625888	0.27094561
cg00333226	670369	1.11E-15	21.9157204	9.01832086	32.774708	0.40408704
cg01942127	670369	8.55E-11	21.9594439	7.00541798	21.2160276	0.30504993
cg12832649	670369	5.62E-11	22.00043	7.08387534	21.6121637	0.30898578
cg17733331	3170128	3.08E-12	22.0084867	7.61674022	22.575906	0.3183775
cg19731268	670369	7.73E-14	22.0370618	8.27307088	28.1567126	0.3681095
cg15050111	2370010	2.61E-11	22.078037	7.22593375	19.8926669	0.29157018
cg14667273	3170128	7.88E-13	22.177628	7.86178422	23.9007017	0.33087867
cg26111030	670369	1.40E-12	22.2699595	7.75907147	25.2036288	0.34273416
cg08145177	670369	1.00E-17	22.2927077	9.95755836	39.1617373	0.44758793
cg08731300	670369	4.29E-11	22.3518252	7.13411595	21.8681489	0.31150552
cg17733331	670369	1.01E-11	22.4350759	7.40168594	23.2619409	0.32490889
cg04391111	670369	4.04E-11	22.514342	7.14521661	21.9249528	0.31206216
cg18952560	670369	1.48E-12	22.5537352	7.74845709	25.1446415	0.34220651
cg16065186	670369	2.41E-12	22.6184346	7.66121566	24.6628737	0.33786514
cg20052718	670369	2.43E-11	22.7493025	7.23959978	22.4114949	0.3167934
cg10549973	2370010	1.05E-13	22.7837265	8.21960274	25.2022038	0.34272142
cg05670348	2370010	2.06E-11	22.784541	7.27035997	20.1154634	0.29387607
cg14667273	670369	9.65E-13	23.0039331	7.82560448	25.5752125	0.34603864
cg19286604	670369	2.00E-15	23.1924012	8.90679545	32.058305	0.39877661
cg19764436	3170128	2.69E-14	23.3280767	8.45767508	27.2973279	0.36092938
cg10362591	2370010	1.16E-11	23.3371024	7.37502783	20.6457689	0.29930469
cg19764436	670369	1.21E-13	23.7612759	8.19411146	27.6907566	0.36423661
cg15050111	7650451	8.64E-12	23.9988831	7.42934927	20.0446279	0.29314457
cg13344740	3170128	5.11E-11	24.1468627	7.10164419	19.9278716	0.29193554
cg20322876	2370010	5.82E-11	25.2586211	7.07719678	19.1566939	0.28384481

cg13344740	670369	1.82E-11	25.6240996	7.29268566	22.6879563	0.31945289
cg09300114	3170128	1.00E-17	26.0559369	10.5809441	41.4167114	0.46146731
cg26619317	7650451	3.27E-11	26.1282016	7.18478852	18.8238902	0.28029584
cg21034676	7650451	8.76E-12	26.2795624	7.42692071	20.0323048	0.29301716
cg04008901	7650451	3.88E-11	26.3574115	7.1529797	18.6681174	0.27862259
cg09300114	670369	1.00E-17	26.6221646	10.2474909	41.2610565	0.4605317
cg15731815	7650451	2.07E-11	27.4585855	7.26935466	19.2413852	0.28474237
cg24120841	7650451	1.92E-13	27.8961405	8.11242605	23.6706677	0.32874101
cg19731268	2370010	3.58E-11	27.9191121	7.16783876	19.6033824	0.28855358
cg24496666	7650451	8.54E-11	29.7774085	7.00574314	17.956081	0.27087403
cg27501458	7650451	4.73E-11	31.4505694	7.11580494	18.4869427	0.27666666

R_squared_a	MethChr	MethLoc	MethGeneID	MethSymbol	XpChr	XpLoc
0.31643359	1	148019991	2209	FCGR1A	14	24145611-24
0.30917492	3	39297107	1524	CX3CR1	14	24145611-24
0.28927491	7	30758396	11185	INMT	2	86865508-86
0.32029224	8	143830944	137797	LYPD2	2	86865508-86
0.28233522	7	30758396	11185	INMT	2	86865488-86
0.35837678	19	56582398	3982	LIM2	14	24145611-24
0.31632641	8	143830964	137797	LYPD2	2	86865508-86
0.31440937	2	241775353	50636	TMEM16G	2	86865488-86
0.29651308	8	143830944	137797	LYPD2	2	86865488-86
0.30112988	8	143830964	137797	LYPD2	2	86865488-86
0.29194396	2	241775353	50636	TMEM16G	2	86865508-86
0.30210538	7	150319489	4846	NOS3	2	86865508-86
0.36003141	1	151851544	140576	S100A16	2	86865508-86
0.3306099	11	66979224	57010	CABP4	2	86865508-86
0.27943123	3	52803834	3699	ITIH3	2	86865488-86
0.34397903	19	53952022	26291	FGF21	2	86865508-86
0.29535034	15	41300290	2038	EPB42	2	86865508-86
0.32146499	11	66979224	57010	CABP4	2	86865488-86
0.34207957	1	151851544	140576	S100A16	2	86865488-86
0.27830839	7	150319489	4846	NOS3	2	86865488-86
0.27634945	1	110456815	164153	UBL4B	2	86865488-86
0.27052255	19	45588003	147746	HIPK4	2	86865488-86
0.27503639	1	199448506	91156	DKFZp434B12	2	86865488-86
0.34098275	11	384607	11187	PKP3	2	86865508-86
0.27895388	15	41300290	2038	EPB42	2	86865488-86
0.3239582	19	53952022	26291	FGF21	2	86865488-86
0.27266715	1	199448025	91156	DKFZp434B12	2	86865488-86
0.35406537	3	39297107	1524	CX3CR1	4	15573160-15
0.40512174	3	39297107	1524	CX3CR1	16	56256163-56
0.39826422	3	39297107	1524	CX3CR1	16	56256102-56
0.31335253	17	37023863	3868	KRT16	2	86865508-86
0.43357761	19	56582398	3982	LIM2	4	15573160-15
0.31527059	11	384607	11187	PKP3	2	86865488-86
0.30423328	11	116205766	345	APOC3	2	86865508-86
0.30103136	17	37023863	3868	KRT16	2	86865488-86
0.29624621	19	3679751	27134	TJP3	2	86865488-86
0.32069555	16	56563625	1258	CNGB1	2	86865508-86
0.31338242	10	80631388	57178	RAI17	2	86865508-86
0.28876232	11	6823856	144124	OR10A5	2	86865508-86
0.31490448	16	56563625	1258	CNGB1	2	86865488-86
0.2845315	11	116205766	345	APOC3	2	86865488-86
0.33786347	19	56564177	125875	MGC33839	19	10484758-10
0.41619982	3	39297107	1524	CX3CR1	19	10484758-10

0.42249328	19	56582398	3982	LIM2	16	56256163-56
0.28133644	7	142340719	56302	TRPV5	2	86865488-86
0.32474064	14	24173141	3002	GZMB	4	15573160-15
0.28060555	17	36996149	3861	KRT14	2	86865488-86
0.40925049	19	56582398	3982	LIM2	16	56256102-56
0.27752253	10	80631388	57178	RAI17	2	86865488-86
0.29319761	1	154963371	81875	ISG20L2	2	86865508-86
0.32174436	1	25164659	864	RUNX3	2	86865508-86
0.3125936	17	41216118	1394	CRHR1	2	86865508-86
0.34762387	14	24173141	3002	GZMB	16	56256102-56
0.29909361	20	34163743	2036	EPB41L1	2	86865508-86
0.29873665	20	34163743	2036	EPB41L1	2	86865488-86
0.32067332	1	25164659	864	RUNX3	2	86865488-86
0.28098228	1	154963371	81875	ISG20L2	2	86865488-86
0.30780198	17	41216118	1394	CRHR1	2	86865488-86
0.34342763	14	24173141	3002	GZMB	16	56256163-56
0.33366786	7	73584338	80112	WBSCR23	2	86865508-86
0.38321236	3	39297107	1524	CX3CR1	19	56566846-56
0.29773374	19	56582398	3982	LIM2	2	85778301-85
0.45367725	19	56582398	3982	LIM2	19	10484758-10
0.32764905	7	73584338	80112	WBSCR23	2	86865488-86
0.33555417	19	56582398	3982	LIM2	2	86865508-86
0.33250344	19	56582398	3982	LIM2	2	86865488-86
0.29475208	1	86706913	1179	CLCA1	2	86865508-86
0.27633701	6	33348842	6293	VPS52	2	86865488-86
0.28912654	1	86706913	1179	CLCA1	2	86865488-86
0.34273779	14	24173141	3002	GZMB	19	10484758-10
0.45284965	19	56582398	3982	LIM2	10	72027389-72
0.31024044	12	50865769	144501	LOC144501	2	86865508-86
0.27730468	1	44217190	8704	B4GALT2	9	93211451-93
0.31218185	12	50865769	144501	LOC144501	2	86865488-86
0.32423779	19	56582398	3982	LIM2	19	3728979-372
0.24034375	3	39297107	1524	CX3CR1	19	3728979-372
0.38773254	10	85922743	387695	C10orf99	17	35963692-35
0.27133366	12	4425047	2251	FGF6	2	86865488-86
0.30924867	22	49523273	49	ACR	2	86865488-86
0.35587731	3	39297107	1524	CX3CR1	10	72027389-72
0.27772475	2	20714785	64342	HS1BP3	6	41351742-41
0.31042781	14	73885069	55237	C14orf115	2	86865508-86
0.26287151	14	24173141	3002	GZMB	19	3728979-372
0.33902257	14	24173141	3002	GZMB	19	56566846-56
0.43156475	1	32488799	3932	LCK	14	98705794-98
0.29606945	22	49523273	49	ACR	2	86865508-86
0.26310189	1	148019991	2209	FCGR1A	17	31222639-31

0.29779783	14	73885069	55237	C14orf115		2	86865488-86
0.36097331	14	24173141	3002	GZMB		10	72027389-72
0.2737223	3	39297107	1524	CX3CR1		11	65407708-65
0.26125412	3	39297107	1524	CX3CR1		1	91920750-91
0.33063932	19	56582398	3982	LIM2		11	65407708-65
0.27577223	11	66979224	57010	CABP4		5	145949378-1
0.30726385	19	56582398	3982	LIM2		12	10353263-10
0.30766024	9	94937037	4814	NINJ1		1	117113051-1
0.28303333	11	64459991	170589	GPHA2		2	86865488-86
0.25733571	3	133518757	55	ACPP		17	55591584-55
0.30178359	8	95291196	1015	CDH17		19	10484758-10
0.33305254	17	37023863	3868	KRT16		1	117113051-1
0.25012993	6	41129139	10930	APOBEC2		20	3734490-373
0.26245139	3	45041975	7123	CLEC3B		5	145949378-1
0.24064879	3	152529997	53829	P2RY13		16	56277117-56
0.28082569	19	56582398	3982	LIM2		1	91920750-91
0.27918681	15	75073287	9051	PSTPIP1		1	117113051-1
0.24601728	11	8911469	56673	C11orf16		5	145949378-1
0.32919138	19	5237082	5802	PTPRS		1	117113051-1
0.25084286	19	46992652	1084	CEACAM3		2	71763245-71
0.24061784	19	40727715	10430	NIFIE14	X		17075527-17
0.29772917	8	95291196	1015	CDH17		19	56566846-56
0.27597676	19	4254012	126259	MGC23244		11	60544218-60
0.25520825	1	36721568	1441	CSF3R		17	55591584-55
0.25558379	11	384607	11187	PKP3		5	145949378-1
0.34261488	16	56563625	1258	CNGB1		1	117113051-1
0.28517285	12	56135137	83729	INHBE		5	10514353-10
0.38389801	1	153253136	51043	ZBTB7B		17	35963692-35
0.32250779	3	39297107	1524	CX3CR1		5	145949378-1
0.26711342	12	13139815	83445	GSG1		17	55591584-55
0.24820693	1	191341814	51022	GLRX2		17	55591584-55
0.31222259	1	32488799	3932	LCK		12	4284480-428
0.29659057	5	76284393	1393	CRHBP		9	93211451-93
0.40911014	19	56582398	3982	LIM2		5	145949378-1
0.3303663	19	56582398	3982	LIM2		19	34858040-34
0.24298986	7	99654750	79037	MGC2463		20	3734490-373
0.25485707	2	241456532	189	AGXT		16	56277117-56
0.27961958	10	129594939	5791	PTPRE		9	93211451-93
0.32686918	16	30582734	64319	FBS1		2	86865508-86
0.27544425	5	76284393	1393	CRHBP		12	94891379-94
0.28668276	19	3679751	27134	TJP3		1	117113051-1
0.27046173	10	134108411	170393	C10orf91		5	10514353-10
0.26784267	2	85774949	10578	GNLY		5	145949378-1
0.2639514	15	89248753	4122	MAN2A2		5	10514353-10

0.37780028	4	47830991	7294	TXK		17	35963692-35
0.28786091	11	6823856	144124	OR10A5		1	117113051-1
0.25052536	7	99808438	29992	PILRA		17	55591584-55
0.25793546	16	56563625	1258	CNGB1		5	145949378-1
0.29776963	7	99654750	79037	MGC2463		1	117113051-1
0.34912882	1	32488799	3932	LCK		14	61087117-61
0.33971779	19	56582398	3982	LIM2		6	130507497-1
0.29068008	1	32488799	3932	LCK		1	117113051-1
0.29363779	12	54646332	1017	CDK2		11	77604355-77
0.25705035	12	6355798	6337	SCNN1A		1	219054033-2
0.34924096	3	35658819	10777	ARPP-21		11	60544218-60
0.25939499	19	13120872	9592	IER2		11	77604355-77
0.25100823	19	13120872	9592	IER2		19	56941267-56
0.2748105	11	808917	57104	PNPLA2		5	10514353-10
0.29180934	11	808917	57104	PNPLA2		6	41351742-41
0.28590097	11	33870664	4005	LMO2		9	93211451-93
0.24854588	19	56582398	3982	LIM2		11	85083465-85
0.26000784	1	191341814	51022	GLRX2		16	56277117-56
0.35234968	10	85922743	387695	C10orf99		22	30007802-30
0.25641794	10	134108411	170393	C10orf91		22	35601693-35
0.29400296	11	33870664	4005	LMO2		5	10514353-10
0.27040948	19	56582398	3982	LIM2		4	57209382-57
0.27086369	12	54646332	1017	CDK2		19	56941267-56
0.25703492	19	649371	400668	PRSSL1		11	77604355-77
0.26105136	10	134108411	170393	C10orf91		6	41351742-41
0.32040437	12	53328560	117159	DCD		5	145949378-1
0.24611328	20	43316404	6590	SLPI		19	4488310-448
0.26111645	11	808917	57104	PNPLA2		22	35601693-35
0.27700896	10	97505212	953	ENTPD1		9	93211451-93
0.36127734	1	32488799	3932	LCK		3	112853511-1
0.35112679	10	102722214	57715	SEMA4G		1	117113051-1
0.30738418	14	24173141	3002	GZMB		5	145949378-1
0.34974533	6	35873567	1208	CLPS		1	117113051-1
0.25256308	10	102722214	57715	SEMA4G		11	60544218-60
0.29852583	12	51886921	3695	ITGB7		11	60544218-60
0.29425996	16	30582734	64319	FBS1		2	86865488-86
0.26076115	10	134108411	170393	C10orf91		11	77604355-77
0.33712176	19	56582398	3982	LIM2		17	31222639-31
0.2837131	14	20493306	6036	RNASE2		5	10514353-10
0.27317721	11	808917	57104	PNPLA2		11	77604355-77
0.25186649	12	13139815	83445	GSG1		16	56277117-56
0.24014738	14	20429255	6037	RNASE3	X		17075527-17
0.2374903	19	4254012	126259	MGC23244		20	3734490-373
0.26978796	3	189379299	401105	FLJ42393		11	60544218-60

0.29195505	10	102722214	57715	SEMA4G		16	3059568-305
0.26117543	10	129594939	5791	PTPRE		11	77604355-77
0.34820035	1	32488799	3932	LCK		5	156754720-1
0.2557966	17	58239624	162333	RNF190		20	3734490-373
0.28351146	3	49566012	8927	BSN		11	77604355-77
0.30342445	19	13120872	9592	IER2		12	105156317-1
0.31717252	19	3711955	9546	APBA3		11	60544218-60
0.27824871	9	138676375	51162	EGFL7		9	93211451-93
0.2572339	11	33870664	4005	LMO2		22	35601693-35
0.26193801	1	25164659	864	RUNX3		5	145949378-1
0.26998218	14	24173141	3002	GZMB		17	31222639-31
0.27233258	11	56951601	29015	SLC43A3		5	10514353-10
0.29167487	14	24173141	3002	GZMB		8	102768224-1
0.26097625	11	808917	57104	PNPLA2	X		17063402-17
0.30274196	12	51886921	3695	ITGB7		20	3734490-373
0.25093237	3	39297107	1524	CX3CR1		8	102768224-1
0.26590699	1	159436198	4720	NDUFS2		11	77604355-77
0.25877931	3	133518757	55	ACPP		1	219054033-2
0.24505836	11	808892	57104	PNPLA2		16	56277117-56
0.26394194	3	152529997	53829	P2RY13	X		17075527-17
0.27875476	19	13120872	9592	IER2		4	113581955-1
0.25757718	14	20429255	6037	RNASE3		5	10514353-10
0.32195699	19	56582398	3982	LIM2		8	102768224-1
0.25673001	3	35658823	10777	ARPP-21		11	60544218-60
0.38526482	4	47831037	7294	TXK		17	35963692-35
0.23559746	5	6685086	6715	SRD5A1	X		17075527-17
0.24411284	3	152529997	53829	P2RY13		6	133085777-1
0.29834776	1	25164659	864	RUNX3		1	117113051-1
0.27658343	12	52975545	4778	NFE2		5	10514353-10
0.34707597	12	51886921	3695	ITGB7		1	117113051-1
0.27687992	1	196874171	5788	PTPRC		11	60544218-60
0.2716569	19	46992652	1084	CEACAM3		11	77604355-77
0.28116267	19	5802504	2525	FUT3		5	10514353-10
0.24357892	4	109034053	166929	MGC26963	X		17075527-17
0.28969932	3	133518757	55	ACPP	X		17075527-17
0.27380669	22	35875369	3560	IL2RB		11	60544218-60
0.26466453	11	59708133	64231	MS4A6A		1	219054033-2
0.26172468	14	20493306	6036	RNASE2		11	77604355-77
0.29211323	12	67643600	1368	CPM		9	93211451-93
0.31425795	3	52249091	11344	PTK9L		1	117113051-1
0.27056349	1	156526550	911	CD1C		11	60544218-60
0.29100361	20	43316404	6590	SLPI		11	77604355-77
0.27334143	2	242594120	285097	FLJ38379		11	60544218-60
0.28226069	7	141293159	23601	CLEC5A		5	10514353-10

0.25555048	12	123569171	9612	NCOR2		5 10514353-10
0.26609499	12	13139815	83445	GSG1		2 71763245-71
0.25540062	19	43995615	3960	LGALS4		22 35601693-35
0.26355104	17	44641801	2793	GNGT2		11 60544218-60
0.24854248	7	73584338	80112	WBSCR23		5 145949378-1
0.24048188	9	129951613	3934	LCN2	X	17075527-17
0.35505	10	98054352	1791	DNTT		11 60544218-60
0.26545179	1	191341814	51022	GLRX2		1 219054033-2
0.2551861	14	20429255	6037	RNASE3		11 77604355-77
0.25777775	20	43316404	6590	SLPI		5 10514353-10
0.2676682	4	109034053	166929	MGC26963		22 35601693-35
0.25498073	12	6355798	6337	SCNN1A		5 10514353-10
0.36710772	3	35658819	10777	ARPP-21		17 18864772-18
0.28227695	4	109034053	166929	MGC26963		1 33562558-33
0.31743718	10	72032298	5551	PRF1		11 60544218-60
0.27058629	14	22658002	1053	CEBPE		5 10514353-10
0.27501933	1	152067323	57459	GATAD2B		5 10514353-10
0.25457417	1	36721568	1441	CSF3R		19 4488310-448
0.24919791	17	15109383	5376	PMP22		16 3059568-305
0.26421006	16	27319997	50615	IL21R		5 10514353-10
0.34665146	22	35875369	3560	IL2RB		1 117113051-1
0.26740191	14	22658002	1053	CEBPE		22 35601693-35
0.29093256	7	73584338	80112	WBSCR23		1 117113051-1
0.26838326	19	38378230	4037	LRP3		11 77604355-77
0.27134327	4	47830991	7294	TXK		11 60544218-60
0.2498199	1	205037818	29949	IL19		1 219054033-2
0.27002762	3	35658819	10777	ARPP-21		20 3734490-373
0.26084325	16	27319997	50615	IL21R		22 35601693-35
0.27617349	3	152529997	53829	P2RY13		1 33562558-33
0.29973544	2	74913110	3099	HK2		5 10514353-10
0.23979724	20	31287206	51297	PLUNC		16 56277117-56
0.28381591	14	103164372	3831	KNS2		22 35601693-35
0.24647194	3	35658823	10777	ARPP-21		20 3734490-373
0.26056296	12	13139815	83445	GSG1		1 219054033-2
0.27313292	22	35875369	3560	IL2RB		20 3734490-373
0.29506678	2	241456532	189	AGXT		9 93211451-93
0.29430624	3	133518757	55	ACPP		22 35601693-35
0.33156528	3	35658819	10777	ARPP-21		1 205144550-2
0.26908999	12	51886921	3695	ITGB7		10 73488877-73
0.23699709	1	36721568	1441	CSF3R		1 219054033-2
0.30462326	11	59708133	64231	MS4A6A		9 93211451-93
0.24270797	19	4490943	116844	LRG1		1 219054033-2
0.27483834	3	152529997	53829	P2RY13		11 77604355-77
0.27566783	10	97505362	953	ENTPD1		5 10514353-10

0.28372686	3	127720063	131669	UROC1		1 117113051-1
0.28124241	6	41362863	54210	TREM1		9 93211451-93
0.28469056	3	133518757	55	ACPP		5 10514353-10
0.27558	19	3711955	9546	APBA3		20 3734490-373
0.27151118	14	103164372	3831	KNS2		5 10514353-10
0.3455161	1	32488799	3932	LCK		6 52236896-52
0.25169554	2	230798884	11262	SP140		11 60544218-60
0.24548246	2	74913110	3099	HK2	X	17075527-17
0.29829817	2	241456532	189	AGXT		22 35601693-35
0.38790843	3	51679391	51368	TEX264		6 31913122-31
0.24680278	20	43316404	6590	SLPI		6 41351742-41
0.33314626	14	99601789	51466	EVL		11 60544218-60
0.26175947	4	109034053	166929	MGC26963		11 77604355-77
0.27223321	1	36721568	1441	CSF3R	X	17075527-17
0.24380275	5	76284505	1393	CRHBP		1 219054033-2
0.25146737	10	134108411	170393	C10orf91		10 99070864-99
0.28291881	12	67643600	1368	CPM		11 77604355-77
0.23705509	14	24115961	1511	CTSG		1 219054033-2
0.26832663	19	779170	566	AZU1		5 10514353-10
0.32893156	19	3711955	9546	APBA3		1 117113051-1
0.29446752	12	52977085	4778	NFE2		5 10514353-10
0.25228268	12	52977085	4778	NFE2	X	17075527-17
0.28768663	17	74487947	3959	LGALS3BP		11 60544218-60
0.2452609	2	241456532	189	AGXT	X	17075527-17
0.25857789	16	27319997	50615	IL21R		11 77604355-77
0.30308513	1	36721568	1441	CSF3R		5 10514353-10
0.30053919	4	47831037	7294	TXK		5 35912241-35
0.31004184	2	108971950	10913	EDAR		11 60544218-60
0.25346535	12	67643600	1368	CPM		22 35601693-35
0.24715493	17	44641801	2793	GNGT2		20 3734490-373
0.24464063	16	27319997	50615	IL21R		12 12373772-12
0.35990538	4	47830991	7294	TXK		22 30007802-30
0.27381566	2	74913110	3099	HK2		22 35601693-35
0.28156411	2	241456532	189	AGXT		5 10514353-10
0.28423925	3	133518757	55	ACPP		1 33562558-33
0.35470135	4	47830991	7294	TXK		17 18864772-18
0.26172381	1	152067323	57459	GATAD2B		11 77604355-77
0.27985772	17	77583552	5881	RAC3		19 59290134-59
0.24781954	19	6717609	10045	SH2D3A		11 60544218-60
0.36589431	17	77868065	924	CD7		17 35963692-35
0.30445518	10	72032298	5551	PRF1		20 3734490-373
0.26376276	3	152529997	53829	P2RY13		6 133087707-1
0.33227298	19	495349	3004	GZMM		1 117113051-1
0.26483783	22	35875369	3560	IL2RB		10 73488877-73

0.25644774	1	191341814	51022	GLRX2	X		17075527-17
0.25657436	3	35658819	10777	ARPP-21		10	73488877-73
0.31541536	12	13139815	83445	GSG1		22	35601693-35
0.3192573	10	72032815	5551	PRF1		11	60544218-60
0.31207222	5	76284505	1393	CRHBP		22	35601693-35
0.25114891	3	152529997	53829	P2RY13	X		17063402-17
0.2936091	1	205037818	29949	IL19		5	10514353-10
0.28133432	15	76701339	1136	CHRNA3		11	60544218-60
0.26063307	1	24386999	163702	IL28RA		5	10514353-10
0.29984815	10	73519632	9806	SPOCK2		11	60544218-60
0.28764922	3	45959946	10663	CXCR6		11	60544218-60
0.29493089	1	191341814	51022	GLRX2		22	35601693-35
0.27324529	19	56582398	3982	LIM2		1	25098722-25
0.25720306	3	133518757	55	ACPP		19	48852486-48
0.35572678	10	72032298	5551	PRF1		1	117113051-1
0.27795707	16	67072	4350	MPG		19	59290134-59
0.26405444	16	3055553	9235	IL32		11	60544218-60
0.24058928	4	109034053	166929	MGC26963	X		17063402-17
0.29011376	1	191341814	51022	GLRX2		5	10514353-10
0.2860246	9	124837105	2844	GPR21		22	35601693-35
0.24698089	17	44643501	51225	ABI3		20	3734490-373
0.27529277	3	133518757	55	ACPP		11	77604355-77
0.24912081	19	13120872	9592	IER2		7	97758418-97
0.30130221	11	59708133	64231	MS4A6A		11	77604355-77
0.38452055	11	117680841	916	CD3E		11	60544218-60
0.2855405	3	35658819	10777	ARPP-21		11	60651626-60
0.24388565	12	67643600	1368	CPM		12	12373772-12
0.28093442	9	124837105	2844	GPR21		5	10514353-10
0.25820921	13	50537954	2974	GUCY1B2		5	10514353-10
0.29056751	16	67932165	84342	COG8		1	117113051-1
0.25604061	14	20429255	6037	RNASE3		4	140265851-1
0.26595759	3	35658823	10777	ARPP-21		11	60651626-60
0.28073961	16	70598877	1723	DHODH		9	93211451-93
0.26503028	15	27001152	321	APBA2		11	60544218-60
0.29890688	12	13139815	83445	GSG1		5	10514353-10
0.27587028	2	241456532	189	AGXT		6	41351742-41
0.28311855	2	241456532	189	AGXT		11	77604355-77
0.2567435	13	50537954	2974	GUCY1B2		22	35601693-35
0.29321264	16	56258962	222487	GPR97		5	10514353-10
0.25350419	5	76284505	1393	CRHBP	X		17075527-17
0.30931793	17	3407893	162514	TRPV3		1	117113051-1
0.26559423	7	75420817	5447	POR		1	219054033-2
0.25756812	1	151630204	6279	S100A8	X		17075527-17
0.2924546	5	76284505	1393	CRHBP		5	10514353-10

0.27489444	1	36721568	1441	CSF3R		22	35601693-35
0.24223992	1	156415852	912	CD1D		4	140265851-1
0.23803664	10	44815977	7570	ZNF22		1	219054033-2
0.28423764	16	70598877	1723	DHODH		5	10514353-10
0.2692222	2	241456532	189	AGXT		19	56941267-56
0.25087714	2	242594120	285097	FLJ38379		10	73488877-73
0.26599641	1	170894886	356	FASLG		11	60544218-60
0.2393983	8	95291196	1015	CDH17		8	102768224-1
0.28515972	22	38627772	9402	GRAP2		1	117113051-1
0.30229839	10	85922743	387695	C10orf99		9	129743305-1
0.25376262	19	3711955	9546	APBA3		10	73488877-73
0.29701977	15	27001150	321	APBA2		11	60544218-60
0.30515037	12	13139815	83445	GSG1		1	33562558-33
0.3156547	1	153253136	51043	ZBTB7B		17	18864772-18
0.26217856	17	24069170	83871	RAB34		6	41351742-41
0.23702405	11	33264921	10114	HIPK3		1	219054033-2
0.27036687	9	126992712	5537	PPP6C		11	60544218-60
0.25047586	3	133518757	55	ACPP		7	99825517-99
0.24186071	11	56951601	29015	SLC43A3		16	1928341-192
0.29149412	17	44643501	51225	ABI3		1	117113051-1
0.26949758	20	43316404	6590	SLPI		4	140265851-1
0.24461219	12	13139815	83445	GSG1	X		17075527-17
0.25648755	1	245648040	114548	CIAS1		11	77604355-77
0.24948903	10	129594939	5791	PTPRE		20	30153066-30
0.25774812	2	106047843	84417	ECRG4		1	219054033-2
0.28548648	7	99808438	29992	PILRA		22	35601693-35
0.25972612	16	31178510	3684	ITGAM		22	35601693-35
0.37269613	11	117718540	915	CD3D		11	60544218-60
0.2720823	19	46951235	4680	CEACAM6		5	10514353-10
0.27734955	1	36721568	1441	CSF3R		1	33562558-33
0.28439412	1	36721568	1441	CSF3R		11	77604355-77
0.26241059	19	56582398	3982	LIM2		2	120451383-1
0.2942199	19	40895672	27033	ZBTB32		11	60544218-60
0.2401758	7	99808438	29992	PILRA	X		17075527-17
0.25729732	6	41362863	54210	TREM1		11	77604355-77
0.25063373	19	56567263	4818	NKG7		19	4488310-448
0.26463059	1	205037818	29949	IL19		22	35601693-35
0.25389426	16	31178510	3684	ITGAM		5	10514353-10
0.25228771	19	40512021	933	CD22		6	41351742-41
0.27396877	9	124837105	2844	GPR21		1	33562558-33
0.25856141	19	56582398	3982	LIM2		1	157213164-1
0.28976456	5	76284505	1393	CRHBP		1	33562558-33
0.31134788	1	17506268	23569	PADI4		1	117113051-1
0.28407249	10	72032298	5551	PRF1		10	73488877-73

0.28913523	5	76284505	1393	CRHBP		19	56941267-56
0.24825052	3	152529997	53829	P2RY13		19	6615860-661
0.26895333	14	24115961	1511	CTSG		5	10514353-10
0.2768891	19	56582398	3982	LIM2		8	102767973-1
0.2563729	12	52977085	4778	NFE2		6	41351742-41
0.23498673	14	24173141	3002	GZMB		8	102767973-1
0.27096629	16	2226602	1775	DNASE1L2		19	59290134-59
0.33946857	2	231499057	9290	GPR55		1	117113051-1
0.26719789	14	24115961	1511	CTSG		22	35601693-35
0.27056264	3	152529997	53829	P2RY13		4	140265851-1
0.25793344	19	40321946	5348	FXYD1		5	10514353-10
0.26562718	1	36721568	1441	CSF3R		22	35603819-35
0.25424173	19	4490943	116844	LRG1		5	10514353-10
0.26513446	16	56258962	222487	GPR97		22	35601693-35
0.27769497	2	230798884	11262	SP140		1	117113051-1
0.3160945	17	77868065	924	CD7		11	60544218-60
0.26553583	17	1611970	5176	SERPINF1		5	10514353-10
0.25972733	16	70598877	1723	DHODH		22	35601693-35
0.35848416	11	117720322	917	CD3G		11	60544218-60
0.38079989	4	47831037	7294	TXK		22	30007802-30
0.26848821	16	30390788	3683	ITGAL		11	60544218-60
0.25562761	6	31648435	4049	LTA		20	3734490-373
0.2444309	1	36721568	1441	CSF3R		19	48852486-48
0.27883433	5	176769301	2161	F12		5	10514353-10
0.27245554	1	36721568	1441	CSF3R	X		17063402-17
0.27922492	5	134763443	9555	H2AFY		19	59290134-59
0.37531431	4	47831037	7294	TXK		17	18864772-18
0.2521801	2	241456532	189	AGXT	X		17063402-17
0.25720993	1	191341814	51022	GLRX2		12	12373772-12
0.24645705	22	25211231	89781	HPS4		10	99071131-99
0.3066125	3	133518757	55	ACPP		4	113581955-1
0.27097707	19	40321946	5348	FXYD1		11	77604355-77
0.2593037	1	191341814	51022	GLRX2		7	99825517-99
0.2465002	10	44815977	7570	ZNF22	X		17075527-17
0.25282816	9	124837105	2844	GPR21		19	56941267-56
0.26089353	1	170894886	356	FASLG		20	3734490-373
0.24838477	1	36721568	1441	CSF3R		12	12373772-12
0.36887869	19	40895672	27033	ZBTB32		1	117113051-1
0.27971812	19	6717609	10045	SH2D3A		11	60651626-60
0.24131168	3	133518757	55	ACPP		6	133087707-1
0.27651826	14	99601789	51466	EVL		20	3734490-373
0.25544918	17	1611970	5176	SERPINF1		22	35601693-35
0.27117715	1	191341814	51022	GLRX2	X		17063402-17
0.26015069	10	98054352	1791	DNTT		20	3734490-373

0.24279855	3	123526489	1475	CSTA	X		17075527-17
0.24222278	19	4490943	116844	LRG1		19	48852486-48
0.24797052	12	32546301	121512	FGD4		4	140265851-1
0.26759156	4	47830991	7294	TXK		11	60651626-60
0.25742701	16	56258956	222487	GPR97		5	10514353-10
0.34614626	3	45959946	10663	CXCR6		17	18864772-18
0.26728655	16	70598877	1723	DHODH		11	77604355-77
0.27023967	11	808892	57104	PNPLA2		5	10514353-10
0.25317856	15	76701339	1136	CHRNA3		20	3734490-373
0.27178379	11	808892	57104	PNPLA2		22	35601693-35
0.26713269	12	13139815	83445	GSG1		22	35603819-35
0.27123076	5	76284505	1393	CRHBP		11	77604355-77
0.26081241	3	133518757	55	ACPP		12	14548081-14
0.28487699	12	53043844	53831	GPR84		9	93211451-93
0.26062476	10	81699171	6441	SFTPD		5	10514353-10
0.23671639	1	191341814	51022	GLRX2		19	48852486-48
0.2474704	13	114064381	65110	UPF3A		11	60544218-60
0.26076765	10	81699171	6441	SFTPD		22	35601693-35
0.32622173	4	47831037	7294	TXK		1	6444014-644
0.26174256	12	13139815	83445	GSG1		19	56941267-56
0.27866297	11	33264921	10114	HIPK3		5	10514353-10
0.24419014	12	52977085	4778	NFE2	X		17063402-17
0.25754157	12	6179035	928	CD9		22	35601693-35
0.30097905	7	75420817	5447	POR		5	10514353-10
0.27565975	3	123526489	1475	CSTA		22	35601693-35
0.25377917	1	191341814	51022	GLRX2		11	77604355-77
0.28923348	12	53043844	53831	GPR84		5	10514353-10
0.24743091	16	70598877	1723	DHODH		12	12373772-12
0.26209534	14	22658002	1053	CEBPE		10	99070864-99
0.23628234	17	3743685	84254	CAMKK1		20	30153066-30
0.28786731	10	72032298	5551	PRF1		11	60651626-60
0.27665395	10	72032815	5551	PRF1		20	3734490-373
0.24289982	3	133518757	55	ACPP		19	12764585-12
0.26275291	19	52530972	27202	GPR77		11	77604355-77
0.24443932	11	808917	57104	PNPLA2		10	97616172-97
0.30962828	10	98054352	1791	DNTT		1	117113051-1
0.26358722	17	1611970	5176	SERPINF1		11	77604355-77
0.25879219	1	151630204	6279	S100A8		19	56941267-56
0.38316414	11	117718482	915	CD3D		11	60544218-60
0.2432376	20	43316404	6590	SLPI		10	99070864-99
0.26374897	12	13139815	83445	GSG1		11	77604355-77
0.25769893	14	24115961	1511	CTSG		11	77604355-77
0.24217703	9	129951613	3934	LCN2		4	140265851-1
0.25615191	2	106047843	84417	ECRG4	X		17075527-17

0.31036966	2	241456532	189	AGXT		12	105156317-1
0.25318929	19	46951235	4680	CEACAM6		11	77604355-77
0.26643218	3	123526489	1475	CSTA		5	10514353-10
0.26221006	7	99808438	29992	PILRA		11	77604355-77
0.2463708	5	76284505	1393	CRHBP		12	12373772-12
0.28479768	1	170894643	356	FASLG		11	60544218-60
0.2409748	12	53043844	53831	GPR84	X		17075527-17
0.26951352	11	33264921	10114	HIPK3		22	35601693-35
0.29087881	7	75420817	5447	POR		22	35601693-35
0.25176342	3	152529997	53829	P2RY13		10	99070864-99
0.27948552	14	99601789	51466	EVL		10	73488877-73
0.27927133	12	53043844	53831	GPR84		22	35601693-35
0.27273655	17	72827828	10801	SEPT9		5	10514353-10
0.33712354	20	34707347	84174	SLA2		11	60544218-60
0.25724778	5	76284505	1393	CRHBP	X		17063402-17
0.25900139	10	44815977	7570	ZNF22		22	35601693-35
0.24133847	7	75420817	5447	POR	X		17075527-17
0.25399583	1	151630204	6279	S100A8		11	77604355-77
0.29166102	15	76701339	1136	CHRNA3		11	60651626-60
0.23649015	2	46600883	90423	ATP6V1E2		1	219054033-2
0.26003488	11	808892	57104	PNPLA2		19	56941267-56
0.23854669	2	108971950	10913	EDAR		20	3734490-373
0.26993839	17	72827828	10801	SEPT9		22	35601693-35
0.24765382	12	123569171	9612	NCOR2		20	30153066-30
0.26680221	11	808892	57104	PNPLA2		11	77604355-77
0.25514298	3	35658819	10777	ARPP-21		7	149208621-1
0.29301126	12	53043844	53831	GPR84		11	77604355-77
0.25073611	13	114064381	65110	UPF3A		20	3734490-373
0.25928511	19	5802504	2525	FUT3		20	30153066-30
0.26957548	19	59258650	284415	UNQ3033		5	10514353-10
0.35934348	10	72032298	5551	PRF1		3	112853511-1
0.27967121	2	106047843	84417	ECRG4		22	35601693-35
0.24513255	10	97505362	953	ENTPD1		4	140265851-1
0.25332673	16	30390788	3683	ITGAL		20	3734490-373
0.26503069	19	880046	1820	ARID3A		5	10514353-10
0.28591635	12	13139815	83445	GSG1		11	61488158-61
0.26627538	19	15252927	23476	BRD4		11	77604355-77
0.24789177	12	13139815	83445	GSG1	X		17063402-17
0.28653495	2	241456532	189	AGXT		10	99070864-99
0.2521091	1	245648040	114548	CIAS1		4	140265851-1
0.3233199	14	99601789	51466	EVL		17	18864772-18
0.26834524	14	22375781	4323	MMP14		19	59290134-59
0.30038408	2	242450682	5133	PDCD1		11	60544218-60
0.27929856	4	47830991	7294	TXK		7	149208621-1

0.25250896	13	31787023	675	BRCA2	X		17075527-17
0.29907176	14	99601789	51466	EVL		1	117113051-1
0.25753769	19	40895672	27033	ZBTB32		20	3734490-373
0.27189522	2	106047843	84417	ECRG4		5	10514353-10
0.32068761	3	112796876	79413	ZBED2		1	117113051-1
0.25121876	12	13139815	83445	GSG1		6	133087707-1
0.24264932	11	56951601	29015	SLC43A3		7	97758418-97
0.25701536	2	242594120	285097	FLJ38379		7	149208621-1
0.24685837	11	808892	57104	PNPLA2		6	41351742-41
0.26866467	15	38388759	5330	PLCB2		5	10514353-10
0.24666241	3	133518757	55	ACPP		4	140265851-1
0.2775474	1	170894886	356	FASLG		11	60651626-60
0.25286637	14	103164372	3831	KNS2		10	99070864-99
0.28121728	3	45959946	10663	CXCR6		11	60651626-60
0.25836175	11	808892	57104	PNPLA2	X		17063402-17
0.25795604	10	72032298	5551	PRF1		1	165666584-1
0.24955455	6	41362863	54210	TREM1		4	140265851-1
0.24027323	1	8195939	50651	SLC45A1	X		17075527-17
0.24231876	1	36721568	1441	CSF3R		10	99071131-99
0.32807681	9	35640561	27240	SIT1		11	60544218-60
0.25677497	19	56567263	4818	NKG7		5	10514353-10
0.24506713	1	156526550	911	CD1C		7	149208621-1
0.28716498	14	99601789	51466	EVL		11	60651626-60
0.2530459	7	99808438	29992	PILRA		19	48842368-48
0.29553455	11	117680841	916	CD3E		20	3734490-373
0.27472475	1	8195939	50651	SLC45A1		5	10514353-10
0.2795801	1	24386999	163702	IL28RA		19	59290134-59
0.24104377	4	109034053	166929	MGC26963		20	30153066-30
0.31907626	12	121781072	27198	GPR81		11	60544218-60
0.27015447	10	98054352	1791	DNTT		11	60651626-60
0.29339824	1	205037818	29949	IL19		12	105156317-1
0.3880019	4	47831037	7294	TXK		6	31913122-31
0.33158774	12	51886921	3695	ITGB7		6	52236896-52
0.27611726	1	36721568	1441	CSF3R		4	113581955-1
0.24236129	2	241456532	189	AGXT		7	99835352-99
0.30245923	16	30390788	3683	ITGAL		1	205144550-2
0.24328832	11	808917	57104	PNPLA2	X		24462036-24
0.25001001	4	47831037	7294	TXK		10	73488877-73
0.2806619	3	133518757	55	ACPP		20	30153066-30
0.26645763	1	205037818	29949	IL19		4	140265851-1
0.26875877	1	8195939	50651	SLC45A1		22	35601693-35
0.27075552	1	159867677	2215	FCGR3B		5	10514353-10
0.25465222	19	649371	400668	PRSSL1		1	44216369-44
0.30576381	11	117720322	917	CD3G		20	3734490-373

0.34629388	4	47831037	7294	TXK		12	45916614-45
0.35064078	17	77868065	924	CD7		17	18864772-18
0.26459941	1	191341814	51022	GLRX2		4	140265851-1
0.31511463	5	76284505	1393	CRHBP		19	59290134-59
0.25269899	12	53043844	53831	GPR84		19	56941267-56
0.35028135	11	117680841	916	CD3E		11	60651626-60
0.24399992	3	133518757	55	ACPP		10	99070864-99
0.23556378	5	176769301	2161	F12	X		17063402-17
0.29799802	12	13139815	83445	GSG1		10	99070864-99
0.25375554	6	41238895	54209	TREM2		16	1928341-192
0.24827137	11	59708133	64231	MS4A6A		4	140265851-1
0.26530242	7	75420817	5447	POR		11	77604355-77
0.25039018	11	33264921	10114	HIPK3	X		17063402-17
0.26452223	1	170894643	356	FASLG		20	3734490-373
0.2536144	17	72827828	10801	SEPT9		11	77604355-77
0.25664562	19	880046	1820	ARID3A		11	77604355-77
0.27113215	1	36721568	1441	CSF3R		10	99070864-99
0.26690276	1	191341814	51022	GLRX2		13	30236347-30
0.26398899	10	72032298	5551	PRF1		6	33772190-33
0.34365342	22	35875369	3560	IL2RB		6	52236896-52
0.23601483	15	72005833	4016	LOXL1		20	30153066-30
0.36036189	4	47831037	7294	TXK		0	0
0.35865565	11	117720322	917	CD3G		1	117113051-1
0.28085068	12	13139815	83445	GSG1		4	113581955-1
0.26995256	12	13139815	83445	GSG1		1	27054486-27
0.28932343	3	133518757	55	ACPP		7	97758418-97
0.28474	11	117718540	915	CD3D		20	3734490-373
0.25403166	17	77868065	924	CD7		20	3734490-373
0.32309808	11	118259745	643	BLR1		1	205144550-2
0.26081067	19	649371	400668	PRSSL1		3	12914665-12
0.2546799	1	36721568	1441	CSF3R		13	30236347-30
0.27634923	16	70598877	1723	DHODH		12	105156317-1
0.23546218	3	133518757	55	ACPP		3	48530189-48
0.24797947	19	4490943	116844	LRG1		4	140265851-1
0.25450399	1	159867677	2215	FCGR3B		22	35601693-35
0.26545533	1	191341814	51022	GLRX2		10	99070864-99
0.24089585	19	56567263	4818	NKG7		22	35603819-35
0.25020139	16	56258962	222487	GPR97		7	99835352-99
0.25629685	7	75420817	5447	POR	X		17063402-17
0.25034419	16	70598877	1723	DHODH		4	140265851-1
0.27565213	5	76284505	1393	CRHBP		10	99070864-99
0.33611983	17	77868065	924	CD7		22	30007802-30
0.26454994	2	46600883	90423	ATP6V1E2		5	10514353-10
0.3358127	11	117718540	915	CD3D		1	117113051-1

0.26582519	10	72032815	5551	PRF1		11	60651626-60
0.29533618	16	30582734	64319	FBS1		1	117113051-1
0.33781212	11	117718540	915	CD3D		11	60651626-60
0.24163972	10	97505362	953	ENTPD1		20	30153066-30
0.26773732	12	54646332	1017	CDK2		3	12914665-12
0.25323934	12	13139815	83445	GSG1		4	140265851-1
0.25238762	20	43316404	6590	SLPI		17	77790597-77
0.33631548	11	117680841	916	CD3E		17	18864772-18
0.27751256	7	50816909	2887	GRB10		1	24671778-24
0.29212103	11	117720322	917	CD3G		10	73488877-73
0.25956527	15	27001150	321	APBA2		1	165666584-1
0.27225341	11	117680841	916	CD3E		10	73488877-73
0.28023128	1	32489589	3932	LCK		11	60544218-60
0.25959058	1	170894886	356	FASLG		6	33772190-33
0.27853646	3	45959946	10663	CXCR6		7	149208621-1
0.3791074	9	35640561	27240	SIT1		14	98705794-98
0.2782198	11	33264921	10114	HIPK3		4	140265851-1
0.24593385	11	808917	57104	PNPLA2		1	44216369-44
0.24274401	2	74913110	3099	HK2		20	30153066-30
0.24435995	10	129594939	5791	PTPRE		1	44216369-44
0.25646392	19	56567263	4818	NKG7		19	48842368-48
0.28528061	20	34707347	84174	SLA2		20	3734490-373
0.25479093	6	31690816	199	AIF1		5	10514353-10
0.26276175	12	13139815	83445	GSG1		12	105156317-1
0.26933438	11	71524436	2352	FOLR3		5	10514353-10
0.24067644	2	106047843	84417	ECRG4	X		17063402-17
0.25556227	12	52977085	4778	NFE2		20	30153066-30
0.25476723	6	31690816	199	AIF1		22	35601693-35
0.25692633	17	1611970	5176	SERPINF1		10	99070864-99
0.27714183	11	117718540	915	CD3D		10	73488877-73
0.31123766	11	118259745	643	BLR1		17	18864772-18
0.30067471	11	117680841	916	CD3E		1	117113051-1
0.28540096	1	202001128	54900	LAX1		6	52236896-52
0.31235598	1	170894643	356	FASLG		17	18864772-18
0.26229838	1	36721568	1441	CSF3R		20	30153066-30
0.28800379	4	47831037	7294	TXK		7	149208621-1
0.24049737	1	191341814	51022	GLRX2		17	8311528-831
0.2876875	19	56582398	3982	LIM2		11	133754175-1
0.27568304	11	59708133	64231	MS4A6A		7	97758418-97
0.27690792	16	56258962	222487	GPR97		20	30153066-30
0.31585981	11	117720322	917	CD3G		11	60651626-60
0.25601862	11	63729582	83706	URP2		5	10514353-10
0.29080377	10	72032298	5551	PRF1	X		51661708-51
0.33489319	20	34707347	84174	SLA2		1	117113051-1

0.32939568	11	117718540	915	CD3D		17	18864772-18
0.26627194	5	176769301	2161	F12		12	105156317-1
0.25860153	11	71524436	2352	FOLR3		22	35601693-35
0.27109547	10	72032815	5551	PRF1		6	33772190-33
0.33095244	11	117720322	917	CD3G		17	18864772-18
0.24462873	3	152529997	53829	P2RY13		3	128882396-1
0.26229586	5	76284393	1393	CRHBP		3	12914665-12
0.29774334	11	56951601	29015	SLC43A3		19	41090225-41
0.27416752	17	77868065	924	CD7		11	60651626-60
0.27661055	1	170894643	356	FASLG		1	117113051-1
0.23900307	1	8195939	50651	SLC45A1	X		17063402-17
0.28834475	11	117718482	915	CD3D		20	3734490-373
0.25761945	11	808892	57104	PNPLA2		10	99070864-99
0.34928402	11	117718482	915	CD3D		1	117113051-1
0.26254687	10	73519632	9806	SPOCK2		7	149208621-1
0.24788878	3	123526489	1475	CSTA		4	140265851-1
0.28352843	17	72827828	10801	SEPT9		12	105156317-1
0.31074893	3	35658819	10777	ARPP-21		14	19995423-19
0.24067781	20	43316404	6590	SLPI	X		24462036-24
0.28940821	1	159032429	4063	LY9		11	60544218-60
0.24306752	14	64480232	2877	GPX2		20	30153066-30
0.31962479	20	34707347	84174	SLA2		11	60651626-60
0.26939118	12	53043844	53831	GPR84		4	113581955-1
0.27851179	19	56582398	3982	LIM2		6	130547006-1
0.25369279	14	99601789	51466	EVL		7	149208621-1
0.24444548	19	56582398	3982	LIM2		6	130547011-1
0.26495225	12	51886921	3695	ITGB7		8	124584719-1
0.23806985	19	5802504	2525	FUT3	X		24462036-24
0.23568983	6	41238895	54209	TREM2		20	30153066-30
0.25562729	2	242450682	5133	PDCD1		10	73488877-73
0.2615461	7	75421357	5447	POR		5	10514353-10
0.2661547	3	189379299	401105	FLJ42393		1	24671778-24
0.26590158	17	58239624	162333	RNF190		19	52951763-52
0.24781436	1	36721568	1441	CSF3R		6	26231711-26
0.29980538	20	43316404	6590	SLPI		19	41090225-41
0.2421328	3	152529997	53829	P2RY13	X		24462036-24
0.24719174	12	53043844	53831	GPR84		4	140265851-1
0.33171161	6	6534074	9450	LY86		17	18864772-18
0.32628786	10	72032298	5551	PRF1		6	52236896-52
0.25009952	7	75420817	5447	POR		7	99835352-99
0.24245944	14	24115961	1511	CTSG		20	30153066-30
0.25831284	10	81699171	6441	SFTPD		20	30153066-30
0.29494722	19	495349	3004	GZMM		6	52236896-52
0.26284565	12	53043844	53831	GPR84		10	99070864-99

0.27699783	6	41238895	54209	TREM2		19	59412606-59
0.29063203	2	241456532	189	AGXT	X		24462036-24
0.24524475	7	99808438	29992	PILRA		20	30153066-30
0.23830828	11	56951601	29015	SLC43A3		1	44216369-44
0.27111921	3	133518757	55	ACPP	X		24462036-24
0.24704312	7	75420817	5447	POR		4	140265851-1
0.33399361	9	35640561	27240	SIT1		17	18864772-18
0.25414857	17	72827828	10801	SEPT9		10	99070864-99
0.28468565	19	3711955	9546	APBA3		8	124584719-1
0.28093103	11	117718482	915	CD3D		10	73488877-73
0.31867103	4	47830991	7294	TXK		1	24671778-24
0.33793808	9	35640561	27240	SIT1		22	30007802-30
0.28963986	17	77868065	924	CD7		7	149208621-1
0.31739479	11	117718482	915	CD3D		11	60651626-60
0.25412124	20	34707347	84174	SLA2		10	73488877-73
0.24367223	9	35640561	27240	SIT1		20	3734490-373
0.24446554	3	133518757	55	ACPP		22	20453432-20
0.30542189	9	35640561	27240	SIT1		1	117113051-1
0.24537695	11	71524436	2352	FOLR3	X		17063402-17
0.33594505	2	242450682	5133	PDCD1		12	45916614-45
0.37767641	11	118259745	643	BLR1		1	23757369-23
0.33361145	1	32489589	3932	LCK		17	18864772-18
0.27712597	3	35658819	10777	ARPP-21		1	24671778-24
0.32343977	12	121781072	27198	GPR81		17	18864772-18
0.29909884	12	121781072	27198	GPR81		1	117113051-1
0.29268207	3	51679391	51368	TEX264		10	71063149-71
0.26474332	17	77868065	924	CD7		6	33772190-33
0.23637534	11	63729582	83706	URP2	X		17063402-17
0.2432673	1	32489589	3932	LCK		20	3734490-373
0.30506423	11	118259745	643	BLR1		9	124911698-1
0.24390537	13	50537954	2974	GUCY1B2		22	20453432-20
0.24237953	19	880046	1820	ARID3A		10	99070864-99
0.30580385	4	47831037	7294	TXK		9	129743305-1
0.2371971	1	36721568	1441	CSF3R		10	97616172-97
0.24205507	17	1611970	5176	SERPINF1		7	97758418-97
0.24581642	7	75420817	5447	POR		10	99070864-99
0.23952579	20	33666135	6676	SPAG4		1	44216369-44
0.29430923	17	3743685	84254	CAMKK1		9	100001298-1
0.28727823	12	121781072	27198	GPR81		11	60651626-60
0.2879034	9	35640561	27240	SIT1		11	60651626-60
0.24996868	9	35640561	27240	SIT1		10	73488877-73
0.25623374	1	32489589	3932	LCK		10	73488877-73
0.26474018	5	76284505	1393	CRHBP		17	77790597-77
0.25684041	12	53043844	53831	GPR84		20	30153066-30

0.28011896	11	117720322	917	CD3G		6	33772190-33
0.2812976	3	133518757	55	ACPP		1	44216369-44
0.26450114	2	106047843	84417	ECRG4		20	30153066-30
0.2712703	11	117680841	916	CD3E		7	149208621-1
0.26056033	12	13139815	83445	GSG1		17	77790597-77
0.25916045	7	75420817	5447	POR		20	30153066-30
0.24784338	19	880046	1820	ARID3A		20	30153066-30
0.25682823	2	242594120	285097	FLJ38379		1	24671778-24
0.29779439	19	59739533	90011	FLJ00060		9	129743305-1
0.25165611	1	159032429	4063	LY9		20	3734490-373
0.25412518	1	36721568	1441	CSF3R	X		24462036-24
0.27873678	11	117720322	917	CD3G		7	149208621-1
0.2498377	3	152529997	53829	P2RY13		3	12914665-12
0.25264466	14	103164372	3831	KNS2		1	44216369-44
0.26839251	11	117718540	915	CD3D		7	149208621-1
0.24196223	1	170894643	356	FASLG		7	149208621-1
0.25323015	1	191341814	51022	GLRX2	X		24462036-24
0.24862071	11	2877365	5002	SLC22A18		1	44216369-44
0.240869	11	59708133	64231	MS4A6A	X		24462036-24
0.25003944	19	4490943	116844	LRG1	X		24462036-24
0.24975895	12	53043844	53831	GPR84		7	97758418-97
0.24197306	12	13139815	83445	GSG1		3	128882396-1
0.28272417	10	98054352	1791	DNTT		6	52236896-52
0.34402351	4	47831037	7294	TXK		1	24671778-24
0.28374086	9	35640561	27240	SIT1		6	33772190-33
0.2897368	11	117718482	915	CD3D		6	33772190-33
0.24088532	11	33870292	4005	LMO2		20	30153066-30
0.29595485	4	47830991	7294	TXK		10	71063149-71
0.2708218	2	46600883	90423	ATP6V1E2		20	30153066-30
0.31669418	1	159032429	4063	LY9		17	18864772-18
0.27960629	10	72032298	5551	PRF1		1	24671778-24
0.29791619	17	77868065	924	CD7		9	129743305-1
0.25817063	20	34707347	84174	SLA2		6	33772190-33
0.30870958	2	108971950	10913	EDAR		1	24671778-24
0.23903909	1	8195939	50651	SLC45A1		20	30153066-30
0.24054343	13	31787023	675	BRCA2		20	30153066-30
0.24130974	5	76284505	1393	CRHBP	X		24462036-24
0.26265843	11	808892	57104	PNPLA2	X		24462036-24
0.29004353	1	36721568	1441	CSF3R		19	41090225-41
0.2406276	2	241456532	189	AGXT		1	44216369-44
0.28994387	3	45959946	10663	CXCR6		1	24671778-24
0.25651615	19	56567263	4818	NKG7		19	59412606-59
0.25802421	7	75420817	5447	POR		19	59412606-59
0.25779019	2	106047843	84417	ECRG4		19	59412606-59

0.27726251	1	170894886	356	FASLG		1	24671778-24
0.25378405	2	241456532	189	AGXT		3	12914665-12
0.24761223	20	34707347	84174	SLA2		7	149208621-1
0.27341918	19	56582398	3982	LIM2		3	50375436-50
0.34286829	4	47831037	7294	TXK		10	71063149-71
0.29109579	19	50517570	1158	CKM		6	52236896-52
0.25957085	16	56258962	222487	GPR97		1	44216369-44
0.2420171	1	36721568	1441	CSF3R		1	44216369-44
0.24686014	1	191341814	51022	GLRX2		1	44216369-44
0.2621039	11	117718482	915	CD3D		7	149208621-1
0.26089025	12	53043844	53831	GPR84	X		24462036-24
0.24591373	7	75421357	5447	POR		10	99070864-99
0.26072933	1	191341814	51022	GLRX2		3	12914665-12
0.24827213	12	121781072	27198	GPR81		7	149208621-1
0.24975138	9	35640561	27240	SIT1		7	149208621-1
0.33470318	17	77868065	924	CD7		1	24671778-24
0.28326504	16	30390788	3683	ITGAL		14	19995423-19
0.28188853	20	24878099	8530	CST7		19	41090225-41
0.25258938	1	32489589	3932	LCK		7	149208621-1
0.26801134	10	73519632	9806	SPOCK2		1	24671778-24
0.24493396	17	1611970	5176	SERPINF1		1	44216369-44
0.29040039	5	76284505	1393	CRHBP		17	70826142-70
0.26334434	14	99601789	51466	EVL		1	24671778-24
0.24212221	7	99808438	29992	PILRA		1	44216369-44
0.26920034	1	159032429	4063	LY9		6	33772190-33
0.24929347	7	75420817	5447	POR	X		24462036-24
0.26440005	3	133518757	55	ACPP		9	36153798-36
0.27095791	10	72032815	5551	PRF1		1	24671778-24
0.25907649	10	72032298	5551	PRF1		15	38653856-38
0.23856362	19	880046	1820	ARID3A	X		24462036-24
0.25273137	2	74913110	3099	HK2		9	36153798-36
0.24942069	13	50537954	2974	GUCY1B2		9	36153798-36
0.25797335	2	106047843	84417	ECRG4		1	44216369-44
0.28110552	19	56567263	4818	NKG7		19	41090225-41
0.25203938	7	75420817	5447	POR		1	44216369-44
0.23836191	19	56582398	3982	LIM2		1	182864519-1
0.24698058	3	123526489	1475	CSTA		3	12914665-12
0.27803304	2	242450682	5133	PDCD1		6	52236896-52
0.28388398	11	117680841	916	CD3E		1	24671778-24
0.26049384	11	117680841	916	CD3E		8	124584719-1
0.26380678	1	170894643	356	FASLG		1	24671778-24
0.24173333	1	36721568	1441	CSF3R		9	36153798-36
0.28725442	11	117718540	915	CD3D		1	24671778-24
0.28894579	11	117720322	917	CD3G		1	24671778-24

0.26160221	11	117718540	915	CD3D	8	124584719-1
0.31078019	9	35640561	27240	SIT1	1	24671778-24
0.31449717	11	117718482	915	CD3D	1	24671778-24
0.25953654	2	242450682	5133	PDCD1	1	24671778-24
0.27609528	11	117718482	915	CD3D	8	124584719-1
0.28590398	1	159032429	4063	LY9	6	52236896-52
0.25984233	1	32489589	3932	LCK	1	24671778-24
0.2891876	12	121781072	27198	GPR81	10	71063149-71
0.25864076	1	159032429	4063	LY9	1	24671778-24
0.25084914	21	33696497	3460	IFNGR2	15	38653856-38
0.32594409	1	181826150	4688	NCF2	1	15771847-15
0.25544082	11	63729582	83706	URP2	1	24671778-24
0.29442901	21	33696497	3460	IFNGR2	10	71063149-71
0.25446032	17	37599199	84514	LGP1	1	24671778-24
0.26760167	11	71524436	2352	FOLR3	1	24671778-24
0.29612723	2	46600883	90423	ATP6V1E2	10	71063149-71
0.28059996	7	75421357	5447	POR	6	52236896-52
0.25297965	12	53043844	53831	GPR84	15	38653856-38
0.27237973	6	31690816	199	AIF1	1	24671778-24
0.29069958	1	8195939	50651	SLC45A1	10	71063149-71
0.27557952	21	33696497	3460	IFNGR2	1	24671778-24
0.27737679	2	46600883	90423	ATP6V1E2	1	24671778-24
0.30914958	13	31787023	675	BRCA2	10	71063149-71
0.29675335	4	5945592	1400	CRMP1	8	72916714-72
0.28722458	9	139047467	2529	FUT7	10	71063149-71
0.29162439	11	71524436	2352	FOLR3	6	52236896-52
0.29981572	9	35608412	971	CD72	10	71063149-71
0.28949891	11	63729582	83706	URP2	6	52236896-52
0.30267677	19	56567263	4818	NKG7	10	71063149-71
0.25371129	1	151630113	6279	S100A8	1	24671778-24
0.38519354	19	880046	1820	ARID3A	4	57021952-57
0.25257257	9	139047467	2529	FUT7	1	24671778-24
0.24920962	14	24115961	1511	CTSG	15	38653856-38
0.28863961	1	8195939	50651	SLC45A1	1	24671778-24
0.26156003	19	880046	1820	ARID3A	1	24671778-24
0.25655069	17	1611970	5176	SERPINF1	15	38653856-38
0.29025444	21	33696497	3460	IFNGR2	6	52236896-52
0.32320719	12	50685980	160622	GRASP	4	143222645-1
0.26711581	17	72827828	10801	SEPT9	1	24671778-24
0.25486046	3	123526489	1475	CSTA	1	24671778-24
0.2642097	5	76284505	1393	CRHBP	15	38653856-38
0.30580587	13	31787023	675	BRCA2	1	24671778-24
0.25372847	19	4490943	116844	LRG1	15	38653856-38
0.25216308	2	241456532	189	AGXT	17	71547046-71

0.2545591	11	59708133	64231	MS4A6A	7	98892836-98
0.2988549	16	63713342	1009	CDH11	8	72916714-72
0.26355341	9	35608412	971	CD72	8	124584719-1
0.29959365	6	146906926	10981	RAB32	8	72916714-72
0.2862096	1	151630204	6279	S100A8	10	71063149-71
0.26755064	10	44815977	7570	ZNF22	1	24671778-24
0.26916968	9	35608412	971	CD72	2	136381356-1
0.29372049	19	56567263	4818	NKG7	1	24671778-24
0.24343119	3	133518757	55	ACPP	7	98892836-98
0.39502414	12	50685980	160622	GRASP	4	57021952-57
0.25146435	16	56258956	222487	GPR97	1	24671778-24
0.26201227	11	59708133	64231	MS4A6A	15	38653856-38
0.28421624	19	56567263	4818	NKG7	2	136381356-1
0.25535626	2	241456532	189	AGXT	15	38653856-38
0.25459955	12	13139815	83445	GSG1	1	24671778-24
0.30994398	2	46600883	90423	ATP6V1E2	21	45470779-45
0.29918425	16	56258962	222487	GPR97	10	71063149-71
0.43177812	19	56567263	4818	NKG7	4	57021952-57
0.30797886	12	53043844	53831	GPR84	1	24671778-24
0.30236435	1	8195939	50651	SLC45A1	6	52236896-52
0.31224187	9	35608412	971	CD72	1	24671778-24
0.25579349	7	75421357	5447	POR	7	149208621-1
0.30478692	5	76284505	1393	CRHBP	10	71063149-71
0.31831241	19	41091025	7305	TYROBP	10	71063149-71
0.25294976	3	133518757	55	ACPP	15	38653856-38
0.29168737	15	38388759	5330	PLCB2	6	52236896-52
0.29397482	1	205037818	29949	IL19	10	71063149-71
0.38179917	19	40321946	5348	FXYD1	4	57021952-57
0.29161664	6	41238895	54209	TREM2	10	71063149-71
0.28376415	9	35608412	971	CD72	6	52236896-52
0.26136525	19	52530972	27202	GPR77	14	19995423-19
0.29287465	6	31662827	7940	LST1	1	24671778-24
0.27924348	10	81699171	6441	SFTPD	1	24671778-24
0.25921339	16	70598877	1723	DHODH	1	24671778-24
0.2453427	19	56567263	4818	NKG7	10	121427028-1
0.27262587	1	151630204	6279	S100A8	1	24671778-24
0.2608369	12	6179035	928	CD9	2	136381356-1
0.38056335	1	36721568	1441	CSF3R	4	57021952-57
0.40144268	7	99808438	29992	PILRA	4	57021952-57
0.25724601	10	53744215	22943	DKK1	1	182864519-1
0.29480817	19	59258650	284415	UNQ3033	6	52236896-52
0.23780932	19	5518758	257000	PLAC2	1	182864519-1
0.28517234	21	44598210	7226	TRPM2	1	24671778-24
0.26611277	19	40321946	5348	FXYD1	1	24671778-24

0.28237729	20	24878099	8530	CST7	1	24671778-24
0.24366995	3	152529997	53829	P2RY13	7	98892836-98
0.28614836	14	36736981	145282	MIPOL1	1	182864519-1
0.30879276	19	15252927	23476	BRD4	1	24671778-24
0.27682172	16	56258962	222487	GPR97	1	24671778-24
0.3972099	19	46951235	4680	CEACAM6	4	57021952-57
0.29450255	17	72827828	10801	SEPT9	6	52236896-52
0.2677187	19	4490943	116844	LRG1	1	24671778-24
0.29859414	19	880046	1820	ARID3A	6	52236896-52
0.28619287	3	139810060	55179	FAIM	10	71063149-71
0.27802473	14	24115961	1511	CTSG	1	24671778-24
0.40382547	16	56258962	222487	GPR97	4	57021952-57
0.26942604	11	59708133	64231	MS4A6A	10	71569035-71
0.297196	12	6179035	928	CD9	1	24671778-24
0.26270892	16	56258962	222487	GPR97	2	136381356-1
0.2691823	1	54786297	26027	ACOT11	2	136381356-1
0.29461693	5	178255530	80108	ZFP2	8	72916714-72
0.26897188	12	13139815	83445	GSG1	2	136381356-1
0.26671807	19	52530972	27202	GPR77	2	136381356-1
0.25386762	1	176779118	400798	FLJ35530	1	24671778-24
0.3025928	2	106869979	84620	ST6GAL2	8	72916714-72
0.26976148	5	76284505	1393	CRHBP	2	136381356-1
0.26001914	12	52977085	4778	NFE2	1	24671778-24
0.30633327	19	41091025	7305	TYROBP	1	24671778-24
0.32935594	19	56567263	4818	NKG7	5	136981237-1
0.26547948	11	59708133	64231	MS4A6A	14	19995423-19
0.36071339	13	31787023	675	BRCA2	9	94886967-94
0.25123261	6	167456511	1235	CCR6	1	24671778-24
0.27313618	1	36721568	1441	CSF3R	14	19995423-19
0.28517614	11	808892	57104	PNPLA2	6	52236896-52
0.26176698	11	63729582	83706	URP2	7	149208621-1
0.26670377	11	71524436	2352	FOLR3	7	149208621-1
0.28129059	10	81699171	6441	SFTPD	6	52236896-52
0.26552084	2	127129833	2995	GYPC	1	24671778-24
0.30110524	11	93774294	10888	GPR83	8	72916714-72
0.29149755	16	21078585	55567	DNAH3	8	124584719-1
0.25704071	20	61962518	140701	C20orf135	1	24671778-24
0.30116616	7	45926892	3486	IGFBP3	8	72916714-72
0.28881547	1	205037818	29949	IL19	1	24671778-24
0.30921622	13	31787023	675	BRCA2	9	129743305-1
0.3348541	14	36736981	145282	MIPOL1	8	72916714-72
0.29519241	1	181826150	4688	NCF2	1	24671778-24
0.28646758	11	117720322	917	CD3G	1	245678605-2
0.28562324	21	44598210	7226	TRPM2	6	52236896-52

0.25022678	1	245646729	114548	CIAS1	1	24671778-24
0.27415195	8	145158870	375686	SPATC1	1	24671778-24
0.25663734	2	46600883	90423	ATP6V1E2	7	149208621-1
0.26403977	11	63729582	83706	URP2	6	33772190-33
0.25908206	4	109034053	166929	MGC26963	15	38653856-38
0.25284008	9	138676375	51162	EGFL7	7	98892836-98
0.26833088	6	31690816	199	AIF1	7	149208621-1
0.28836037	12	67643600	1368	CPM	15	38653856-38
0.2815062	14	24115961	1511	CTSG	6	52236896-52
0.30349863	12	67643600	1368	CPM	7	98892836-98
0.31770892	13	50538949	2974	GUCY1B2	10	71063149-71
0.29779573	1	7922901	3604	TNFRSF9	10	71063149-71
0.40998583	12	52977085	4778	NFE2	4	57021952-57
0.28090619	3	133518757	55	ACPP	10	71569035-71
0.26666574	17	53714577	4353	MPO	1	24671778-24
0.29066625	12	13139815	83445	GSG1	6	52236896-52
0.38521137	11	2499438	3784	KCNQ1	4	57021952-57
0.32763128	17	1611970	5176	SERPINF1	1	24671778-24
0.27842564	19	4490943	116844	LRG1	6	52236896-52
0.26935471	2	127129833	2995	GYPC	8	124584719-1
0.3056012	1	36721568	1441	CSF3R	1	24671778-24
0.31686061	17	53714577	4353	MPO	3	58132673-58
0.32845856	19	52530972	27202	GPR77	1	24671778-24
0.29943034	16	56258956	222487	GPR97	6	52236896-52
0.27915551	21	33696497	3460	IFNGR2	7	149208621-1
0.26534389	11	67534528	221	ALDH3B1	1	24671778-24
0.30186157	2	176680359	3237	HOXD11	8	72916714-72
0.29216053	13	98028005	8428	STK24	1	24671778-24
0.26897845	21	33696497	3460	IFNGR2	6	33772190-33
0.25831349	20	58063710	284756	FLJ33860	1	24671778-24
0.35355978	13	31787023	675	BRCA2	4	48582339-48
0.27371036	11	118259745	643	BLR1	6	26305014-26
0.28057997	7	75421357	5447	POR	1	117113051-1
0.26920174	1	7922901	3604	TNFRSF9	1	24671778-24
0.24064874	7	75420817	5447	POR	7	149208621-1
0.29030884	1	28375495	5724	PTAFR	6	52236896-52
0.24851942	1	156415852	912	CD1D	7	98892836-98
0.26794324	9	139047066	2529	FUT7	1	24671778-24
0.25672472	20	24878099	8530	CST7	3	59710182-59
0.27027399	2	46600883	90423	ATP6V1E2	6	33772190-33
0.32476929	6	41238895	54209	TREM2	1	24671778-24
0.27664556	10	97505362	953	ENTPD1	1	24671778-24
0.38040019	19	60484508	23640	HSPBP1	4	57021952-57
0.28098254	12	67643600	1368	CPM	10	71569035-71

0.31920143	19	56567263	4818	NKG7		9 129743305-1
0.27566602	7	36395645	54443	ANLN		15 38653856-38
0.24042111	19	59258650	284415	UNQ3033		7 149208621-1
0.29010391	1	245648040	114548	CIAS1		1 24671778-24
0.31133373	17	1611970	5176	SERPINF1		6 52236896-52
0.38451539	7	141293159	23601	CLEC5A		4 57021952-57
0.26051031	4	109034053	166929	MGC26963		10 71569035-71
0.2572361	14	20429783	6037	RNASE3		17 71547046-71
0.2991931	2	74913110	3099	HK2		1 24671778-24
0.25033023	16	30390788	3683	ITGAL	X	24462036-24
0.27644408	1	92725229	2672	GFI1		10 15293996-15
0.30711543	13	50537954	2974	GUCY1B2		1 24671778-24
0.39592356	1	92725229	2672	GFI1		4 57021952-57
0.30686667	19	40321946	5348	FXYD1		6 52236896-52
0.25487373	4	109034053	166929	MGC26963		1 24671778-24
0.28524357	2	127129833	2995	GYPC		6 52236896-52
0.26594779	1	32489589	3932	LCK		12 105156317-1
0.26214026	3	152529997	53829	P2RY13		1 24671778-24
0.2655311	13	31787023	675	BRCA2		6 33772190-33
0.28929049	14	103164372	3831	KNS2		1 24671778-24
0.28257498	13	31787023	675	BRCA2		7 149208621-1
0.25147124	11	61861296	80150	ASRGL1		1 24671778-24
0.28574915	2	241456532	189	AGXT		6 52236896-52
0.26304482	5	76284505	1393	CRHBP		16 68843829-68
0.28056229	1	8195939	50651	SLC45A1		7 149208621-1
0.34399692	16	56258962	222487	GPR97		9 94886967-94
0.29581678	11	2877365	5002	SLC22A18		1 24671778-24
0.28208127	20	61962518	140701	C20orf135		6 52236896-52
0.31704741	6	41362863	54210	TREM1		1 24671778-24
0.3191062	13	50538949	2974	GUCY1B2		1 24671778-24
0.29710002	1	27822930	2268	FGR		10 71063149-71
0.35201902	19	880046	1820	ARID3A		4 48582339-48
0.27099123	1	8195939	50651	SLC45A1		6 33772190-33
0.32644769	16	56258962	222487	GPR97		6 52236896-52
0.38470804	20	33666135	6676	SPAG4		4 57021952-57
0.34423346	11	59708133	64231	MS4A6A		1 24671778-24
0.27909231	8	38950850	203100	HTRA4		1 117113051-1
0.25857695	12	6355798	6337	SCNN1A		1 24671778-24
0.25549392	19	495349	3004	GZMM		20 1480341-148
0.3216916	3	133518757	55	ACPP		1 24671778-24
0.38166265	19	56567263	4818	NKG7		1 6168145-616
0.27342306	20	57016289	1522	CTSZ		1 24671778-24
0.29027431	2	74913110	3099	HK2		6 52236896-52
0.34002819	2	241456532	189	AGXT		1 24671778-24

0.28385661	19	60484508	23640	HSPBP1	1	24671778-24
0.26102048	9	139047467	2529	FUT7	7	149208621-1
0.2748347	19	56567263	4818	NKG7	7	149208621-1
0.27053629	19	43995615	3960	LGALS4	10	71569035-71
0.32186759	1	36721568	1441	CSF3R	6	52236896-52
0.25729508	11	59708133	64231	MS4A6A	16	68843829-68
0.26423676	16	31274037	3687	ITGAX	7	98892836-98
0.25666787	6	32299818	4855	NOTCH4	1	24671778-24
0.23490136	14	57932850	387990	UNQ9438	19	56368225-56
0.2753625	16	2951662	124222	PAQR4	1	24671778-24
0.28473866	10	97505362	953	ENTPD1	6	52236896-52
0.27562758	19	880046	1820	ARID3A	7	149208621-1
0.29602938	11	71524436	2352	FOLR3	1	117113051-1
0.36868307	20	24878099	8530	CST7	9	94886967-94
0.27442602	20	43316404	6590	SLPI	1	24671778-24
0.30831074	11	2877375	5002	SLC22A18	1	24671778-24
0.27177632	19	60484508	23640	HSPBP1	2	136381356-1
0.35820564	1	181826150	4688	NCF2	9	94886967-94
0.28344878	19	5802504	2525	FUT3	1	24671778-24
0.28595206	10	124729456	118672	C10orf89	10	71063149-71
0.36730775	19	52530972	27202	GPR77	9	94886967-94
0.32426581	11	71524436	2352	FOLR3	17	18864772-18
0.31327214	20	24878099	8530	CST7	9	129743305-1
0.26204882	9	130911839	5524	PPP2R4	1	24671778-24
0.27419339	7	75420817	5447	POR	6	33772190-33
0.38455216	11	56951601	29015	SLC43A3	4	57021952-57
0.24646276	19	41091025	7305	TYROBP	7	149208621-1
0.25130986	7	56085732	908	CCT6A	1	24671778-24
0.27976987	20	43316404	6590	SLPI	14	19995423-19
0.26228041	6	31662827	7940	LST1	7	149208621-1
0.29031671	6	31690816	199	AIF1	1	117113051-1
0.30724108	15	72005833	4016	LOXL1	1	24671778-24
0.32532148	12	52977085	4778	NFE2	6	52236896-52
0.25283691	11	65081734	4054	LTBP3	14	90768860-90
0.2912963	1	115682737	4803	NGFB	8	72916714-72
0.29051386	9	35608412	971	CD72	7	149208621-1
0.241007	7	99808438	29992	PILRA	7	149208621-1
0.2996242	6	41238895	54209	TREM2	9	129743305-1
0.32520392	12	67643600	1368	CPM	1	24671778-24
0.26262194	1	156415852	912	CD1D	10	71569035-71
0.23776456	12	121781072	27198	GPR81	19	48842368-48
0.26458176	11	59708133	64231	MS4A6A	14	93587296-93
0.2527342	1	156415852	912	CD1D	1	24671778-24
0.27786773	9	35608412	971	CD72	6	33772190-33

0.2817317	1	152067323	57459	GATAD2B	6	52236896-52
0.26871124	20	43316404	6590	SLPI	2	136381356-1
0.35609053	11	808892	57104	PNPLA2	4	48582339-48
0.24531096	10	72032298	5551	PRF1	X	24462036-24
0.2748042	3	123526489	1475	CSTA	7	149208621-1
0.2910771	20	33666135	6676	SPAG4	1	24671778-24
0.43812881	19	56567263	4818	NKG7	9	94886967-94
0.27872039	17	3766139	5023	P2RX1	6	52236896-52
0.410764	1	27822930	2268	FGR	4	57021952-57
0.31173874	11	63729582	83706	URP2	1	117113051-1
0.23690002	6	31690816	199	AIF1	20	3734490-373
0.32371752	6	31690816	199	AIF1	17	18864772-18
0.31497388	1	181826150	4688	NCF2	9	129743305-1
0.25340639	16	56258956	222487	GPR97	7	149208621-1
0.24632712	5	76284393	1393	CRHBP	10	99208306-99
0.24030393	19	46951235	4680	CEACAM6	7	149208621-1
0.28004681	10	44815977	7570	ZNF22	7	149208621-1
0.34448397	6	41362863	54210	TREM1	9	94886967-94
0.30242224	21	33696497	3460	IFNGR2	1	117113051-1
0.29569329	17	35147939	2886	GRB7	6	52236896-52
0.29644996	7	36395645	54443	ANLN	1	24671778-24
0.2712148	11	808892	57104	PNPLA2	7	149208621-1
0.27225869	11	808917	57104	PNPLA2	15	38653856-38
0.26011448	21	44598210	7226	TRPM2	7	149208621-1
0.26937359	3	123526489	1475	CSTA	6	33772190-33
0.25430033	12	13139815	83445	GSG1	7	149208621-1
0.25890279	10	134108411	170393	C10orf91	15	38653856-38
0.25239452	5	76284505	1393	CRHBP	7	149208621-1
0.25771236	7	75421357	5447	POR	11	60544218-60
0.34234404	17	53714577	4353	MPO	9	94886967-94
0.2922379	19	43995615	3960	LGALS4	1	24671778-24
0.25818162	11	59580569	932	MS4A3	7	149208621-1
0.28176958	5	179153151	4056	LTC4S	1	24671778-24
0.26739657	17	53714577	4353	MPO	3	59710182-59
0.30482154	1	151630113	6279	S100A8	7	149208621-1
0.27400481	14	20429255	6037	RNASE3	1	24671778-24
0.28845228	1	151630113	6279	S100A8	6	33772190-33
0.24112983	2	46600883	90423	ATP6V1E2	20	3734490-373
0.25263273	14	24115961	1511	CTSG	7	149208621-1
0.26883076	12	6179035	928	CD9	7	149208621-1
0.26925443	19	15252927	23476	BRD4	6	33772190-33
0.30919257	9	139047066	2529	FUT7	6	52236896-52
0.28973311	6	43720958	221421	C6orf206	8	124584719-1
0.39550261	19	2232919	374872	C19orf35	4	57021952-57

0.28163259	19	5802504	2525	FUT3	6	52236896-52
0.29303027	7	141293159	23601	CLEC5A	6	52236896-52
0.26297765	1	54786297	26027	ACOT11	7	149208621-1
0.29331794	14	22658002	1053	CEBPE	6	52236896-52
0.25364538	16	70598877	1723	DHODH	7	149208621-1
0.26529617	20	24878099	8530	CST7	7	149208621-1
0.27133308	3	133518757	55	ACPP	16	68843829-68
0.25109084	3	133518757	55	ACPP	3	49028156-49
0.29639899	12	53043844	53831	GPR84	6	33772190-33
0.25065603	1	8195939	50651	SLC45A1	10	73488877-73
0.27445774	16	56134975	221188	GPR114	1	24671778-24
0.28206914	14	23610255	9362	CPNE6	3	59710182-59
0.29110227	6	11887927	84830	C6orf105	10	71063149-71
0.36319233	13	98028005	8428	STK24	9	94886967-94
0.25271117	19	40321946	5348	FXYD1	7	149208621-1
0.31714814	12	53043844	53831	GPR84	7	149208621-1
0.3546045	16	56258962	222487	GPR97	4	48582339-48
0.28446428	19	46992652	1084	CEACAM3	1	24671778-24
0.36055133	7	99808438	29992	PILRA	4	48582339-48
0.40864251	19	46992652	1084	CEACAM3	4	57021952-57
0.25720788	11	59580569	932	MS4A3	6	33772190-33
0.30019408	6	41362863	54210	TREM1	9	129743305-1
0.2902907	11	33714989	966	CD59	1	117113051-1
0.25870118	12	67643600	1368	CPM	16	68843829-68
0.2998456	19	60484508	23640	HSPBP1	6	52236896-52
0.27994779	14	22375620	4323	MMP14	1	117113051-1
0.26506187	16	56258962	222487	GPR97	7	149208621-1
0.28463179	11	59708133	64231	MS4A6A	5	133946601-1
0.33910301	2	46600883	90423	ATP6V1E2	17	18864772-18
0.25822529	12	13139815	83445	GSG1	6	33772190-33
0.29889897	3	133518757	55	ACPP	9	129743305-1
0.27984057	10	97505212	953	ENTPD1	1	24671778-24
0.28874706	10	44815977	7570	ZNF22	6	33772190-33
0.28664874	7	36395645	54443	ANLN	6	52236896-52
0.28819647	10	81699171	6441	SFTPD	7	149208621-1
0.38280187	11	808917	57104	PNPLA2	4	57021952-57
0.33182655	14	103164372	3831	KNS2	6	52236896-52
0.27535738	19	52530972	27202	GPR77	7	149208621-1
0.35104259	1	7922901	3604	TNFRSF9	9	94886967-94
0.28183468	1	151630204	6279	S100A8	7	149208621-1
0.30223952	11	56951601	29015	SLC43A3	1	24671778-24
0.4185111	19	56567263	4818	NKG7	1	23757369-23
0.34360854	1	92725229	2672	GFI1	9	94886967-94
0.26368007	5	76284505	1393	CRHBP	6	33772190-33

0.30451959	1	8195939	50651	SLC45A1	1	117113051-1
0.32910884	11	67534258	221	ALDH3B1	4	143222645-1
0.26995183	1	92725229	2672	GFI1	3	59710182-59
0.32027028	9	138676375	51162	EGFL7	1	24671778-24
0.36984531	5	76284505	1393	CRHBP	4	48582339-48
0.24440847	8	71479423	10499	NCOA2	7	149208621-1
0.29689257	20	33666135	6676	SPAG4	6	52236896-52
0.24981744	7	80386631	10512	SEMA3C	17	72183709-72
0.25099839	12	52977085	4778	NFE2	7	149208621-1
0.28472526	14	20429783	6037	RNASE3	1	24671778-24
0.33090853	1	8195939	50651	SLC45A1	17	18864772-18
0.27007624	19	4490943	116844	LRG1	7	149208621-1
0.26532711	6	41238895	54209	TREM2	7	149208621-1
0.30472824	5	80565096	1160	CKMT2	1	117113051-1
0.31730781	19	15252927	23476	BRD4	7	149208621-1
0.25597848	12	54646332	1017	CDK2	1	24671778-24
0.2838396	1	159436198	4720	NDUFS2	1	24671778-24
0.31660087	7	75420817	5447	POR	17	18864772-18
0.28600487	10	81699171	6441	SFTPD	6	33772190-33
0.37748851	3	133518757	55	ACPP	5	43075279-43
0.25966878	11	63729582	83706	URP2	11	60544218-60
0.27377841	17	1611970	5176	SERPINF1	6	33772190-33
0.2531204	2	127129833	2995	GYPC	7	149208621-1
0.32436505	1	154177985	339403	RLN3R2	1	24671778-24
0.34378128	1	92725229	2672	GFI1	5	136981237-1
0.27340685	1	205037818	29949	IL19	7	149208621-1
0.26016135	1	205037818	29949	IL19	6	33772190-33
0.27692088	16	31274037	3687	ITGAX	1	24671778-24
0.30797251	13	31787023	675	BRCA2	1	6444014-644
0.24868226	1	151630113	6279	S100A8	10	73488877-73
0.28363242	2	107969437	60482	SLC5A7	14	90768860-90
0.29139565	19	59258650	284415	UNQ3033	1	117113051-1
0.35625776	19	56567263	4818	NKG7	12	45916614-45
0.31643862	3	69144793	285203	AER61	4	143222645-1
0.29904663	11	72606702	5029	P2RY2	1	117113051-1
0.28360334	1	242279981	10472	ZNF238	14	90768860-90
0.25188715	19	40512021	933	CD22	7	149208621-1
0.38938394	20	24878099	8530	CST7	1	23757369-23
0.39445373	19	56567263	4818	NKG7	6	31913122-31
0.27657738	14	24115961	1511	CTSG	6	33772190-33
0.25971317	2	101974781	7850	IL1R2	7	149208621-1
0.27154243	11	71524436	2352	FOLR3	11	60544218-60
0.27784967	16	56258962	222487	GPR97	6	33772190-33
0.26193114	1	36721568	1441	CSF3R	6	33772190-33

0.25793909	1	8195939	50651	SLC45A1	20	3734490-373
0.38300347	21	42792092	54020	SLC37A1	4	57021952-57
0.24346838	2	106047843	84417	ECRG4	20	3734490-373
0.25738346	12	53043844	53831	GPR84	10	73488877-73
0.39463852	11	67534258	221	ALDH3B1	4	57021952-57
0.2653909	7	100076686	7036	TFR2	1	24671778-24
0.3342421	13	50537954	2974	GUCY1B2	9	129743305-1
0.31109451	16	56258962	222487	GPR97	12	44868183-44
0.27448385	19	4490943	116844	LRG1	6	33772190-33
0.26242168	1	3558072	7161	TP73	14	90768860-90
0.26534468	12	6179035	928	CD9	11	60651626-60
0.29952805	12	53043844	53831	GPR84	1	117113051-1
0.25002598	17	72151326	55808	ST6GALNAC1	10	99070864-99
0.24973749	17	53714577	4353	MPO	7	149208621-1
0.29550772	5	154210501	10826	C5orf4	1	117113051-1
0.28075621	19	15252927	23476	BRD4	1	117113051-1
0.29999203	17	72827828	10801	SEPT9	1	117113051-1
0.28721023	1	36721568	1441	CSF3R	7	149208621-1
0.238179	11	73856409	10008	KCNE3	20	3734490-373
0.30395041	19	880046	1820	ARID3A	1	117113051-1
0.31114874	17	1611970	5176	SERPINF1	7	149208621-1
0.26051488	2	46600883	90423	ATP6V1E2	11	60544218-60
0.37802531	12	67643600	1368	CPM	5	43075279-43
0.35066015	3	133518757	55	ACPP	4	48582339-48
0.24452072	14	103164372	3831	KNS2	7	149208621-1
0.36308115	8	71479423	10499	NCOA2	4	48582339-48
0.3188849	2	106047843	84417	ECRG4	1	117113051-1
0.27374207	9	35608412	971	CD72	10	73488877-73
0.26320972	1	176779118	400798	FLJ35530	6	33772190-33
0.2951219	6	11887927	84830	C6orf105	1	24671778-24
0.31144983	9	130911839	5524	PPP2R4	6	52236896-52
0.28910791	11	47356570	6688	SPI1	6	52236896-52
0.24518381	9	139047066	2529	FUT7	7	149208621-1
0.2860224	11	33870664	4005	LMO2	6	52236896-52
0.30631426	19	56567263	4818	NKG7	1	6444014-644
0.36828095	20	24878099	8530	CST7	6	31913122-31
0.24220142	11	2877375	5002	SLC22A18	7	149208621-1
0.31534685	9	35608412	971	CD72	1	117113051-1
0.27456006	13	50537954	2974	GUCY1B2	7	149208621-1
0.24874377	1	151630113	6279	S100A8	20	3734490-373
0.36989754	13	31787023	675	BRCA2	22	30007802-30
0.368153	13	31787023	675	BRCA2	17	18864772-18
0.25386396	9	35608412	971	CD72	20	3734490-373
0.33814115	19	880046	1820	ARID3A	17	18864772-18

0.28167167	10	134108411	170393	C10orf91		1	24671778-24
0.27843518	6	31690816	199	AIF1		11	60544218-60
0.25381056	11	2499438	3784	KCNQ1		7	149208621-1
0.27042041	2	101974781	7850	IL1R2		6	33772190-33
0.29193768	9	103540851	116443	GRIN3A		9	128308937-1
0.27186003	2	127129833	2995	GYPC		6	33772190-33
0.25472498	19	46992652	1084	CEACAM3		3	59710182-59
0.24735069	20	58063710	284756	FLJ33860		7	149208621-1
0.27302582	8	11763503	1508	CTSB		1	24671778-24
0.32457365	3	123526489	1475	CSTA		17	18864772-18
0.26089953	11	67534528	221	ALDH3B1		7	149208621-1
0.25642323	12	53043844	53831	GPR84		20	3734490-373
0.32059234	5	76284393	1393	CRHBP		1	24671778-24
0.24869718	16	30390788	3683	ITGAL		4	140265851-1
0.2432756	12	51886921	3695	ITGB7	X		24462036-24
0.34085944	1	151630113	6279	S100A8		17	18864772-18
0.27097612	14	24115961	1511	CTSG		11	60651626-60
0.34415981	12	53043844	53831	GPR84		17	18864772-18
0.3426084	20	24878099	8530	CST7		12	45916614-45
0.27417462	1	245648040	114548	CIAS1		7	149208621-1
0.30700762	10	129594939	5791	PTPRE		1	24671778-24
0.3222693	11	73856409	10008	KCNE3		1	117113051-1
0.35152804	20	43317160	6590	SLPI		9	94886967-94
0.24227285	16	64958100	1003	CDH5		7	149208621-1
0.24594139	7	145444372	26047	CNTNAP2		14	90768860-90
0.27480933	16	30390788	3683	ITGAL		12	105156317-1
0.28968039	21	33696497	3460	IFNGR2		11	60544218-60
0.25602879	11	117720322	917	CD3G		6	41351742-41
0.32876933	6	31662827	7940	LST1		17	18864772-18
0.34389875	17	1611970	5176	SERPINF1		12	45916614-45
0.28230056	19	649371	400668	PRSSL1		1	24671778-24
0.26903337	10	97505362	953	ENTPD1		7	149208621-1
0.26008247	3	159771755	4291	MLF1		14	90768860-90
0.26518231	1	245648040	114548	CIAS1		6	33772190-33
0.34111542	16	56258962	222487	GPR97		12	45916614-45
0.30936606	11	59708133	64231	MS4A6A		7	149208621-1
0.40394	11	1817920	7136	TNNI2		4	57021952-57
0.3147523	21	44598210	7226	TRPM2		17	18864772-18
0.2959992	9	138676375	51162	EGFL7		9	129743305-1
0.31286322	10	44815977	7570	ZNF22		1	117113051-1
0.29293934	11	59708133	64231	MS4A6A		6	33772190-33
0.26407828	19	59295936	126014	OSCAR		7	149208621-1
0.32805303	1	151630204	6279	S100A8		6	33772190-33
0.28449001	17	1611970	5176	SERPINF1		1	117113051-1

0.29419148	1	154177985	339403	RLN3R2	9	129743305-1
0.27449045	20	61962518	140701	C20orf135	6	33772190-33
0.31980127	12	6179035	928	CD9	17	18864772-18
0.24743486	19	15252927	23476	BRD4	20	3734490-373
0.24867756	7	75420817	5447	POR	11	60544218-60
0.34790675	9	139047467	2529	FUT7	17	18864772-18
0.28978606	1	151630204	6279	S100A8	1	117113051-1
0.29720075	12	6179035	928	CD9	1	117113051-1
0.29877655	19	41091025	7305	TYROBP	11	60651626-60
0.25323754	15	72005833	4016	LOXL1	7	149208621-1
0.26281558	2	5750620	6664	SOX11	14	90768860-90
0.25977889	14	103164372	3831	KNS2	6	33772190-33
0.2476806	3	52235130	54106	TLR9	14	90768860-90
0.32542389	12	127903721	144423	GLT1D1	19	52405148-52
0.25427065	15	38388759	5330	PLCB2	11	60544218-60
0.25892525	21	44598210	7226	TRPM2	10	73488877-73
0.33588254	19	15252927	23476	BRD4	17	18864772-18
0.30812854	11	808892	57104	PNPLA2	1	117113051-1
0.27615921	2	74913110	3099	HK2	6	33772190-33
0.29859124	6	37081496	221472	FGD2	1	117113051-1
0.32463771	10	81699171	6441	SFTPD	17	18864772-18
0.28590985	16	56258962	222487	GPR97	11	60651626-60
0.27452486	1	1207521	6339	SCNN1D	1	24671778-24
0.37570152	19	56567263	4818	NKG7	13	40942702-40
0.35633871	9	139047467	2529	FUT7	22	30007802-30
0.31822181	1	151630204	6279	S100A8	17	18864772-18
0.25124662	16	27319997	50615	IL21R	7	149208621-1
0.26329306	3	45163212	64866	CDCP1	6	33772190-33
0.3520457	11	56951601	29015	SLC43A3	9	94886967-94
0.25903611	9	37025381	5079	PAX5	14	90768860-90
0.34933949	19	46992652	1084	CEACAM3	9	94886967-94
0.27834235	11	118259745	643	BLR1	1	44215475-44
0.31593369	5	76284505	1393	CRHBP	17	18864772-18
0.25191175	11	68536712	219928	MRGPRF	1	24671778-24
0.31377563	14	24115961	1511	CTSG	17	18864772-18
0.35158307	6	89984204	2569	GABRR1	4	48582339-48
0.31469013	1	27822930	2268	FGR	9	129743305-1
0.27941762	15	64577444	10302	SNAPC5	3	59710182-59
0.3324496	11	808917	57104	PNPLA2	1	24671778-24
0.25191798	19	880046	1820	ARID3A	11	60544218-60
0.24207599	20	33666135	6676	SPAG4	7	149208621-1
0.30227067	2	74913110	3099	HK2	7	149208621-1
0.29686669	17	72151326	55808	ST6GALNAC1	12	105156317-1
0.29341534	14	24115961	1511	CTSG	1	117113051-1

0.24802398	10	81699171	6441	SFTPD	20	3734490-373
0.31194623	10	81699171	6441	SFTPD	1	117113051-1
0.31696134	2	241456532	189	AGXT	7	149208621-1
0.23989977	1	151630204	6279	S100A8	20	3734490-373
0.26215939	17	1611970	5176	SERPINF1	10	73488877-73
0.34435964	6	41238895	54209	TREM2	12	45916614-45
0.37498924	19	56567263	4818	NKG7	17	18864772-18
0.2522675	17	72827828	10801	SEPT9	11	60544218-60
0.26333321	19	60851011	29903	HSU79303	16	3059568-305
0.26435297	6	89984204	2569	GABRR1	7	149208621-1
0.26257943	3	152529997	53829	P2RY13	7	149208621-1
0.26135249	16	56258962	222487	GPR97	10	73488877-73
0.28590206	1	8195939	50651	SLC45A1	11	60544218-60
0.2409874	12	13139815	83445	GSG1	20	3734490-373
0.24129277	17	1611970	5176	SERPINF1	20	3734490-373
0.27118543	12	67643600	1368	CPM	6	33772190-33
0.30455437	2	241456532	189	AGXT	6	33772190-33
0.27506152	16	21078585	55567	DNAH3	16	3059568-305
0.28004722	11	59708133	64231	MS4A6A	11	60651626-60
0.30921056	3	133518757	55	ACPP	7	149208621-1
0.28739592	12	67643600	1368	CPM	7	149208621-1
0.31726139	13	50538949	2974	GUCY1B2	7	149208621-1
0.29288003	13	31787023	675	BRCA2	11	60544218-60
0.27321962	2	127129833	2995	GYPC	11	60651626-60
0.25056755	1	170894886	356	FASLG	4	140265851-1
0.26103449	20	57016289	1522	CTSZ	7	149208621-1
0.32256168	19	46951235	4680	CEACAM6	17	18864772-18
0.31969235	19	41091025	7305	TYROBP	1	117113051-1
0.24274385	9	138676375	51162	EGFL7	7	149208621-1
0.28899914	1	36721568	1441	CSF3R	11	60651626-60
0.33451787	20	24878099	8530	CST7	17	18864772-18
0.41159912	16	56258962	222487	GPR97	2	30336048-30
0.26083402	19	5802504	2525	FUT3	7	149208621-1
0.28639539	3	49434859	275	AMT	1	117113051-1
0.24695965	5	39255455	2533	FYB	7	149208621-1
0.31035215	10	104525844	54838	C10orf26	1	117113051-1
0.32868492	16	56258962	222487	GPR97	17	18864772-18
0.27624762	2	106047843	84417	ECRG4	11	60544218-60
0.27999491	11	2510986	3784	KCNQ1	6	52236896-52
0.23820636	12	112058587	8437	RASAL1	19	48852486-48
0.31014938	5	76284505	1393	CRHBP	1	117113051-1
0.25773096	20	43316404	6590	SLPI	7	149208621-1
0.33545445	12	13139815	83445	GSG1	17	18864772-18
0.23615255	19	4490943	116844	LRG1	20	3734490-373

0.24786418	10	81698971	6441	SFTPD	7	149208621-1
0.28756314	10	97505362	953	ENTPD1	6	33772190-33
0.27616543	19	60484508	23640	HSPBP1	7	149208621-1
0.23933699	2	157822650	11227	GALNT5	20	3734490-373
0.25733435	2	15998211	4613	MYCN	9	128308937-1
0.26101303	16	27319997	50615	IL21R	6	33772190-33
0.24755547	1	205735931	1378	CR1	16	3059568-305
0.24943191	5	76284505	1393	CRHBP	20	3734490-373
0.25829954	7	36395645	54443	ANLN	7	149208621-1
0.26317225	19	41091025	7305	TYROBP	20	3734490-373
0.34223194	17	1611970	5176	SERPINF1	22	30007802-30
0.32081796	16	56258956	222487	GPR97	1	117113051-1
0.39326184	1	27822930	2268	FGR	9	94886967-94
0.2572209	6	11887927	84830	C6orf105	16	68843829-68
0.31768638	19	13120872	9592	IER2	10	71569035-71
0.33060462	3	133518757	55	ACPP	12	45916614-45
0.36784998	1	181826150	4688	NCF2	12	45916614-45
0.24873917	16	1605095	57585	CRAMP1L	16	3059568-305
0.26937332	11	73856409	10008	KCNE3	11	60544218-60
0.28288694	2	160363282	9936	CD302	1	117113051-1
0.32002934	11	2877365	5002	SLC22A18	12	44868183-44
0.29586051	2	106047843	84417	ECRG4	11	117715070-1
0.30875221	16	56134975	221188	GPR114	9	129743305-1
0.30967139	1	7922901	3604	TNFRSF9	7	149208621-1
0.28058971	9	102830780	54886	PRG-3	2	73154118-73
0.2575259	11	59708133	64231	MS4A6A	10	73488877-73
0.30576503	5	139705861	1839	HBEGF	1	117113051-1
0.25298671	17	7394101	407977	TNFSF12-TNF X		24462036-24
0.29418486	10	44815977	7570	ZNF22	20	3734490-373
0.26440427	4	109034053	166929	MGC26963	6	33772190-33
0.25071755	11	808892	57104	PNPLA2	11	60544218-60
0.25360095	17	43977036	3212	HOXB2	5	145949378-1
0.34835733	13	98028005	8428	STK24	12	45916614-45
0.32228254	8	11605305	2626	GATA4	1	117113051-1
0.33302777	19	4490943	116844	LRG1	17	18864772-18
0.27249317	19	60484508	23640	HSPBP1	6	33772190-33
0.29133665	19	40939709	126393	HSPB6	1	117113051-1
0.24485522	19	34847706	79156	PLEKHF1	7	149208621-1
0.24419587	17	58239624	162333	RNF190	3	38159138-38
0.25033298	16	11258416	8651	SOCS1	1	44889783-44
0.33702186	1	181826150	4688	NCF2	17	18864772-18
0.26571881	17	72151326	55808	ST6GALNAC1	19	48842368-48
0.30156428	16	31178510	3684	ITGAM	1	117113051-1
0.39336643	1	202595930	22874	PLEKHA6	4	57021952-57

0.32947584	19	52530972	27202	GPR77	1	205144550-2
0.31225241	13	98028005	8428	STK24	17	18864772-18
0.2910437	2	101974781	7850	IL1R2	1	117113051-1
0.33565194	1	36721568	1441	CSF3R	22	30007802-30
0.29111423	13	31787023	675	BRCA2	5	35912241-35
0.24685788	19	50599986	10848	PPP1R13L	5	145949378-1
0.26486824	3	123526489	1475	CSTA	11	60544218-60
0.26041409	20	43316404	6590	SLPI	6	33772190-33
0.27765764	5	154210501	10826	C5orf4	11	60544218-60
0.29272315	17	37196456	3728	JUP	14	90768860-90
0.33151847	1	36721568	1441	CSF3R	17	18864772-18
0.26208224	5	76284393	1393	CRHBP	3	49028156-49
0.31006882	19	19600820	9170	EDG4	1	117113051-1
0.25839429	8	145205121	54512	EXOSC4	10	71569035-71
0.26875231	15	63691065	81556	C15orf44	14	90768860-90
0.2923341	2	241456532	189	AGXT	1	117113051-1
0.33466346	6	41238895	54209	TREM2	17	18864772-18
0.34866519	6	41362863	54210	TREM1	7	149208621-1
0.33621575	11	67534258	221	ALDH3B1	5	136981237-1
0.28928168	4	109034053	166929	MGC26963	7	149208621-1
0.3566144	19	52530972	27202	GPR77	22	30007802-30
0.3073463	19	50599986	10848	PPP1R13L	1	117113051-1
0.29718364	8	145158870	375686	SPATC1	1	117113051-1
0.26181535	2	127129833	2995	GYPC	10	73488877-73
0.34389527	17	53714577	4353	MPO	12	45916614-45
0.28956655	12	53043844	53831	GPR84	11	60544218-60
0.27106352	19	43995615	3960	LGALS4	7	149208621-1
0.35679596	16	56258962	222487	GPR97	22	30007802-30
0.41347024	19	56567263	4818	NKG7	22	30007802-30
0.35744261	17	1611970	5176	SERPINF1	17	18864772-18
0.25944549	2	241456532	189	AGXT	10	73488877-73
0.27386258	19	5802504	2525	FUT3	6	33772190-33
0.33402747	16	56258962	222487	GPR97	1	117113051-1
0.26224911	7	81911509	781	CACNA2D1	14	90768860-90
0.32255968	11	808917	57104	PNPLA2	21	45470779-45
0.34874181	11	67534258	221	ALDH3B1	9	94886967-94
0.3330287	19	13120872	9592	IER2	1	24671778-24
0.32768357	3	133518757	55	ACPP	6	33772190-33
0.31875082	1	36721568	1441	CSF3R	1	117113051-1
0.23651797	4	110844529	839	CASP6	20	3734490-373
0.28411216	19	61711185	57573	ZNF471	1	117113051-1
0.24985505	1	54786297	26027	ACOT11	11	60544218-60
0.30245248	20	43316404	6590	SLPI	12	44868183-44
0.34230588	11	2877365	5002	SLC22A18	12	45916614-45

0.3335162	20	58063710	284756	FLJ33860	12	45916614-45
0.3203875	13	40262245	10166	SLC25A15	1	117113051-1
0.29034795	5	57914758	115827	RAB3C	14	90768860-90
0.34950735	1	205037818	29949	IL19	17	18864772-18
0.25278897	11	59580569	932	MS4A3	11	60544218-60
0.32515663	14	64480232	2877	GPX2	1	117113051-1
0.34458258	11	808917	57104	PNPLA2	6	52236896-52
0.33364803	2	157822650	11227	GALNT5	1	117113051-1
0.37223543	20	24878099	8530	CST7	22	30007802-30
0.24567809	2	101974781	7850	IL1R2	20	3734490-373
0.25824285	12	121473767	6249	RSN	14	90768860-90
0.28355156	11	2877365	5002	SLC22A18	11	60651626-60
0.25896641	14	23107943	8906	AP1G2	6	33772190-33
0.25187132	17	72151326	55808	ST6GALNAC1	6	41351742-41
0.28078017	14	24115961	1511	CTSG	20	3734490-373
0.35398016	14	20493306	6036	RNASE2	4	48582339-48
0.33375933	6	11887927	84830	C6orf105	21	45470779-45
0.27117792	5	39255455	2533	FYB	6	33772190-33
0.34451402	11	125657567	114609	TIRAP	1	154121345-1
0.33864269	11	2877375	5002	SLC22A18	12	45916614-45
0.31956944	1	245648040	114548	CIAS1	17	18864772-18
0.25051892	2	127129833	2995	GYPC	20	3734490-373
0.25146679	16	30390788	3683	ITGAL	6	41351742-41
0.25972178	3	133518757	55	ACPP	10	73488877-73
0.24655539	2	108360946	27233	SULT1C2	20	3734490-373
0.27926249	7	36395645	54443	ANLN	6	33772190-33
0.33254078	10	134108411	170393	C10orf91	6	52236896-52
0.3584308	14	20429255	6037	RNASE3	4	48582339-48
0.28237487	6	32299818	4855	NOTCH4	7	149208621-1
0.25222522	1	159099286	51744	CD244	7	149208621-1
0.28821943	10	44815977	7570	ZNF22	11	60544218-60
0.25262377	16	30390788	3683	ITGAL	19	56941267-56
0.27281344	12	123569171	9612	NCOR2	6	33772190-33
0.28373679	19	15252927	23476	BRD4	11	60544218-60
0.28602311	15	73426486	79661	NEIL1	1	117113051-1
0.24700578	1	159436198	4720	NDUFS2	7	149208621-1
0.25042162	14	103164372	3831	KNS2	10	73488877-73
0.25630671	19	52530972	27202	GPR77	11	60544218-60
0.26919041	1	36721568	1441	CSF3R	20	3734490-373
0.59745727	6	31651544	7124	TNF	11	65838641-65
0.33581115	2	241456532	189	AGXT	17	18864772-18
0.26058559	10	124729456	118672	C10orf89	7	149208621-1
0.36665564	1	181826150	4688	NCF2	22	30007802-30
0.24284048	20	61962518	140701	C20orf135	20	3734490-373

0.3423313	11	1817920	7136	TNNI2	9	94886967-94
0.2874787	6	31662827	7940	LST1	11	60544218-60
0.32783336	2	74913110	3099	HK2	17	18864772-18
0.29993462	3	52296306	132158	GLYCTK	1	24671778-24
0.39234558	11	61861296	80150	ASRGL1	1	23757369-23
0.28080147	18	26876591	1825	DSC3	1	117113051-1
0.31541011	2	108360946	27233	SULT1C2	1	117113051-1
0.24255376	14	22375781	4323	MMP14	16	3059568-305
0.24349015	2	74913110	3099	HK2	20	3734490-373
0.28253044	14	23107943	8906	AP1G2	7	149208621-1
0.27228062	6	37081496	221472	FGD2	11	60544218-60
0.27347559	19	46992652	1084	CEACAM3	7	149208621-1
0.2387388	13	114064381	65110	UPF3A	19	48852486-48
0.31223738	9	139047066	2529	FUT7	17	18864772-18
0.25162991	2	157822650	11227	GALNT5	11	60544218-60
0.40239212	11	2877365	5002	SLC22A18	2	30336048-30
0.24847699	5	76284393	1393	CRHBP	7	149208621-1
0.29648882	3	24512439	7068	THRB	1	117113051-1
0.30606893	3	152529997	53829	P2RY13	6	33772190-33
0.27924958	19	43995615	3960	LGALS4	6	33772190-33
0.34451003	1	92725229	2672	GFI1	12	45916614-45
0.24243554	1	245648040	114548	CIAS1	20	3734490-373
0.29718707	11	67534528	221	ALDH3B1	1	117113051-1
0.27263136	16	21078585	55567	DNAH3	11	60544218-60
0.35347843	11	59708133	64231	MS4A6A	17	18864772-18
0.27370204	13	50537954	2974	GUCY1B2	1	25767674-25
0.24892067	11	2877375	5002	SLC22A18	10	73488877-73
0.25109224	13	50538949	2974	GUCY1B2	20	3734490-373
0.24936256	14	99601789	51466	EVL	6	41351742-41
0.25931751	17	59817773	5175	PECAM1	11	60544218-60
0.27360192	16	56258956	222487	GPR97	11	60544218-60
0.2558825	11	65081734	4054	LTBP3	16	3059568-305
0.28382073	11	56951601	29015	SLC43A3	7	149208621-1
0.29522319	11	65081734	4054	LTBP3	1	117113051-1
0.29260827	11	101291869	253935	ANGPTL5	1	117113051-1
0.37860524	19	56567263	4818	NKG7	12	43188674-43
0.31368582	2	74913110	3099	HK2	1	117113051-1
0.25964767	3	44601496	285349	ZNF660	5	145949378-1
0.31028498	13	50537954	2974	GUCY1B2	1	6444014-644
0.27377423	1	156415852	912	CD1D	7	149208621-1
0.26972418	9	124836630	2844	GPR21	20	3734490-373
0.29744098	5	2804541	153572	IRX2	1	117113051-1
0.2434139	11	808917	57104	PNPLA2	7	149208621-1
0.31022842	22	43451388	55615	PRR5	12	44868183-44

0.2656435	14	20429783	6037	RNASE3	7 149208621-1
0.28553742	10	81699171	6441	SFTPD	11 60544218-60
0.2797775	2	74913110	3099	HK2	10 73488877-73
0.29694898	9	139047066	2529	FUT7	1 117113051-1
0.29387757	19	41091025	7305	TYROBP	11 117715070-1
0.26510683	14	20493306	6036	RNASE2	6 33772190-33
0.34602758	11	1817920	7136	TNNI2	5 136981237-1
0.27904403	19	60484508	23640	HSPBP1	11 60651626-60
0.27150669	5	76284505	1393	CRHBP	11 60544218-60
0.24792875	6	11887927	84830	C6orf105	7 149208621-1
0.30278673	14	103164372	3831	KNS2	1 117113051-1
0.37414345	2	238264500	9208	LRRFIP1	1 117113051-1
0.28473365	15	72005833	4016	LOXL1	11 60651626-60
0.25452386	9	124837105	2844	GPR21	11 60544218-60
0.30425333	11	2877365	5002	SLC22A18	1 117113051-1
0.2797321	11	59708133	64231	MS4A6A	20 3734490-373
0.28721206	19	41091025	7305	TYROBP	11 60544218-60
0.29614313	11	2877375	5002	SLC22A18	11 60651626-60
0.32012385	3	133518757	55	ACPP	11 60651626-60
0.25540579	16	30390788	3683	ITGAL	5 10514353-10
0.26852771	16	70598877	1723	DHODH	11 60544218-60
0.32846295	1	151630113	6279	S100A8	11 60544218-60
0.34364602	16	1605095	57585	CRAMP1L	1 117113051-1
0.27008723	2	241456532	189	AGXT	20 3734490-373
0.28755636	21	44598210	7226	TRPM2	11 60544218-60
0.34885713	13	50538949	2974	GUCY1B2	17 18864772-18
0.25008482	3	160002104	64747	MFSD1	20 3734490-373
0.25465008	2	224974590	79843	FLJ22746	11 60544218-60
0.34316772	12	52977085	4778	NFE2	1 117113051-1
0.33162707	19	40512021	933	CD22	1 117113051-1
0.24830935	9	124836630	2844	GPR21	11 60544218-60
0.3308063	1	27822930	2268	FGR	12 45916614-45
0.25184022	11	65081734	4054	LTBP3	5 145949378-1
0.32395709	11	2877375	5002	SLC22A18	17 18864772-18
0.34011736	9	35608412	971	CD72	11 60544218-60
0.25724037	16	31178510	3684	ITGAM	11 60544218-60
0.24574222	10	73394703	9469	CHST3	14 90768860-90
0.34309305	2	127129833	2995	GYPC	1 117113051-1
0.31088787	3	45163212	64866	CDCP1	1 117113051-1
0.31511963	3	73756760	23024	PDZRN3	1 117113051-1
0.32772462	12	67643600	1368	CPM	17 18864772-18
0.28617926	2	107969437	60482	SLC5A7	1 117113051-1
0.25071865	10	97505362	953	ENTPD1	20 3734490-373
0.29141889	5	76284505	1393	CRHBP	11 117715070-1

0.28419831	12	13139815	83445	GSG1	11	60544218-60
0.2874053	3	152529997	53829	P2RY13	1	117113051-1
0.28971662	16	56258962	222487	GPR97	11	117715070-1
0.34797694	9	124836630	2844	GPR21	1	117113051-1
0.26469578	14	64480232	2877	GPX2	11	60544218-60
0.24715781	18	26876591	1825	DSC3	5	145949378-1
0.30788468	19	59295936	126014	OSCAR	1	117113051-1
0.37001953	1	28375495	5724	PTAFR	1	117113051-1
0.31479335	6	89984204	2569	GABRR1	17	18864772-18
0.34220577	11	2877365	5002	SLC22A18	22	30007802-30
0.29503696	14	20493306	6036	RNASE2	7	149208621-1
0.34689662	17	53714577	4353	MPO	17	18864772-18
0.24335911	1	26070442	164091	PAQR7	7	149208621-1
0.34238352	4	110844529	839	CASP6	1	117113051-1
0.2842276	1	176779118	400798	FLJ35530	20	3734490-373
0.28517459	5	174084385	4488	MSX2	1	117113051-1
0.34084842	9	139047066	2529	FUT7	22	30007802-30
0.33927733	11	2499438	3784	KCNQ1	17	18864772-18
0.25369016	15	27001152	321	APBA2	11	77604355-77
0.26001015	11	32068730	5954	RCN1	6	33772190-33
0.40833075	19	52530972	27202	GPR77	17	18864772-18
0.26286206	16	1815964	81889	FAHD1	14	90768860-90
0.30831672	12	12394113	54682	MANSC1	1	117113051-1
0.29254844	17	1611970	5176	SERPINF1	11	60544218-60
0.31727953	1	154808727	128239	IQGAP3	1	117113051-1
0.28872924	21	36428781	874	CBR3	1	117113051-1
0.27967797	12	6355798	6337	SCNN1A	1	117113051-1
0.40102429	19	60851011	29903	HSU79303	1	117113051-1
0.3101609	12	121473767	6249	RSN	2	242464566-2
0.29007229	14	20429255	6037	RNASE3	6	33772190-33
0.41225031	16	21078585	55567	DNAH3	1	117113051-1
0.30350891	10	99249711	80019	UBTD1	1	117113051-1
0.29804302	19	54707592	2217	FCGRT	1	117113051-1
0.30470999	2	228754650	80309	SKIP	1	117113051-1
0.37495107	13	98028005	8428	STK24	22	30007802-30
0.28243549	1	205037818	29949	IL19	11	60544218-60
0.248049	1	170894886	356	FASLG	19	48852486-48
0.27318782	8	26427270	10687	PNMA2	14	90768860-90
0.25850952	2	108360946	27233	SULT1C2	11	60544218-60
0.26963629	1	245646729	114548	CIAS1	20	3734490-373
0.28825497	17	59437937	3384	ICAM2	1	117113051-1
0.36244876	1	176779118	400798	FLJ35530	1	117113051-1
0.24223093	6	89984204	2569	GABRR1	20	3734490-373
0.35244325	1	7922901	3604	TNFRSF9	22	30007802-30

0.31289319	20	43316404	6590	SLPI	17	18864772-18
0.3281722	3	48576589	90226	UCN2	9	129743305-1
0.31555105	16	2951662	124222	PAQR4	17	18864772-18
0.33588938	4	24404928	6649	SOD3	12	45916614-45
0.36711736	3	133518757	55	ACPP	22	30007802-30
0.35186665	20	61962518	140701	C20orf135	1	117113051-1
0.38089467	13	31787023	675	BRCA2	17	35963692-35
0.40160424	16	56258962	222487	GPR97	14	98705794-98
0.30677346	10	97505212	953	ENTPD1	7	149208621-1
0.26203399	12	67643600	1368	CPM	20	3734490-373
0.33948492	15	73803390	161753	ODF3L1	22	30007802-30
0.32128817	19	5802504	2525	FUT3	17	18864772-18
0.27126128	12	52977085	4778	NFE2	11	60544218-60
0.30184383	16	56258962	222487	GPR97	11	60544218-60
0.35634101	11	61861296	80150	ASRGL1	12	45916614-45
0.33352215	1	36720372	1441	CSF3R	1	117113051-1
0.32064433	14	20429255	6037	RNASE3	7	149208621-1
0.27874368	9	17569227	6456	SH3GL2	1	117113051-1
0.24945801	8	11763503	1508	CTSB	7	149208621-1
0.32039852	1	7922901	3604	TNFRSF9	1	6444014-644
0.28650138	5	39255455	2533	FYB	1	117113051-1
0.35655249	1	24386999	163702	IL28RA	1	117113051-1
0.30205292	11	47356689	6688	SPI1	1	117113051-1
0.33398583	14	93926737	5265	SERPINA1	12	45916614-45
0.34337673	1	154177985	339403	RLN3R2	12	45916614-45
0.3139261	1	151630204	6279	S100A8	11	60544218-60
0.31137934	19	60484508	23640	HSPBP1	1	117113051-1
0.3435517	1	245646729	114548	CIAS1	1	117113051-1
0.26455601	13	50537954	2974	GUCY1B2	11	60544218-60
0.28675552	20	11819375	22903	BTBD3	5	145949378-1
0.327623	11	2877375	5002	SLC22A18	1	117113051-1
0.26116765	6	43720958	221421	C6orf206	16	3059568-305
0.31772897	12	6355798	6337	SCNN1A	17	18864772-18
0.40512608	1	44217190	8704	B4GALT2	4	57021952-57
0.25618058	1	170894886	356	FASLG	5	10514353-10
0.25628416	2	107969437	60482	SLC5A7	5	145949378-1
0.30769628	14	24115961	1511	CTSG	11	60544218-60
0.29753698	20	33666135	6676	SPAG4	1	117113051-1
0.31852401	1	27822930	2268	FGR	17	18864772-18
0.27459149	8	145158870	375686	SPATC1	11	60544218-60
0.29843611	5	178300789	285676	ZNF454	1	117113051-1
0.29267454	12	64869047	11213	IRAK3	1	117113051-1
0.2839863	9	130911839	5524	PPP2R4	1	117113051-1
0.316103	16	27319997	50615	IL21R	1	117113051-1

0.2659248	20	61962518	140701	C20orf135	11	60544218-60
0.38947104	1	205735931	1378	CR1	1	117113051-1
0.37845051	3	133518757	55	ACPP	17	18864772-18
0.33252407	16	56134975	221188	GPR114	12	45916614-45
0.33082412	22	38075476	9145	SYNGR1	1	117113051-1
0.29583684	14	54104965	23034	SAMD4A	1	117113051-1
0.26857504	6	41362863	54210	TREM1	11	60544218-60
0.39114132	13	50537954	2974	GUCY1B2	22	30007802-30
0.26996171	19	10258780	3386	ICAM4	16	3059568-305
0.32940618	11	61861296	80150	ASRGL1	17	18864772-18
0.35221375	12	109424670	144715	RAD9B	12	45916614-45
0.2778807	17	70220621	146722	CD300LF	1	117113051-1
0.34544949	3	52296306	132158	GLYCTK	9	94886967-94
0.34357381	3	152529997	53829	P2RY13	17	18864772-18
0.33584735	19	2232919	374872	C19orf35	12	45916614-45
0.30058745	3	133518757	55	ACPP	20	3734490-373
0.37637524	17	24069170	83871	RAB34	1	117113051-1
0.24955336	11	101291869	253935	ANGPTL5	11	60544218-60
0.30031969	1	36721568	1441	CSF3R	11	60544218-60
0.30715807	20	43316404	6590	SLPI	1	117113051-1
0.25094103	6	27949844	8368	HIST1H4L	11	60544218-60
0.30385412	20	60810316	4923	NTSR1	1	117113051-1
0.25012107	1	154177985	339403	RLN3R2	1	25767674-25
0.25233548	9	139047066	2529	FUT7	11	60544218-60
0.3409288	12	6179035	928	CD9	11	60544218-60
0.34483911	19	46992652	1084	CEACAM3	12	45916614-45
0.30787853	11	808917	57104	PNPLA2	12	44868183-44
0.32318882	19	43995615	3960	LGALS4	17	18864772-18
0.25272563	19	56320008	27180	SIGLEC9	11	60544218-60
0.33196758	5	176869169	79930	DOK3	1	117113051-1
0.39810483	13	50537954	2974	GUCY1B2	17	18864772-18
0.26398553	19	60484508	23640	HSPBP1	20	3734490-373
0.2903275	5	179153151	4056	LTC4S	1	117113051-1
0.34155599	4	109034053	166929	MGC26963	17	18864772-18
0.26406466	3	152529997	53829	P2RY13	20	3734490-373
0.26375665	16	27319997	50615	IL21R	20	3734490-373
0.3758681	12	54646332	1017	CDK2	4	48582339-48
0.2821456	11	808917	57104	PNPLA2	6	33772190-33
0.24015546	9	138763172	158062	LCN6	7	149208621-1
0.33587299	4	8251449	54436	SH3TC1	1	117113051-1
0.2358427	9	130911839	5524	PPP2R4	20	3734490-373
0.27724247	17	31083373	91608	RASL10B	1	117113051-1
0.26741627	2	107969437	60482	SLC5A7	17	31222639-31
0.40201606	6	41362863	54210	TREM1	17	18864772-18

0.33796816	7	36395645	54443	ANLN	17	18864772-18
0.27318077	19	649371	400668	PRSSL1	7	149208621-1
0.36078398	16	54247366	6530	SLC6A2	1	154121345-1
0.25589495	4	101657827	51705	EMCN	11	60544218-60
0.30882948	12	123569171	9612	NCOR2	1	117113051-1
0.32969025	9	138676375	51162	EGFL7	17	18864772-18
0.28955493	6	84197712	4199	ME1	1	117113051-1
0.31537623	19	38378230	4037	LRP3	1	117113051-1
0.36327531	22	20636651	9647	PPM1F	1	117113051-1
0.26705585	14	103164372	3831	KNS2	11	60544218-60
0.32939664	6	146906926	10981	RAB32	1	117113051-1
0.29772323	2	101974781	7850	IL1R2	11	60544218-60
0.25791636	17	59437937	3384	ICAM2	20	3734490-373
0.29228788	2	110230685	7851	MALL	1	117113051-1
0.31194333	16	56134975	221188	GPR114	17	18864772-18
0.3177731	14	20429255	6037	RNASE3	17	18864772-18
0.30744889	5	76284393	1393	CRHBP	6	33772190-33
0.29323812	3	133518757	55	ACPP	11	117715070-1
0.38589964	1	7922901	3604	TNFRSF9	17	18864772-18
0.30676252	1	3558072	7161	TP73	1	117113051-1
0.30117839	15	89248753	4122	MAN2A2	12	44868183-44
0.29630973	19	40512021	933	CD22	11	60544218-60
0.25349901	1	3558072	7161	TP73	5	145949378-1
0.36317565	11	56951601	29015	SLC43A3	12	45916614-45
0.34626733	17	3766139	5023	P2RX1	1	117113051-1
0.33377589	1	154177985	339403	RLN3R2	17	18864772-18
0.29158923	8	76059530	83690	CRISPLD1	1	117113051-1
0.27585423	11	2877365	5002	SLC22A18	11	60544218-60
0.30517965	12	54646332	1017	CDK2	7	149208621-1
0.29838291	14	20429255	6037	RNASE3	1	117113051-1
0.26534642	20	43316404	6590	SLPI	20	3734490-373
0.29270904	1	52381146	9372	ZFYVE9	1	117113051-1
0.24263545	3	130479985	56941	C3orf37	16	28242520-28
0.34606399	19	43995615	3960	LGALS4	22	30007802-30
0.28804289	19	15078781	85360	SYDE1	1	117113051-1
0.30859081	1	176779118	400798	FLJ35530	11	60544218-60
0.29377732	11	44543081	3732	CD82	1	117113051-1
0.31659116	3	6878153	2917	GRM7	1	117113051-1
0.31422235	1	27822930	2268	FGR	1	6444014-644
0.27689373	3	45163212	64866	CDCP1	11	60544218-60
0.33596839	6	43720958	221421	C6orf206	1	117113051-1
0.28376996	10	134108411	170393	C10orf91	6	33772190-33
0.23805325	17	72151326	55808	ST6GALNAC1	1	219054033-2
0.39356038	19	56567263	4818	NKG7	17	35963692-35

0.28383069	1	245646729	114548	CIAS1	11	60544218-60
0.30055704	19	13074451	4066	LYL1	1	117113051-1
0.28261552	11	67534528	221	ALDH3B1	11	60544218-60
0.29219298	1	245648040	114548	CIAS1	11	60544218-60
0.31926163	10	124729456	118672	C10orf89	17	18864772-18
0.34574447	22	43451388	55615	PRR5	22	30007802-30
0.36606241	19	5802504	2525	FUT3	22	30007802-30
0.31065516	12	64869698	11213	IRAK3	1	117113051-1
0.28588627	10	97505362	953	ENTPD1	11	60544218-60
0.28684737	4	171247839	51166	AADAT	1	117113051-1
0.30461496	13	50538949	2974	GUCY1B2	11	60544218-60
0.27933754	2	173648514	51776	ZAK	1	117113051-1
0.27752522	14	57932850	387990	UNQ9438	17	31222639-31
0.30902185	6	146907180	10981	RAB32	1	117113051-1
0.35926498	1	27822930	2268	FGR	22	30007802-30
0.31494335	2	241456532	189	AGXT	11	60544218-60
0.28427516	11	32068730	5954	RCN1	1	117113051-1
0.3179462	14	20429783	6037	RNASE3	17	18864772-18
0.32608521	1	156415852	912	CD1D	17	18864772-18
0.25082252	11	68536712	219928	MRGPRF	7	149208621-1
0.30832028	10	129425888	399823	FLJ46831	1	117113051-1
0.25948146	11	33870664	4005	LMO2	10	73488877-73
0.25068226	19	3711955	9546	APBA3	6	41351742-41
0.27801045	10	72032298	5551	PRF1	5	10514353-10
0.27070104	2	190916477	3628	INPP1	7	149208621-1
0.23670357	3	49566012	8927	BSN	20	3734490-373
0.27376264	3	6878153	2917	GRM7	5	145949378-1
0.24478328	5	76284393	1393	CRHBP	12	51871605-51
0.25709895	21	36428781	874	CBR3	11	60544218-60
0.31604244	11	117640480	10205	EVA1	17	18864772-18
0.27113936	15	72005833	4016	LOXL1	11	60544218-60
0.32327494	1	159099286	51744	CD244	17	18864772-18
0.31139522	22	35734834	339669	MGC35206	17	18864772-18
0.28816379	19	47266036	64763	ZNF574	1	117113051-1
0.26219598	1	152560106	89872	AQP10	11	60544218-60
0.34333206	19	15612949	4051	CYP4F3	17	18864772-18
0.25451291	8	76059530	83690	CRISPLD1	5	145949378-1
0.32075455	11	10429416	272	AMPD3	1	117113051-1
0.33085075	1	199109052	2848	GPR25	1	117113051-1
0.25080727	12	6355798	6337	SCNN1A	11	60544218-60
0.33025398	2	127129833	2995	GYPC	11	60544218-60
0.28492979	1	54293020	9528	TMEM59	1	117113051-1
0.28779854	11	93774294	10888	GPR83	1	117113051-1
0.26314715	2	79593450	1496	CTNNA2	5	145949378-1

0.2794436	3	130479985	56941	C3orf37	1	117113051-1
0.26288364	4	109034053	166929	MGC26963	11	60544218-60
0.32286525	22	35586846	4689	NCF4	1	117113051-1
0.29400176	5	76284393	1393	CRHBP	1	117113051-1
0.28103213	10	129594939	5791	PTPRE	1	117113051-1
0.23589349	16	67072	4350	MPG	20	3734490-373
0.30751078	9	90795819	1903	EDG3	1	117113051-1
0.39133927	17	35147939	2886	GRB7	1	117113051-1
0.33966547	10	124729456	118672	C10orf89	22	30007802-30
0.30010243	14	57932850	387990	UNQ9438	5	145949378-1
0.29149625	12	67643600	1368	CPM	11	60544218-60
0.26248472	19	5802504	2525	FUT3	11	60544218-60
0.34541179	11	59708133	64231	MS4A6A	11	60544218-60
0.3141648	17	67629125	6662	SOX9	1	117113051-1
0.31680062	6	19946190	3400	ID4	1	117113051-1
0.25133321	6	63053922	202559	KHDRBS2	5	145949378-1
0.29199884	11	2877375	5002	SLC22A18	11	60544218-60
0.30601708	19	40939917	126393	HSPB6	1	117113051-1
0.29211603	15	72005833	4016	LOXL1	11	117715070-1
0.29442103	20	58063710	284756	FLJ33860	11	60544218-60
0.2504035	16	54247366	6530	SLC6A2	5	145949378-1
0.25359681	12	123569171	9612	NCOR2	11	60544218-60
0.25163978	11	65424853	8061	FOSL1	12	130973505-1
0.29277716	1	218988746	54996	MOSC2	1	117113051-1
0.35463707	11	56951601	29015	SLC43A3	17	18864772-18
0.26363504	20	33666135	6676	SPAG4	11	60544218-60
0.36744313	19	10258780	3386	ICAM4	1	117113051-1
0.2563569	11	68536712	219928	MRGPRF	6	33772190-33
0.26595914	20	43316404	6590	SLPI	11	60544218-60
0.24446903	2	5750620	6664	SOX11	17	31222639-31
0.24702317	5	179153151	4056	LTC4S	11	60544218-60
0.27587881	20	57016289	1522	CTSZ	11	60544218-60
0.25541547	20	9767805	57144	PAK7	5	145949378-1
0.2795269	10	134108411	170393	C10orf91	11	60651626-60
0.37934136	20	24878099	8530	CST7	17	35963692-35
0.36406139	11	56951601	29015	SLC43A3	22	30007802-30
0.2794701	6	84197716	4199	ME1	1	117113051-1
0.25552501	20	60228263	11255	HRH3	5	145949378-1
0.28808621	12	54646332	1017	CDK2	1	117113051-1
0.35825334	9	134986976	5900	RALGDS	1	117113051-1
0.26694564	11	44543081	3732	CD82	20	3734490-373
0.4133798	14	22375781	4323	MMP14	1	117113051-1
0.30324239	1	27565985	9064	MAP3K6	1	117113051-1
0.26162434	10	53744215	22943	DKK1	5	145949378-1

0.29947016	1	9807209	22883	CLSTN1	1	117113051-1
0.26422169	19	43995615	3960	LGALS4	11	60544218-60
0.29385892	6	89984204	2569	GABRR1	11	60544218-60
0.37821526	1	154177985	339403	RLN3R2	22	30007802-30
0.36535059	6	41238895	54209	TREM2	17	35963692-35
0.29758908	19	13120872	9592	IER2	6	33772190-33
0.27835286	19	17218619	2063	NR2F6	1	117113051-1
0.29281277	16	27319997	50615	IL21R	11	60544218-60
0.25455594	5	178255530	80108	ZFP2	17	31222639-31
0.34371004	2	74913110	3099	HK2	11	60544218-60
0.28743038	14	36121444	26257	NKX2-8	1	117113051-1
0.36855778	19	46992652	1084	CEACAM3	22	30007802-30
0.27693344	11	2510986	3784	KCNQ1	1	117113051-1
0.34936052	1	159436198	4720	NDUFS2	22	30007802-30
0.27723283	5	150380194	2878	GPX3	1	117113051-1
0.37498718	1	44217190	8704	B4GALT2	1	24671778-24
0.25939702	9	37025381	5079	PAX5	5	145949378-1
0.33179722	1	110950438	3737	KCNA2	1	117113051-1
0.27766723	19	40727715	10430	NIFIE14	1	117113051-1
0.30179033	19	60484508	23640	HSPBP1	11	60544218-60
0.33475415	6	11887927	84830	C6orf105	17	18864772-18
0.25910311	1	159099286	51744	CD244	17	77866045-77
0.30914669	2	106870601	84620	ST6GAL2	1	117113051-1
0.25081528	11	44543081	3732	CD82	11	60544218-60
0.24379877	10	11692956	9712	USP6NL	17	31222639-31
0.24821953	11	124272665	54538	ROBO4	11	60544218-60
0.2791837	1	159451716	2207	FCER1G	1	117113051-1
0.25119602	6	110904190	85413	SLC22A16	5	145949378-1
0.35370445	1	159436198	4720	NDUFS2	17	18864772-18
0.37302531	11	56951601	29015	SLC43A3	14	98705794-98
0.34693554	11	1817920	7136	TNNI2	12	45916614-45
0.36544009	10	97505212	953	ENTPD1	17	18864772-18
0.27771559	4	673240	84179	MFSD7	1	117113051-1
0.25250982	11	808917	57104	PNPLA2	20	3734490-373
0.33442165	19	13120872	9592	IER2	7	149208621-1
0.34601687	6	11887927	84830	C6orf105	22	30007802-30
0.2546964	3	45162721	64866	CDCP1	5	145949378-1
0.28255421	1	28791949	115273	RAB42	1	117113051-1
0.30595689	5	135392869	7045	TGFBI	1	117113051-1
0.245452	4	74921592	6372	CXCL6	16	3059568-305
0.38142411	19	46992652	1084	CEACAM3	17	18864772-18
0.31659386	19	5518758	257000	PLAC2	1	117113051-1
0.31188405	9	138127639	138151	BTBD14A	1	117113051-1
0.24701627	12	54646332	1017	CDK2	20	3734490-373

0.2425685	5	57914758	115827	RAB3C	17	31222639-31
0.33672957	7	116750728	7472	WNT2	1	117113051-1
0.31472237	3	152529997	53829	P2RY13	11	60544218-60
0.36596767	3	133518757	55	ACPP	11	60544218-60
0.26322056	1	156415852	912	CD1D	11	60544218-60
0.32458363	16	66120919	79567	FAM65A	1	117113051-1
0.30154132	1	26309828	149420	PDIK1L	1	117113051-1
0.30667795	1	44217190	8704	B4GALT2	9	129743305-1
0.26919255	14	20493306	6036	RNASE2	11	60544218-60
0.39653626	11	2877365	5002	SLC22A18	12	43188674-43
0.28582464	9	102830780	54886	PRG-3	1	117113051-1
0.25007001	6	170457198	84498	KIAA1838	5	145949378-1
0.30350341	19	50599986	10848	PPP1R13L	2	86869963-86
0.3451951	4	57382303	6691	SPINK2	1	117113051-1
0.37263377	13	98028005	8428	STK24	17	35963692-35
0.2464884	1	3557410	49856	WDR8	5	145949378-1
0.30132996	16	66120018	79567	FAM65A	1	117113051-1
0.40641525	19	52530972	27202	GPR77	17	35963692-35
0.25103562	10	128067313	8038	ADAM12	5	145949378-1
0.29842191	7	81911509	781	CACNA2D1	1	117113051-1
0.36447625	3	133518757	55	ACPP	17	35963692-35
0.26330152	14	20429783	6037	RNASE3	11	60544218-60
0.27918511	7	145444372	26047	CNTNAP2	17	31222639-31
0.24744943	19	17274767	79575	ABHD8	5	145949378-1
0.31452031	5	150264697	91975	ZNF300	1	117113051-1
0.33809985	6	41276938	79865	TREML2	1	117113051-1
0.35891878	16	67072	4350	MPG	1	117113051-1
0.33262191	19	649371	400668	PRSSL1	17	18864772-18
0.32803049	3	52504125	23166	STAB1	1	117113051-1
0.35620905	11	67534258	221	ALDH3B1	22	30007802-30
0.3603204	19	1353626	2593	GAMT	1	117113051-1
0.24203045	17	58058114	9902	MRC2	16	3059568-305
0.39802703	16	56134975	221188	GPR114	22	30007802-30
0.31732778	7	100076686	7036	TFR2	1	6444014-644
0.23727434	19	13120872	9592	IER2	20	3734490-373
0.26096236	11	32068730	5954	RCN1	11	60544218-60
0.28536615	22	28206534	4744	NEFH	1	117113051-1
0.35341858	17	77583552	5881	RAC3	1	117113051-1
0.35607486	7	100076686	7036	TFR2	22	30007802-30
0.34605345	3	52235130	54106	TLR9	1	117113051-1
0.28885618	14	20429255	6037	RNASE3	11	60544218-60
0.26369366	11	65081734	4054	LTBP3	1	91920750-91
0.37731963	6	41362863	54210	TREM1	17	35963692-35
0.32887311	19	61711185	57573	ZNF471	12	6757348-675

0.38375371	11	47356570	6688	SPI1		1	117113051-1
0.26025178	9	21995995	1030	CDKN2B		5	145949378-1
0.31515624	9	138763172	158062	LCN6		17	18864772-18
0.2802463	1	154808727	128239	IQGAP3		2	86869963-86
0.27722072	11	68536712	219928	MRGPRF		1	117113051-1
0.28412537	1	32488799	3932	LCK	X		47368761-47
0.32136468	5	39255455	2533	FYB		11	60544218-60
0.29996622	13	98202507	6564	SLC15A1		1	117113051-1
0.33152802	3	52296306	132158	GLYCTK		12	45916614-45
0.41706966	1	181826150	4688	NCF2		17	35963692-35
0.28260668	5	57914758	115827	RAB3C		5	145949378-1
0.28484835	17	43977490	3212	HOXB2		1	117113051-1
0.28357563	7	50829086	2887	GRB10		1	117113051-1
0.27281218	4	3413423	3083	HGFAC		1	24671778-24
0.30216127	19	55613809	6689	SPIB		11	117715070-1
0.29653492	2	74594080	3196	TLX2		1	117113051-1
0.30388732	16	66257644	84080	C16orf48		1	117113051-1
0.38921375	13	50537954	2974	GUCY1B2		17	35963692-35
0.25728603	11	110675314	399948	FLJ45803		5	145949378-1
0.34041795	10	134108411	170393	C10orf91		1	117113051-1
0.31872799	8	55533097	64321	SOX17		1	117113051-1
0.28618537	3	35658819	10777	ARPP-21		9	93211451-93
0.29632579	17	583237	79850	FAM57A		1	117113051-1
0.36215786	11	808917	57104	PNPLA2		1	117113051-1
0.24832941	15	73036967	54913	RPP25		16	3059568-305
0.31249469	7	117300039	83992	CTTNBP2		1	117113051-1
0.3551075	5	127902724	2201	FBN2		1	117113051-1
0.35806609	8	11763503	1508	CTSB		22	30007802-30
0.31826149	1	158883494	6504	SLAMF1		19	56562184-56
0.29176867	19	17274767	79575	ABHD8		16	3059568-305
0.27336489	2	238264500	9208	LRRFIP1		2	86865488-86
0.38594599	17	53714577	4353	MPO		17	35963692-35
0.32106128	5	76284393	1393	CRHBP		20	3734490-373
0.29681732	7	55608208	81552	ECOP		1	117113051-1
0.26938783	10	129594939	5791	PTPRE		11	60544218-60
0.26086422	12	54646332	1017	CDK2		11	60544218-60
0.30130058	10	97505212	953	ENTPD1		11	60544218-60
0.37719532	3	52504564	23166	STAB1		1	117113051-1
0.33819519	2	190916477	3628	INPP1		22	30007802-30
0.33140379	5	138747273	9963	SLC23A1		17	18864772-18
0.30823906	17	7289063	1140	CHRNB1		1	117113051-1
0.37846056	1	7922901	3604	TNFRSF9		17	35963692-35
0.27996299	19	60851011	29903	HSU79303		2	86865488-86
0.24651595	4	19863834	9353	SLIT2		5	145949378-1

0.26902642	1	44217190	8704	B4GALT2	7	149208621-1
0.27587471	11	808917	57104	PNPLA2	11	60544218-60
0.2974046	15	73036967	54913	RPP25	1	117113051-1
0.29396694	10	134108411	170393	C10orf91	20	3734490-373
0.26013369	16	60627573	1006	CDH8	17	31222639-31
0.30447667	5	2804541	153572	IRX2	2	86869963-86
0.28224183	6	11887927	84830	C6orf105	11	60544218-60
0.27738239	5	131591820	8974	P4HA2	1	117113051-1
0.25915949	14	57932850	387990	UNQ9438	1	91920750-91
0.29046491	3	13565419	2199	FBLN2	1	117113051-1
0.37399723	16	31273878	3687	ITGAX	6	31913122-31
0.30934237	11	65081734	4054	LTBP3	2	86869963-86
0.31721684	20	11819375	22903	BTBD3	2	86869963-86
0.29526515	8	145521839	8694	DGAT1	1	117113051-1
0.34846624	5	138747273	9963	SLC23A1	22	30007802-30
0.30460808	17	19231301	4239	MFAP4	1	117113051-1
0.30615631	17	40402398	10882	C1QL1	1	117113051-1
0.27406193	3	52504125	23166	STAB1	11	60544218-60
0.34612684	2	190916477	3628	INPP1	17	18864772-18
0.35503028	19	14648227	84658	EMR3	9	94886967-94
0.37361195	11	1817920	7136	TNNI2	22	30007802-30
0.2880494	12	54646332	1017	CDK2	11	117715070-1
0.35589806	11	65081734	4054	LTBP3	12	6757348-675
0.35460341	16	29731369	112476	PRRT2	1	117113051-1
0.32380215	4	159350966	55314	FLJ11155	2	86865488-86
0.2849388	9	102830780	54886	PRG-3	5	145949378-1
0.35375986	4	74921592	6372	CXCL6	1	117113051-1
0.30862289	7	128364988	3663	IRF5	1	117113051-1
0.28472399	19	10258780	3386	ICAM4	2	86869963-86
0.31185231	18	26876591	1825	DSC3	2	86869963-86
0.29010165	19	40223243	3249	HPN	1	117113051-1
0.32802226	11	118716856	114902	C1QTNF5	1	117113051-1
0.3004563	8	11605305	2626	GATA4	2	86865488-86
0.29651041	4	74953788	2919	CXCL1	2	86869963-86
0.27622642	1	205735931	1378	CR1	2	86865488-86
0.37136183	16	2951662	124222	PAQR4	17	35963692-35
0.31789226	5	76284393	1393	CRHBP	11	60544218-60
0.3293235	4	159350966	55314	FLJ11155	2	86865508-86
0.28979677	17	43977036	3212	HOXB2	2	86865508-86
0.30465027	19	60851011	29903	HSU79303	2	86865508-86
0.28581806	7	70235027	64409	WBSCR17	2	86869963-86
0.37986005	11	1817920	7136	TNNI2	14	98705794-98
0.33809678	1	158883494	6504	SLAMF1	10	72027389-72
0.2772776	9	17569227	6456	SH3GL2	2	86869963-86

0.2938275	22	32646316	9215	LARGE	12	6757348-675
0.29505595	17	58239624	162333	RNF190	11	77604355-77
0.30546096	8	11605305	2626	GATA4	2	86865508-86
0.28547904	11	46310677	8525	DGKZ	17	31222639-31
0.28331723	16	1605095	57585	CRAMP1L	2	86865488-86
0.29574398	10	134108411	170393	C10orf91	11	60544218-60
0.30283448	1	15957345	54751	FBLIM1	1	117113051-1
0.38879081	19	13120872	9592	IER2	17	18864772-18
0.31707512	8	145205121	54512	EXOSC4	17	18864772-18
0.28369062	1	3559246	7161	TP73	1	117113051-1
0.24832678	17	43977490	3212	HOXB2	11	60544218-60
0.28696965	1	3558072	7161	TP73	2	86869963-86
0.2838157	4	4912584	4487	MSX1	1	117113051-1
0.36406145	19	15612949	4051	CYP4F3	17	35963692-35
0.29263131	9	100906254	7046	TGFBR1	1	117113051-1
0.33917531	16	64957821	1003	CDH5	17	18864772-18
0.35318421	3	52296306	132158	GLYCTK	22	30007802-30
0.36779851	11	56951601	29015	SLC43A3	17	35963692-35
0.29627564	16	1605095	57585	CRAMP1L	2	86865508-86
0.36769384	15	64577444	10302	SNAPC5	17	35963692-35
0.280001	8	76059530	83690	CRISPLD1	2	86869963-86
0.29779385	6	44373032	202500	MGC33600	1	117113051-1
0.28136158	15	96305177	91947	ARRDC4	2	86865488-86
0.28584823	3	44601496	285349	ZNF660	2	86865488-86
0.23838473	1	158883494	6504	SLAMF1	19	3728979-372
0.40413912	19	17274767	79575	ABHD8	1	117113051-1
0.27632517	10	128066926	8038	ADAM12	2	86865488-86
0.28322077	8	105548234	1807	DPYS	2	86869963-86
0.38257829	1	154177985	339403	RLN3R2	17	35963692-35
0.26998341	4	5945592	1400	CRMP1	2	86865488-86
0.26197457	6	44373032	202500	MGC33600	16	3059568-305
0.38290451	11	118693355	4162	MCAM	1	117113051-1
0.36838342	19	50787402	2828	GPR4	12	45916614-45
0.26186665	11	68536712	219928	MRGPRF	11	60544218-60
0.35783508	19	14648227	84658	EMR3	4	48582339-48
0.3021078	4	76077131	25849	DKFZP564O0{	1	117113051-1
0.30930904	19	46626153	374907	B3GALT7	1	117113051-1
0.29459202	19	63262280	7694	ZNF135	2	86865508-86
0.36886987	1	159099286	51744	CD244	17	35963692-35
0.34857495	1	53300023	127435	PODN	1	117113051-1
0.26307246	17	70595924	9121	SLC16A5	5	145949378-1
0.28229758	5	178255530	80108	ZFP2	2	86869963-86
0.35242026	1	44217190	8704	B4GALT2	12	45916614-45
0.25444542	3	52235130	54106	TLR9	1	91920750-91

0.30583799	1	1359797	64856	VWA1	1	117113051-1
0.26970291	4	77447378	8987	GENX-3414	2	86865488-86
0.29128633	3	147361774	5352	PLOD2	2	86865488-86
0.28869065	15	73426486	79661	NEIL1	2	86865508-86
0.30098534	15	96305177	91947	ARRDC4	2	86865508-86
0.2886005	4	5945592	1400	CRMP1	2	86865508-86
0.28328501	9	37025381	5079	PAX5	2	86869963-86
0.340905	1	158883494	6504	SLAMF1	19	56566846-56
0.31717698	19	13120872	9592	IER2	11	60544218-60
0.36324695	6	11887927	84830	C6orf105	17	35963692-35
0.31466741	10	129425888	399823	FLJ46831	2	86869963-86
0.30915258	2	149895469	130574	MGC52057	2	86865488-86
0.29444554	20	60228263	11255	HRH3	2	86869963-86
0.31457645	3	44601496	285349	ZNF660	2	86865508-86
0.30301075	3	159771755	4291	MLF1	2	86869963-86
0.29050166	10	26263316	53904	MYO3A	2	86865508-86
0.2928201	5	125958769	501	ALDH7A1	2	86865488-86
0.28535045	4	25266527	10568	SLC34A2	2	86869963-86
0.29998168	2	20714785	64342	HS1BP3	1	117113051-1
0.2702624	4	96689607	8633	UNC5C	2	86865488-86
0.25354809	18	59055398	596	BCL2	11	60544218-60
0.31740331	1	65503082	9829	DNAJC6	2	86865488-86
0.29240674	5	115326619	206338	FLJ90650	2	86869963-86
0.33420953	1	44217190	8704	B4GALT2	17	18864772-18
0.3378598	13	27264814	219409	GSH1	2	86865488-86
0.27504039	9	21995419	1030	CDKN2B	2	86865488-86
0.31529405	4	158216813	2743	GLRB	2	86865488-86
0.3168309	2	149895469	130574	MGC52057	2	86865508-86
0.34641015	18	5533801	23136	EPB41L3	2	86865488-86
0.3142142	10	128066926	8038	ADAM12	2	86865508-86
0.40181243	16	56134975	221188	GPR114	17	35963692-35
0.29725867	22	38075476	9145	SYNGR1	2	86865508-86
0.32279745	1	207915487	50486	G0S2	2	86865488-86
0.29506042	4	16509297	9079	LDB2	2	86865488-86
0.29047172	14	28306074	2290	FOXG1B	17	31222639-31
0.30574066	14	22375781	4323	MMP14	2	86865488-86
0.33010327	3	73756860	23024	PDZRN3	2	86865488-86
0.3838213	16	31274037	3687	ITGAX	17	35963692-35
0.29444397	5	113725531	3781	KCNN2	2	86865488-86
0.3568993	17	70595924	9121	SLC16A5	1	117113051-1
0.29119633	11	46310677	8525	DGKZ	12	6757348-675
0.31855555	3	147361774	5352	PLOD2	2	86865508-86
0.31417882	1	44217190	8704	B4GALT2	1	6444014-644
0.30120166	4	16509297	9079	LDB2	2	86865508-86

0.28867121	11	125657567	114609	TIRAP	2	86865488-86
0.36557362	3	48576589	90226	UCN2	17	35963692-35
0.35426253	18	5533801	23136	EPB41L3	2	86865508-86
0.36633766	3	6878153	2917	GRM7	2	86869963-86
0.28951375	6	43720958	221421	C6orf206	2	86865488-86
0.36240354	8	11763503	1508	CTSB	17	35963692-35
0.33568165	3	73756860	23024	PDZRN3	2	86865508-86
0.28823077	2	5749173	6664	SOX11	2	86869963-86
0.33760302	1	65503082	9829	DNAJC6	2	86865508-86
0.38062164	19	50599986	10848	PPP1R13L	2	86865488-86
0.28609445	10	128067313	8038	ADAM12	2	86869963-86
0.30608264	2	107969437	60482	SLC5A7	2	86865488-86
0.34869556	1	199126988	55765	C1orf106	2	86865488-86
0.30346548	1	242279981	10472	ZNF238	2	86865488-86
0.29621215	9	21995419	1030	CDKN2B	2	86865508-86
0.34196598	1	219027162	64757	MOSC1	2	86869963-86
0.33593302	4	158216813	2743	GLRB	2	86865508-86
0.31972151	5	125958769	501	ALDH7A1	2	86865508-86
0.32448012	14	28306074	2290	FOXG1B	5	145949378-1
0.29618931	4	96689607	8633	UNC5C	2	86865508-86
0.36136686	13	27264814	219409	GSH1	2	86865508-86
0.31487971	19	5518758	257000	PLAC2	2	86869963-86
0.34530636	3	73756760	23024	PDZRN3	2	86865488-86
0.25372961	11	46310677	8525	DGKZ	1	91920750-91
0.27760731	1	6191847	148646	C1orf188	2	86869963-86
0.2992327	16	63714551	1009	CDH11	2	86865488-86
0.29166764	8	57521059	5179	PENK	2	86865508-86
0.28692504	1	177978921	148753	C1orf76	2	86869963-86
0.34435291	1	207915487	50486	G0S2	2	86865508-86
0.28997949	10	20145647	84898	PLXDC2	2	86865488-86
0.37039553	1	158883494	6504	SLAMF1	19	10484758-10
0.29401864	6	19945329	3400	ID4	2	86865508-86
0.30902126	1	242279981	10472	ZNF238	2	86865508-86
0.27802088	10	129425958	399823	FLJ46831	2	86865488-86
0.30439831	6	43720958	221421	C6orf206	2	86865508-86
0.29259582	19	17274767	79575	ABHD8	2	86869963-86
0.33162933	14	22375781	4323	MMP14	2	86865508-86
0.36044958	19	61711185	57573	ZNF471	2	86865488-86
0.27928948	20	54013603	140689	CBLN4	2	86865488-86
0.30770672	5	178300789	285676	ZNF454	2	86865488-86
0.31912154	5	174084385	4488	MSX2	2	86865488-86
0.28262242	15	71447667	10021	HCN4	2	86865488-86
0.30926055	5	170668994	30012	TLX3	2	86865508-86
0.3246707	16	63713342	1009	CDH11	2	86865488-86

0.3284823	20	54012762	140689	CBLN4	2	86865488-86
0.27232021	9	90795819	1903	EDG3	2	86865488-86
0.31507252	11	125657567	114609	TIRAP	2	86865508-86
0.32324597	18	7107680	284217	LAMA1	2	86865488-86
0.38261879	1	44217190	8704	B4GALT2	22	30007802-30
0.32564833	4	74953788	2919	CXCL1	2	86865488-86
0.3199051	5	174084385	4488	MSX2	2	86865508-86
0.29182398	8	23595572	4824	NKX3-1	2	86865488-86
0.32781501	2	107969437	60482	SLC5A7	2	86865508-86
0.40311565	19	50599986	10848	PPP1R13L	2	86865508-86
0.31239128	7	70235027	64409	WBSCR17	2	86865488-86
0.28927434	13	98202507	6564	SLC15A1	2	86869963-86
0.30424401	18	48122676	1630	DCC	2	86865488-86
0.27958113	9	21995563	1030	CDKN2B	2	86865488-86
0.28759133	22	42589512	25830	SULT4A1	2	86865488-86
0.30527162	18	24011209	1000	CDH2	2	86869963-86
0.36577874	1	154808727	128239	IQGAP3	2	86865488-86
0.32034141	16	63714551	1009	CDH11	2	86865508-86
0.27370841	16	54247366	6530	SLC6A2	2	86865488-86
0.37369588	22	20636651	9647	PPM1F	2	86865488-86
0.31070244	1	199109052	2848	GPR25	2	86865488-86
0.33337128	21	26867425	116159	CYYR1	2	86865488-86
0.29141487	7	49784309	375567	UNQ739	2	86869963-86
0.2983983	2	110230685	7851	MALL	2	86865488-86
0.2769272	5	179267849	23061	KIAA0676	2	86865488-86
0.30053499	8	23595572	4824	NKX3-1	2	86865508-86
0.28054913	14	37133863	3169	FOXA1	2	86869963-86
0.33988525	20	54012762	140689	CBLN4	2	86865508-86
0.37700683	5	2804541	153572	IRX2	2	86865488-86
0.33499581	5	178255358	80108	ZFP2	2	86865488-86
0.33946206	17	6620257	162517	FBXO39	2	86865488-86
0.29280666	13	111769787	6656	SOX1	2	86865508-86
0.38558167	1	199126988	55765	C1orf106	2	86865508-86
0.26954762	11	2906389	7262	PHLDA2	2	86865488-86
0.27819561	X	106050345	9075	CLDN2	5	35912241-35
0.27356905	13	87122598	26050	SLITRK5	2	86865488-86
0.29620656	5	178301021	285676	ZNF454	2	86865488-86
0.29329198	8	73150424	8989	TRPA1	2	86865508-86
0.28747024	15	87840913	51458	RHCG	2	86865488-86
0.28735855	4	8633200	27201	GPR78	2	86865488-86
0.30193374	2	210798060	33	ACADL	2	86865508-86
0.27486711	19	61741669	140612	ZFP28	2	86865488-86
0.29849204	5	178301021	285676	ZNF454	2	86865508-86
0.35567158	17	6620321	162517	FBXO39	2	86865488-86

0.32291384	10	20145647	84898	PLXDC2	2	86865508-86
0.30905061	6	84197712	4199	ME1	2	86865488-86
0.33944904	21	26867425	116159	CYYR1	2	86865508-86
0.38997017	19	61711185	57573	ZNF471	2	86865508-86
0.32121555	8	142081035	5747	PTK2	2	86865488-86
0.32345567	4	672834	84179	MFSD7	2	86865488-86
0.34569746	18	7107680	284217	LAMA1	2	86865508-86
0.28961952	18	68362495	147381	CBLN2	2	86865508-86
0.30040005	20	1823084	140885	PTPNS1	2	86865488-86
0.31786608	8	142081035	5747	PTK2	2	86865508-86
0.29840754	11	12355685	55742	PARVA	2	86865488-86
0.3224528	18	48122676	1630	DCC	2	86865508-86
0.31066757	20	54013603	140689	CBLN4	2	86865508-86
0.3142707	10	129425958	399823	FLJ46831	2	86865508-86
0.34769021	2	188865287	51454	GULP1	2	86865488-86
0.3161945	14	36736981	145282	MIPOL1	2	86865488-86
0.35884383	1	199126986	55765	C1orf106	2	86865488-86
0.34580005	18	6404330	91133	L3MBTL4	2	86865488-86
0.3443577	5	178255358	80108	ZFP2	2	86865508-86
0.31294441	15	71447667	10021	HCN4	2	86865508-86
0.39365815	3	73756760	23024	PDZRN3	2	86865508-86
0.2974075	1	110950438	3737	KCNA2	2	86865508-86
0.3084025	17	67629125	6662	SOX9	2	86865488-86
0.3096437	22	42589512	25830	SULT4A1	2	86865508-86
0.34184764	5	178300789	285676	ZNF454	2	86865508-86
0.29077793	4	57382303	6691	SPINK2	2	86865488-86
0.30186282	19	51010588	81492	RSHL1	2	86865508-86
0.29338016	11	131286156	50863	HNT	2	86865488-86
0.36435473	6	146906926	10981	RAB32	2	86865488-86
0.31584146	6	84197712	4199	ME1	2	86865508-86
0.36004553	16	63713342	1009	CDH11	2	86865508-86
0.31554235	6	19946190	3400	ID4	2	86865488-86
0.29060003	20	4178092	146	ADRA1D	2	86865508-86
0.28265057	4	25266527	10568	SLC34A2	2	86865488-86
0.28321709	1	9807209	22883	CLSTN1	2	86865488-86
0.35946833	4	74953788	2919	CXCL1	2	86865508-86
0.36205978	19	10258780	3386	ICAM4	2	86865488-86
0.29890513	2	149894952	130574	MGC52057	2	86865508-86
0.36514658	17	6620321	162517	FBXO39	2	86865508-86
0.28299167	4	48683078	80157	FLJ21511	2	86865488-86
0.31993627	2	110230685	7851	MALL	2	86865508-86
0.32726324	2	79593450	1496	CTNNA2	2	86865488-86
0.39646771	1	154808727	128239	IQGAP3	2	86865508-86
0.29792448	17	70595924	9121	SLC16A5	12	6757348-675

0.31110289	9	21995563	1030	CDKN2B	2	86865508-86
0.36338	1	199126986	55765	C1orf106	2	86865508-86
0.37196023	3	52296306	132158	GLYCTK	17	35963692-35
0.42388432	20	11819375	22903	BTBD3	2	86865488-86
0.27623708	5	50715118	3670	ISL1	2	86869963-86
0.32353916	1	24518437	57822	GRHL3	2	86865508-86
0.28826616	7	145445085	26047	CNTNAP2	2	86865488-86
0.40075414	5	2804541	153572	IRX2	2	86865508-86
0.33697179	1	199109052	2848	GPR25	2	86865508-86
0.36874961	14	57932850	387990	UNQ9438	2	86865488-86
0.33011507	1	24518437	57822	GRHL3	2	86865488-86
0.29433186	2	80385353	347730	LRRTM1	2	86865488-86
0.36283569	17	6620257	162517	FBXO39	2	86865508-86
0.2896577	6	41276938	79865	TREML2	2	86865488-86
0.36649187	6	146906926	10981	RAB32	2	86865508-86
0.40599645	18	26876591	1825	DSC3	2	86865488-86
0.32710665	14	36736981	145282	MIPOL1	2	86865508-86
0.29761585	13	87122598	26050	SLITRK5	2	86865508-86
0.30727898	2	5750620	6664	SOX11	2	86865488-86
0.28940715	7	145445085	26047	CNTNAP2	2	86865508-86
0.28717811	18	490979	81035	COLEC12	2	86865488-86
0.30626166	7	116750728	7472	WNT2	2	86865488-86
0.35349932	7	70235027	64409	WBSCR17	2	86865508-86
0.40872733	22	20636651	9647	PPM1F	2	86865508-86
0.33372076	11	10429416	272	AMPD3	2	86865508-86
0.31934613	1	49014946	79656	C1orf165	2	86865488-86
0.28906966	20	49073405	3755	KCNG1	2	86865508-86
0.30014367	5	113726190	3781	KCNN2	2	86865488-86
0.29834302	19	61741669	140612	ZFP28	2	86865508-86
0.31027603	16	54247366	6530	SLC6A2	2	86865508-86
0.32389382	17	67629125	6662	SOX9	2	86865508-86
0.34628323	4	672834	84179	MFSD7	2	86865508-86
0.31491111	4	8633200	27201	GPR78	2	86865508-86
0.31889678	3	166397165	22865	SLITRK3	2	86865488-86
0.34646038	11	10429416	272	AMPD3	2	86865488-86
0.28956615	13	94162909	11166	SOX21	2	86865488-86
0.32103601	3	159771755	4291	MLF1	2	86865488-86
0.29808873	6	41276938	79865	TREML2	2	86865508-86
0.28407642	7	100156562	2056	EPO	2	86865488-86
0.32191479	5	168660654	6586	SLIT3	2	86865488-86
0.43496898	11	65081734	4054	LTBP3	2	86865488-86
0.31073819	11	93774294	10888	GPR83	2	86865488-86
0.31827073	3	166397165	22865	SLITRK3	2	86865508-86
0.28784905	7	79602227	2770	GNAI1	2	86865488-86

0.3607939	9	17569227	6456	SH3GL2	2	86865488-86
0.43037851	20	11819375	22903	BTBD3	2	86865508-86
0.37032944	18	6404330	91133	L3MBTL4	2	86865508-86
0.3035267	2	106870601	84620	ST6GAL2	2	86865488-86
0.3139072	4	57382303	6691	SPINK2	2	86865508-86
0.31576096	8	26427270	10687	PNMA2	2	86869963-86
0.31489145	11	131286156	50863	HNT	2	86865508-86
0.26949371	1	15958229	54751	FBLIM1	2	86865488-86
0.34213276	2	79593450	1496	CTNNA2	2	86865508-86
0.27822034	5	150380194	2878	GPX3	2	86865488-86
0.3229047	8	32525393	3084	NRG1	2	86865488-86
0.30301904	4	48683078	80157	FLJ21511	2	86865508-86
0.30512439	1	9807209	22883	CLSTN1	2	86865508-86
0.33871664	5	32747994	4883	NPR3	2	86865488-86
0.32588039	3	159771755	4291	MLF1	2	86865508-86
0.36994971	1	3558072	7161	TP73	2	86865488-86
0.29952939	18	68685514	81832	NETO1	2	86865488-86
0.38599924	14	57932850	387990	UNQ9438	2	86865508-86
0.3008103	5	135392869	7045	TGFBI	2	86865488-86
0.29021459	4	164485195	4889	NPY5R	2	86865488-86
0.30443094	5	127902724	2201	FBN2	2	86865488-86
0.32403825	7	116750728	7472	WNT2	2	86865508-86
0.35078702	8	105548234	1807	DPYS	2	86865488-86
0.29510138	7	100156562	2056	EPO	2	86865508-86
0.29764708	7	79602227	2770	GNAI1	2	86865508-86
0.34695838	10	95350726	5950	RBP4	2	86865488-86
0.26963097	5	169863670	30820	KCNIP1	2	86865488-86
0.27997293	8	55533097	64321	SOX17	2	86865488-86
0.42714438	18	26876591	1825	DSC3	2	86865508-86
0.38817129	2	188865287	51454	GULP1	2	86865508-86
0.28094734	1	159451716	2207	FCER1G	2	86865488-86
0.31195335	2	106870601	84620	ST6GAL2	2	86865508-86
0.35175931	8	37942632	155	ADRB3	2	86865488-86
0.29406091	4	164485195	4889	NPY5R	2	86865508-86
0.34806147	22	32646316	9215	LARGE	2	86865488-86
0.30073011	6	84197716	4199	ME1	2	86865488-86
0.34475386	20	1823084	140885	PTPNS1	2	86865508-86
0.2886711	9	21995995	1030	CDKN2B	2	86865488-86
0.31199147	18	490979	81035	COLEC12	2	86865508-86
0.28831898	2	15998211	4613	MYCN	2	86865488-86
0.3553193	19	13074451	4066	LYL1	2	86865508-86
0.33060786	16	66252773	50855	PARD6A	2	86865488-86
0.34344444	11	12355685	55742	PARVA	2	86865508-86
0.28761	1	177978921	148753	C1orf76	2	86865488-86

0.39744712	19	10258780	3386	ICAM4	2	86865508-86
0.30615066	16	60627307	1006	CDH8	2	86865488-86
0.29005776	6	39389863	89822	KCNK17	2	86865488-86
0.27215726	13	98202507	6564	SLC15A1	2	86865488-86
0.3530886	6	19946190	3400	ID4	2	86865508-86
0.29285503	19	61680914	63934	ZNF667	2	86865488-86
0.29977343	1	95164977	1266	CNN3	2	86865508-86
0.32787777	11	93774294	10888	GPR83	2	86865508-86
0.34751545	5	32747994	4883	NPR3	2	86865508-86
0.28557163	1	31618510	2170	FABP3	2	86865488-86
0.34483403	20	9767805	57144	PAK7	2	86865488-86
0.34550283	1	49014946	79656	C1orf165	2	86865508-86
0.34720156	4	171247839	51166	AADAT	2	86865488-86
0.29010573	9	102830899	54886	PRG-3	2	86865488-86
0.32429385	19	5518758	257000	PLAC2	2	86865488-86
0.28407015	4	40954040	7345	UCHL1	2	86865488-86
0.30295162	6	170457198	84498	KIAA1838	2	86865488-86
0.28513685	11	110675314	399948	FLJ45803	2	86865488-86
0.34525481	15	87840913	51458	RHCG	2	86865508-86
0.45403801	11	65081734	4054	LTBP3	2	86865508-86
0.29594265	20	13924190	140733	C20orf133	2	86865488-86
0.33212922	1	218988746	54996	MOSC2	2	86865488-86
0.29565917	19	61680914	63934	ZNF667	2	86865508-86
0.31263394	18	68685514	81832	NETO1	2	86865508-86
0.27874235	16	66257644	84080	C16orf48	2	86865488-86
0.38177862	9	17569227	6456	SH3GL2	2	86865508-86
0.30720532	6	84197716	4199	ME1	2	86865508-86
0.33573378	5	115326619	206338	FLJ90650	2	86865488-86
0.29192367	1	28791949	115273	RAB42	2	86865508-86
0.34357334	20	9767805	57144	PAK7	2	86865508-86
0.29212945	11	65573218	89792	GAL3ST3	2	86865488-86
0.29744611	5	150380194	2878	GPX3	2	86865508-86
0.27943888	3	127596397	348807	CCDC37	2	86865488-86
0.28303373	12	2938338	7004	TEAD4	2	86865488-86
0.3100326	14	36121444	26257	NKX2-8	2	86865508-86
0.27829461	4	177160433	2823	GPM6A	2	86865488-86
0.37999575	19	13074451	4066	LYL1	2	86865488-86
0.3740455	8	76059530	83690	CRISPLD1	2	86865488-86
0.28855577	4	40954040	7345	UCHL1	2	86865508-86
0.31812993	5	135392869	7045	TGFBI	2	86865508-86
0.29484988	1	159451716	2207	FCER1G	2	86865508-86
0.31893937	13	94162909	11166	SOX21	2	86865508-86
0.29860613	7	100091850	51412	ACTL6B	2	86865488-86
0.36752121	8	105548234	1807	DPYS	2	86865508-86

0.29043808	16	60627573	1006	CDH8	2 86865488-86
0.3475375	1	32600427	81629	TSSK3	2 86865488-86
0.33905967	2	80385353	347730	LRRTM1	2 86865508-86
0.2993216	9	102830899	54886	PRG-3	2 86865508-86
0.34007916	5	9598976	9037	SEMA5A	2 86865488-86
0.35127999	2	5750620	6664	SOX11	2 86865508-86
0.29866173	3	114414080	91653	BOC	2 86865488-86
0.39379982	1	3558072	7161	TP73	2 86865508-86
0.3034817	7	81911509	781	CACNA2D1	2 86865488-86
0.36245952	22	32646316	9215	LARGE	2 86865508-86
0.36991532	6	63053922	202559	KHDRBS2	2 86865488-86
0.2923056	5	160907602	2561	GABRB2	2 86865508-86
0.35052063	8	32525393	3084	NRG1	2 86865508-86
0.28748161	11	118693355	4162	MCAM	2 86865488-86
0.31476755	7	81910715	781	CACNA2D1	2 86865488-86
0.30552109	6	39389863	89822	KCNK17	2 86865508-86
0.33340016	5	135392451	7045	TGFBI	2 86865488-86
0.27410165	10	60606632	84457	PHYHIPL	2 86865488-86
0.30064916	12	49949751	57228	LOC57228	2 86865488-86
0.31507973	6	170457198	84498	KIAA1838	2 86865508-86
0.30244741	1	15958229	54751	FBLIM1	2 86865508-86
0.37138107	10	95350726	5950	RBP4	2 86865508-86
0.28398349	11	118716856	114902	C1QTNF5	2 86865488-86
0.35054139	16	66252773	50855	PARD6A	2 86865508-86
0.2827235	5	127901610	2201	FBN2	2 86865488-86
0.29489157	21	38210248	3763	KCNJ6	2 86865488-86
0.32583337	16	60627307	1006	CDH8	2 86865508-86
0.36225449	4	171247839	51166	AADAT	2 86865508-86
0.37281704	10	53744215	22943	DKK1	2 86865488-86
0.39763104	10	129425888	399823	FLJ46831	2 86865488-86
0.34911065	5	113726190	3781	KCNN2	2 86865508-86
0.31267369	20	13924190	140733	C20orf133	2 86865508-86
0.29753632	12	2938338	7004	TEAD4	2 86865508-86
0.28982261	17	7289063	1140	CHRNB1	2 86865508-86
0.30330595	7	117300039	83992	CTTNBP2	2 86865488-86
0.29897047	5	169863670	30820	KCNIP1	2 86865508-86
0.38709271	1	219027162	64757	MOSC1	2 86865488-86
0.3669126	10	53744215	22943	DKK1	2 86865508-86
0.2948118	6	94185610	2045	EPHA7	2 86865508-86
0.30080847	16	60627573	1006	CDH8	2 86865508-86
0.29251337	6	146906222	10981	RAB32	2 86865508-86
0.329746	15	63691065	81556	C15orf44	2 86865488-86
0.28227833	11	106393866	2977	GUCY1A2	2 86865488-86
0.36766826	7	145444372	26047	CNTNAP2	2 86865488-86

0.38323382	8	37942632	155	ADRB3	2	86865508-86
0.37925123	5	178255530	80108	ZFP2	2	86865488-86
0.3093177	5	160907602	2561	GABRB2	2	86865488-86
0.274342	18	30057065	8715	NOL4	2	86865488-86
0.34240805	5	127902724	2201	FBN2	2	86865508-86
0.30158452	16	66257644	84080	C16orf48	2	86865508-86
0.32235337	4	74921592	6372	CXCL6	2	86865488-86
0.34218008	5	135392451	7045	TGFBI	2	86865508-86
0.37495968	1	44217190	8704	B4GALT2	17	35963692-35
0.27240875	5	44425039	2255	FGF10	2	86865488-86
0.35735339	1	218988746	54996	MOSC2	2	86865508-86
0.30798765	10	26263106	53904	MYO3A	2	86865508-86
0.32881625	1	3557410	49856	WDR8	2	86865488-86
0.30924616	12	121473767	6249	RSN	2	86865488-86
0.32237977	18	24011209	1000	CDH2	2	86865488-86
0.31185831	12	49949751	57228	LOC57228	2	86865508-86
0.30982697	2	132890188	2863	GPR39	2	86865488-86
0.30251182	3	127596397	348807	CCDC37	2	86865508-86
0.35258074	19	5518758	257000	PLAC2	2	86865508-86
0.39424421	8	76059530	83690	CRISPLD1	2	86865508-86
0.27481462	20	43974028	5360	PLTP	2	86865488-86
0.28616522	2	176672834	3238	HOXD12	2	86865488-86
0.36098677	5	115326619	206338	FLJ90650	2	86865508-86
0.29972481	14	64002431	9495	AKAP5	2	86865488-86
0.32382132	9	21995995	1030	CDKN2B	2	86865508-86
0.31712192	11	65573218	89792	GAL3ST3	2	86865508-86
0.33944269	2	5749173	6664	SOX11	2	86865488-86
0.30396794	11	118693355	4162	MCAM	2	86865508-86
0.26464931	19	61711185	57573	ZNF471	14	24145611-24
0.32374412	2	15998211	4613	MYCN	2	86865508-86
0.33066773	17	70595924	9121	SLC16A5	2	86869963-86
0.34854556	21	21292108	4685	NCAM2	2	86865488-86
0.2640381	20	11819375	22903	BTBD3	14	24145611-24
0.35214696	5	57914758	115827	RAB3C	2	86865488-86
0.29122422	14	37133863	3169	FOXA1	2	86865488-86
0.28838355	5	1935871	50805	IRX4	2	86865508-86
0.30761715	13	98202507	6564	SLC15A1	2	86865508-86
0.32601876	1	177978921	148753	C1orf76	2	86865508-86
0.32415589	7	100091850	51412	ACTL6B	2	86865508-86
0.31485274	7	117300039	83992	CTTNBP2	2	86865508-86
0.3716469	1	32600427	81629	TSSK3	2	86865508-86
0.3191619	11	110675314	399948	FLJ45803	2	86865508-86
0.45270231	3	6878153	2917	GRM7	2	86865488-86
0.32448259	12	128953814	121256	KIAA1944	2	86865488-86

0.40989732	10	129425888	399823	FLJ46831	2	86865508-86
0.38804453	5	168660654	6586	SLIT3	2	86865508-86
0.39334589	6	63053922	202559	KHDRBS2	2	86865508-86
0.31589096	11	46310677	8525	DGKZ	2	86865488-86
0.376297	7	145444372	26047	CNTNAP2	2	86865508-86
0.33771145	7	81910715	781	CACNA2D1	2	86865508-86
0.30981761	4	177160433	2823	GPM6A	2	86865508-86
0.31642306	12	121473767	6249	RSN	2	86865508-86
0.29657152	3	39826645	25924	MYRIP	2	86865508-86
0.32622925	1	31618510	2170	FABP3	2	86865508-86
0.34361104	15	63691065	81556	C15orf44	2	86865508-86
0.29805447	10	60606632	84457	PHYHIPL	2	86865508-86
0.40012842	1	219027162	64757	MOSC1	2	86865508-86
0.30081688	3	39826645	25924	MYRIP	2	86865488-86
0.3256267	21	30234199	2897	GRIK1	2	86865488-86
0.44296494	3	6878153	2917	GRM7	2	86865508-86
0.39373088	9	37025381	5079	PAX5	2	86865488-86
0.30051474	8	19841725	4023	LPL	2	86865488-86
0.37252391	5	9598976	9037	SEMA5A	2	86865508-86
0.31032641	14	64002431	9495	AKAP5	2	86865508-86
0.35520002	21	21292108	4685	NCAM2	2	86865508-86
0.31542057	4	41058077	22998	DKFZP686A0[illegible]	2	86865488-86
0.32987924	11	123030875	55800	SCN3B	2	86865488-86
0.35459649	10	128067313	8038	ADAM12	2	86865488-86
0.33425071	9	102830780	54886	PRG-3	2	86865488-86
0.29203805	20	43974028	5360	PLTP	2	86865508-86
0.28856159	1	115682737	4803	NGFB	2	86865488-86
0.34243611	4	74921592	6372	CXCL6	2	86865508-86
0.29098625	15	68971644	56906	THAP10	2	86865488-86
0.34157148	7	81911509	781	CACNA2D1	2	86865508-86
0.41438823	20	60228263	11255	HRH3	2	86865488-86
0.33722916	3	114414080	91653	BOC	2	86865508-86
0.27554892	11	65081734	4054	LTBP3	14	24145611-24
0.31670485	11	118716856	114902	C1QTNF5	2	86865508-86
0.32586428	4	85639806	4825	NKX6-1	2	86865488-86
0.34287526	18	24011209	1000	CDH2	2	86865508-86
0.321326	17	58058114	9902	MRC2	2	86865488-86
0.28050381	11	29995364	3739	KCNA4	2	86865488-86
0.40335671	20	60228263	11255	HRH3	2	86865508-86
0.31251577	15	73036967	54913	RPP25	2	86865488-86
0.35220822	1	3557410	49856	WDR8	2	86865508-86
0.37063308	5	57914758	115827	RAB3C	2	86865508-86
0.33385542	2	132890188	2863	GPR39	2	86865508-86
0.36132186	2	5749173	6664	SOX11	2	86865508-86

0.28089076	4	187882442	2195	FAT	2	86865488-86
0.40990743	5	178255530	80108	ZFP2	2	86865508-86
0.33686556	21	30234199	2897	GRIK1	2	86865508-86
0.31732351	8	65873881	9420	CYP7B1	2	86865488-86
0.39537838	3	45162721	64866	CDCP1	2	86865488-86
0.32266163	5	127901610	2201	FBN2	2	86865508-86
0.31412822	15	73036967	54913	RPP25	2	86865508-86
0.40445931	9	37025381	5079	PAX5	2	86865508-86
0.28800855	5	50715118	3670	ISL1	2	86865488-86
0.27584051	4	11039451	9957	HS3ST1	2	86865488-86
0.31623924	2	176672834	3238	HOXD12	2	86865508-86
0.32985833	4	41058077	22998	DKFZP686A0[illegible]	2	86865508-86
0.31890862	8	65873881	9420	CYP7B1	2	86865508-86
0.29275115	4	44145115	386617	KCTD8	2	86865488-86
0.32216793	11	106393866	2977	GUCY1A2	2	86865508-86
0.34169536	21	38210248	3763	KCNJ6	2	86865508-86
0.317023	6	114285706	4082	MARCKS	2	86865508-86
0.39280144	3	45162721	64866	CDCP1	2	86865508-86
0.37007068	10	128067313	8038	ADAM12	2	86865508-86
0.34236443	5	45731679	348980	HCN1	2	86865488-86
0.33272306	17	58058114	9902	MRC2	2	86865508-86
0.27549565	22	42590023	25830	SULT4A1	2	86865488-86
0.29472905	11	29995364	3739	KCNA4	2	86865508-86
0.3109683	22	19121733	91179	SCARF2	2	86865488-86
0.35418126	9	102830780	54886	PRG-3	2	86865508-86
0.32581751	8	26427270	10687	PNMA2	2	86865488-86
0.30044874	4	44145115	386617	KCTD8	2	86865508-86
0.25742182	11	65081734	4054	LTBP3	1	26560769-26
0.35538724	7	49784309	375567	UNQ739	2	86865488-86
0.28965002	4	11039451	9957	HS3ST1	2	86865508-86
0.26303391	2	165186164	2888	GRB14	14	24145611-24
0.3542384	11	46310677	8525	DGKZ	2	86865508-86
0.32902333	16	1815964	81889	FAHD1	2	86865488-86
0.30450862	17	31082710	91608	RASL10B	2	86865508-86
0.36552329	12	128953814	121256	KIAA1944	2	86865508-86
0.31921754	10	73394703	9469	CHST3	2	86865488-86
0.33486204	8	19841725	4023	LPL	2	86865508-86
0.38119484	1	6191847	148646	C1orf188	2	86865488-86
0.36712158	11	123030875	55800	SCN3B	2	86865508-86
0.32696613	1	153290905	8751	ADAM15	2	86865488-86
0.30870425	4	187882442	2195	FAT	2	86865508-86
0.36070962	5	45731679	348980	HCN1	2	86865508-86
0.32576549	1	153290905	8751	ADAM15	2	86865508-86
0.27330826	7	80386631	10512	SEMA3C	14	24145611-24

0.30942739	14	28306074	2290	FOXG1B	2	86865488-86
0.33360694	22	19121733	91179	SCARF2	2	86865508-86
0.36759105	4	19863834	9353	SLIT2	2	86865488-86
0.27036554	7	19123427	7291	TWIST1	2	86865488-86
0.39030382	1	6191847	148646	C1orf188	2	86865508-86
0.34541476	16	1815964	81889	FAHD1	2	86865508-86
0.33565723	1	115682737	4803	NGFB	2	86865508-86
0.34863264	8	26427270	10687	PNMA2	2	86865508-86
0.29785215	17	35110189	2064	ERBB2	2	86865488-86
0.31368548	17	35109904	2064	ERBB2	2	86865488-86
0.30654673	22	42590023	25830	SULT4A1	2	86865508-86
0.37055078	4	19863834	9353	SLIT2	2	86865508-86
0.4285926	19	17274767	79575	ABHD8	2	86865488-86
0.35199733	11	124870348	403312	MGC39545	2	86865488-86
0.32231388	14	28306074	2290	FOXG1B	2	86865508-86
0.31448398	20	1823040	140885	PTPNS1	2	86865488-86
0.28662232	10	35970331	8325	FZD8	2	86865488-86
0.30700772	7	116751038	7472	WNT2	2	86865508-86
0.25586172	2	165186164	2888	GRB14	1	26560769-26
0.3917578	7	49784309	375567	UNQ739	2	86865508-86
0.29067165	3	53504299	776	CACNA1D	2	86865508-86
0.29468893	5	136862762	6695	SPOCK	2	86865508-86
0.30427496	16	67236499	1001	CDH3	2	86865488-86
0.3550359	10	73394703	9469	CHST3	2	86865508-86
0.27691301	1	24518437	57822	GRHL3	14	24145611-24
0.31703478	1	1359797	64856	VWA1	2	86865488-86
0.32913556	17	35109904	2064	ERBB2	2	86865508-86
0.43615871	19	17274767	79575	ABHD8	2	86865508-86
0.2972608	10	35970331	8325	FZD8	2	86865508-86
0.31094149	16	67236499	1001	CDH3	2	86865508-86
0.29782897	1	3557864	7161	TP73	2	86865508-86
0.32859699	20	1823040	140885	PTPNS1	2	86865508-86
0.3241658	17	35110189	2064	ERBB2	2	86865508-86
0.30265809	7	19123427	7291	TWIST1	2	86865508-86
0.32912255	14	57932850	387990	UNQ9438	14	24145611-24
0.27926661	22	32646316	9215	LARGE	14	24145611-24
0.3325084	1	1359797	64856	VWA1	2	86865508-86
0.38633751	11	124870348	403312	MGC39545	2	86865508-86
0.34770723	22	21743260	2781	GNAZ	2	86865488-86
0.28480755	16	54247366	6530	SLC6A2	14	24145611-24
0.35108288	22	21743260	2781	GNAZ	2	86865508-86
0.27851997	1	24518437	57822	GRHL3	1	26560769-26
0.27728593	7	96492068	1749	DLX5	2	86865488-86
0.26902781	11	110675314	399948	FLJ45803	14	24145611-24

0.30537261	7	96492068	1749	DLX5	2	86865508-86
0.45032525	17	70595924	9121	SLC16A5	2	86865488-86
0.26540541	1	95164977	1266	CNN3	1	26560769-26
0.27838992	5	135392451	7045	TGFBI	1	26560769-26
0.26369754	7	81910715	781	CACNA2D1	1	26560769-26
0.44937029	17	70595924	9121	SLC16A5	2	86865508-86
0.26994393	1	6191847	148646	C1orf188	1	26560769-26
0.31485289	3	24511566	7068	THRB	1	26560769-26
0.273834	10	73394703	9469	CHST3	14	24145611-24
0.25578867	1	78284567	54810	GIPC2	1	26560769-26
0.26170114	22	42590023	25830	SULT4A1	1	26560769-26

Xp_cytoband	XpGeneID	XpSymbol	CPG_ISLAND	Distance_to_TSS
24145649-24	2999	GZMH	FALSE	-921
24145649-24	2999	GZMH	FALSE	-576
2p11.2e	925	CD8A	FALSE	120
2p11.2e	925	CD8A	FALSE	10
2p11.2e	925	CD8A	FALSE	120
24145649-24	2999	GZMH	FALSE	611
2p11.2e	925	CD8A	FALSE	-10
2p11.2e	925	CD8A	FALSE	-1244
2p11.2e	925	CD8A	FALSE	10
2p11.2e	925	CD8A	FALSE	-10
2p11.2e	925	CD8A	FALSE	-1244
2p11.2e	925	CD8A	FALSE	409
2p11.2e	925	CD8A	FALSE	594
2p11.2e	925	CD8A	FALSE	-170
2p11.2e	925	CD8A	FALSE	-20
2p11.2e	925	CD8A	FALSE	866
2p11.2e	925	CD8A	FALSE	483
2p11.2e	925	CD8A	FALSE	-170
2p11.2e	925	CD8A	FALSE	594
2p11.2e	925	CD8A	FALSE	409
2p11.2e	925	CD8A	FALSE	230
2p11.2e	925	CD8A	Shore	-69
2p11.2e	925	CD8A	FALSE	113
2p11.2e	925	CD8A	TRUE	390
2p11.2e	925	CD8A	FALSE	483
2p11.2e	925	CD8A	FALSE	866
2p11.2e	925	CD8A	FALSE	-368
15573179-15	83888	FGFBP2	FALSE	-576
16q13d	9289	GPR56	FALSE	-576
16q13d	9289	GPR56	FALSE	-576
2p11.2e	925	CD8A	FALSE	-1318
15573179-15	83888	FGFBP2	FALSE	611
2p11.2e	925	CD8A	TRUE	390
2p11.2e	925	CD8A	FALSE	-68
2p11.2e	925	CD8A	FALSE	-1318
2p11.2e	925	CD8A	Shore	377
2p11.2e	925	CD8A	FALSE	-1128
2p11.2e	925	CD8A	FALSE	551
2p11.2e	925	CD8A	FALSE	366
2p11.2e	925	CD8A	FALSE	-1128
2p11.2e	925	CD8A	FALSE	-68
19p13.2b	53637	S1PR5	Shore	-108
19p13.2b	53637	S1PR5	FALSE	-576

16q13d	9289	GPR56	FALSE	611
2p11.2e	925	CD8A	FALSE	308
15573179-15	83888	FGFBP2	FALSE	131
2p11.2e	925	CD8A	FALSE	524
16q13d	9289	GPR56	FALSE	611
2p11.2e	925	CD8A	FALSE	551
2p11.2e	925	CD8A	Shore	958
2p11.2e	925	CD8A	FALSE	-597
2p11.2e	925	CD8A	Shore	-1331
16q13d	9289	GPR56	FALSE	131
2p11.2e	925	CD8A	FALSE	-19
2p11.2e	925	CD8A	FALSE	-19
2p11.2e	925	CD8A	FALSE	-597
2p11.2e	925	CD8A	Shore	958
2p11.2e	925	CD8A	Shore	-1331
16q13d	9289	GPR56	FALSE	131
2p11.2e	925	CD8A	FALSE	-219
19q13.33d	4818	NKG7	FALSE	-576
85779198-85	10578	GNLY	FALSE	611
19p13.2b	53637	S1PR5	FALSE	611
2p11.2e	925	CD8A	FALSE	-219
2p11.2e	925	CD8A	FALSE	611
2p11.2e	925	CD8A	FALSE	611
2p11.2e	925	CD8A	FALSE	274
2p11.2e	925	CD8A	Shore	-1202
2p11.2e	925	CD8A	FALSE	274
19p13.2b	53637	S1PR5	FALSE	131
10q22.1b	5551	PRF1	FALSE	611
2p11.2e	925	CD8A	FALSE	31
9q22.31a	4783	NFIL3	Shore	-263
2p11.2e	925	CD8A	FALSE	31
19p13.3e	4145	MATK	FALSE	611
19p13.3e	4145	MATK	FALSE	-576
17q21.2a	1236	CCR7	FALSE	-804
2p11.2e	925	CD8A	Shore	-6
2p11.2e	925	CD8A	FALSE	-240
10q22.1b	5551	PRF1	FALSE	-576
6p21.1g	54210	TREM1	Shore	-440
2p11.2e	925	CD8A	FALSE	104
19p13.3e	4145	MATK	FALSE	131
19q13.33d	4818	NKG7	FALSE	131
14q32.2b	64919	BCL11B	FALSE	-721
2p11.2e	925	CD8A	FALSE	-240
17q12b	6352	CCL5	FALSE	-921

2p11.2e	925	CD8A	FALSE	104
10q22.1b	5551	PRF1	FALSE	131
11q13.1d	1521	CTSW	FALSE	-576
1p22.2a-p22.	7049	TGFBR3	FALSE	-576
11q13.1d	1521	CTSW	FALSE	611
5q32d-q32e	5521	PPP2R2B	FALSE	-170
12p13.2c	3824	KLRD1	FALSE	611
1p13.1b	914	CD2	Shore	-646
2p11.2e	925	CD8A	FALSE	-55
17q23.1a	762	CA4	FALSE	-192
19p13.2b	53637	S1PR5	FALSE	-1210
1p13.1b	914	CD2	FALSE	-1318
20p13b	994	CDC25B	FALSE	148
5q32d-q32e	5521	PPP2R2B	FALSE	-799
16q13d	222487	GPR97	FALSE	28
1p22.2a-p22.	7049	TGFBR3	FALSE	611
1p13.1b	914	CD2	FALSE	-1322
5q32d-q32e	5521	PPP2R2B	FALSE	-371
1p13.1b	914	CD2	FALSE	317
2p13.3b	8291	DYSF	FALSE	256
Xp22.2	9185	REPS2	Shore	-670
19q13.33d	4818	NKG7	FALSE	-1210
11q12.2a	923	CD6	Shore	-584
17q23.1a	762	CA4	FALSE	-472
5q32d-q32e	5521	PPP2R2B	TRUE	390
1p13.1b	914	CD2	FALSE	-1128
5p15.2c	83853	ROPN1L	FALSE	-226
17q21.2a	1236	CCR7	FALSE	-412
5q32d-q32e	5521	PPP2R2B	FALSE	-576
17q23.1a	762	CA4	FALSE	137
17q23.1a	762	CA4	Shore	53
12p13.32a	894	CCND2	FALSE	-721
9q22.31a	4783	NFIL3	Shore	-43
5q32d-q32e	5521	PPP2R2B	FALSE	611
19q12c	79156	PLEKHF1	FALSE	611
20p13b	994	CDC25B	FALSE	-57
16q13d	222487	GPR97	FALSE	-303
9q22.31a	4783	NFIL3	Shore	-376
2p11.2e	925	CD8A	FALSE	-1155
12q23.1a	3034	HAL	Shore	-43
1p13.1b	914	CD2	Shore	377
5p15.2c	83853	ROPN1L	FALSE	-272
5q32d-q32e	5521	PPP2R2B	FALSE	24
5p15.2c	83853	ROPN1L	Shore	329

17q21.2a	1236	CCR7	FALSE	39
1p13.1b	914	CD2	FALSE	366
17q23.1a	762	CA4	FALSE	-566
5q32d-q32e	5521	PPP2R2B	FALSE	-1128
1p13.1b	914	CD2	FALSE	-57
14q23.1c	5583	PRKCH	FALSE	-721
6q23.1a-q23.	154075	SAMD3	FALSE	611
1p13.1b	914	CD2	FALSE	-721
11q14.1a	9846	GAB2	Shore	-494
1q41d	64757	MOSC1	FALSE	-822
11q12.2a	923	CD6	FALSE	-34
11q14.1a	9846	GAB2	Shore	-1410
19q13.33e	2357	FPR1	Shore	-1410
5p15.2c	83853	ROPN1L	Shore	15
6p21.1g	54210	TREM1	Shore	15
9q22.31a	4783	NFIL3	FALSE	-252
11q14.1e	54843	SYTL2	FALSE	611
16q13d	222487	GPR97	Shore	53
22q12.2c	113791	PIK3IP1	FALSE	-804
22q12.3d	4689	NCF4	FALSE	-272
5p15.2c	83853	ROPN1L	FALSE	-252
4q12e	84525	HOPX	FALSE	611
19q13.33e	2357	FPR1	Shore	-494
11q14.1a	9846	GAB2	FALSE	222
6p21.1g	54210	TREM1	FALSE	-272
5q32d-q32e	5521	PPP2R2B	FALSE	-147
19p13.3d	116844	LRG1	FALSE	216
22q12.3d	4689	NCF4	Shore	15
9q22.31a	4783	NFIL3	FALSE	-694
3q13.13d-q1	10225	CD96	FALSE	-721
1p13.1b	914	CD2	FALSE	-62
5q32d-q32e	5521	PPP2R2B	FALSE	131
1p13.1b	914	CD2	FALSE	-487
11q12.2a	923	CD6	FALSE	-62
11q12.2a	923	CD6	FALSE	346
2p11.2e	925	CD8A	FALSE	-1155
11q14.1a	9846	GAB2	FALSE	-272
17q12b	6352	CCL5	FALSE	611
5p15.2c	83853	ROPN1L	FALSE	-164
11q14.1a	9846	GAB2	Shore	15
16q13d	222487	GPR97	FALSE	137
Xp22.2	9185	REPS2	FALSE	-147
20p13b	994	CDC25B	Shore	-584
11q12.2a	923	CD6	FALSE	274

16p13.3d	9235	IL32	FALSE	-62
11q14.1a	9846	GAB2	Shore	-376
5q33.3a-q33.	26999	CYFIP2	FALSE	-721
20p13b	994	CDC25B	Shore	-197
11q14.1a	9846	GAB2	Shore	-914
12q23.3c	10970	CKAP4	Shore	-1410
11q12.2a	923	CD6	Shore	718
9q22.31a	4783	NFIL3	FALSE	-823
22q12.3d	4689	NCF4	FALSE	-252
5q32d-q32e	5521	PPP2R2B	FALSE	-597
17q12b	6352	CCL5	FALSE	131
5p15.2c	83853	ROPN1L	Shore	28
8q22.3b	83988	NCALD	FALSE	131
17066900-17	9185	REPS2	Shore	15
20p13b	994	CDC25B	FALSE	346
8q22.3b	83988	NCALD	FALSE	-576
11q14.1a	9846	GAB2	FALSE	469
1q41d	64757	MOSC1	FALSE	-192
16q13d	222487	GPR97	Shore	-10
Xp22.2	9185	REPS2	FALSE	28
4q25f	80216	ALPK1	Shore	-1410
5p15.2c	83853	ROPN1L	FALSE	-147
8q22.3b	83988	NCALD	FALSE	611
11q12.2a	923	CD6	FALSE	-30
17q21.2a	1236	CCR7	FALSE	-7
Xp22.2	9185	REPS2	Shore	-1477
6q23.2b	55350	VNN3	FALSE	28
1p13.1b	914	CD2	FALSE	-597
5p15.2c	83853	ROPN1L	FALSE	266
1p13.1b	914	CD2	FALSE	346
11q12.2a	923	CD6	FALSE	-677
11q14.1a	9846	GAB2	FALSE	256
5p15.2c	83853	ROPN1L	FALSE	-22
Xp22.2	9185	REPS2	FALSE	174
Xp22.2	9185	REPS2	FALSE	-192
11q12.2a	923	CD6	FALSE	539
1q41d	64757	MOSC1	FALSE	-883
11q14.1a	9846	GAB2	FALSE	-164
9q22.31a	4783	NFIL3	FALSE	-313
1p13.1b	914	CD2	Shore	-868
11q12.2a	923	CD6	FALSE	122
11q14.1a	9846	GAB2	FALSE	216
11q12.2a	923	CD6	TRUE	-30
5p15.2c	83853	ROPN1L	FALSE	93

5p15.2c	83853	ROPN1L	TRUE	-378
2p13.3b	8291	DYSF	FALSE	137
22q12.3d	4689	NCF4	FALSE	-193
11q12.2a	923	CD6	FALSE	-59
5q32d-q32e	5521	PPP2R2B	FALSE	-219
Xp22.2	9185	REPS2	FALSE	74
11q12.2a	923	CD6	FALSE	277
1q41d	64757	MOSC1	Shore	53
11q14.1a	9846	GAB2	FALSE	-147
5p15.2c	83853	ROPN1L	FALSE	216
22q12.3d	4689	NCF4	FALSE	174
5p15.2c	83853	ROPN1L	FALSE	-822
17p11.2e	10750	GRAP	FALSE	-34
1p35.1a	1912	PHC2	FALSE	174
11q12.2a	923	CD6	Shore	222
5p15.2c	83853	ROPN1L	FALSE	312
5p15.2c	83853	ROPN1L	FALSE	125
19p13.3d	116844	LRG1	FALSE	-472
16p13.3d	9235	IL32	FALSE	-14
5p15.2c	83853	ROPN1L	FALSE	-1227
1p13.1b	914	CD2	FALSE	539
22q12.3d	4689	NCF4	FALSE	312
1p13.1b	914	CD2	FALSE	-219
11q14.1a	9846	GAB2	Shore	900
11q12.2a	923	CD6	FALSE	39
1q41d	64757	MOSC1	FALSE	-1020
20p13b	994	CDC25B	FALSE	-34
22q12.3d	4689	NCF4	FALSE	-1227
1p35.1a	1912	PHC2	FALSE	28
5p15.2c	83853	ROPN1L	Shore	-180
16q13d	222487	GPR97	FALSE	-257
22q12.3d	4689	NCF4	TRUE	-945
20p13b	994	CDC25B	FALSE	-30
1q41d	64757	MOSC1	FALSE	137
20p13b	994	CDC25B	FALSE	539
9q22.31a	4783	NFIL3	FALSE	-303
22q12.3d	4689	NCF4	FALSE	-192
1q32.1h	9214	FAIM3	FALSE	-34
10q22.1e	9806	SPOCK2	FALSE	346
1q41d	64757	MOSC1	FALSE	-472
9q22.31a	4783	NFIL3	FALSE	-883
1q41d	64757	MOSC1	FALSE	93
11q14.1a	9846	GAB2	FALSE	28
5p15.2c	83853	ROPN1L	FALSE	-544

1p13.1b	914	CD2	FALSE	-779
9q22.31a	4783	NFIL3	FALSE	-428
5p15.2c	83853	ROPN1L	FALSE	-192
20p13b	994	CDC25B	Shore	718
5p15.2c	83853	ROPN1L	TRUE	-945
6p12.2a	4172	MCM3	FALSE	-721
11q12.2a	923	CD6	FALSE	194
Xp22.2	9185	REPS2	Shore	-180
22q12.3d	4689	NCF4	FALSE	-303
6p21.32b	50854	C6orf48	Shore	-939
6p21.1g	54210	TREM1	FALSE	216
11q12.2a	923	CD6	FALSE	285
11q14.1a	9846	GAB2	FALSE	174
Xp22.2	9185	REPS2	FALSE	-472
1q41d	64757	MOSC1	Shore	69
10q24.1b	10023	FRAT1	FALSE	-272
11q14.1a	9846	GAB2	FALSE	-313
1q41d	64757	MOSC1	FALSE	-655
5p15.2c	83853	ROPN1L	FALSE	339
1p13.1b	914	CD2	Shore	718
5p15.2c	83853	ROPN1L	FALSE	-1274
Xp22.2	9185	REPS2	FALSE	-1274
11q12.2a	923	CD6	FALSE	-291
Xp22.2	9185	REPS2	FALSE	-303
11q14.1a	9846	GAB2	FALSE	-1227
5p15.2c	83853	ROPN1L	FALSE	-472
5p13.2c	3575	IL7R	FALSE	-7
11q12.2a	923	CD6	FALSE	310
22q12.3d	4689	NCF4	FALSE	-313
20p13b	994	CDC25B	FALSE	-59
12p13.2a	54682	MANSC1	FALSE	-1227
22q12.2c	113791	PIK3IP1	FALSE	39
22q12.3d	4689	NCF4	Shore	-180
5p15.2c	83853	ROPN1L	FALSE	-303
1p35.1a	1912	PHC2	FALSE	-192
17p11.2e	10750	GRAP	FALSE	39
11q14.1a	9846	GAB2	FALSE	125
19q13.42a	126014	OSCAR	TRUE	731
11q12.2a	923	CD6	Shore	871
17q21.2a	1236	CCR7	FALSE	704
20p13b	994	CDC25B	Shore	222
6q23.2b	55350	VNN3	FALSE	28
1p13.1b	914	CD2	FALSE	322
10q22.1e	9806	SPOCK2	FALSE	539

Xp22.2	9185	REPS2	Shore	53
10q22.1e	9806	SPOCK2	FALSE	-34
22q12.3d	4689	NCF4	FALSE	137
11q12.2a	923	CD6	FALSE	-295
22q12.3d	4689	NCF4	Shore	69
17066900-17	9185	REPS2	FALSE	28
5p15.2c	83853	ROPN1L	FALSE	-1020
11q12.2a	923	CD6	Shore	-962
5p15.2c	83853	ROPN1L	TRUE	-661
11q12.2a	923	CD6	Shore	-859
11q12.2a	923	CD6	FALSE	-31
22q12.3d	4689	NCF4	Shore	53
1p36.11c	864	RUNX3	FALSE	611
19q13.31a	5329	PLAUR	FALSE	-192
1p13.1b	914	CD2	Shore	222
19q13.42a	126014	OSCAR	Shore	54
11q12.2a	923	CD6	FALSE	239
17066900-17	9185	REPS2	FALSE	174
5p15.2c	83853	ROPN1L	Shore	53
22q12.3d	4689	NCF4	FALSE	438
20p13b	994	CDC25B	FALSE	913
11q14.1a	9846	GAB2	FALSE	-192
7q21.3d	25798	BRI3	Shore	-1410
11q14.1a	9846	GAB2	FALSE	-883
11q12.2a	923	CD6	FALSE	179
11q12.2b	921	CD5	FALSE	-34
12p13.2a	54682	MANSC1	FALSE	-313
5p15.2c	83853	ROPN1L	FALSE	438
5p15.2c	83853	ROPN1L	FALSE	189
1p13.1b	914	CD2	Shore	-1151
4q31.1b-q31.	1998	ELF2	FALSE	-147
11q12.2b	921	CD5	FALSE	-30
9q22.31a	4783	NFIL3	Shore	-1267
11q12.2a	923	CD6	FALSE	7
5p15.2c	83853	ROPN1L	FALSE	137
6p21.1g	54210	TREM1	FALSE	-303
11q14.1a	9846	GAB2	FALSE	-303
22q12.3d	4689	NCF4	FALSE	189
5p15.2c	83853	ROPN1L	FALSE	-710
Xp22.2	9185	REPS2	Shore	69
1p13.1b	914	CD2	FALSE	146
1q41d	64757	MOSC1	FALSE	-426
Xp22.2	9185	REPS2	FALSE	-31
5p15.2c	83853	ROPN1L	Shore	69

22q12.3d	4689	NCF4	FALSE	-472
4q31.1b-q31.	1998	ELF2	Shore	-509
1q41d	64757	MOSC1	Shore	49
5p15.2c	83853	ROPN1L	Shore	-1267
19q13.33e	2357	FPR1	FALSE	-303
10q22.1e	9806	SPOCK2	TRUE	-30
11q12.2a	923	CD6	FALSE	78
8q22.3b	83988	NCALD	FALSE	-1210
1p13.1b	914	CD2	FALSE	740
9q34.11a	399665	FAM102A	FALSE	-804
10q22.1e	9806	SPOCK2	Shore	718
11q12.2a	923	CD6	FALSE	5
1p35.1a	1912	PHC2	FALSE	137
17p11.2e	10750	GRAP	FALSE	-412
6p21.1g	54210	TREM1	TRUE	-157
1q41d	64757	MOSC1	FALSE	390
11q12.2a	923	CD6	Shore	-826
7q22.1c	29992	PILRA	FALSE	-192
16p13.3e	51734	SEPX1	Shore	28
1p13.1b	914	CD2	FALSE	913
4q31.1b-q31.	1998	ELF2	FALSE	216
Xp22.2	9185	REPS2	FALSE	137
11q14.1a	9846	GAB2	FALSE	66
20q11.21b	3055	HCK	Shore	-376
1q41d	64757	MOSC1	Shore	-702
22q12.3d	4689	NCF4	FALSE	-566
22q12.3d	4689	NCF4	FALSE	-302
11q12.2a	923	CD6	FALSE	-7
5p15.2c	83853	ROPN1L	FALSE	-106
1p35.1a	1912	PHC2	FALSE	-472
11q14.1a	9846	GAB2	FALSE	-472
2q14.2c	5775	PTPN4	FALSE	611
11q12.2a	923	CD6	FALSE	2
Xp22.2	9185	REPS2	FALSE	-566
11q14.1a	9846	GAB2	FALSE	-428
19p13.3d	116844	LRG1	FALSE	509
22q12.3d	4689	NCF4	FALSE	-1020
5p15.2c	83853	ROPN1L	FALSE	-302
6p21.1g	54210	TREM1	FALSE	68
1p35.1a	1912	PHC2	FALSE	438
1q23.1g	149628	PYHIN1	FALSE	611
1p35.1a	1912	PHC2	Shore	69
1p13.1b	914	CD2	FALSE	-1011
10q22.1e	9806	SPOCK2	Shore	222

19q13.33e	2357	FPR1	Shore	69
19p13.3a	8740	TNFSF14	FALSE	28
5p15.2c	83853	ROPN1L	FALSE	-655
8q22.3b	83988	NCALD	FALSE	611
6p21.1g	54210	TREM1	FALSE	-1274
8q22.3b	83988	NCALD	FALSE	131
19q13.42a	126014	OSCAR	TRUE	133
1p13.1b	914	CD2	FALSE	-872
22q12.3d	4689	NCF4	FALSE	-655
4q31.1b-q31.	1998	ELF2	FALSE	28
5p15.2c	83853	ROPN1L	FALSE	374
22q12.3d	4689	NCF4	FALSE	-472
5p15.2c	83853	ROPN1L	FALSE	93
22q12.3d	4689	NCF4	FALSE	-710
1p13.1b	914	CD2	FALSE	194
11q12.2a	923	CD6	FALSE	704
5p15.2c	83853	ROPN1L	FALSE	-39
22q12.3d	4689	NCF4	Shore	-1267
11q12.2a	923	CD6	FALSE	11
22q12.2c	113791	PIK3IP1	FALSE	-7
11q12.2a	923	CD6	FALSE	-784
20p13b	994	CDC25B	FALSE	363
19q13.31a	5329	PLAUR	FALSE	-472
5p15.2c	83853	ROPN1L	FALSE	-118
17066900-17	9185	REPS2	FALSE	-472
19q13.42a	126014	OSCAR	Shore	33
17p11.2e	10750	GRAP	FALSE	-7
17066900-17	9185	REPS2	FALSE	-303
12p13.2a	54682	MANSC1	Shore	53
10q24.1b	10023	FRAT1	Shore	-1411
4q25f	80216	ALPK1	FALSE	-192
11q14.1a	9846	GAB2	FALSE	374
7q22.1c	29992	PILRA	Shore	53
Xp22.2	9185	REPS2	Shore	49
19q13.33e	2357	FPR1	FALSE	438
20p13b	994	CDC25B	FALSE	78
12p13.2a	54682	MANSC1	FALSE	-472
1p13.1b	914	CD2	FALSE	2
11q12.2b	921	CD5	Shore	871
6q23.2b	55350	VNN3	FALSE	-192
20p13b	994	CDC25B	FALSE	285
22q12.3d	4689	NCF4	FALSE	-39
17066900-17	9185	REPS2	Shore	53
20p13b	994	CDC25B	FALSE	277

Xp22.2	9185	REPS2	FALSE	-212
19q13.31a	5329	PLAUR	FALSE	93
4q31.1b-q31.	1998	ELF2	FALSE	-60
11q12.2b	921	CD5	FALSE	39
5p15.2c	83853	ROPN1L	FALSE	-716
17p11.2e	10750	GRAP	FALSE	-31
11q14.1a	9846	GAB2	Shore	-1267
5p15.2c	83853	ROPN1L	Shore	-10
20p13b	994	CDC25B	Shore	-962
22q12.3d	4689	NCF4	Shore	-10
22q12.3d	4689	NCF4	FALSE	137
11q14.1a	9846	GAB2	Shore	69
12p13.1a	79887	FLJ22662	FALSE	-192
9q22.31a	4783	NFIL3	FALSE	681
5p15.2c	83853	ROPN1L	FALSE	-330
19q13.31a	5329	PLAUR	Shore	53
11q12.2a	923	CD6	Shore	-799
22q12.3d	4689	NCF4	FALSE	-330
1p36.31a	8718	TNFRSF25	FALSE	-7
19q13.33e	2357	FPR1	FALSE	137
5p15.2c	83853	ROPN1L	FALSE	390
17066900-17	9185	REPS2	FALSE	-1274
22q12.3d	4689	NCF4	Shore	-781
5p15.2c	83853	ROPN1L	FALSE	-426
22q12.3d	4689	NCF4	FALSE	-212
11q14.1a	9846	GAB2	Shore	53
5p15.2c	83853	ROPN1L	FALSE	681
12p13.2a	54682	MANSC1	Shore	-1267
10q24.1b	10023	FRAT1	FALSE	312
20q11.21b	3055	HCK	Shore	-599
11q12.2b	921	CD5	Shore	222
20p13b	994	CDC25B	FALSE	-295
12764630-12	3726	JUNB	FALSE	-192
11q14.1a	9846	GAB2	FALSE	-1239
10q23.33d	953	ENTPD1	Shore	15
1p13.1b	914	CD2	FALSE	277
11q14.1a	9846	GAB2	FALSE	-39
19q13.33e	2357	FPR1	FALSE	-31
11q12.2a	923	CD6	FALSE	51
10q24.1b	10023	FRAT1	FALSE	216
11q14.1a	9846	GAB2	FALSE	137
11q14.1a	9846	GAB2	FALSE	-655
4q31.1b-q31.	1998	ELF2	FALSE	74
Xp22.2	9185	REPS2	Shore	-702

12q23.3c	10970	CKAP4	FALSE	-303
11q14.1a	9846	GAB2	FALSE	-106
5p15.2c	83853	ROPN1L	FALSE	-212
11q14.1a	9846	GAB2	FALSE	-566
12p13.2a	54682	MANSC1	Shore	69
11q12.2a	923	CD6	FALSE	-165
Xp22.2	9185	REPS2	FALSE	681
22q12.3d	4689	NCF4	FALSE	390
22q12.3d	4689	NCF4	FALSE	-426
10q24.1b	10023	FRAT1	FALSE	28
10q22.1e	9806	SPOCK2	FALSE	285
22q12.3d	4689	NCF4	FALSE	681
5p15.2c	83853	ROPN1L	FALSE	84
11q12.2a	923	CD6	FALSE	625
17066900-17	9185	REPS2	Shore	69
22q12.3d	4689	NCF4	Shore	49
Xp22.2	9185	REPS2	FALSE	-426
11q14.1a	9846	GAB2	FALSE	-31
11q12.2b	921	CD5	Shore	-962
1q41d	64757	MOSC1	FALSE	-283
19q13.33e	2357	FPR1	Shore	-10
20p13b	994	CDC25B	FALSE	310
22q12.3d	4689	NCF4	FALSE	84
20q11.21b	3055	HCK	TRUE	-378
11q14.1a	9846	GAB2	Shore	-10
7q36.1b	155066	ATP6V0E2	FALSE	-34
11q14.1a	9846	GAB2	FALSE	681
20p13b	994	CDC25B	Shore	-799
20q11.21b	3055	HCK	FALSE	-22
5p15.2c	83853	ROPN1L	FALSE	369
3q13.13d-q1	10225	CD96	Shore	222
22q12.3d	4689	NCF4	Shore	-702
4q31.1b-q31.	1998	ELF2	FALSE	-544
20p13b	994	CDC25B	FALSE	-784
5p15.2c	83853	ROPN1L	Shore	-283
61488171-61	7439	BEST1	FALSE	137
11q14.1a	9846	GAB2	FALSE	-665
17066900-17	9185	REPS2	FALSE	137
10q24.1b	10023	FRAT1	FALSE	-303
4q31.1b-q31.	1998	ELF2	FALSE	66
17p11.2e	10750	GRAP	FALSE	285
19q13.42a	126014	OSCAR	TRUE	148
11q12.2a	923	CD6	FALSE	-951
7q36.1b	155066	ATP6V0E2	FALSE	39

Xp22.2	9185	REPS2	Shore	-594
1p13.1b	914	CD2	FALSE	285
20p13b	994	CDC25B	FALSE	2
5p15.2c	83853	ROPN1L	Shore	-702
1p13.1b	914	CD2	FALSE	-20
6q23.2b	55350	VNN3	FALSE	137
7q21.3d	25798	BRI3	Shore	28
7q36.1b	155066	ATP6V0E2	TRUE	-30
6p21.1g	54210	TREM1	Shore	-10
5p15.2c	83853	ROPN1L	FALSE	-1429
4q31.1b-q31.	1998	ELF2	FALSE	-192
11q12.2b	921	CD5	FALSE	78
10q24.1b	10023	FRAT1	TRUE	-945
11q12.2b	921	CD5	FALSE	-31
17066900-17	9185	REPS2	Shore	-10
1q24.2a	919	CD247	Shore	222
4q31.1b-q31.	1998	ELF2	FALSE	-428
Xp22.2	9185	REPS2	Shore	-1440
10q24.1b	10023	FRAT1	FALSE	-472
11q12.2a	923	CD6	FALSE	386
5p15.2c	83853	ROPN1L	FALSE	509
7q36.1b	155066	ATP6V0E2	FALSE	122
11q12.2b	921	CD5	FALSE	285
19q13.31a	5329	PLAUR	FALSE	-566
20p13b	994	CDC25B	FALSE	179
5p15.2c	83853	ROPN1L	Shore	-1440
19q13.42a	126014	OSCAR	TRUE	-661
20q11.21b	3055	HCK	FALSE	174
11q12.2a	923	CD6	FALSE	10
11q12.2b	921	CD5	FALSE	277
12q23.3c	10970	CKAP4	FALSE	-1020
6p21.32b	50854	C6orf48	FALSE	-7
6p12.2a	4172	MCM3	FALSE	346
4q25f	80216	ALPK1	FALSE	-472
7q22.1c	29992	PILRA	FALSE	-303
1q32.1h	9214	FAIM3	FALSE	-784
Xp22.11a	5165	PDK3	Shore	15
10q22.1e	9806	SPOCK2	FALSE	-7
20q11.21b	3055	HCK	FALSE	-192
4q31.1b-q31.	1998	ELF2	FALSE	-1020
22q12.3d	4689	NCF4	Shore	-1440
5p15.2c	83853	ROPN1L	FALSE	-152
1p34.1f	533	ATP6V0B	FALSE	222
20p13b	994	CDC25B	FALSE	11

12q13.11b	91523	FAM113B	FALSE	-7
17p11.2e	10750	GRAP	FALSE	704
4q31.1b-q31.	1998	ELF2	Shore	53
19q13.42a	126014	OSCAR	Shore	69
19q13.33e	2357	FPR1	FALSE	681
11q12.2b	921	CD5	FALSE	179
10q24.1b	10023	FRAT1	FALSE	-192
17066900-17	9185	REPS2	FALSE	-118
10q24.1b	10023	FRAT1	FALSE	137
16p13.3e	51734	SEPX1	FALSE	-3
4q31.1b-q31.	1998	ELF2	FALSE	-883
11q14.1a	9846	GAB2	FALSE	-426
17066900-17	9185	REPS2	FALSE	390
20p13b	994	CDC25B	FALSE	-165
11q14.1a	9846	GAB2	FALSE	84
11q14.1a	9846	GAB2	Shore	-283
10q24.1b	10023	FRAT1	FALSE	-472
13q12.3c	241	ALOX5AP	Shore	53
6p21.31f	3710	ITPR3	Shore	222
6p12.2a	4172	MCM3	FALSE	539
20q11.21b	3055	HCK	TRUE	-9
16p13.3e	79652	C16orf30	FALSE	-7
1p13.1b	914	CD2	FALSE	11
4q25f	80216	ALPK1	FALSE	137
1p36.11a	84243	ZDHHC18	FALSE	137
7q21.3d	25798	BRI3	FALSE	-192
20p13b	994	CDC25B	FALSE	-7
20p13b	994	CDC25B	FALSE	704
1q32.1h	9214	FAIM3	FALSE	-32
3p25.1d-p25.	9922	IQSEC1	FALSE	222
13q12.3c	241	ALOX5AP	FALSE	-472
12q23.3c	10970	CKAP4	Shore	-1267
3p21.31e	5210	PFKFB4	FALSE	-192
4q31.1b-q31.	1998	ELF2	FALSE	93
22q12.3d	4689	NCF4	FALSE	-152
10q24.1b	10023	FRAT1	Shore	53
22q12.3d	4689	NCF4	FALSE	509
7q22.1c	29992	PILRA	FALSE	-710
17066900-17	9185	REPS2	FALSE	-426
4q31.1b-q31.	1998	ELF2	Shore	-1267
10q24.1b	10023	FRAT1	Shore	69
22q12.2c	113791	PIK3IP1	FALSE	704
5p15.2c	83853	ROPN1L	FALSE	-283
1p13.1b	914	CD2	FALSE	-7

11q12.2b	921	CD5	FALSE	-295
1p13.1b	914	CD2	FALSE	-1155
11q12.2b	921	CD5	FALSE	-7
20q11.21b	3055	HCK	FALSE	-544
3p25.1d-p25.	9922	IQSEC1	Shore	-494
4q31.1b-q31.	1998	ELF2	FALSE	137
17q25.3g	9123	SLC16A3	FALSE	216
17p11.2e	10750	GRAP	FALSE	179
1p36.11d	57185	NPAL3	Shore	-50816909
10q22.1e	9806	SPOCK2	FALSE	11
1q24.2a	919	CD247	FALSE	5
10q22.1e	9806	SPOCK2	FALSE	179
11q12.2a	923	CD6	FALSE	69
6p21.31f	3710	ITPR3	FALSE	78
7q36.1b	155066	ATP6V0E2	FALSE	-31
14q32.2b	64919	BCL11B	FALSE	386
4q31.1b-q31.	1998	ELF2	FALSE	390
1p34.1f	533	ATP6V0B	Shore	15
20q11.21b	3055	HCK	Shore	-180
1p34.1f	533	ATP6V0B	Shore	-376
19q13.31a	5329	PLAUR	FALSE	509
20p13b	994	CDC25B	FALSE	625
5p15.2c	83853	ROPN1L	FALSE	-196
12q23.3c	10970	CKAP4	FALSE	137
5p15.2c	83853	ROPN1L	FALSE	17
17066900-17	9185	REPS2	Shore	-702
20q11.21b	3055	HCK	FALSE	-1274
22q12.3d	4689	NCF4	FALSE	-196
10q24.1b	10023	FRAT1	FALSE	-39
10q22.1e	9806	SPOCK2	FALSE	-7
17p11.2e	10750	GRAP	FALSE	-32
1p13.1b	914	CD2	FALSE	179
6p12.2a	4172	MCM3	FALSE	168
17p11.2e	10750	GRAP	FALSE	-165
20q11.21b	3055	HCK	FALSE	-472
7q36.1b	155066	ATP6V0E2	FALSE	-7
17p13.1c	81565	NDEL1	Shore	53
11q25e	27087	B3GAT1	FALSE	611
7q21.3d	25798	BRI3	FALSE	-883
20q11.21b	3055	HCK	FALSE	-710
11q12.2b	921	CD5	FALSE	11
5p15.2c	83853	ROPN1L	Shore	-1200
Xp11.22b	9500	MAGED1	Shore	222
1p13.1b	914	CD2	FALSE	625

17p11.2e	10750	GRAP	FALSE	-7
12q23.3c	10970	CKAP4	FALSE	-118
22q12.3d	4689	NCF4	FALSE	17
6p21.31f	3710	ITPR3	FALSE	-295
17p11.2e	10750	GRAP	FALSE	11
3q21.3b	80325	ABTB1	FALSE	28
3p25.1d-p25.	9922	IQSEC1	Shore	-43
19q13.12a	7305	TYROBP	Shore	28
11q12.2b	921	CD5	FALSE	704
1p13.1b	914	CD2	FALSE	-165
17066900-17	9185	REPS2	Shore	-1440
20p13b	994	CDC25B	FALSE	51
10q24.1b	10023	FRAT1	Shore	-10
1p13.1b	914	CD2	FALSE	51
7q36.1b	155066	ATP6V0E2	Shore	-859
4q31.1b-q31.	1998	ELF2	FALSE	-212
12q23.3c	10970	CKAP4	FALSE	84
14q11.2b	328	APEX1	FALSE	-34
Xp22.11a	5165	PDK3	FALSE	216
11q12.2a	923	CD6	FALSE	-123
20q11.21b	3055	HCK	FALSE	-948
11q12.2b	921	CD5	FALSE	625
4q25f	80216	ALPK1	FALSE	681
130547276-1	154075	SAMD3	FALSE	611
7q36.1b	155066	ATP6V0E2	FALSE	285
130547276-1	154075	SAMD3	FALSE	611
8q24.13c	114907	FBXO32	FALSE	346
Xp22.11a	5165	PDK3	FALSE	-22
20q11.21b	3055	HCK	FALSE	-3
10q22.1e	9806	SPOCK2	FALSE	-951
5p15.2c	83853	ROPN1L	FALSE	114
1p36.11d	57185	NPAL3	FALSE	274
19q13.32c	29997	GLTSCR2	Shore	-197
26231731-26	8347	HIST1H2BC	FALSE	-472
19q13.12a	7305	TYROBP	FALSE	216
Xp22.11a	5165	PDK3	FALSE	28
4q31.1b-q31.	1998	ELF2	FALSE	681
17p11.2e	10750	GRAP	FALSE	141
6p12.2a	4172	MCM3	Shore	222
7q22.1c	29992	PILRA	FALSE	-426
20q11.21b	3055	HCK	FALSE	-655
20q11.21b	3055	HCK	FALSE	-330
6p12.2a	4172	MCM3	FALSE	322
10q24.1b	10023	FRAT1	FALSE	681

19q13.42a	11025	LILRB3	FALSE	-3
Xp22.11a	5165	PDK3	FALSE	-303
20q11.21b	3055	HCK	FALSE	-566
1p34.1f	533	ATP6V0B	Shore	28
Xp22.11a	5165	PDK3	FALSE	-192
4q31.1b-q31.	1998	ELF2	FALSE	-426
17p11.2e	10750	GRAP	FALSE	386
10q24.1b	10023	FRAT1	FALSE	84
8q24.13c	114907	FBXO32	Shore	718
10q22.1e	9806	SPOCK2	FALSE	51
1p36.11d	57185	NPAL3	FALSE	39
22q12.2c	113791	PIK3IP1	FALSE	386
7q36.1b	155066	ATP6V0E2	FALSE	704
11q12.2b	921	CD5	FALSE	51
10q22.1e	9806	SPOCK2	FALSE	625
20p13b	994	CDC25B	FALSE	386
22q11.21f-q1	5594	MAPK1	FALSE	-192
1p13.1b	914	CD2	FALSE	386
17066900-17	9185	REPS2	FALSE	17
12q13.11b	91523	FAM113B	FALSE	-951
1p36.12a	3399	ID3	FALSE	-32
17p11.2e	10750	GRAP	FALSE	69
1p36.11d	57185	NPAL3	FALSE	-34
17p11.2e	10750	GRAP	FALSE	10
1p13.1b	914	CD2	FALSE	10
10q21.3e	219738	C10orf35	Shore	-939
6p21.31f	3710	ITPR3	FALSE	704
17066900-17	9185	REPS2	Shore	-1200
20p13b	994	CDC25B	FALSE	69
9q33.2b	81571	C9orf45	FALSE	-32
22q11.21f-q1	5594	MAPK1	FALSE	189
10q24.1b	10023	FRAT1	Shore	-283
9q34.11a	399665	FAM102A	FALSE	-7
10q23.33d	953	ENTPD1	FALSE	-472
7q21.3d	25798	BRI3	FALSE	-39
10q24.1b	10023	FRAT1	FALSE	-426
1p34.1f	533	ATP6V0B	Shore	-1088
9q22.33b	55357	TBC1D2	Shore	-599
11q12.2b	921	CD5	FALSE	10
11q12.2b	921	CD5	FALSE	386
10q22.1e	9806	SPOCK2	FALSE	386
10q22.1e	9806	SPOCK2	FALSE	69
17q25.3g	9123	SLC16A3	Shore	69
20q11.21b	3055	HCK	FALSE	681

6p21.31f	3710	ITPR3	FALSE	11
1p34.1f	533	ATP6V0B	FALSE	-192
20q11.21b	3055	HCK	Shore	-702
7q36.1b	155066	ATP6V0E2	FALSE	179
17q25.3g	9123	SLC16A3	FALSE	137
20q11.21b	3055	HCK	FALSE	-426
20q11.21b	3055	HCK	Shore	-283
1p36.11d	57185	NPAL3	TRUE	-30
9q34.11a	399665	FAM102A	FALSE	-449
20p13b	994	CDC25B	FALSE	-123
Xp22.11a	5165	PDK3	FALSE	-472
7q36.1b	155066	ATP6V0E2	FALSE	11
3p25.1d-p25.	9922	IQSEC1	FALSE	28
1p34.1f	533	ATP6V0B	TRUE	-945
7q36.1b	155066	ATP6V0E2	FALSE	-7
7q36.1b	155066	ATP6V0E2	FALSE	-165
Xp22.11a	5165	PDK3	Shore	53
1p34.1f	533	ATP6V0B	FALSE	-162
Xp22.11a	5165	PDK3	FALSE	-883
Xp22.11a	5165	PDK3	FALSE	93
7q21.3d	25798	BRI3	FALSE	681
3q21.3b	80325	ABTB1	FALSE	137
6p12.2a	4172	MCM3	FALSE	277
1p36.11d	57185	NPAL3	FALSE	-7
6p21.31f	3710	ITPR3	FALSE	386
6p21.31f	3710	ITPR3	FALSE	51
20q11.21b	3055	HCK	FALSE	120
10q21.3e	219738	C10orf35	FALSE	39
20q11.21b	3055	HCK	FALSE	-283
17p11.2e	10750	GRAP	FALSE	-123
1p36.11d	57185	NPAL3	Shore	222
9q34.11a	399665	FAM102A	FALSE	704
6p21.31f	3710	ITPR3	FALSE	625
1p36.11d	57185	NPAL3	FALSE	310
20q11.21b	3055	HCK	Shore	-1440
20q11.21b	3055	HCK	Shore	-594
Xp22.11a	5165	PDK3	Shore	69
Xp22.11a	5165	PDK3	Shore	-10
19q13.12a	7305	TYROBP	FALSE	-472
1p34.1f	533	ATP6V0B	FALSE	-303
1p36.11d	57185	NPAL3	FALSE	-31
19q13.42a	11025	LILRB3	FALSE	509
19q13.42a	11025	LILRB3	FALSE	-426
19q13.42a	11025	LILRB3	Shore	-702

1p36.11d	57185	NPAL3	FALSE	78
3p25.1d-p25.	9922	IQSEC1	FALSE	-303
7q36.1b	155066	ATP6V0E2	FALSE	625
3p21.31b	9254	CACNA2D2	FALSE	611
10q21.3e	219738	C10orf35	FALSE	-7
6p12.2a	4172	MCM3	Shore	404
1p34.1f	533	ATP6V0B	FALSE	-710
1p34.1f	533	ATP6V0B	FALSE	-472
1p34.1f	533	ATP6V0B	Shore	53
7q36.1b	155066	ATP6V0E2	FALSE	51
Xp22.11a	5165	PDK3	FALSE	681
10q24.1b	10023	FRAT1	FALSE	114
3p25.1d-p25.	9922	IQSEC1	Shore	53
7q36.1b	155066	ATP6V0E2	FALSE	10
7q36.1b	155066	ATP6V0E2	FALSE	386
1p36.11d	57185	NPAL3	FALSE	704
14q11.2b	328	APEX1	FALSE	-784
19q13.12a	7305	TYROBP	FALSE	233
7q36.1b	155066	ATP6V0E2	FALSE	69
1p36.11d	57185	NPAL3	Shore	-859
1p34.1f	533	ATP6V0B	FALSE	-39
17q25.1c	2885	GRB2	Shore	69
1p36.11d	57185	NPAL3	FALSE	285
1p34.1f	533	ATP6V0B	FALSE	-566
6p21.31f	3710	ITPR3	FALSE	-123
Xp22.11a	5165	PDK3	FALSE	-426
9p13.3a	152007	GLIPR2	FALSE	-192
1p36.11d	57185	NPAL3	FALSE	-295
15q15.1b	27079	RPUSD2	Shore	222
Xp22.11a	5165	PDK3	Shore	-283
9p13.3a	152007	GLIPR2	Shore	-180
9p13.3a	152007	GLIPR2	FALSE	189
1p34.1f	533	ATP6V0B	Shore	-702
19q13.12a	7305	TYROBP	FALSE	509
1p34.1f	533	ATP6V0B	FALSE	-426
1q25.3f	81563	C1orf21	FALSE	611
3p25.1d-p25.	9922	IQSEC1	FALSE	-212
6p12.2a	4172	MCM3	FALSE	-951
1p36.11d	57185	NPAL3	FALSE	179
8q24.13c	114907	FBXO32	FALSE	179
1p36.11d	57185	NPAL3	FALSE	-165
9p13.3a	152007	GLIPR2	FALSE	-472
1p36.11d	57185	NPAL3	FALSE	-7
1p36.11d	57185	NPAL3	FALSE	11

8q24.13c	114907	FBXO32	FALSE	-7
1p36.11d	57185	NPAL3	FALSE	386
1p36.11d	57185	NPAL3	FALSE	51
1p36.11d	57185	NPAL3	FALSE	-951
8q24.13c	114907	FBXO32	FALSE	51
6p12.2a	4172	MCM3	FALSE	-123
1p36.11d	57185	NPAL3	FALSE	69
10q21.3e	219738	C10orf35	FALSE	10
1p36.11d	57185	NPAL3	FALSE	-123
15q15.1b	27079	RPUSD2	Shore	-575
1p36.21a	79814	AGMAT	FALSE	189
1p36.11d	57185	NPAL3	Shore	-1200
10q21.3e	219738	C10orf35	Shore	-575
1p36.11d	57185	NPAL3	FALSE	540
1p36.11d	57185	NPAL3	FALSE	17
10q21.3e	219738	C10orf35	FALSE	-283
6p12.2a	4172	MCM3	FALSE	114
15q15.1b	27079	RPUSD2	FALSE	681
1p36.11d	57185	NPAL3	FALSE	-196
10q21.3e	219738	C10orf35	Shore	-1440
1p36.11d	57185	NPAL3	Shore	-575
1p36.11d	57185	NPAL3	FALSE	-283
10q21.3e	219738	C10orf35	Shore	-594
8q13.3c	9242	MSC	TRUE	94
10q21.3e	219738	C10orf35	Shore	-184
6p12.2a	4172	MCM3	FALSE	17
10q21.3e	219738	C10orf35	Shore	-4
6p12.2a	4172	MCM3	Shore	-1200
10q21.3e	219738	C10orf35	FALSE	509
1p36.11d	57185	NPAL3	FALSE	60
4q12e	10606	PAICS	Shore	-283
1p36.11d	57185	NPAL3	Shore	-184
15q15.1b	27079	RPUSD2	FALSE	-655
1p36.11d	57185	NPAL3	Shore	-1440
1p36.11d	57185	NPAL3	Shore	-283
15q15.1b	27079	RPUSD2	FALSE	-39
6p12.2a	4172	MCM3	Shore	-575
4q31.21b-q3:	8821	INPP4B	Shore	-1035
1p36.11d	57185	NPAL3	FALSE	84
1p36.11d	57185	NPAL3	FALSE	-212
15q15.1b	27079	RPUSD2	Shore	69
1p36.11d	57185	NPAL3	Shore	-594
15q15.1b	27079	RPUSD2	FALSE	93
17q25.1d	6730	SRP68	FALSE	-303

7q22.1b	10898	CPSF4	FALSE	-883
8q13.3c	9242	MSC	TRUE	78
8q24.13c	114907	FBXO32	Shore	-4
8q13.3c	9242	MSC	TRUE	405
10q21.3e	219738	C10orf35	FALSE	-31
1p36.11d	57185	NPAL3	Shore	49
2q21.3b	1615	DARS	Shore	-4
1p36.11d	57185	NPAL3	FALSE	509
7q22.1b	10898	CPSF4	FALSE	-192
4q12e	10606	PAICS	Shore	-1035
1p36.11d	57185	NPAL3	FALSE	-716
15q15.1b	27079	RPUSD2	FALSE	-883
2q21.3b	1615	DARS	FALSE	509
15q15.1b	27079	RPUSD2	FALSE	-303
1p36.11d	57185	NPAL3	FALSE	137
21q22.3e	104	ADARB1	FALSE	-283
10q21.3e	219738	C10orf35	FALSE	-710
4q12e	10606	PAICS	FALSE	509
1p36.11d	57185	NPAL3	FALSE	681
6p12.2a	4172	MCM3	Shore	-1440
1p36.11d	57185	NPAL3	Shore	-4
7q36.1b	155066	ATP6V0E2	FALSE	114
10q21.3e	219738	C10orf35	Shore	69
10q21.3e	219738	C10orf35	FALSE	1
15q15.1b	27079	RPUSD2	FALSE	-192
6p12.2a	4172	MCM3	FALSE	-1429
10q21.3e	219738	C10orf35	FALSE	-1020
4q12e	10606	PAICS	FALSE	374
10q21.3e	219738	C10orf35	FALSE	-3
6p12.2a	4172	MCM3	Shore	-4
14q11.2b	328	APEX1	FALSE	-1239
1p36.11d	57185	NPAL3	FALSE	877
1p36.11d	57185	NPAL3	FALSE	-330
1p36.11d	57185	NPAL3	Shore	-1267
10q26.11d	9531	BAG3	FALSE	509
1p36.11d	57185	NPAL3	FALSE	-31
2q21.3b	1615	DARS	Shore	-781
4q12e	10606	PAICS	FALSE	-472
4q12e	10606	PAICS	FALSE	-566
1q25.3f	81563	C1orf21	TRUE	168
6p12.2a	4172	MCM3	FALSE	369
1q25.3f	81563	C1orf21	TRUE	207
1p36.11d	57185	NPAL3	FALSE	298
1p36.11d	57185	NPAL3	FALSE	374

1p36.11d	57185	NPAL3	FALSE	233
7q22.1b	10898	CPSF4	FALSE	28
1q25.3f	81563	C1orf21	TRUE	74
1p36.11d	57185	NPAL3	FALSE	-665
1p36.11d	57185	NPAL3	FALSE	-710
4q12e	10606	PAICS	FALSE	-106
6p12.2a	4172	MCM3	FALSE	84
1p36.11d	57185	NPAL3	FALSE	93
6p12.2a	4172	MCM3	Shore	-283
10q21.3e	219738	C10orf35	Shore	-172
1p36.11d	57185	NPAL3	FALSE	-655
4q12e	10606	PAICS	FALSE	-710
10q22.1a	219743	TYSND1	FALSE	-883
1p36.11d	57185	NPAL3	Shore	-781
2q21.3b	1615	DARS	FALSE	-710
2q21.3b	1615	DARS	FALSE	-192
8q13.3c	9242	MSC	TRUE	8
2q21.3b	1615	DARS	FALSE	137
2q21.3b	1615	DARS	FALSE	-1239
1p36.11d	57185	NPAL3	Shore	564
8q13.3c	9242	MSC	TRUE	3
2q21.3b	1615	DARS	Shore	69
1p36.11d	57185	NPAL3	FALSE	-1274
1p36.11d	57185	NPAL3	FALSE	1
5q31.2b	26249	KLHL3	FALSE	509
14q11.2b	328	APEX1	FALSE	-883
9q22.31b	203328	SUSD3	Shore	-594
1p36.11d	57185	NPAL3	FALSE	264
14q11.2b	328	APEX1	FALSE	-472
6p12.2a	4172	MCM3	Shore	-10
7q36.1b	155066	ATP6V0E2	Shore	-1200
7q36.1b	155066	ATP6V0E2	FALSE	17
6p12.2a	4172	MCM3	FALSE	-330
1p36.11d	57185	NPAL3	Shore	-321
8q13.3c	9242	MSC	TRUE	-228
8q24.13c	114907	FBXO32	Shore	-322
1p36.11d	57185	NPAL3	Shore	-492
8q13.3c	9242	MSC	TRUE	-45926892
1p36.11d	57185	NPAL3	FALSE	-1020
9q34.11a	399665	FAM102A	Shore	-594
8q13.3c	9242	MSC	TRUE	74
1p36.11d	57185	NPAL3	FALSE	189
1q44e	114548	NLRP3	FALSE	11
6p12.2a	4172	MCM3	FALSE	298

1p36.11d	57185	NPAL3	FALSE	-1245
1p36.11d	57185	NPAL3	FALSE	275
7q36.1b	155066	ATP6V0E2	FALSE	-283
6p21.31f	3710	ITPR3	Shore	-1200
15q15.1b	27079	RPUSD2	FALSE	174
7q22.1b	10898	CPSF4	FALSE	-823
7q36.1b	155066	ATP6V0E2	FALSE	-196
15q15.1b	27079	RPUSD2	FALSE	-313
6p12.2a	4172	MCM3	FALSE	-655
7q22.1b	10898	CPSF4	FALSE	-313
10q21.3e	219738	C10orf35	FALSE	-806
10q21.3e	219738	C10orf35	FALSE	573
4q12e	10606	PAICS	FALSE	-1274
10q22.1a	219743	TYSND1	FALSE	-192
1p36.11d	57185	NPAL3	FALSE	-1282
6p12.2a	4172	MCM3	FALSE	137
4q12e	10606	PAICS	FALSE	2499438
1p36.11d	57185	NPAL3	FALSE	-39
6p12.2a	4172	MCM3	FALSE	93
8q24.13c	114907	FBXO32	Shore	-321
1p36.11d	57185	NPAL3	FALSE	-472
3p14.3a	2317	FLNB	FALSE	-1282
1p36.11d	57185	NPAL3	FALSE	-1239
6p12.2a	4172	MCM3	FALSE	-716
7q36.1b	155066	ATP6V0E2	Shore	-575
1p36.11d	57185	NPAL3	FALSE	162
8q13.3c	9242	MSC	TRUE	29
1p36.11d	57185	NPAL3	Shore	-655
6p21.31f	3710	ITPR3	Shore	-575
1p36.11d	57185	NPAL3	FALSE	-665
48589546-48	132299	OCIAD2	Shore	-594
26305047-26	8351	HIST1H3D	FALSE	-32
1p13.1b	914	CD2	FALSE	114
1p36.11d	57185	NPAL3	FALSE	573
7q36.1b	155066	ATP6V0E2	FALSE	-426
6p12.2a	4172	MCM3	FALSE	283
7q22.1b	10898	CPSF4	Shore	-509
1p36.11d	57185	NPAL3	Shore	217
3p14.2d-p14.	2272	FHIT	FALSE	233
6p21.31f	3710	ITPR3	FALSE	-283
1p36.11d	57185	NPAL3	FALSE	-3
1p36.11d	57185	NPAL3	FALSE	-544
4q12e	10606	PAICS	Shore	-968
10q22.1a	219743	TYSND1	FALSE	-313

9q34.11a	399665	FAM102A	FALSE	509
15q15.1b	27079	RPUSD2	Shore	-312
7q36.1b	155066	ATP6V0E2	FALSE	369
1p36.11d	57185	NPAL3	FALSE	66
6p12.2a	4172	MCM3	FALSE	-39
4q12e	10606	PAICS	FALSE	93
10q22.1a	219743	TYSND1	FALSE	174
17q25.1d	6730	SRP68	FALSE	381
1p36.11d	57185	NPAL3	Shore	-180
Xp22.11a	5165	PDK3	FALSE	-784
10p13c-p13b	221061	FAM171A1	Shore	-208
1p36.11d	57185	NPAL3	FALSE	189
4q12e	10606	PAICS	Shore	-208
6p12.2a	4172	MCM3	FALSE	374
1p36.11d	57185	NPAL3	FALSE	174
6p12.2a	4172	MCM3	Shore	-321
12q23.3c	10970	CKAP4	FALSE	69
1p36.11d	57185	NPAL3	FALSE	28
6p21.31f	3710	ITPR3	Shore	-594
1p36.11d	57185	NPAL3	TRUE	-945
7q36.1b	155066	ATP6V0E2	Shore	-594
1p36.11d	57185	NPAL3	Shore	-245
6p12.2a	4172	MCM3	FALSE	-303
16q22.1f	16	AARS	Shore	69
7q36.1b	155066	ATP6V0E2	Shore	-1440
9q22.31b	203328	SUSD3	FALSE	-710
1p36.11d	57185	NPAL3	FALSE	-162
6p12.2a	4172	MCM3	Shore	-492
1p36.11d	57185	NPAL3	FALSE	-428
1p36.11d	57185	NPAL3	FALSE	-806
10q21.3e	219738	C10orf35	FALSE	230
48589546-48	132299	OCIAD2	Shore	-283
6p21.31f	3710	ITPR3	Shore	-1440
6p12.2a	4172	MCM3	FALSE	-710
4q12e	10606	PAICS	Shore	-1088
1p36.11d	57185	NPAL3	FALSE	-883
1p13.1b	914	CD2	TRUE	-4
1p36.11d	57185	NPAL3	FALSE	-822
20p13e	128646	SIRPD	FALSE	322
1p36.11d	57185	NPAL3	FALSE	-192
1p36.31b	6146	RPL22	FALSE	509
1p36.11d	57185	NPAL3	Shore	-592
6p12.2a	4172	MCM3	Shore	-180
1p36.11d	57185	NPAL3	FALSE	-303

1p36.11d	57185	NPAL3	Shore	-968
7q36.1b	155066	ATP6V0E2	Shore	-184
7q36.1b	155066	ATP6V0E2	FALSE	509
10q22.1a	219743	TYSND1	FALSE	-193
6p12.2a	4172	MCM3	FALSE	-472
16q22.1f	16	AARS	FALSE	-883
7q22.1b	10898	CPSF4	FALSE	27
1p36.11d	57185	NPAL3	FALSE	4
19q13.33d	284367	SIGLECP3	TRUE	465
1p36.11d	57185	NPAL3	Shore	71
6p12.2a	4172	MCM3	FALSE	-544
7q36.1b	155066	ATP6V0E2	Shore	-283
1p13.1b	914	CD2	FALSE	17
9q22.31b	203328	SUSD3	FALSE	233
1p36.11d	57185	NPAL3	FALSE	216
1p36.11d	57185	NPAL3	FALSE	-152
2q21.3b	1615	DARS	Shore	-968
9q22.31b	203328	SUSD3	FALSE	189
1p36.11d	57185	NPAL3	FALSE	-22
10q21.3e	219738	C10orf35	Shore	-90
9q22.31b	203328	SUSD3	FALSE	-1239
17p11.2e	10750	GRAP	FALSE	17
9q34.11a	399665	FAM102A	FALSE	233
1p36.11d	57185	NPAL3	Shore	-1226
6p21.31f	3710	ITPR3	FALSE	-426
4q12e	10606	PAICS	Shore	28
7q36.1b	155066	ATP6V0E2	FALSE	1
1p36.11d	57185	NPAL3	Shore	-1140
14q11.2b	328	APEX1	FALSE	216
7q36.1b	155066	ATP6V0E2	FALSE	877
1p13.1b	914	CD2	FALSE	-196
1p36.11d	57185	NPAL3	TRUE	-9
6p12.2a	4172	MCM3	FALSE	-1274
14q32.12a	8111	GPR68	TRUE	272
8q13.3c	9242	MSC	TRUE	-357
7q36.1b	155066	ATP6V0E2	Shore	-4
7q36.1b	155066	ATP6V0E2	FALSE	-566
9q34.11a	399665	FAM102A	FALSE	-3
1p36.11d	57185	NPAL3	FALSE	-313
10q22.1a	219743	TYSND1	Shore	-509
19q13.31a	5329	PLAUR	FALSE	10
14q32.13a	57062	DDX24	FALSE	-883
1p36.11d	57185	NPAL3	Shore	-509
6p21.31f	3710	ITPR3	Shore	-4

6p12.2a	4172	MCM3	FALSE	125
2q21.3b	1615	DARS	FALSE	216
48589546-48	132299	OCIAD2	Shore	-10
Xp22.11a	5165	PDK3	Shore	222
7q36.1b	155066	ATP6V0E2	FALSE	-212
1p36.11d	57185	NPAL3	Shore	-1088
9q22.31b	203328	SUSD3	FALSE	509
6p12.2a	4172	MCM3	FALSE	570
4q12e	10606	PAICS	FALSE	230
1p13.1b	914	CD2	Shore	-1200
20p13b	994	CDC25B	FALSE	-196
17p11.2e	10750	GRAP	FALSE	-196
9q34.11a	399665	FAM102A	FALSE	189
7q36.1b	155066	ATP6V0E2	FALSE	-716
10q24.1b	64210	MMS19L	Shore	-43
7q36.1b	155066	ATP6V0E2	FALSE	-106
7q36.1b	155066	ATP6V0E2	Shore	49
9q22.31b	203328	SUSD3	FALSE	-428
1p13.1b	914	CD2	Shore	-575
6p12.2a	4172	MCM3	FALSE	226
1p36.11d	57185	NPAL3	Shore	-312
7q36.1b	155066	ATP6V0E2	Shore	-10
15q15.1b	27079	RPUSD2	Shore	15
7q36.1b	155066	ATP6V0E2	FALSE	298
6p21.31f	3710	ITPR3	FALSE	-212
7q36.1b	155066	ATP6V0E2	FALSE	137
15q15.1b	27079	RPUSD2	FALSE	-272
7q36.1b	155066	ATP6V0E2	Shore	69
11q12.2a	923	CD6	FALSE	114
9q22.31b	203328	SUSD3	FALSE	-1282
1p36.11d	57185	NPAL3	FALSE	-193
7q36.1b	155066	ATP6V0E2	FALSE	-108
1p36.11d	57185	NPAL3	FALSE	-441
3p14.2d-p14.	2272	FHIT	FALSE	-1282
7q36.1b	155066	ATP6V0E2	FALSE	60
1p36.11d	57185	NPAL3	FALSE	-147
6p21.31f	3710	ITPR3	FALSE	60
20p13b	994	CDC25B	FALSE	-283
7q36.1b	155066	ATP6V0E2	FALSE	-655
7q36.1b	155066	ATP6V0E2	Shore	-781
6p21.31f	3710	ITPR3	FALSE	-665
6p12.2a	4172	MCM3	Shore	217
8q24.13c	114907	FBXO32	TRUE	170
4q12e	10606	PAICS	FALSE	256

6p12.2a	4172	MCM3	FALSE	-22
6p12.2a	4172	MCM3	FALSE	93
7q36.1b	155066	ATP6V0E2	FALSE	-192
6p12.2a	4172	MCM3	FALSE	312
7q36.1b	155066	ATP6V0E2	Shore	-1267
7q36.1b	155066	ATP6V0E2	FALSE	233
16q22.1f	16	AARS	FALSE	-192
3p21.31d	11180	WDR6	FALSE	-192
6p21.31f	3710	ITPR3	FALSE	681
10q22.1e	9806	SPOCK2	Shore	-1440
1p36.11d	57185	NPAL3	FALSE	873
3p14.2d-p14.	2272	FHIT	FALSE	-341
10q21.3e	219738	C10orf35	FALSE	-875
9q22.31b	203328	SUSD3	Shore	-655
7q36.1b	155066	ATP6V0E2	FALSE	374
7q36.1b	155066	ATP6V0E2	FALSE	681
48589546-48	132299	OCIAD2	FALSE	-710
1p36.11d	57185	NPAL3	FALSE	256
48589546-48	132299	OCIAD2	FALSE	-566
4q12e	10606	PAICS	FALSE	256
6p21.31f	3710	ITPR3	FALSE	-108
9q34.11a	399665	FAM102A	FALSE	-428
1p13.1b	914	CD2	Shore	-389
16q22.1f	16	AARS	FALSE	-313
6p12.2a	4172	MCM3	Shore	-968
1p13.1b	914	CD2	Shore	-13
7q36.1b	155066	ATP6V0E2	FALSE	-710
5q31.1e	23338	PHF15	FALSE	-883
17p11.2e	10750	GRAP	FALSE	-283
6p21.31f	3710	ITPR3	FALSE	137
9q34.11a	399665	FAM102A	FALSE	-192
1p36.11d	57185	NPAL3	FALSE	-694
6p21.31f	3710	ITPR3	Shore	49
6p12.2a	4172	MCM3	Shore	-312
7q36.1b	155066	ATP6V0E2	FALSE	-330
4q12e	10606	PAICS	Shore	15
6p12.2a	4172	MCM3	TRUE	-945
7q36.1b	155066	ATP6V0E2	FALSE	-1239
9q22.31b	203328	SUSD3	FALSE	573
7q36.1b	155066	ATP6V0E2	FALSE	-31
1p36.11d	57185	NPAL3	Shore	28
1p36.12a	3399	ID3	FALSE	509
9q22.31b	203328	SUSD3	Shore	-208
6p21.31f	3710	ITPR3	Shore	69

1p13.1b	914	CD2	Shore	-1440
4q31.21b-q3:	8821	INPP4B	FALSE	-108
3p14.2d-p14.	2272	FHIT	Shore	-208
1p36.11d	57185	NPAL3	FALSE	-823
48589546-48	132299	OCIAD2	Shore	69
7q36.1b	155066	ATP6V0E2	Shore	-849
6p12.2a	4172	MCM3	Shore	-1088
17q25.1d-q2!	439921	MXRA7	TRUE	-28
7q36.1b	155066	ATP6V0E2	FALSE	-1274
1p36.11d	57185	NPAL3	FALSE	381
17p11.2e	10750	GRAP	Shore	-1440
7q36.1b	155066	ATP6V0E2	FALSE	93
7q36.1b	155066	ATP6V0E2	FALSE	-3
1p13.1b	914	CD2	FALSE	158
7q36.1b	155066	ATP6V0E2	FALSE	-665
1p36.11d	57185	NPAL3	Shore	-494
1p36.11d	57185	NPAL3	FALSE	469
17p11.2e	10750	GRAP	FALSE	-426
6p21.31f	3710	ITPR3	FALSE	-330
43075324-43	389289	C5orf39	FALSE	-192
11q12.2a	923	CD6	Shore	-1200
6p21.31f	3710	ITPR3	FALSE	-39
7q36.1b	155066	ATP6V0E2	Shore	-321
1p36.11d	57185	NPAL3	FALSE	-140
5q31.2b	26249	KLHL3	Shore	-208
7q36.1b	155066	ATP6V0E2	FALSE	-1020
6p21.31f	3710	ITPR3	FALSE	-1020
1p36.11d	57185	NPAL3	FALSE	27
1p36.31a	8718	TNFRSF25	Shore	-594
10q22.1e	9806	SPOCK2	FALSE	60
14q32.12a	8111	GPR68	TRUE	10
1p13.1b	914	CD2	FALSE	369
12q13.11b	91523	FAM113B	FALSE	509
4q31.21b-q3:	8821	INPP4B	Shore	671
1p13.1b	914	CD2	TRUE	-290
14q32.12a	8111	GPR68	Shore	-1227
7q36.1b	155066	ATP6V0E2	FALSE	68
1p36.12a	3399	ID3	FALSE	233
6p21.32b	50854	C6orf48	FALSE	509
6p21.31f	3710	ITPR3	FALSE	-655
7q36.1b	155066	ATP6V0E2	FALSE	43
11q12.2a	923	CD6	FALSE	17
6p21.31f	3710	ITPR3	FALSE	-710
6p21.31f	3710	ITPR3	FALSE	-472

20p13b	994	CDC25B	Shore	-1440
4q12e	10606	PAICS	Shore	-719
20p13b	994	CDC25B	Shore	-702
10q22.1e	9806	SPOCK2	FALSE	681
4q12e	10606	PAICS	FALSE	-108
1p36.11d	57185	NPAL3	FALSE	409
9q34.11a	399665	FAM102A	FALSE	189
12q13.11a	81539	SLC38A1	FALSE	-710
6p21.31f	3710	ITPR3	FALSE	93
14q32.12a	8111	GPR68	TRUE	-917
11q12.2b	921	CD5	Shore	-781
1p13.1b	914	CD2	FALSE	681
10q24.1b	10023	FRAT1	FALSE	2
7q36.1b	155066	ATP6V0E2	FALSE	-1282
1p13.1b	914	CD2	FALSE	-139
1p13.1b	914	CD2	FALSE	-665
1p13.1b	914	CD2	FALSE	84
7q36.1b	155066	ATP6V0E2	FALSE	-472
20p13b	994	CDC25B	TRUE	-223
1p13.1b	914	CD2	Shore	-283
7q36.1b	155066	ATP6V0E2	FALSE	-39
11q12.2a	923	CD6	FALSE	-283
43075324-43	389289	C5orf39	FALSE	-313
48589546-48	132299	OCIAD2	FALSE	-192
7q36.1b	155066	ATP6V0E2	TRUE	-945
48589546-48	132299	OCIAD2	Shore	-849
1p13.1b	914	CD2	Shore	-702
10q22.1e	9806	SPOCK2	Shore	-4
6p21.31f	3710	ITPR3	Shore	564
1p36.11d	57185	NPAL3	FALSE	-875
6p12.2a	4172	MCM3	Shore	-1226
6p12.2a	4172	MCM3	TRUE	104
7q36.1b	155066	ATP6V0E2	Shore	217
6p12.2a	4172	MCM3	FALSE	-252
1p36.31a	8718	TNFRSF25	FALSE	509
6p21.32b	50854	C6orf48	FALSE	233
7q36.1b	155066	ATP6V0E2	FALSE	-152
1p13.1b	914	CD2	Shore	-4
7q36.1b	155066	ATP6V0E2	FALSE	189
20p13b	994	CDC25B	FALSE	60
22q12.2c	113791	PIK3IP1	Shore	-594
17p11.2e	10750	GRAP	Shore	-594
20p13b	994	CDC25B	Shore	-4
17p11.2e	10750	GRAP	Shore	-283

1p36.11d	57185	NPAL3	FALSE	-272
11q12.2a	923	CD6	FALSE	-196
7q36.1b	155066	ATP6V0E2	FALSE	2499438
6p21.31f	3710	ITPR3	FALSE	43
9q33.3b	89853	FAM125B	TRUE	-168
6p21.31f	3710	ITPR3	Shore	-321
3p14.2d-p14.	2272	FHIT	FALSE	256
7q36.1b	155066	ATP6V0E2	FALSE	-665
1p36.11d	57185	NPAL3	Shore	-448
17p11.2e	10750	GRAP	FALSE	-212
7q36.1b	155066	ATP6V0E2	FALSE	162
20p13b	994	CDC25B	FALSE	681
1p36.11d	57185	NPAL3	Shore	-43
4q31.1b-q31.	1998	ELF2	FALSE	-784
Xp22.11a	5165	PDK3	FALSE	346
17p11.2e	10750	GRAP	FALSE	60
11q12.2b	921	CD5	FALSE	-655
17p11.2e	10750	GRAP	FALSE	681
12q13.11b	91523	FAM113B	FALSE	233
7q36.1b	155066	ATP6V0E2	FALSE	66
1p36.11d	57185	NPAL3	Shore	-376
1p13.1b	914	CD2	TRUE	-223
9q22.31b	203328	SUSD3	FALSE	-540
7q36.1b	155066	ATP6V0E2	FALSE	36
14q32.12a	8111	GPR68	TRUE	-390
12q23.3c	10970	CKAP4	FALSE	-784
11q12.2a	923	CD6	Shore	-575
6p21.1g	54210	TREM1	FALSE	11
17p11.2e	10750	GRAP	FALSE	877
12q13.11b	91523	FAM113B	FALSE	-39
1p36.11d	57185	NPAL3	FALSE	222
7q36.1b	155066	ATP6V0E2	FALSE	-544
14q32.12a	8111	GPR68	TRUE	77
6p21.31f	3710	ITPR3	FALSE	66
12q13.11b	91523	FAM113B	FALSE	-710
7q36.1b	155066	ATP6V0E2	FALSE	-883
4q12e	10606	PAICS	FALSE	441
17p11.2e	10750	GRAP	FALSE	298
9q34.11a	399665	FAM102A	FALSE	-823
1p13.1b	914	CD2	Shore	49
6p21.31f	3710	ITPR3	FALSE	-883
7q36.1b	155066	ATP6V0E2	Shore	24
6p21.31f	3710	ITPR3	FALSE	-31
1p13.1b	914	CD2	FALSE	-39

9q34.11a	399665	FAM102A	FALSE	-140
6p21.31f	3710	ITPR3	Shore	-492
17p11.2e	10750	GRAP	Shore	-781
20p13b	994	CDC25B	FALSE	-665
11q12.2a	923	CD6	FALSE	-426
17p11.2e	10750	GRAP	Shore	-184
1p13.1b	914	CD2	FALSE	-31
1p13.1b	914	CD2	Shore	-781
11q12.2b	921	CD5	FALSE	1
7q36.1b	155066	ATP6V0E2	TRUE	-9
14q32.12a	8111	GPR68	TRUE	370
6p21.31f	3710	ITPR3	TRUE	-945
14q32.12a	8111	GPR68	FALSE	89
19q13.32b	10055	SAE1	Shore	-313
11q12.2a	923	CD6	FALSE	-1429
10q22.1e	9806	SPOCK2	FALSE	298
17p11.2e	10750	GRAP	FALSE	-665
1p13.1b	914	CD2	Shore	-10
6p21.31f	3710	ITPR3	Shore	-180
1p13.1b	914	CD2	FALSE	95
17p11.2e	10750	GRAP	FALSE	-330
11q12.2b	921	CD5	FALSE	-710
1p36.11d	57185	NPAL3	FALSE	82
13q14.11b	28984	C13orf15	FALSE	509
22q12.2c	113791	PIK3IP1	Shore	-184
17p11.2e	10750	GRAP	FALSE	-31
7q36.1b	155066	ATP6V0E2	FALSE	-1227
6p21.31f	3710	ITPR3	Shore	-294
9q22.31b	203328	SUSD3	Shore	28
14q32.12a	8111	GPR68	TRUE	-905
9q22.31b	203328	SUSD3	FALSE	256
44216240-44	533	ATP6V0B	FALSE	-32
17p11.2e	10750	GRAP	Shore	69
1p36.11d	57185	NPAL3	Shore	599
17p11.2e	10750	GRAP	FALSE	-655
48589546-48	132299	OCIAD2	FALSE	-425
9q34.11a	399665	FAM102A	FALSE	230
3p14.2d-p14.	2272	FHIT	Shore	-321
1p36.11d	57185	NPAL3	Shore	15
11q12.2a	923	CD6	Shore	-283
7q36.1b	155066	ATP6V0E2	Shore	-1088
7q36.1b	155066	ATP6V0E2	Shore	-180
12q23.3c	10970	CKAP4	FALSE	2
1p13.1b	914	CD2	FALSE	-655

20p13b	994	CDC25B	FALSE	-330
1p13.1b	914	CD2	FALSE	-330
7q36.1b	155066	ATP6V0E2	FALSE	-303
20p13b	994	CDC25B	FALSE	-31
10q22.1e	9806	SPOCK2	FALSE	-39
12q13.11b	91523	FAM113B	FALSE	-3
17p11.2e	10750	GRAP	FALSE	509
11q12.2a	923	CD6	FALSE	84
16p13.3d	9235	IL32	Shore	-202
7q36.1b	155066	ATP6V0E2	FALSE	-425
7q36.1b	155066	ATP6V0E2	FALSE	28
10q22.1e	9806	SPOCK2	FALSE	-710
11q12.2a	923	CD6	Shore	-1440
20p13b	994	CDC25B	FALSE	137
20p13b	994	CDC25B	FALSE	-39
6p21.31f	3710	ITPR3	FALSE	-313
6p21.31f	3710	ITPR3	FALSE	-303
16p13.3d	9235	IL32	Shore	-322
11q12.2b	921	CD5	FALSE	-883
7q36.1b	155066	ATP6V0E2	FALSE	-192
7q36.1b	155066	ATP6V0E2	FALSE	-313
7q36.1b	155066	ATP6V0E2	FALSE	-806
11q12.2a	923	CD6	Shore	-594
11q12.2b	921	CD5	Shore	-321
4q31.1b-q31.	1998	ELF2	FALSE	78
7q36.1b	155066	ATP6V0E2	Shore	-592
17p11.2e	10750	GRAP	FALSE	-106
1p13.1b	914	CD2	FALSE	1
7q36.1b	155066	ATP6V0E2	FALSE	-823
11q12.2b	921	CD5	FALSE	-472
17p11.2e	10750	GRAP	FALSE	233
2p23.1b	81606	LBH	FALSE	-710
7q36.1b	155066	ATP6V0E2	FALSE	-22
1p13.1b	914	CD2	FALSE	157
7q36.1b	155066	ATP6V0E2	FALSE	-31
1p13.1b	914	CD2	FALSE	-152
17p11.2e	10750	GRAP	FALSE	-710
11q12.2a	923	CD6	Shore	-702
6p12.2a	4172	MCM3	Shore	2510986
19q13.31a	5329	PLAUR	Shore	-183
1p13.1b	914	CD2	Shore	69
7q36.1b	155066	ATP6V0E2	FALSE	216
17p11.2e	10750	GRAP	FALSE	137
20p13b	994	CDC25B	FALSE	93

7q36.1b	155066	ATP6V0E2	FALSE	-130
6p21.31f	3710	ITPR3	FALSE	-544
7q36.1b	155066	ATP6V0E2	Shore	-968
20p13b	994	CDC25B	FALSE	64
9q33.3b	89853	FAM125B	TRUE	77
6p21.31f	3710	ITPR3	FALSE	-1227
16p13.3d	9235	IL32	Shore	-194
20p13b	994	CDC25B	Shore	69
7q36.1b	155066	ATP6V0E2	Shore	-312
20p13b	994	CDC25B	FALSE	1
22q12.2c	113791	PIK3IP1	FALSE	-39
1p13.1b	914	CD2	FALSE	-716
9q22.31b	203328	SUSD3	FALSE	230
16q22.1f	16	AARS	FALSE	-875
10q22.1a	219743	TYSND1	Shore	-1410
12q13.11b	91523	FAM113B	FALSE	-192
12q13.11b	91523	FAM113B	FALSE	189
16p13.3d	9235	IL32	TRUE	193
11q12.2a	923	CD6	TRUE	-223
1p13.1b	914	CD2	Shore	-283
12q13.11a	81539	SLC38A1	FALSE	-162
117715087-1	915	CD3D	Shore	-702
9q34.11a	399665	FAM102A	FALSE	873
7q36.1b	155066	ATP6V0E2	FALSE	573
2p13.2b	26056	RAB11FIP5	TRUE	-72
10q22.1e	9806	SPOCK2	FALSE	-883
1p13.1b	914	CD2	TRUE	497
Xp22.11a	5165	PDK3	Shore	961
20p13b	994	CDC25B	Shore	49
6p21.31f	3710	ITPR3	FALSE	174
11q12.2a	923	CD6	Shore	-10
5q32d-q32e	5521	PPP2R2B	Shore	355
12q13.11b	91523	FAM113B	Shore	-655
1p13.1b	914	CD2	Shore	11605305
17p11.2e	10750	GRAP	FALSE	93
6p21.31f	3710	ITPR3	Shore	-968
1p13.1b	914	CD2	TRUE	61
7q36.1b	155066	ATP6V0E2	Shore	-97
3p22.2a	4615	MYD88	Shore	-197
1p34.1e-p34.	55182	RNF220	Shore	-876
17p11.2e	10750	GRAP	FALSE	189
19q13.31a	5329	PLAUR	FALSE	2
1p13.1b	914	CD2	FALSE	-302
4q12e	10606	PAICS	FALSE	-263

1q32.1h	9214	FAIM3	FALSE	-1239
17p11.2e	10750	GRAP	Shore	-655
1p13.1b	914	CD2	FALSE	43
22q12.2c	113791	PIK3IP1	FALSE	-472
5p13.2c	3575	IL7R	Shore	-594
5q32d-q32e	5521	PPP2R2B	TRUE	143
11q12.2a	923	CD6	FALSE	-212
6p21.31f	3710	ITPR3	FALSE	216
11q12.2a	923	CD6	FALSE	-139
14q32.12a	8111	GPR68	TRUE	19
17p11.2e	10750	GRAP	FALSE	-472
3p21.31d	11180	WDR6	Shore	-43
1p13.1b	914	CD2	TRUE	-803
10q22.1a	219743	TYSND1	Shore	-389
14q32.12a	8111	GPR68	Shore	-605
1p13.1b	914	CD2	FALSE	-303
17p11.2e	10750	GRAP	FALSE	-3
7q36.1b	155066	ATP6V0E2	FALSE	-428
5q31.2b	26249	KLHL3	FALSE	-108
7q36.1b	155066	ATP6V0E2	FALSE	174
22q12.2c	113791	PIK3IP1	FALSE	-1239
1p13.1b	914	CD2	TRUE	143
1p13.1b	914	CD2	FALSE	275
10q22.1e	9806	SPOCK2	Shore	-321
12q13.11b	91523	FAM113B	FALSE	-1282
11q12.2a	923	CD6	FALSE	681
7q36.1b	155066	ATP6V0E2	FALSE	-193
22q12.2c	113791	PIK3IP1	FALSE	-710
22q12.2c	113791	PIK3IP1	FALSE	509
17p11.2e	10750	GRAP	FALSE	-39
10q22.1e	9806	SPOCK2	FALSE	-303
6p21.31f	3710	ITPR3	FALSE	-22
1p13.1b	914	CD2	FALSE	-710
14q32.12a	8111	GPR68	Shore	-542
21q22.3e	104	ADARB1	Shore	15
9q22.31b	203328	SUSD3	FALSE	-108
1p36.11d	57185	NPAL3	Shore	-1410
6p21.31f	3710	ITPR3	FALSE	-192
1p13.1b	914	CD2	FALSE	-472
20p13b	994	CDC25B	Shore	-451
1p13.1b	914	CD2	TRUE	161
11q12.2a	923	CD6	FALSE	-192
12q13.11a	81539	SLC38A1	FALSE	216
12q13.11b	91523	FAM113B	FALSE	-162

12q13.11b	91523	FAM113B	FALSE	-665
1p13.1b	914	CD2	TRUE	510
14q32.12a	8111	GPR68	TRUE	62
17p11.2e	10750	GRAP	FALSE	-1020
11q12.2a	923	CD6	FALSE	-108
1p13.1b	914	CD2	FALSE	-948
6p12.2a	4172	MCM3	Shore	15
1p13.1b	914	CD2	FALSE	64
22q12.2c	113791	PIK3IP1	FALSE	233
20p13b	994	CDC25B	FALSE	43
14q32.12a	8111	GPR68	Shore	-698
11q12.2b	921	CD5	FALSE	-162
6p21.31f	3710	ITPR3	Shore	-824
6p21.1g	54210	TREM1	FALSE	2
20p13b	994	CDC25B	FALSE	-655
48589546-48	132299	OCIAD2	FALSE	-164
21q22.3e	104	ADARB1	FALSE	-875
6p21.31f	3710	ITPR3	FALSE	-31
1q22c	23208	SYT11	Shore	-645
12q13.11b	91523	FAM113B	FALSE	-152
17p11.2e	10750	GRAP	FALSE	66
20p13b	994	CDC25B	Shore	-321
6p21.1g	54210	TREM1	FALSE	-784
10q22.1e	9806	SPOCK2	FALSE	-192
20p13b	994	CDC25B	FALSE	93
6p21.31f	3710	ITPR3	Shore	-312
6p12.2a	4172	MCM3	FALSE	-272
48589546-48	132299	OCIAD2	FALSE	-147
7q36.1b	155066	ATP6V0E2	FALSE	4
7q36.1b	155066	ATP6V0E2	FALSE	-17
11q12.2a	923	CD6	Shore	49
19q13.33e	2357	FPR1	FALSE	-784
6p21.31f	3710	ITPR3	TRUE	-378
11q12.2a	923	CD6	FALSE	-665
1p13.1b	914	CD2	Shore	23
7q36.1b	155066	ATP6V0E2	FALSE	469
10q22.1e	9806	SPOCK2	TRUE	-945
11q12.2a	923	CD6	FALSE	-1239
20p13b	994	CDC25B	FALSE	-472
11q13.1e	57124	CD248	FALSE	215
17p11.2e	10750	GRAP	FALSE	-303
7q36.1b	155066	ATP6V0E2	Shore	-90
22q12.2c	113791	PIK3IP1	FALSE	189
20p13b	994	CDC25B	Shore	-492

9q22.31b	203328	SUSD3	FALSE	441
11q12.2a	923	CD6	FALSE	877
17p11.2e	10750	GRAP	Shore	-180
1p36.11d	57185	NPAL3	Shore	-606
1p36.12a	3399	ID3	Shore	-245
1p13.1b	914	CD2	TRUE	96
1p13.1b	914	CD2	FALSE	93
16p13.3d	9235	IL32	TRUE	148
20p13b	994	CDC25B	Shore	-180
7q36.1b	155066	ATP6V0E2	Shore	-824
11q12.2a	923	CD6	FALSE	95
7q36.1b	155066	ATP6V0E2	FALSE	256
19q13.31a	5329	PLAUR	Shore	-799
17p11.2e	10750	GRAP	Shore	217
11q12.2a	923	CD6	FALSE	64
2p23.1b	81606	LBH	FALSE	-162
7q36.1b	155066	ATP6V0E2	Shore	-43
1p13.1b	914	CD2	TRUE	-1122
6p21.31f	3710	ITPR3	FALSE	28
6p21.31f	3710	ITPR3	FALSE	-193
12q13.11b	91523	FAM113B	Shore	-208
20p13b	994	CDC25B	FALSE	66
1p13.1b	914	CD2	FALSE	162
11q12.2a	923	CD6	Shore	-322
17p11.2e	10750	GRAP	FALSE	-883
1p36.11c	26119	LDLRAP1	FALSE	189
10q22.1e	9806	SPOCK2	FALSE	-152
20p13b	994	CDC25B	FALSE	-806
6p21.1g	54210	TREM1	FALSE	285
11q12.2a	923	CD6	FALSE	-50
11q12.2a	923	CD6	FALSE	-716
16p13.3d	9235	IL32	TRUE	272
7q36.1b	155066	ATP6V0E2	Shore	28
1p13.1b	914	CD2	TRUE	272
1p13.1b	914	CD2	Shore	596
12q12g	4753	NELL2	FALSE	509
1p13.1b	914	CD2	Shore	-180
5q32d-q32e	5521	PPP2R2B	TRUE	36
1p36.31a	8718	TNFRSF25	FALSE	189
7q36.1b	155066	ATP6V0E2	Shore	-509
20p13b	994	CDC25B	FALSE	-37
1p13.1b	914	CD2	TRUE	228
7q36.1b	155066	ATP6V0E2	Shore	15
12q13.11a	81539	SLC38A1	FALSE	36

7q36.1b	155066	ATP6V0E2	FALSE	381
11q12.2a	923	CD6	FALSE	-330
10q22.1e	9806	SPOCK2	Shore	-180
1p13.1b	914	CD2	Shore	217
117715087-1	915	CD3D	FALSE	1
6p21.31f	3710	ITPR3	FALSE	-164
5q31.2b	26249	KLHL3	FALSE	441
11q12.2b	921	CD5	Shore	-968
11q12.2a	923	CD6	Shore	69
7q36.1b	155066	ATP6V0E2	FALSE	-875
1p13.1b	914	CD2	TRUE	-945
1p13.1b	914	CD2	Shore	-1168
11q12.2b	921	CD5	TRUE	-9
11q12.2a	923	CD6	FALSE	438
1p13.1b	914	CD2	FALSE	-162
20p13b	994	CDC25B	FALSE	-883
11q12.2a	923	CD6	FALSE	1
11q12.2b	921	CD5	FALSE	-152
11q12.2b	921	CD5	FALSE	-192
5p15.2c	83853	ROPN1L	FALSE	-784
11q12.2a	923	CD6	Shore	-1267
11q12.2a	923	CD6	FALSE	60
1p13.1b	914	CD2	TRUE	193
20p13b	994	CDC25B	FALSE	-303
11q12.2a	923	CD6	FALSE	298
17p11.2e	10750	GRAP	FALSE	-806
20p13b	994	CDC25B	Shore	-502
11q12.2a	923	CD6	FALSE	365
1p13.1b	914	CD2	FALSE	-1274
1p13.1b	914	CD2	FALSE	68
11q12.2a	923	CD6	FALSE	-37
12q13.11b	91523	FAM113B	FALSE	230
5q32d-q32e	5521	PPP2R2B	TRUE	272
17p11.2e	10750	GRAP	FALSE	-152
11q12.2a	923	CD6	Shore	-4
11q12.2a	923	CD6	FALSE	-302
14q32.12a	8111	GPR68	TRUE	577
1p13.1b	914	CD2	Shore	-321
1p13.1b	914	CD2	Shore	-294
1p13.1b	914	CD2	TRUE	2
17p11.2e	10750	GRAP	FALSE	-313
1p13.1b	914	CD2	TRUE	10
20p13b	994	CDC25B	FALSE	-544
117715087-1	915	CD3D	Shore	69

11q12.2a	923	CD6	FALSE	137
1p13.1b	914	CD2	FALSE	28
117715087-1	915	CD3D	FALSE	-710
1p13.1b	914	CD2	FALSE	-37
11q12.2a	923	CD6	FALSE	-948
5q32d-q32e	5521	PPP2R2B	TRUE	96
1p13.1b	914	CD2	Shore	24
1p13.1b	914	CD2	FALSE	283
17p11.2e	10750	GRAP	FALSE	-425
22q12.2c	113791	PIK3IP1	FALSE	-162
7q36.1b	155066	ATP6V0E2	FALSE	-164
17p11.2e	10750	GRAP	FALSE	-1282
7q36.1b	155066	ATP6V0E2	FALSE	-111
1p13.1b	914	CD2	Shore	-451
20p13b	994	CDC25B	Shore	564
1p13.1b	914	CD2	TRUE	177
22q12.2c	113791	PIK3IP1	Shore	217
17p11.2e	10750	GRAP	FALSE	2499438
11q14.1a	9846	GAB2	FALSE	7
6p21.31f	3710	ITPR3	Shore	-537
17p11.2e	10750	GRAP	FALSE	-1239
14q32.12a	8111	GPR68	Shore	-1262
1p13.1b	914	CD2	Shore	323
11q12.2a	923	CD6	FALSE	-39
1p13.1b	914	CD2	TRUE	293
1p13.1b	914	CD2	Shore	-352
1p13.1b	914	CD2	FALSE	-822
1p13.1b	914	CD2	Shore	-202
2q37.3g	285093	FLJ33590	Shore	-698
6p21.31f	3710	ITPR3	FALSE	-147
1p13.1b	914	CD2	Shore	-322
1p13.1b	914	CD2	Shore	953
1p13.1b	914	CD2	Shore	-712
1p13.1b	914	CD2	TRUE	-64
22q12.2c	113791	PIK3IP1	Shore	-655
11q12.2a	923	CD6	FALSE	-1020
19q13.31a	5329	PLAUR	FALSE	78
14q32.12a	8111	GPR68	TRUE	72
11q12.2a	923	CD6	FALSE	93
20p13b	994	CDC25B	FALSE	-1245
1p13.1b	914	CD2	FALSE	-98
1p13.1b	914	CD2	Shore	564
20p13b	994	CDC25B	FALSE	-425
22q12.2c	113791	PIK3IP1	FALSE	573

17p11.2e	10750	GRAP	FALSE	216
9q34.11a	399665	FAM102A	FALSE	-384
17p11.2e	10750	GRAP	Shore	71
12q13.11b	91523	FAM113B	FALSE	-225
22q12.2c	113791	PIK3IP1	FALSE	-192
1p13.1b	914	CD2	Shore	-492
17q21.2a	1236	CCR7	Shore	-594
14q32.2b	64919	BCL11B	FALSE	-710
7q36.1b	155066	ATP6V0E2	FALSE	-694
20p13b	994	CDC25B	FALSE	-313
22q12.2c	113791	PIK3IP1	FALSE	16
17p11.2e	10750	GRAP	FALSE	-22
11q12.2a	923	CD6	FALSE	-1274
11q12.2a	923	CD6	FALSE	-710
12q13.11b	91523	FAM113B	Shore	-245
1p13.1b	914	CD2	FALSE	724
7q36.1b	155066	ATP6V0E2	FALSE	-147
1p13.1b	914	CD2	TRUE	33
7q36.1b	155066	ATP6V0E2	Shore	-448
1p36.31a	8718	TNFRSF25	FALSE	573
1p13.1b	914	CD2	FALSE	-31
1p13.1b	914	CD2	TRUE	-661
1p13.1b	914	CD2	Shore	-15
12q13.11b	91523	FAM113B	FALSE	45
12q13.11b	91523	FAM113B	FALSE	-140
11q12.2a	923	CD6	FALSE	-31
1p13.1b	914	CD2	Shore	-968
1p13.1b	914	CD2	FALSE	-1245
11q12.2a	923	CD6	FALSE	189
5q32d-q32e	5521	PPP2R2B	TRUE	-102
1p13.1b	914	CD2	FALSE	-152
16p13.3d	9235	IL32	TRUE	170
17p11.2e	10750	GRAP	FALSE	-822
4q12e	10606	PAICS	Shore	-263
5p15.2c	83853	ROPN1L	FALSE	78
5q32d-q32e	5521	PPP2R2B	TRUE	10
11q12.2a	923	CD6	FALSE	-655
1p13.1b	914	CD2	Shore	-1088
17p11.2e	10750	GRAP	FALSE	230
11q12.2a	923	CD6	FALSE	275
1p13.1b	914	CD2	TRUE	-41
1p13.1b	914	CD2	TRUE	-237
1p13.1b	914	CD2	Shore	-1226
1p13.1b	914	CD2	FALSE	-1227

11q12.2a	923	CD6	Shore	-492
1p13.1b	914	CD2	Shore	-194
17p11.2e	10750	GRAP	FALSE	-192
12q13.11b	91523	FAM113B	FALSE	873
1p13.1b	914	CD2	Shore	-424
1p13.1b	914	CD2	Shore	578
11q12.2a	923	CD6	FALSE	-428
22q12.2c	113791	PIK3IP1	FALSE	189
16p13.3d	9235	IL32	TRUE	100
17p11.2e	10750	GRAP	Shore	-245
12q13.11b	91523	FAM113B	Shore	187
1p13.1b	914	CD2	FALSE	82
9q22.31b	203328	SUSD3	Shore	-606
17p11.2e	10750	GRAP	FALSE	28
12q13.11b	91523	FAM113B	FALSE	256
20p13b	994	CDC25B	FALSE	-192
1p13.1b	914	CD2	TRUE	-157
11q12.2a	923	CD6	Shore	596
11q12.2a	923	CD6	FALSE	-472
1p13.1b	914	CD2	FALSE	216
11q12.2a	923	CD6	Shore	-576
1p13.1b	914	CD2	TRUE	-318
1p36.11c	26119	LDLRAP1	FALSE	-140
11q12.2a	923	CD6	Shore	217
11q12.2a	923	CD6	Shore	-781
12q13.11b	91523	FAM113B	FALSE	256
12q13.11a	81539	SLC38A1	Shore	15
17p11.2e	10750	GRAP	FALSE	-193
11q12.2a	923	CD6	FALSE	31
1p13.1b	914	CD2	FALSE	290
17p11.2e	10750	GRAP	FALSE	189
20p13b	994	CDC25B	Shore	-968
1p13.1b	914	CD2	FALSE	-441
17p11.2e	10750	GRAP	FALSE	174
20p13b	994	CDC25B	FALSE	28
20p13b	994	CDC25B	FALSE	-1227
48589546-48	132299	OCIAD2	Shore	-494
6p21.31f	3710	ITPR3	Shore	15
7q36.1b	155066	ATP6V0E2	FALSE	-371
1p13.1b	914	CD2	FALSE	-545
20p13b	994	CDC25B	Shore	-1226
1p13.1b	914	CD2	Shore	502
17q12b	6352	CCL5	TRUE	10
17p11.2e	10750	GRAP	FALSE	-428

17p11.2e	10750	GRAP	Shore	-312
7q36.1b	155066	ATP6V0E2	FALSE	222
1q22c	23208	SYT11	TRUE	-691
11q12.2a	923	CD6	FALSE	375
1p13.1b	914	CD2	TRUE	-378
17p11.2e	10750	GRAP	FALSE	-823
1p13.1b	914	CD2	TRUE	-213
1p13.1b	914	CD2	Shore	900
1p13.1b	914	CD2	TRUE	566
11q12.2a	923	CD6	TRUE	-945
1p13.1b	914	CD2	TRUE	405
11q12.2a	923	CD6	FALSE	43
20p13b	994	CDC25B	FALSE	-98
1p13.1b	914	CD2	TRUE	252
17p11.2e	10750	GRAP	FALSE	873
17p11.2e	10750	GRAP	FALSE	-147
6p21.31f	3710	ITPR3	Shore	-43
117715087-1	915	CD3D	FALSE	-192
17p11.2e	10750	GRAP	FALSE	573
1p13.1b	914	CD2	TRUE	-917
12q13.11a	81539	SLC38A1	Shore	329
11q12.2a	923	CD6	FALSE	68
5q32d-q32e	5521	PPP2R2B	TRUE	-917
12q13.11b	91523	FAM113B	Shore	28
1p13.1b	914	CD2	FALSE	570
17p11.2e	10750	GRAP	FALSE	-140
1p13.1b	914	CD2	TRUE	-1
11q12.2a	923	CD6	FALSE	-162
7q36.1b	155066	ATP6V0E2	Shore	-494
1p13.1b	914	CD2	FALSE	-147
20p13b	994	CDC25B	FALSE	216
1p13.1b	914	CD2	Shore	512
16p11.2e	388228	SBK1	Shore	-389
22q12.2c	113791	PIK3IP1	FALSE	-193
1p13.1b	914	CD2	Shore	-433
11q12.2a	923	CD6	Shore	564
1p13.1b	914	CD2	Shore	-636
1p13.1b	914	CD2	TRUE	226
1p36.31a	8718	TNFRSF25	FALSE	230
11q12.2a	923	CD6	Shore	-294
1p13.1b	914	CD2	TRUE	170
6p21.31f	3710	ITPR3	FALSE	-272
1q41d	64757	MOSC1	FALSE	2
17q21.2a	1236	CCR7	FALSE	509

11q12.2a	923	CD6	FALSE	-1245
1p13.1b	914	CD2	TRUE	230
11q12.2a	923	CD6	FALSE	162
11q12.2a	923	CD6	FALSE	66
17p11.2e	10750	GRAP	Shore	-90
22q12.2c	113791	PIK3IP1	FALSE	36
22q12.2c	113791	PIK3IP1	FALSE	-22
1p13.1b	914	CD2	Shore	414
11q12.2a	923	CD6	FALSE	-544
1p13.1b	914	CD2	TRUE	108
11q12.2a	923	CD6	FALSE	-806
1p13.1b	914	CD2	Shore	-302
17q12b	6352	CCL5	TRUE	465
1p13.1b	914	CD2	Shore	146907180
22q12.2c	113791	PIK3IP1	FALSE	230
11q12.2a	923	CD6	FALSE	-303
1p13.1b	914	CD2	Shore	-537
17p11.2e	10750	GRAP	FALSE	381
17p11.2e	10750	GRAP	Shore	-509
7q36.1b	155066	ATP6V0E2	Shore	599
1p13.1b	914	CD2	TRUE	-54
10q22.1e	9806	SPOCK2	FALSE	-252
6p21.1g	54210	TREM1	Shore	718
5p15.2c	83853	ROPN1L	Shore	222
7q36.1b	155066	ATP6V0E2	Shore	-207
20p13b	994	CDC25B	Shore	-914
5q32d-q32e	5521	PPP2R2B	TRUE	226
51871607-51	3695	ITGB7	Shore	-43
11q12.2a	923	CD6	Shore	-352
17p11.2e	10750	GRAP	FALSE	-261
11q12.2a	923	CD6	TRUE	-9
17p11.2e	10750	GRAP	FALSE	-17
17p11.2e	10750	GRAP	FALSE	-1049
1p13.1b	914	CD2	FALSE	-322
11q12.2a	923	CD6	FALSE	-110
17p11.2e	10750	GRAP	FALSE	242
5q32d-q32e	5521	PPP2R2B	TRUE	-1
1p13.1b	914	CD2	TRUE	616
1p13.1b	914	CD2	TRUE	263
11q12.2a	923	CD6	FALSE	-822
11q12.2a	923	CD6	Shore	-321
1p13.1b	914	CD2	Shore	-1321
1p13.1b	914	CD2	TRUE	-228
5q32d-q32e	5521	PPP2R2B	TRUE	-184

1p13.1b	914	CD2	Shore	-389
11q12.2a	923	CD6	FALSE	174
1p13.1b	914	CD2	FALSE	-145
1p13.1b	914	CD2	Shore	-43
1p13.1b	914	CD2	Shore	-376
20p13b	994	CDC25B	Shore	54
1p13.1b	914	CD2	TRUE	-363
1p13.1b	914	CD2	FALSE	226
22q12.2c	113791	PIK3IP1	Shore	-90
5q32d-q32e	5521	PPP2R2B	TRUE	465
11q12.2a	923	CD6	FALSE	-313
11q12.2a	923	CD6	FALSE	-22
11q12.2a	923	CD6	FALSE	-883
1p13.1b	914	CD2	TRUE	369
1p13.1b	914	CD2	TRUE	594
5q32d-q32e	5521	PPP2R2B	TRUE	169
11q12.2a	923	CD6	FALSE	-152
1p13.1b	914	CD2	Shore	-147
117715087-1	915	CD3D	TRUE	-9
11q12.2a	923	CD6	FALSE	-665
5q32d-q32e	5521	PPP2R2B	TRUE	-691
11q12.2a	923	CD6	TRUE	-378
12q24.33c	8408	ULK1	Shore	-280
1p13.1b	914	CD2	TRUE	447
17p11.2e	10750	GRAP	Shore	28
11q12.2a	923	CD6	Shore	-1088
1p13.1b	914	CD2	TRUE	100
6p21.31f	3710	ITPR3	Shore	599
11q12.2a	923	CD6	FALSE	216
17q12b	6352	CCL5	TRUE	370
11q12.2a	923	CD6	FALSE	-441
11q12.2a	923	CD6	Shore	-592
5q32d-q32e	5521	PPP2R2B	TRUE	-283
11q12.2b	921	CD5	FALSE	-272
17q21.2a	1236	CCR7	FALSE	233
22q12.2c	113791	PIK3IP1	Shore	28
1p13.1b	914	CD2	TRUE	-217
5q32d-q32e	5521	PPP2R2B	TRUE	455
1p13.1b	914	CD2	Shore	-494
1p13.1b	914	CD2	Shore	-594
20p13b	994	CDC25B	Shore	-636
1p13.1b	914	CD2	TRUE	148
1p13.1b	914	CD2	Shore	-61
5q32d-q32e	5521	PPP2R2B	TRUE	168

1p13.1b	914	CD2	TRUE	-72
11q12.2a	923	CD6	FALSE	-193
11q12.2a	923	CD6	FALSE	-425
22q12.2c	113791	PIK3IP1	FALSE	-140
17q21.2a	1236	CCR7	FALSE	-3
6p21.31f	3710	ITPR3	Shore	-1410
1p13.1b	914	CD2	Shore	-1468
11q12.2a	923	CD6	FALSE	-1227
17q12b	6352	CCL5	TRUE	8
11q12.2a	923	CD6	Shore	-180
1p13.1b	914	CD2	TRUE	93
22q12.2c	113791	PIK3IP1	FALSE	256
1p13.1b	914	CD2	Shore	2510986
22q12.2c	113791	PIK3IP1	FALSE	469
1p13.1b	914	CD2	TRUE	82
1p36.11d	57185	NPAL3	Shore	-263
5q32d-q32e	5521	PPP2R2B	TRUE	-905
1p13.1b	914	CD2	Shore	-570
1p13.1b	914	CD2	Shore	-670
11q12.2a	923	CD6	Shore	-968
17p11.2e	10750	GRAP	FALSE	-875
17q25.3g	924	CD7	FALSE	-17
1p13.1b	914	CD2	TRUE	-619
11q12.2a	923	CD6	Shore	-636
17q12b	6352	CCL5	TRUE	708
11q12.2a	923	CD6	FALSE	285
1p13.1b	914	CD2	FALSE	5
5q32d-q32e	5521	PPP2R2B	TRUE	347
17p11.2e	10750	GRAP	FALSE	469
14q32.2b	64919	BCL11B	Shore	28
12q13.11b	91523	FAM113B	FALSE	441
17p11.2e	10750	GRAP	FALSE	-694
1p13.1b	914	CD2	Shore	-267
20p13b	994	CDC25B	Shore	15
7q36.1b	155066	ATP6V0E2	Shore	-1410
22q12.2c	113791	PIK3IP1	FALSE	-875
5q32d-q32e	5521	PPP2R2B	TRUE	197
1p13.1b	914	CD2	TRUE	650
1p13.1b	914	CD2	TRUE	272
16p13.3d	9235	IL32	Shore	315
17p11.2e	10750	GRAP	FALSE	256
1p13.1b	914	CD2	TRUE	207
1p13.1b	914	CD2	TRUE	-687
20p13b	994	CDC25B	Shore	-494

17q12b	6352	CCL5	TRUE	62
1p13.1b	914	CD2	TRUE	-149
11q12.2a	923	CD6	FALSE	28
11q12.2a	923	CD6	FALSE	-192
11q12.2a	923	CD6	Shore	-509
1p13.1b	914	CD2	Shore	664
1p13.1b	914	CD2	Shore	-1085
9q34.11a	399665	FAM102A	Shore	-263
11q12.2a	923	CD6	FALSE	-164
12q12g	4753	NELL2	FALSE	-162
1p13.1b	914	CD2	TRUE	-72
5q32d-q32e	5521	PPP2R2B	TRUE	-571
2p11.2e	925	CD8A	TRUE	143
1p13.1b	914	CD2	Shore	347
17q21.2a	1236	CCR7	Shore	-655
5q32d-q32e	5521	PPP2R2B	TRUE	-913
1p13.1b	914	CD2	Shore	-237
17q21.2a	1236	CCR7	FALSE	-1239
5q32d-q32e	5521	PPP2R2B	TRUE	-258
1p13.1b	914	CD2	Shore	-542
17q21.2a	1236	CCR7	FALSE	-192
11q12.2a	923	CD6	FALSE	381
17q12b	6352	CCL5	TRUE	-390
5q32d-q32e	5521	PPP2R2B	TRUE	467
1p13.1b	914	CD2	TRUE	-113
1p13.1b	914	CD2	FALSE	-36
1p13.1b	914	CD2	Shore	54
17p11.2e	10750	GRAP	FALSE	222
1p13.1b	914	CD2	FALSE	-271
22q12.2c	113791	PIK3IP1	FALSE	-108
1p13.1b	914	CD2	Shore	-1074
16p13.3d	9235	IL32	Shore	-739
22q12.2c	113791	PIK3IP1	FALSE	873
1p36.31a	8718	TNFRSF25	FALSE	409
20p13b	994	CDC25B	Shore	-1410
11q12.2a	923	CD6	Shore	-537
1p13.1b	914	CD2	TRUE	315
1p13.1b	914	CD2	TRUE	731
22q12.2c	113791	PIK3IP1	FALSE	409
1p13.1b	914	CD2	FALSE	89
11q12.2a	923	CD6	FALSE	-147
1p22.2a-p22.	7049	TGFBR3	TRUE	272
17q21.2a	1236	CCR7	FALSE	-428
6757671-675	3902	LAG3	TRUE	161

1p13.1b	914	CD2	TRUE	104
5q32d-q32e	5521	PPP2R2B	TRUE	-21995995
17p11.2e	10750	GRAP	FALSE	-371
2p11.2e	925	CD8A	TRUE	293
1p13.1b	914	CD2	Shore	599
47370401-47	5199	CFP	FALSE	-721
11q12.2a	923	CD6	FALSE	-31
1p13.1b	914	CD2	TRUE	402
12q13.11b	91523	FAM113B	Shore	-606
17q21.2a	1236	CCR7	FALSE	189
5q32d-q32e	5521	PPP2R2B	TRUE	62
1p13.1b	914	CD2	Shore	-99
1p13.1b	914	CD2	TRUE	-434
1p36.11d	57185	NPAL3	FALSE	-101
117715087-1	915	CD3D	FALSE	-219
1p13.1b	914	CD2	TRUE	-146
1p13.1b	914	CD2	TRUE	485
17q21.2a	1236	CCR7	FALSE	189
5q32d-q32e	5521	PPP2R2B	TRUE	435
1p13.1b	914	CD2	FALSE	-272
1p13.1b	914	CD2	TRUE	49
9q22.31a	4783	NFIL3	FALSE	-34
1p13.1b	914	CD2	TRUE	640
1p13.1b	914	CD2	Shore	15
16p13.3d	9235	IL32	TRUE	-139
1p13.1b	914	CD2	Shore	758
1p13.1b	914	CD2	TRUE	-1090
22q12.2c	113791	PIK3IP1	Shore	-448
56562208-56	125875	CLDND2	FALSE	-1
16p13.3d	9235	IL32	TRUE	467
2p11.2e	925	CD8A	Shore	-1168
17q21.2a	1236	CCR7	FALSE	-1282
20p13b	994	CDC25B	Shore	-43
1p13.1b	914	CD2	Shore	-567
11q12.2a	923	CD6	Shore	-376
11q12.2a	923	CD6	Shore	-494
11q12.2a	923	CD6	FALSE	-694
1p13.1b	914	CD2	FALSE	168
22q12.2c	113791	PIK3IP1	Shore	-207
17p11.2e	10750	GRAP	FALSE	-373
1p13.1b	914	CD2	TRUE	-67
17q21.2a	1236	CCR7	FALSE	573
2p11.2e	925	CD8A	Shore	-202
5q32d-q32e	5521	PPP2R2B	TRUE	-499

7q36.1b	155066	ATP6V0E2	Shore	-263
11q12.2a	923	CD6	Shore	15
1p13.1b	914	CD2	TRUE	-139
20p13b	994	CDC25B	FALSE	-272
17q12b	6352	CCL5	TRUE	-36
2p11.2e	925	CD8A	TRUE	228
11q12.2a	923	CD6	FALSE	-875
1p13.1b	914	CD2	TRUE	-365
1p22.2a-p22.	7049	TGFBR3	TRUE	465
1p13.1b	914	CD2	TRUE	-206
6p21.32b	50854	C6orf48	FALSE	-132
2p11.2e	925	CD8A	TRUE	272
2p11.2e	925	CD8A	TRUE	-102
1p13.1b	914	CD2	TRUE	-489
22q12.2c	113791	PIK3IP1	FALSE	-373
1p13.1b	914	CD2	FALSE	-215
1p13.1b	914	CD2	Shore	-1228
11q12.2a	923	CD6	FALSE	-271
17p11.2e	10750	GRAP	Shore	-207
9q22.31b	203328	SUSD3	FALSE	-1417
22q12.2c	113791	PIK3IP1	FALSE	441
117715087-1	915	CD3D	Shore	-494
6757671-675	3902	LAG3	TRUE	272
1p13.1b	914	CD2	TRUE	349
2p11.2e	925	CD8A	TRUE	-160
5q32d-q32e	5521	PPP2R2B	TRUE	-72
1p13.1b	914	CD2	Shore	315
1p13.1b	914	CD2	Shore	-242
2p11.2e	925	CD8A	TRUE	100
2p11.2e	925	CD8A	TRUE	96
1p13.1b	914	CD2	Shore	-7
1p13.1b	914	CD2	TRUE	-65
2p11.2e	925	CD8A	Shore	11605305
2p11.2e	925	CD8A	TRUE	-185
2p11.2e	925	CD8A	Shore	-194
17q21.2a	1236	CCR7	Shore	71
11q12.2a	923	CD6	Shore	-43
2p11.2e	925	CD8A	TRUE	-160
2p11.2e	925	CD8A	Shore	355
2p11.2e	925	CD8A	Shore	-202
2p11.2e	925	CD8A	TRUE	-698
14q32.2b	64919	BCL11B	FALSE	441
10q22.1b	5551	PRF1	FALSE	-1
2p11.2e	925	CD8A	TRUE	33

6757671-675	3902	LAG3	TRUE	94
11q14.1a	9846	GAB2	Shore	-197
2p11.2e	925	CD8A	Shore	11605305
17q12b	6352	CCL5	TRUE	-638
2p11.2e	925	CD8A	TRUE	193
11q12.2a	923	CD6	FALSE	-272
1p13.1b	914	CD2	Shore	-497
17p11.2e	10750	GRAP	Shore	-1410
17p11.2e	10750	GRAP	Shore	-389
1p13.1b	914	CD2	TRUE	257
11q12.2a	923	CD6	Shore	-99
2p11.2e	925	CD8A	TRUE	-917
1p13.1b	914	CD2	TRUE	277
17q21.2a	1236	CCR7	FALSE	242
1p13.1b	914	CD2	Shore	-979
17p11.2e	10750	GRAP	FALSE	-243
22q12.2c	113791	PIK3IP1	Shore	-606
17q21.2a	1236	CCR7	Shore	28
2p11.2e	925	CD8A	TRUE	193
17q21.2a	1236	CCR7	Shore	-321
2p11.2e	925	CD8A	TRUE	-1
1p13.1b	914	CD2	TRUE	373
2p11.2e	925	CD8A	TRUE	230
2p11.2e	925	CD8A	TRUE	36
19p13.3e	4145	MATK	FALSE	-1
1p13.1b	914	CD2	TRUE	467
2p11.2e	925	CD8A	TRUE	129
2p11.2e	925	CD8A	TRUE	219
17q21.2a	1236	CCR7	FALSE	-140
2p11.2e	925	CD8A	TRUE	94
16p13.3d	9235	IL32	TRUE	373
1p13.1b	914	CD2	Shore	-305
12q13.11b	91523	FAM113B	Shore	155
11q12.2a	923	CD6	Shore	599
48589546-48	132299	OCIAD2	FALSE	-1417
1p13.1b	914	CD2	Shore	-191
1p13.1b	914	CD2	FALSE	322
2p11.2e	925	CD8A	TRUE	-144
17q21.2a	1236	CCR7	FALSE	-17
1p13.1b	914	CD2	Shore	-450
5q32d-q32e	5521	PPP2R2B	TRUE	274
2p11.2e	925	CD8A	TRUE	8
12q13.11b	91523	FAM113B	Shore	-263
1p22.2a-p22.	7049	TGFBR3	FALSE	89

1p13.1b	914	CD2	Shore	-975
2p11.2e	925	CD8A	TRUE	555
2p11.2e	925	CD8A	TRUE	198
2p11.2e	925	CD8A	Shore	23
2p11.2e	925	CD8A	TRUE	230
2p11.2e	925	CD8A	TRUE	94
2p11.2e	925	CD8A	TRUE	-905
19q13.33d	4818	NKG7	FALSE	-1
11q12.2a	923	CD6	Shore	-1410
17q21.2a	1236	CCR7	FALSE	-875
2p11.2e	925	CD8A	TRUE	-54
2p11.2e	925	CD8A	TRUE	110
2p11.2e	925	CD8A	TRUE	455
2p11.2e	925	CD8A	TRUE	36
2p11.2e	925	CD8A	TRUE	77
2p11.2e	925	CD8A	TRUE	114
2p11.2e	925	CD8A	TRUE	70
2p11.2e	925	CD8A	TRUE	-44
1p13.1b	914	CD2	Shore	-440
2p11.2e	925	CD8A	TRUE	-422
11q12.2a	923	CD6	FALSE	-59055398
2p11.2e	925	CD8A	Shore	64
2p11.2e	925	CD8A	TRUE	449
17p11.2e	10750	GRAP	Shore	-263
2p11.2e	925	CD8A	TRUE	34
2p11.2e	925	CD8A	Shore	-21995419
2p11.2e	925	CD8A	TRUE	25
2p11.2e	925	CD8A	TRUE	110
2p11.2e	925	CD8A	TRUE	185
2p11.2e	925	CD8A	TRUE	129
17q21.2a	1236	CCR7	FALSE	873
2p11.2e	925	CD8A	Shore	-424
2p11.2e	925	CD8A	TRUE	99
2p11.2e	925	CD8A	FALSE	128
17q12b	6352	CCL5	Shore	36
2p11.2e	925	CD8A	TRUE	148
2p11.2e	925	CD8A	TRUE	-98
17q21.2a	1236	CCR7	FALSE	27
2p11.2e	925	CD8A	TRUE	-384
1p13.1b	914	CD2	TRUE	274
6757671-675	3902	LAG3	TRUE	-638
2p11.2e	925	CD8A	TRUE	198
1p36.31a	8718	TNFRSF25	Shore	-263
2p11.2e	925	CD8A	FALSE	128

2p11.2e	925	CD8A	Shore	-645
17q21.2a	1236	CCR7	FALSE	-384
2p11.2e	925	CD8A	TRUE	185
2p11.2e	925	CD8A	TRUE	226
2p11.2e	925	CD8A	TRUE	170
17q21.2a	1236	CCR7	Shore	-448
2p11.2e	925	CD8A	TRUE	-98
2p11.2e	925	CD8A	Shore	-1077
2p11.2e	925	CD8A	Shore	64
2p11.2e	925	CD8A	TRUE	143
2p11.2e	925	CD8A	TRUE	-258
2p11.2e	925	CD8A	TRUE	10
2p11.2e	925	CD8A	TRUE	-304
2p11.2e	925	CD8A	Shore	-1227
2p11.2e	925	CD8A	Shore	-21995419
2p11.2e	925	CD8A	TRUE	268
2p11.2e	925	CD8A	TRUE	25
2p11.2e	925	CD8A	TRUE	70
5q32d-q32e	5521	PPP2R2B	Shore	36
2p11.2e	925	CD8A	TRUE	-422
2p11.2e	925	CD8A	TRUE	34
2p11.2e	925	CD8A	TRUE	207
2p11.2e	925	CD8A	TRUE	2
1p22.2a-p22.	7049	TGFBR3	TRUE	-638
2p11.2e	925	CD8A	TRUE	340
2p11.2e	925	CD8A	TRUE	-1131
2p11.2e	925	CD8A	TRUE	84
2p11.2e	925	CD8A	TRUE	0
2p11.2e	925	CD8A	TRUE	99
2p11.2e	925	CD8A	TRUE	269
19p13.2b	53637	S1PR5	FALSE	-1
2p11.2e	925	CD8A	Shore	-267
2p11.2e	925	CD8A	Shore	-1227
2p11.2e	925	CD8A	TRUE	16
2p11.2e	925	CD8A	TRUE	170
2p11.2e	925	CD8A	TRUE	467
2p11.2e	925	CD8A	TRUE	148
2p11.2e	925	CD8A	TRUE	161
2p11.2e	925	CD8A	TRUE	-184
2p11.2e	925	CD8A	TRUE	-41
2p11.2e	925	CD8A	TRUE	177
2p11.2e	925	CD8A	TRUE	563
2p11.2e	925	CD8A	TRUE	101
2p11.2e	925	CD8A	TRUE	78

2p11.2e	925	CD8A	TRUE	657
2p11.2e	925	CD8A	TRUE	-363
2p11.2e	925	CD8A	Shore	-645
2p11.2e	925	CD8A	TRUE	133
22q12.2c	113791	PIK3IP1	Shore	-263
2p11.2e	925	CD8A	TRUE	-185
2p11.2e	925	CD8A	TRUE	177
2p11.2e	925	CD8A	Shore	823
2p11.2e	925	CD8A	TRUE	10
2p11.2e	925	CD8A	TRUE	143
2p11.2e	925	CD8A	TRUE	-698
2p11.2e	925	CD8A	TRUE	402
2p11.2e	925	CD8A	TRUE	48122676
2p11.2e	925	CD8A	Shore	-21995563
2p11.2e	925	CD8A	TRUE	177
2p11.2e	925	CD8A	TRUE	-20
2p11.2e	925	CD8A	TRUE	293
2p11.2e	925	CD8A	TRUE	-1131
2p11.2e	925	CD8A	TRUE	-691
2p11.2e	925	CD8A	TRUE	566
2p11.2e	925	CD8A	TRUE	263
2p11.2e	925	CD8A	TRUE	27
2p11.2e	925	CD8A	TRUE	506
2p11.2e	925	CD8A	TRUE	252
2p11.2e	925	CD8A	Shore	-385
2p11.2e	925	CD8A	Shore	823
2p11.2e	925	CD8A	TRUE	377
2p11.2e	925	CD8A	TRUE	657
2p11.2e	925	CD8A	TRUE	228
2p11.2e	925	CD8A	TRUE	-164
2p11.2e	925	CD8A	TRUE	-39
2p11.2e	925	CD8A	TRUE	-127
2p11.2e	925	CD8A	TRUE	-304
2p11.2e	925	CD8A	TRUE	-2906389
5p13.2c	3575	IL7R	FALSE	55
2p11.2e	925	CD8A	TRUE	-273
2p11.2e	925	CD8A	TRUE	191
2p11.2e	925	CD8A	TRUE	-51
2p11.2e	925	CD8A	TRUE	-110
2p11.2e	925	CD8A	TRUE	9
2p11.2e	925	CD8A	TRUE	332
2p11.2e	925	CD8A	TRUE	-460
2p11.2e	925	CD8A	TRUE	191
2p11.2e	925	CD8A	TRUE	25

2p11.2e	925	CD8A	TRUE	269
2p11.2e	925	CD8A	TRUE	-213
2p11.2e	925	CD8A	TRUE	27
2p11.2e	925	CD8A	TRUE	161
2p11.2e	925	CD8A	TRUE	-521
2p11.2e	925	CD8A	TRUE	139
2p11.2e	925	CD8A	TRUE	133
2p11.2e	925	CD8A	TRUE	47
2p11.2e	925	CD8A	TRUE	-341
2p11.2e	925	CD8A	TRUE	-521
2p11.2e	925	CD8A	TRUE	6
2p11.2e	925	CD8A	TRUE	48122676
2p11.2e	925	CD8A	TRUE	-184
2p11.2e	925	CD8A	TRUE	16
2p11.2e	925	CD8A	TRUE	-348
2p11.2e	925	CD8A	TRUE	74
2p11.2e	925	CD8A	TRUE	-306
2p11.2e	925	CD8A	TRUE	571
2p11.2e	925	CD8A	TRUE	-164
2p11.2e	925	CD8A	TRUE	563
2p11.2e	925	CD8A	TRUE	2
2p11.2e	925	CD8A	Shore	-570
2p11.2e	925	CD8A	TRUE	369
2p11.2e	925	CD8A	TRUE	177
2p11.2e	925	CD8A	TRUE	-41
2p11.2e	925	CD8A	Shore	347
2p11.2e	925	CD8A	TRUE	-171
2p11.2e	925	CD8A	TRUE	234
2p11.2e	925	CD8A	TRUE	405
2p11.2e	925	CD8A	TRUE	-213
2p11.2e	925	CD8A	TRUE	78
2p11.2e	925	CD8A	TRUE	594
2p11.2e	925	CD8A	TRUE	-433
2p11.2e	925	CD8A	TRUE	-44
2p11.2e	925	CD8A	TRUE	-72
2p11.2e	925	CD8A	TRUE	-185
2p11.2e	925	CD8A	TRUE	100
2p11.2e	925	CD8A	TRUE	-407
2p11.2e	925	CD8A	TRUE	25
2p11.2e	925	CD8A	TRUE	56
2p11.2e	925	CD8A	TRUE	252
2p11.2e	925	CD8A	TRUE	-184
2p11.2e	925	CD8A	TRUE	293
6757671-675	3902	LAG3	TRUE	274

2p11.2e	925	CD8A	Shore	-21995563
2p11.2e	925	CD8A	TRUE	-306
17q21.2a	1236	CCR7	Shore	-606
2p11.2e	925	CD8A	TRUE	-102
2p11.2e	925	CD8A	TRUE	92
2p11.2e	925	CD8A	FALSE	-31
2p11.2e	925	CD8A	Shore	323
2p11.2e	925	CD8A	TRUE	228
2p11.2e	925	CD8A	TRUE	263
2p11.2e	925	CD8A	TRUE	465
2p11.2e	925	CD8A	FALSE	-31
2p11.2e	925	CD8A	Shore	13
2p11.2e	925	CD8A	TRUE	-39
2p11.2e	925	CD8A	FALSE	-36
2p11.2e	925	CD8A	TRUE	405
2p11.2e	925	CD8A	TRUE	96
2p11.2e	925	CD8A	TRUE	74
2p11.2e	925	CD8A	TRUE	-273
2p11.2e	925	CD8A	TRUE	370
2p11.2e	925	CD8A	Shore	323
2p11.2e	925	CD8A	Shore	-294
2p11.2e	925	CD8A	TRUE	-149
2p11.2e	925	CD8A	TRUE	-698
2p11.2e	925	CD8A	TRUE	566
2p11.2e	925	CD8A	TRUE	616
2p11.2e	925	CD8A	Shore	144
2p11.2e	925	CD8A	TRUE	-381
2p11.2e	925	CD8A	TRUE	275
2p11.2e	925	CD8A	TRUE	-460
2p11.2e	925	CD8A	TRUE	-691
2p11.2e	925	CD8A	TRUE	369
2p11.2e	925	CD8A	TRUE	139
2p11.2e	925	CD8A	TRUE	9
2p11.2e	925	CD8A	FALSE	-2
2p11.2e	925	CD8A	TRUE	616
2p11.2e	925	CD8A	TRUE	-519
2p11.2e	925	CD8A	TRUE	77
2p11.2e	925	CD8A	FALSE	-36
2p11.2e	925	CD8A	TRUE	203
2p11.2e	925	CD8A	TRUE	-100
2p11.2e	925	CD8A	TRUE	272
2p11.2e	925	CD8A	TRUE	-228
2p11.2e	925	CD8A	FALSE	-2
2p11.2e	925	CD8A	TRUE	151

2p11.2e	925	CD8A	TRUE	33
2p11.2e	925	CD8A	TRUE	-102
2p11.2e	925	CD8A	TRUE	571
2p11.2e	925	CD8A	TRUE	-619
2p11.2e	925	CD8A	Shore	347
2p11.2e	925	CD8A	TRUE	72
2p11.2e	925	CD8A	TRUE	234
2p11.2e	925	CD8A	TRUE	387
2p11.2e	925	CD8A	TRUE	-184
2p11.2e	925	CD8A	TRUE	82
2p11.2e	925	CD8A	TRUE	98
2p11.2e	925	CD8A	TRUE	56
2p11.2e	925	CD8A	TRUE	-72
2p11.2e	925	CD8A	TRUE	572
2p11.2e	925	CD8A	TRUE	77
2p11.2e	925	CD8A	TRUE	-917
2p11.2e	925	CD8A	TRUE	276
2p11.2e	925	CD8A	TRUE	465
2p11.2e	925	CD8A	TRUE	272
2p11.2e	925	CD8A	TRUE	654
2p11.2e	925	CD8A	TRUE	-1090
2p11.2e	925	CD8A	TRUE	-149
2p11.2e	925	CD8A	TRUE	219
2p11.2e	925	CD8A	TRUE	203
2p11.2e	925	CD8A	TRUE	151
2p11.2e	925	CD8A	TRUE	257
2p11.2e	925	CD8A	TRUE	44
2p11.2e	925	CD8A	TRUE	49
2p11.2e	925	CD8A	TRUE	96
2p11.2e	925	CD8A	TRUE	-348
2p11.2e	925	CD8A	FALSE	5
2p11.2e	925	CD8A	TRUE	-619
2p11.2e	925	CD8A	TRUE	709
2p11.2e	925	CD8A	TRUE	654
2p11.2e	925	CD8A	TRUE	94
2p11.2e	925	CD8A	TRUE	-217
2p11.2e	925	CD8A	TRUE	-341
2p11.2e	925	CD8A	TRUE	-21995995
2p11.2e	925	CD8A	Shore	-294
2p11.2e	925	CD8A	TRUE	77
2p11.2e	925	CD8A	TRUE	230
2p11.2e	925	CD8A	TRUE	410
2p11.2e	925	CD8A	TRUE	6
2p11.2e	925	CD8A	TRUE	0

2p11.2e	925	CD8A	TRUE	100
2p11.2e	925	CD8A	TRUE	230
2p11.2e	925	CD8A	TRUE	313
2p11.2e	925	CD8A	TRUE	402
2p11.2e	925	CD8A	TRUE	594
2p11.2e	925	CD8A	TRUE	-359
2p11.2e	925	CD8A	TRUE	312
2p11.2e	925	CD8A	TRUE	-228
2p11.2e	925	CD8A	TRUE	572
2p11.2e	925	CD8A	FALSE	0
2p11.2e	925	CD8A	TRUE	-283
2p11.2e	925	CD8A	Shore	144
2p11.2e	925	CD8A	TRUE	108
2p11.2e	925	CD8A	TRUE	47
2p11.2e	925	CD8A	TRUE	207
2p11.2e	925	CD8A	TRUE	354
2p11.2e	925	CD8A	TRUE	-571
2p11.2e	925	CD8A	TRUE	435
2p11.2e	925	CD8A	TRUE	-110
2p11.2e	925	CD8A	TRUE	272
2p11.2e	925	CD8A	TRUE	-79
2p11.2e	925	CD8A	TRUE	447
2p11.2e	925	CD8A	TRUE	-359
2p11.2e	925	CD8A	TRUE	276
2p11.2e	925	CD8A	TRUE	485
2p11.2e	925	CD8A	TRUE	33
2p11.2e	925	CD8A	TRUE	-217
2p11.2e	925	CD8A	TRUE	449
2p11.2e	925	CD8A	TRUE	650
2p11.2e	925	CD8A	TRUE	-283
2p11.2e	925	CD8A	TRUE	9
2p11.2e	925	CD8A	TRUE	82
2p11.2e	925	CD8A	TRUE	-75
2p11.2e	925	CD8A	TRUE	-419
2p11.2e	925	CD8A	TRUE	93
2p11.2e	925	CD8A	TRUE	209
2p11.2e	925	CD8A	TRUE	230
2p11.2e	925	CD8A	TRUE	-1
2p11.2e	925	CD8A	TRUE	354
2p11.2e	925	CD8A	TRUE	272
2p11.2e	925	CD8A	FALSE	5
2p11.2e	925	CD8A	TRUE	-519
2p11.2e	925	CD8A	TRUE	136
2p11.2e	925	CD8A	TRUE	219

2p11.2e	925	CD8A	TRUE	-36
2p11.2e	925	CD8A	TRUE	-22
2p11.2e	925	CD8A	Shore	13
2p11.2e	925	CD8A	TRUE	47
2p11.2e	925	CD8A	TRUE	182
2p11.2e	925	CD8A	TRUE	370
2p11.2e	925	CD8A	TRUE	15
2p11.2e	925	CD8A	TRUE	-917
2p11.2e	925	CD8A	Shore	-542
2p11.2e	925	CD8A	TRUE	94
2p11.2e	925	CD8A	TRUE	169
2p11.2e	925	CD8A	TRUE	106
2p11.2e	925	CD8A	TRUE	98
2p11.2e	925	CD8A	Shore	-305
2p11.2e	925	CD8A	TRUE	252
2p11.2e	925	CD8A	TRUE	313
2p11.2e	925	CD8A	TRUE	-146
2p11.2e	925	CD8A	TRUE	279
2p11.2e	925	CD8A	TRUE	718
2p11.2e	925	CD8A	TRUE	-571
2p11.2e	925	CD8A	TRUE	387
2p11.2e	925	CD8A	TRUE	257
2p11.2e	925	CD8A	TRUE	-65
2p11.2e	925	CD8A	TRUE	410
2p11.2e	925	CD8A	TRUE	24
2p11.2e	925	CD8A	TRUE	318
2p11.2e	925	CD8A	TRUE	230
2p11.2e	925	CD8A	TRUE	108
2p11.2e	925	CD8A	TRUE	168
2p11.2e	925	CD8A	TRUE	-54
2p11.2e	925	CD8A	TRUE	275
2p11.2e	925	CD8A	TRUE	-79
2p11.2e	925	CD8A	TRUE	-419
2p11.2e	925	CD8A	TRUE	-67
2p11.2e	925	CD8A	Shore	758
2p11.2e	925	CD8A	TRUE	44
2p11.2e	925	CD8A	TRUE	268
2p11.2e	925	CD8A	TRUE	168
2p11.2e	925	CD8A	TRUE	383
2p11.2e	925	CD8A	TRUE	-36
2p11.2e	925	CD8A	TRUE	-299
2p11.2e	925	CD8A	Shore	-605
2p11.2e	925	CD8A	TRUE	515
2p11.2e	925	CD8A	TRUE	-390

2p11.2e	925	CD8A	TRUE	709
2p11.2e	925	CD8A	TRUE	8
2p11.2e	925	CD8A	TRUE	106
2p11.2e	925	CD8A	TRUE	454
2p11.2e	925	CD8A	TRUE	-1090
2p11.2e	925	CD8A	TRUE	485
2p11.2e	925	CD8A	Shore	315
2p11.2e	925	CD8A	TRUE	-146
17q21.2a	1236	CCR7	Shore	-263
2p11.2e	925	CD8A	FALSE	-498
2p11.2e	925	CD8A	TRUE	447
2p11.2e	925	CD8A	TRUE	-96
2p11.2e	925	CD8A	TRUE	-913
2p11.2e	925	CD8A	Shore	-698
2p11.2e	925	CD8A	TRUE	-20
2p11.2e	925	CD8A	TRUE	718
2p11.2e	925	CD8A	TRUE	-898
2p11.2e	925	CD8A	TRUE	-75
2p11.2e	925	CD8A	TRUE	207
2p11.2e	925	CD8A	TRUE	-1
2p11.2e	925	CD8A	TRUE	165
2p11.2e	925	CD8A	TRUE	58
2p11.2e	925	CD8A	TRUE	449
2p11.2e	925	CD8A	Shore	-1147
2p11.2e	925	CD8A	TRUE	-21995995
2p11.2e	925	CD8A	TRUE	9
2p11.2e	925	CD8A	Shore	-1077
2p11.2e	925	CD8A	Shore	-305
24145649-24	2999	GZMH	TRUE	161
2p11.2e	925	CD8A	TRUE	77
2p11.2e	925	CD8A	TRUE	274
2p11.2e	925	CD8A	TRUE	-396
24145649-24	2999	GZMH	TRUE	-102
2p11.2e	925	CD8A	TRUE	62
2p11.2e	925	CD8A	TRUE	377
2p11.2e	925	CD8A	TRUE	9
2p11.2e	925	CD8A	TRUE	402
2p11.2e	925	CD8A	TRUE	0
2p11.2e	925	CD8A	TRUE	136
2p11.2e	925	CD8A	Shore	758
2p11.2e	925	CD8A	TRUE	-22
2p11.2e	925	CD8A	TRUE	435
2p11.2e	925	CD8A	TRUE	226
2p11.2e	925	CD8A	TRUE	352

2p11.2e	925	CD8A	TRUE	-54
2p11.2e	925	CD8A	TRUE	-100
2p11.2e	925	CD8A	TRUE	169
2p11.2e	925	CD8A	TRUE	-638
2p11.2e	925	CD8A	TRUE	-390
2p11.2e	925	CD8A	TRUE	252
2p11.2e	925	CD8A	TRUE	209
2p11.2e	925	CD8A	Shore	-698
2p11.2e	925	CD8A	TRUE	338
2p11.2e	925	CD8A	FALSE	0
2p11.2e	925	CD8A	Shore	-605
2p11.2e	925	CD8A	TRUE	279
2p11.2e	925	CD8A	TRUE	268
2p11.2e	925	CD8A	TRUE	338
2p11.2e	925	CD8A	Shore	-46
2p11.2e	925	CD8A	TRUE	226
2p11.2e	925	CD8A	TRUE	-905
2p11.2e	925	CD8A	TRUE	667
2p11.2e	925	CD8A	TRUE	182
2p11.2e	925	CD8A	Shore	-1147
2p11.2e	925	CD8A	TRUE	-396
2p11.2e	925	CD8A	TRUE	516
2p11.2e	925	CD8A	TRUE	-353
2p11.2e	925	CD8A	TRUE	-258
2p11.2e	925	CD8A	TRUE	-72
2p11.2e	925	CD8A	TRUE	165
2p11.2e	925	CD8A	TRUE	-357
2p11.2e	925	CD8A	Shore	315
2p11.2e	925	CD8A	TRUE	163
2p11.2e	925	CD8A	Shore	-542
2p11.2e	925	CD8A	TRUE	455
2p11.2e	925	CD8A	TRUE	15
24145649-24	2999	GZMH	TRUE	272
2p11.2e	925	CD8A	TRUE	-65
2p11.2e	925	CD8A	TRUE	-1395
2p11.2e	925	CD8A	TRUE	-20
2p11.2e	925	CD8A	Shore	-739
2p11.2e	925	CD8A	TRUE	-300
2p11.2e	925	CD8A	TRUE	455
2p11.2e	925	CD8A	TRUE	-139
2p11.2e	925	CD8A	TRUE	-913
2p11.2e	925	CD8A	TRUE	62
2p11.2e	925	CD8A	TRUE	-898
2p11.2e	925	CD8A	Shore	-1077

2p11.2e	925	CD8A	TRUE	-461
2p11.2e	925	CD8A	TRUE	8
2p11.2e	925	CD8A	Shore	-46
2p11.2e	925	CD8A	TRUE	21
2p11.2e	925	CD8A	TRUE	197
2p11.2e	925	CD8A	TRUE	24
2p11.2e	925	CD8A	TRUE	-139
2p11.2e	925	CD8A	TRUE	-905
2p11.2e	925	CD8A	TRUE	92
2p11.2e	925	CD8A	TRUE	184
2p11.2e	925	CD8A	TRUE	58
2p11.2e	925	CD8A	TRUE	516
2p11.2e	925	CD8A	TRUE	21
2p11.2e	925	CD8A	TRUE	466
2p11.2e	925	CD8A	TRUE	515
2p11.2e	925	CD8A	TRUE	318
2p11.2e	925	CD8A	Shore	457
2p11.2e	925	CD8A	TRUE	197
2p11.2e	925	CD8A	TRUE	-258
2p11.2e	925	CD8A	TRUE	298
2p11.2e	925	CD8A	Shore	-739
2p11.2e	925	CD8A	Shore	-334
2p11.2e	925	CD8A	TRUE	-300
2p11.2e	925	CD8A	TRUE	413
2p11.2e	925	CD8A	TRUE	-72
2p11.2e	925	CD8A	TRUE	72
2p11.2e	925	CD8A	TRUE	466
1p36.11b	257101	ZNF683	TRUE	272
2p11.2e	925	CD8A	TRUE	506
2p11.2e	925	CD8A	TRUE	184
24145649-24	2999	GZMH	TRUE	442
2p11.2e	925	CD8A	TRUE	-638
2p11.2e	925	CD8A	Shore	-1262
2p11.2e	925	CD8A	TRUE	-161
2p11.2e	925	CD8A	TRUE	352
2p11.2e	925	CD8A	TRUE	577
2p11.2e	925	CD8A	TRUE	667
2p11.2e	925	CD8A	TRUE	340
2p11.2e	925	CD8A	TRUE	-353
2p11.2e	925	CD8A	TRUE	519
2p11.2e	925	CD8A	TRUE	-461
2p11.2e	925	CD8A	TRUE	298
2p11.2e	925	CD8A	TRUE	519
24145649-24	2999	GZMH	TRUE	-28

2p11.2e	925	CD8A	Shore	36
2p11.2e	925	CD8A	TRUE	413
2p11.2e	925	CD8A	TRUE	-499
2p11.2e	925	CD8A	TRUE	-19123427
2p11.2e	925	CD8A	TRUE	340
2p11.2e	925	CD8A	Shore	-1262
2p11.2e	925	CD8A	TRUE	-357
2p11.2e	925	CD8A	TRUE	72
2p11.2e	925	CD8A	TRUE	35110189
2p11.2e	925	CD8A	Shore	35109904
2p11.2e	925	CD8A	Shore	-334
2p11.2e	925	CD8A	TRUE	-499
2p11.2e	925	CD8A	TRUE	467
2p11.2e	925	CD8A	TRUE	28
2p11.2e	925	CD8A	Shore	36
2p11.2e	925	CD8A	TRUE	-385
2p11.2e	925	CD8A	TRUE	37
2p11.2e	925	CD8A	TRUE	-459
1p36.11b	257101	ZNF683	TRUE	442
2p11.2e	925	CD8A	TRUE	506
2p11.2e	925	CD8A	TRUE	183
2p11.2e	925	CD8A	TRUE	132
2p11.2e	925	CD8A	TRUE	222
2p11.2e	925	CD8A	TRUE	577
24145649-24	2999	GZMH	FALSE	-31
2p11.2e	925	CD8A	Shore	-975
2p11.2e	925	CD8A	Shore	35109904
2p11.2e	925	CD8A	TRUE	467
2p11.2e	925	CD8A	TRUE	37
2p11.2e	925	CD8A	TRUE	222
2p11.2e	925	CD8A	TRUE	3557864
2p11.2e	925	CD8A	TRUE	-385
2p11.2e	925	CD8A	TRUE	35110189
2p11.2e	925	CD8A	TRUE	-19123427
24145649-24	2999	GZMH	TRUE	465
24145649-24	2999	GZMH	TRUE	94
2p11.2e	925	CD8A	Shore	-975
2p11.2e	925	CD8A	TRUE	28
2p11.2e	925	CD8A	TRUE	591
24145649-24	2999	GZMH	TRUE	-691
2p11.2e	925	CD8A	TRUE	591
1p36.11b	257101	ZNF683	FALSE	-31
2p11.2e	925	CD8A	TRUE	11
24145649-24	2999	GZMH	TRUE	435

2p11.2e	925	CD8A	TRUE	11
2p11.2e	925	CD8A	TRUE	274
1p36.11b	257101	ZNF683	TRUE	312
1p36.11b	257101	ZNF683	TRUE	-146
1p36.11b	257101	ZNF683	TRUE	252
2p11.2e	925	CD8A	TRUE	274
1p36.11b	257101	ZNF683	TRUE	340
1p36.11b	257101	ZNF683	TRUE	-249
24145649-24	2999	GZMH	TRUE	577
1p36.11b	257101	ZNF683	TRUE	390
1p36.11b	257101	ZNF683	Shore	-334
