# Supplementary material for: Genetic analysis of DNA methylation and gene expression levels in whole blood of healthy human subjects
Source: BMC Genomics. 2012 Nov 17;13:636. doi: 10.1186/1471-2164-13-636 (PMC3583143; doi:10.1186/1471-2164-13-636)
Supplement: Additional file 2 — Figure S1. Are two figures that show the coefficient and explained variance of associations between methylation and expression. [file 1471-2164-13-636-S2.pdf]

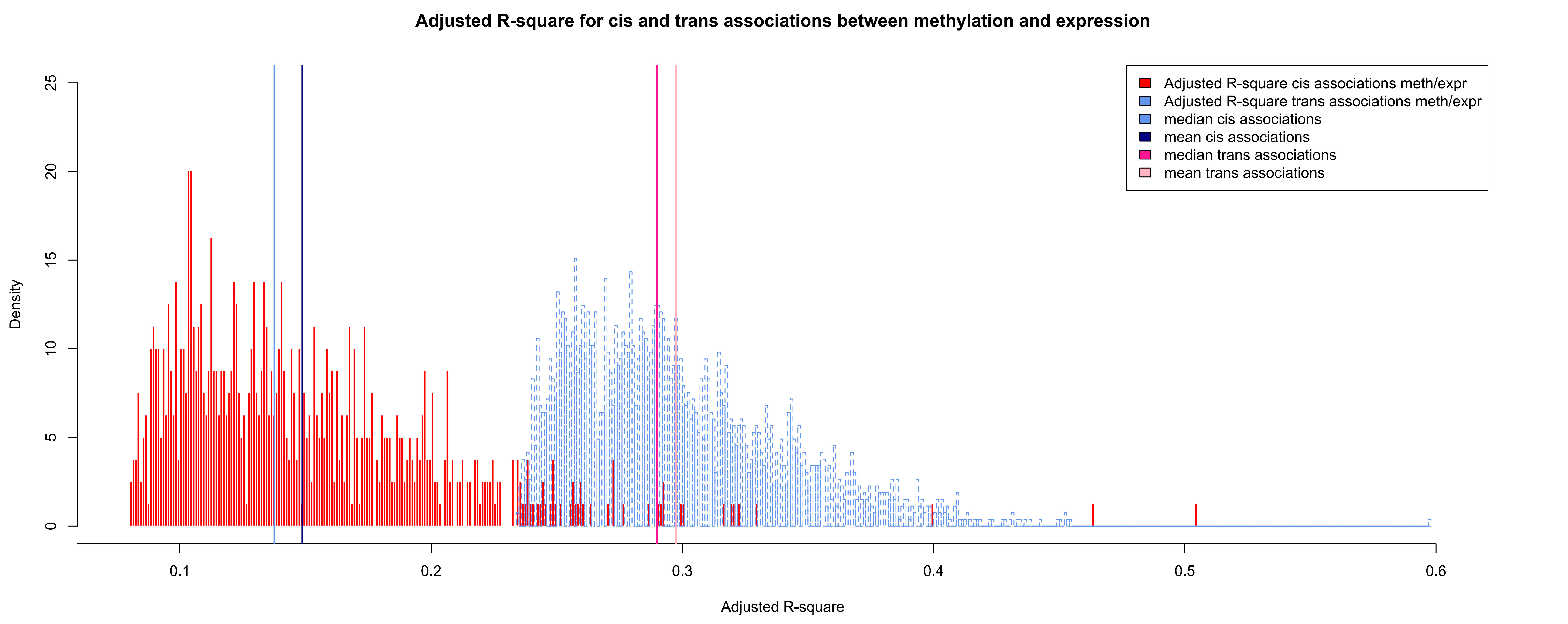
Adjusted R-square for cis and trans associations between methylation and expression
Density
Adjusted R-square
0
5
10
15
20
25
0.1
0.2
0.3
0.4
0.5
0.6
Adjusted R-square cis associations meth/expr
Adjusted R-square trans associations meth/expr
median cis associations
mean cis associations
median trans associations
mean trans associations

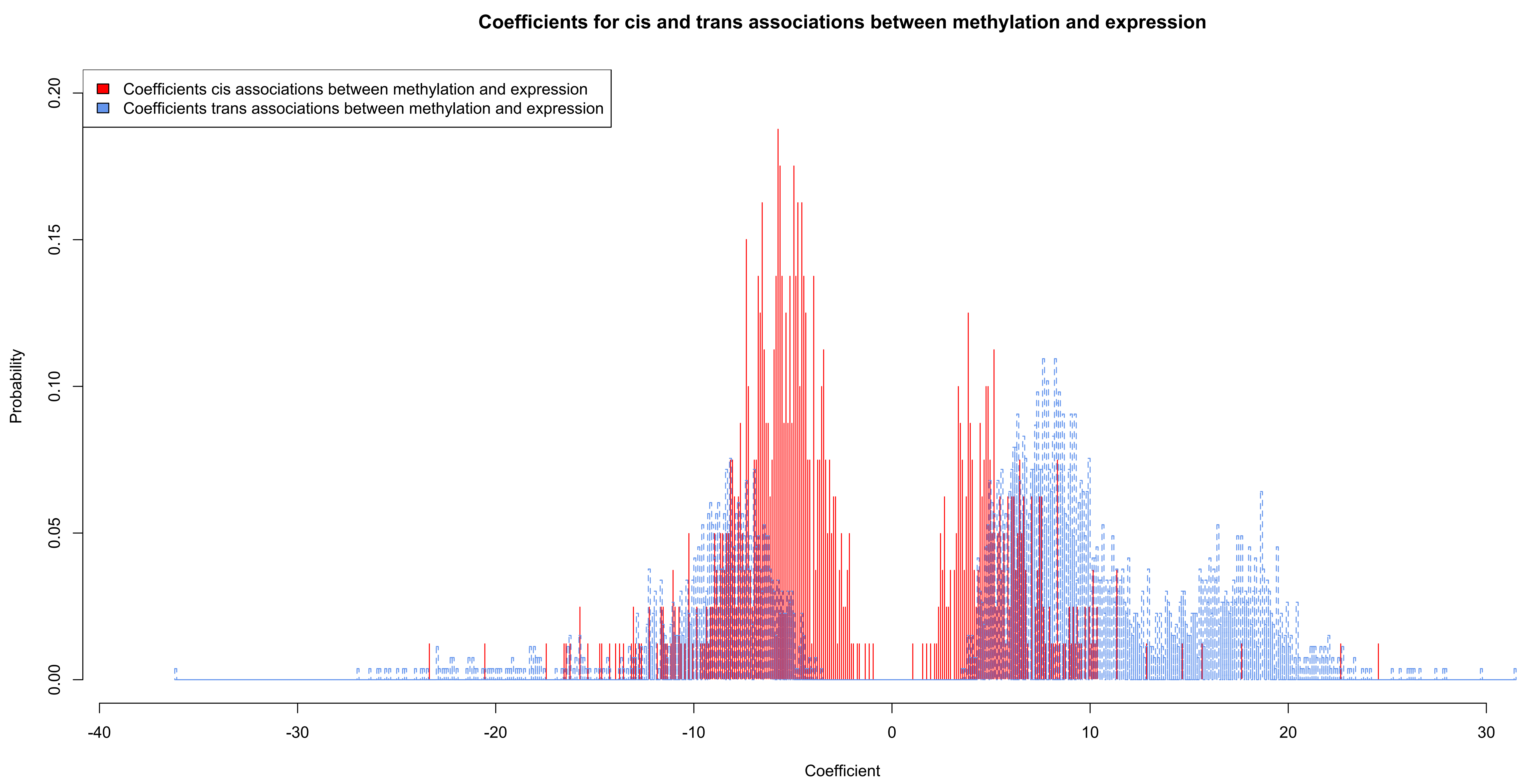

Coefficients for cis and trans associations between methylation and expression
Coefficients cis associations between methylation and expression
Coefficients trans associations between methylation and expression
Probability
0.00
0.05
0.10
0.15
0.20
-40
-30
-20
-10
0
10
20
30
Coefficient
