# Supplementary material for: Genetic analysis of DNA methylation and gene expression levels in whole blood of healthy human subjects
Source: BMC Genomics. 2012 Nov 17;13:636. doi: 10.1186/1471-2164-13-636 (PMC3583143; doi:10.1186/1471-2164-13-636)
Supplement: Additional file 5 — Contains supplementary methods, namely, a more detailed description of Weighted Correlation Network Analysis (WGCNA). [file 1471-2164-13-636-S5.pdf]

Supplementary Methods: Weighted Correlation Network Analysis

Peter Langfelder and Steve Horvath*
*Corresponding author: shorvath@mednet.ucla.edu

Contents

1 Collapsing probe-level expression and methylation data to gene-level data **1**

2 Weighted Correlation Network Analysis **1**

3 Summarizing module profiles by the eigengene **2**

4 Male-female consensus modules **2**

5 Matching expression and methylation modules **2**

6 Continuous measure of module membership **2**

7 Module preservation statistics **3**

1 Collapsing probe-level expression and methylation data to gene-level data

Comparing expression and methylation networks necessitates that the network nodes can be matched between expression and methylation networks. Since expression and methylation probe sets are very different, a simple approach is to aggregate ("collapse") the probe-level data to gene-level data. To this end we use the function `collapseRows` [6] that selects a representative probe for each gene represented on the microarray. For expression data we use the probe with highest mean expression while for methylation data we use the probe with highest variance. This procedure resulted in expression profiles for 13384 genes and methylation profiles for 13569 genes, of which 10483 are common.

We note that while the individual network analyses used the full sets of 13384 and 13569 genes, respectively, the preservation analysis only used the common subset of 10483 genes. Hence, the indicated numbers of genes in the modules are slightly different between the enrichment tables (Tables 4 and 5 in the main text) and the module preservation figures (Figures 2A, 2B, and the supplementary figure in Supplementary File 11).

2 Weighted Correlation Network Analysis

Weighted correlation network analysis [11] uses a pairwise measure of correlation (or more generally statistical association) among thousands of variables (for example, gene expression or methylation profiles across tens or hundreds of samples) to provide insights into the organization of the variables (for example, expressome or methylome). Weighted networks have the advantage of preserving the continuous nature of inter-variable associations that is lost in unweighted networks. In our calculations we use the WGCNA package [4] and the R language and environment for statistical computing [7].

Network construction starts by forming a pairwise similarity measure between all pairs of variables. Here we use the "signed hybrid" similarity that equals the pairwise correlation if the correlation is positive, and is zero otherwise. To arrive at the network adjacency, the pairwise similarity is raised to a power (typically between 5 and 7) to suppress low correlations that may be due to noise. This amounts to a soft-thresholding since high correlations are emphasized at

the expense of low correlations in a continuous manner. Thus, in the final network variables with negative correlations are considered unconnected, and variables with low positive correlations have near-zero adjacency. To minimize effects of possible outliers, we use a robust correlation measure, specifically the biweight midcorrelation [9] implemented in the WGCNA package.

One of the central aims of network analysis is to identify correlation modules, that is groups of highly correlated genes. We use the Topological Overlap-based dissimilarity [8,10] measure as input to average-linkage hierarchical clustering. Modules are identified as branches in the resulting hierarchical clustering tree using the Dynamic Tree Cut [3]. Since modules are identified from the data in an unsupervised fashion, it can be useful to ascertain their possible biological interpretation by enrichment in known gene ontologies or pathways. We calculated enrichment in Gene Ontology (GO [1] as of March 20, 2010, `GO.db` R package version 2.4.1) terms using the function `GOenrichmentAnalysis` contained in the WGCNA package. This function performs the Fisher exact test on overlaps of modules and GO terms (plus their GO offspring), with the background given by the intersection of the list of genes in the analysis and all genes known to GO. Obtained p-values are corrected for the number of known GO terms (several thousand), but not for the number of modules (which is much smaller).

3 Summarizing module profiles by the eigengene

Since modules group together highly correlated variables, it is advantageous to summarize the variable profiles using a single representative. We use the module eigengene (or more generally eigennode) E, defined as the first principal component of the standardized matrix containing the variables in the module. The module eigengene can be intuitively understood as a weighted average of the variable profiles in the module.

Module analysis can be also viewed as a data reduction method in which entire modules consisting of anywhere between tens and thousands of genes are reduced to single module eigengene profiles. Correlation relationships among modules can then be easily quantified using the correlations of their eigengenes.

4 Male-female consensus modules

Both expression and methylation exhibit gender effects that can in some cases be responsible for string correlations of groups of genes and thus modules. To counter this possibility we used the consensus module approach described in [2] to construct consensus modules between male and female samples (separately in expression and methylation data). By definition, the consensus modules group together genes that are strongly correlated separately in male and in female samples. Thus, apart from protecting against modules driven by gender differences, the consensus module approach also results in modules can be considered "validated" since they are present in both input data sets (male and female). Although male-female consensus modules are derived by splitting the data into male and female data sets, the resulting module assignment can be carried back to the combined data set.

5 Matching expression and methylation modules

For easier interpretation of the relationships between expression and methylation modules, we use the same module labels for modules that show significant overlap. The matching of module labels was performed using the function matchLabels from the WGCNA R package; it is based on significance of module overlaps quantified using Fishers exact test. Briefly, given a reference and target set of labels, the function calculates the overlaps and significance (by Fishers exact test) for each pair of unique labels. It then matches target labels to the reference labels one by one, starting from the most significant overlap and only taking into account overlaps whose significance (p-value) is stronger than 0.05.

6 Continuous measure of module membership

Module eigengenes lead to a natural measure of similarity (membership) of all individual genes to all modules. We define a fuzzy measure of module membership of gene i in module q, as $kME_i = \text{cor}(x_i, E^q)$, where x_i is the profile of gene i and E^q is the eigengene of module q. The value of module membership lies between -1 and 1. The higher kME_i, the more similar the expression profile of gene i is to the summary profile of module q. Since we use signed

networks here, we consider module membership near -1 low. The advantage of using a correlation to quantify module membership is that the corresponding statistical significance (p-values) can be easily computed. Genes represented by probes with highest module membership are called hub genes. Hub genes are centrally located inside the module and represent the expression profiles of the entire module.

7 Module preservation statistics

To assess the preservation of expression and methylation modules in the corresponding complementary data set, we use the network module preservation statistics described in [5] and implemented in the function `modulePreservation` in the WGCNA R package. Network module preservation statistics assess whether the density and connectivity patterns of modules defined in a reference data set are preserved in a test data set. Network preservation statistics do not require that modules be identified in the test data set and hence independent of the ambiguities associated with module identification in the test data set.

Calculation of module preservation statistics requires that the genes in each network are the same (in other words, genes present in only one network must be removed). Matching expression and methylation genes by their Entrez ID led to a common set of 10483 genes.

Although it is useful to study all statistics to determine which module properties are preserved, it is practical to consider summary statistics that summarize the evidence for preservation of each module. Here we report the $Z_{summary}$ statistic that summarizes evidence that a module is preserved more significantly than a random sample of all network genes. We use the thresholds proposed by [5]: $Z_{summary} < 2$ implies no evidence for module preservation, $2 < Z_{summary} < 10$ implies weak to moderate evidence, and $Z_{summary} < 10$ implies strong evidence for module preservation. Thus, we report $Z_{summary}$ for each expression and methylation module in the methylation and expression test data sets, respectively.

References

1. Michael Ashburner, Catherine A. Ball, Judith A. Blake, David Botstein, Heather Butler, J. Michael Cherry, Allan P. Davis, Kara Dolinski, Selina S. Dwight, Janan T. Eppig, Midori A. Harris, David P. Hill, Laurie Issel-Tarver, Andrew Kasarskis, Suzanna Lewis, John C. Matese, Joel E. Richardson, Martin Ringwald, Gerald M. Rubin, and Gavin Sherlock. Gene ontology: tool for the unification of biology. *Nat Genet*, 25:25–29, 2000.

2. P Langfelder and S Horvath. Eigengene networks for studying the relationships between co-expression modules. *BMC Systems Biology*, 1(1):54, 2007.

3. P Langfelder, B Zhang, and S Horvath. Defining clusters from a hierarchical cluster tree: the Dynamic Tree Cut library for R. *Bioinformatics*, 24(5):719–20, 2007.

4. Peter Langfelder and Steve Horvath. WGCNA: an R package for weighted correlation network analysis. *BMC Bioinformatics*, 9(1):559, 2008.

5. Peter Langfelder, Rui Luo, Michael C. Oldham, and Steve Horvath. Is my network module preserved and reproducible? *PLoS Comput Biol*, 7(1):e1001057, 01 2011.

6. Jeremy Miller, Chaochao Cai, Peter Langfelder, Daniel Geschwind, Sunil Kurian, Daniel Salomon, and Steve Horvath. Strategies for aggregating gene expression data: The collapserows r function. *BMC Bioinformatics*, 12(1):322, 2011.

7. R Development Core Team. *R: A Language and Environment for Statistical Computing.* R Foundation for Statistical Computing, Vienna, Austria, 2010. ISBN 3-900051-07-0.

8. E Ravasz, A L Somera, D A Mongru, Z N Oltvai, and A L Barabasi. Hierarchical organization of modularity in metabolic networks. *Science*, 297(5586):1551–5, 2002.

9. Rand R. Wilcox. *Introduction to Robust Estimation and Hypothesis Testing, 2nd ed.* Academic Press, 2005.

10. A Yip and S Horvath. Gene network interconnectedness and the generalized topological overlap measure. *BMC Bioinformatics*, 8(8):22, 2007.

11. B Zhang and S. Horvath. General framework for weighted gene coexpression analysis. *Statistical Applications in Genetics and Molecular Biology.*, 4(17), 2005.
