# Supplementary material for: Genetic analysis of DNA methylation and gene expression levels in whole blood of healthy human subjects
Source: BMC Genomics. 2012 Nov 17;13:636. doi: 10.1186/1471-2164-13-636 (PMC3583143; doi:10.1186/1471-2164-13-636)
Supplement: Additional file 6 — Is an overview of the modules identified in the expression (Table 1) and methylation (Table 2) data. [file 1471-2164-13-636-S6.pdf]

Supplementary Tables:
Overview of expression and methylation modules

Here we provide a basic overview of all modules identified in the expression and methylation data. Tables below list the module label, module size (number of genes in each module), and the top 10 hub genes (genes with the highest module membership *kME*). A complete list of all genes in each module can be found in Supplementary Tables S7 and S8. Functional enrichment (where significant) is summarized in Tables 4 and 5 in the main article.

Module	Size	Top 10 hub genes
1	1520	ZNF800, TMED5, SNRK, ARGLU1, PNRC2, RASA1, RPS6KB1, RYK, REEP5, FUBP3
2	703	YIF1A, ZNF526, CUL9, APEH, ARL6IP4, WDR18, MRPS12, NFATC2IP, TSR2, PRPF3
3	658	ADPRH, LILRA4, TIMM13, VWA5A, FLJ46836, TRIM62, CXXC4, B4GALT2, CD84, SLC28A1
4	647	TRIM28, DAXX, PIP5K1C, GMPPA, ARHGDIA, NDUFV1, SUPT5H, CENPT, DPP9, IKBKE
5	457	CBX3, C20orf132, RTF1, PHLDB1, FAM116B, BDKRB1, FZD7, ACPT, NR1D1, FEZ1
6	442	DENND5A, FPR2, IFNGR2, AQP9, LRMP, MXD1, TM6SF1, PTPRE, STX3, STEAP4
7	426	CANT1, ARID3A, PHC2, IMPDH1, C3orf62, NCF4, PFKFB4, DYSF, ZDHHC18, REPS2
8	407	ABI1, TMED2, METTL23, GNL3, HSPA9, IRF2BP2, COA5, NSUN2, SET, PIGY
9	387	CDKN1B, PTPLB, SACM1L, ATP11B, LUC7L3, CLIP1, SMARCA2, YY1, CUL4A, PTBP3
10	355	WBSCR22, TXLNA, BOP1, GPS1, NT5C, JMJD8, ARL2, MRPL37, PSMC3, C17orf70
11	322	DMC1, PPM1K, CYCSP55, PLA2G2D, DTWD2, IL17RD, GSTTP2, SSTR2, ZNF577, DENR
12	306	EPRS, DNAJC9, RPSA, MDH1, POLE3, NSMCE4A, CCDC25, PCID2, RPL12, RPAIN
13	260	KLF1, GATA1, ST6GALNAC4, SLC6A10P, MARCH2, MCOLN1, SLC6A8, TNS1, MAF1, DPM2
14	237	OGFR, CTSD, DNM2, MAP3K11, MKL1, RENBP, PCIF1, RIN3, TSC22D4, TAOK2
15	118	ST6GALNAC2, HLX, CXCR1, PAK1, C20orf3, PTAFR, LPPR2, SLC19A1, USP32, LPAR2
16	108	ECI2, EIF3M, PPIAP29, FKBP3, FRG1, HSPE1, PCNA, C14orf166, RPL23A, UQCRC2
17	99	SEC61G, PRICKLE4, NDUFS5, EXOSC1, MRPS33, ATP5O, POLE4, RPL24, UQCRH, NDUFAF2
18	72	CTDSPL, TSPAN9, CMTM5, PTCRA, ALOX12, LY6G6F, GP9, TREML1, ITGA2B, ITGB5
19	60	COX7A2, COX7C, UQCRQ, NDUFA4, TOMM7, RPL39, RPL41, RPS17, RPS27, SNRPG
20	52	IFI44L, IFI35, IFIT1, IFIT3, MX1, HERC5, XAF1, STAT1, RSAD2, EPSTI1
21	38	EGR3, CALHM1, LHX6, MLH3, GK2, DEFB131, POLR2M, ZNF93, DYNLRB2, GEMIN2
22	37	DNTTIP1, NFIL3, ABHD5, MANSC1, PANX2, PYGL, S100A11, HIST1H2BC, DGAT2, SLC25A44
23	32	VPREB3, PNOC, BLK, CXCR5, MIR600HG, FCRLA, CD19, CD72, CD79A, CD79B

Table 1. Overview of co-expression modules identified in our analysis.

Module	Size	Top 10 hub genes
1	1045	SPRY1, LSM6, NCOA7, FN1, ASPM, MYBL1, RARB, ALKBH8, CYTIP, DMTF1
2	105	DNTT, SIT1, FASLG, LCK, LTA, LY9, EVL, SLA2, CD3E, CD3G
3	427	ADAMTS13, CLCA1, C20orf160, C14orf166B, CELA3B, IGL@, LBP, ACR, EDDM3B, NA
7	594	ARID3A, AIF1, ALDH3B1, GPR97, FOLR3, TSPAN16, KCNE1, SERPINF1, FXYD1, FERMT3
12	130	TADA1, CLK2, TAMM41, GTF2I, HRASLS, RPL17, PBLD, TRPS1, RAD54L, SETDB1
19	37	MOSPD2, CENPI, MCTS1, ARMCX1, ATRX, FAM120C, MTMR8, RBM3, TCEAL8, AIFM1
25	67	NA, EDA, ELK1, LDOC1, PCSK1N, MPP1, PLP2, BEX1, TSR2, ZMYM3
30	616	PPP1R13L, GPR83, FBXO39, GRM7, CXCL1, ICAM4, FOXI2, RBP4, TP73, CRISPLD1
32	1067	STX12, BPIFC, BRD7, BLNK, SLC10A6, JAK1, RCN1, PIEZO2, NSUN3, HIATL1

Table 2. Overview of co-methylation modules identified in our analysis.
