# Supplementary figures and images for: Genetic analysis of DNA methylation and gene expression levels in whole blood of healthy human subjects
Source: BMC Genomics. 2012 Nov 17;13:636. doi: 10.1186/1471-2164-13-636 (PMC3583143; doi:10.1186/1471-2164-13-636)

# Overlap of expression and methylation modules

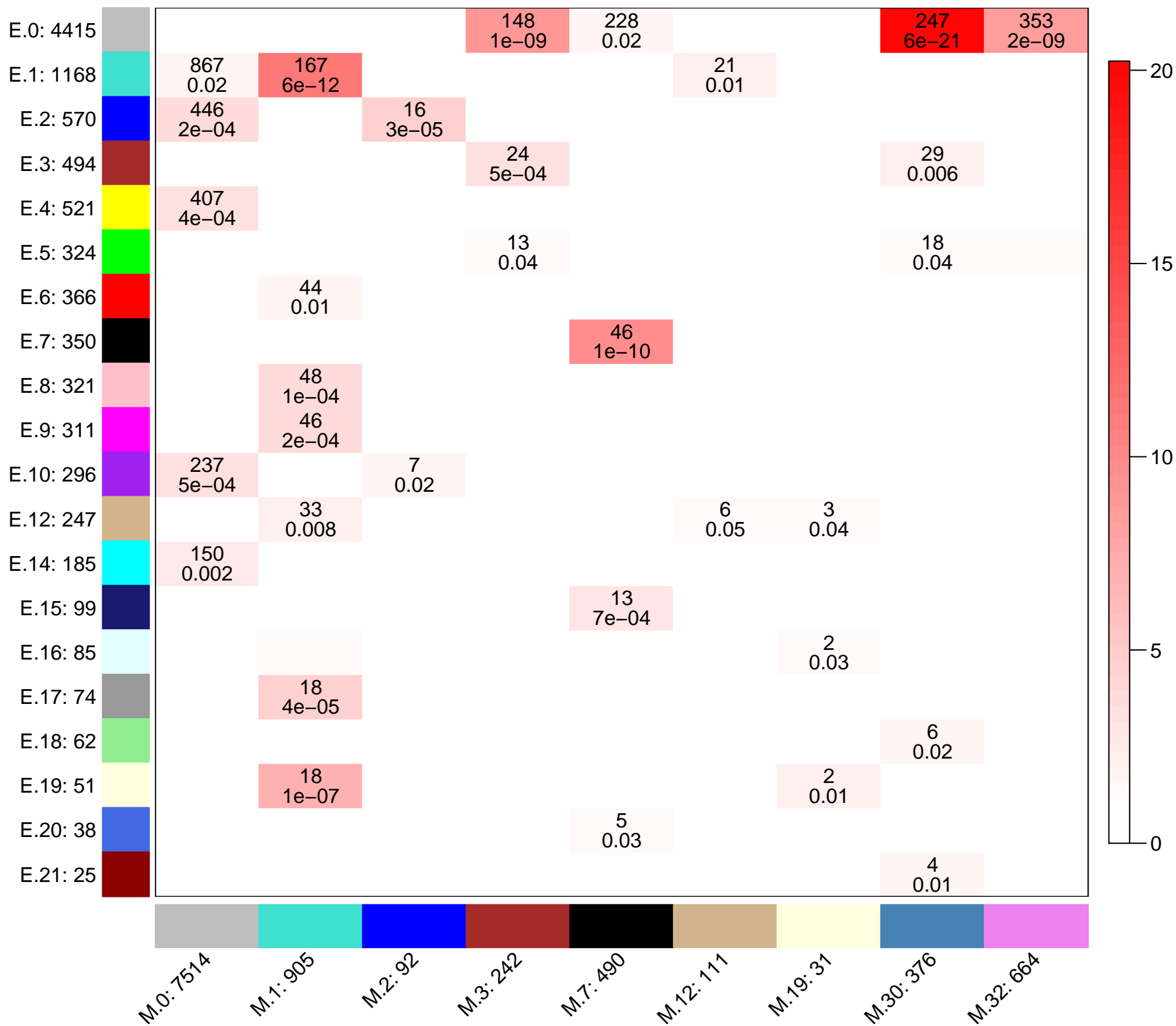

Supplement: Additional file 9 — Shows the overlap of expression and methylation modules. Each row corresponds to an expression module (labelled by the numeric labels, colours and total number of genes in the module, on the left), and each column corresponds to a methylation module (labelled the numeric labels, colours, and total number of genes in the module, at the bottom). Numbers in the table indicate number of genes in the overlap, and the Fisher exact test p-value for the overlap. Only overlaps whose p-value is below 0.05 are shown. The table is coloured such that significant overlaps are coloured in strong red colour. Most overlaps are quite small but some are nevertheless statistically highly significant. [file 1471-2164-13-636-S9.pdf]

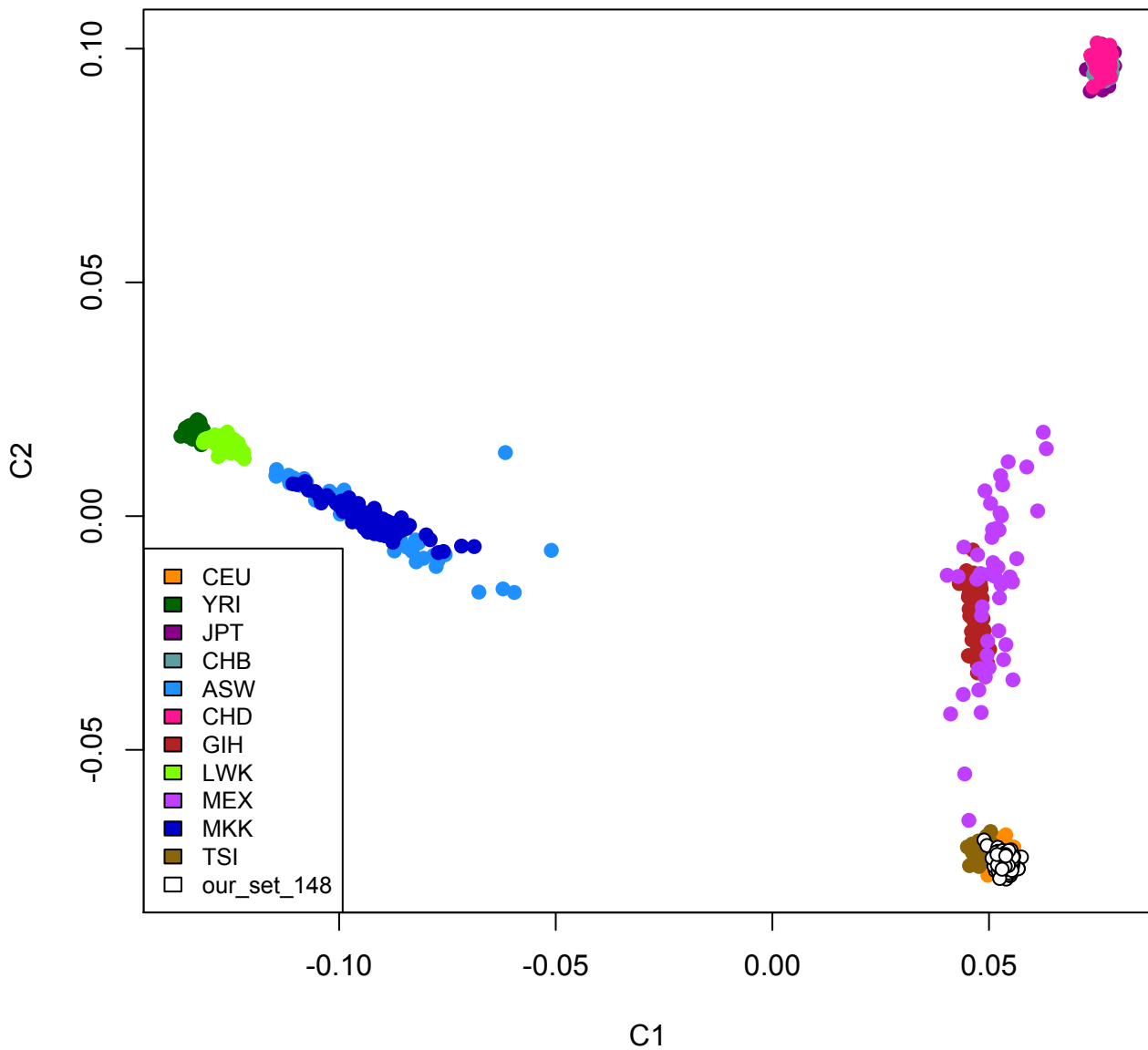

Supplement: Additional file 12 — Is a clusterplot of all samples together with Hapmap phase 3 populations. [file 1471-2164-13-636-S12.pdf]
