# Supplementary material for: Genetic analysis of DNA methylation and gene expression levels in whole blood of healthy human subjects
Source: BMC Genomics. 2012 Nov 17;13:636. doi: 10.1186/1471-2164-13-636 (PMC3583143; doi:10.1186/1471-2164-13-636)
Supplement: Additional file 10 — Shows the medianRank statistics for the Module preservation with in (A) preservation of expression modules in methylation data, and in (B) preservation of methylation modules in expression data. [file 1471-2164-13-636-S10.pdf]

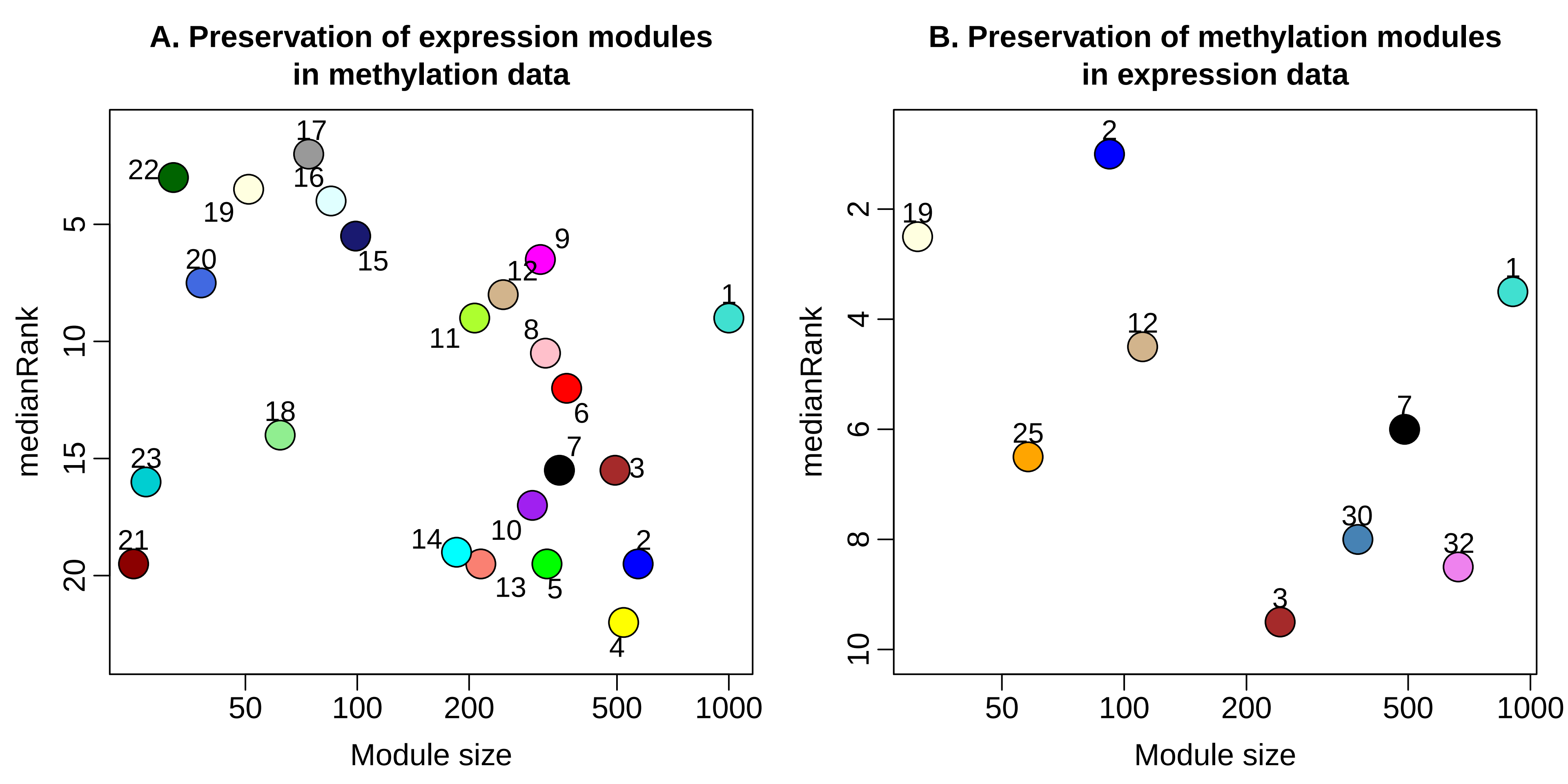

A. Preservation of expression modules
in methylation data
B. Preservation of methylation modules
in expression data
medianRank
Module size
5
10
15
20
2
4
6
8
10
50
100
200
500
1000
1
2
3
4
5
6
7
8
9
10
11
12
13
14
15
16
17
18
19
20
21
22
23
25
30
32
